# Analysis of gender- and age-stratified asthma burden: forecasting prevalence trends in 2030

**DOI:** 10.3389/fmed.2025.1612688

**Published:** 2025-08-20

**Authors:** Zhenzhen Pan, Hongye Yang, Yuting Jin, Qin Zhou, Qian Wang, Chuangli Hao, Ling Li

**Affiliations:** ^1^Department of Respiratory Medicine, Children’s Hospital of Soochow University, Suzhou, China; ^2^Department of Respiratory Medicine & Clinical Allergy Center, Affiliated Children’s Hospital of Jiangnan University, Wuxi, China; ^3^Department of Pediatric, Jiangyin No. 3 People’s Hospital, Wuxi, China; ^4^Department of Pediatrics, The First Affiliated Hospital of Shandong First Medical University, Jinan, China

**Keywords:** asthma, GBD, estimated annual percentage change, socio-demographic index, age

## Abstract

**Background:**

Asthma poses a global health challenge, requiring an understanding of its burden to guide policy. Using GBD 2021 data, this study aims to assess the burden of asthma worldwide.

**Methods:**

We extracted data on asthma prevalence, incidence, years lived with disability (YLDs), and disability-adjusted life-years (DALYs) from GBD for the period 1990–2021. Age-standardized rates (ASR) and estimated annual percentage changes (EAPC) were calculated to understand trends. We analyzed variations in asthma burden across gender and age groups, and explored the correlation between socio-demographic index (SDI) and asthma burden. Furthermore, we examined the main risk factors contributing to asthma. Lastly, we predicted the future asthma burden over the next 8 years.

**Results:**

High population countries such as India and China reported higher numbers of prevalence, incidence, and YLDs. From 1990 to 2021, the ASR of prevalence, incidence, and YLDs for asthma showed an overall downward trend. Children under 14 years of age demonstrated notably higher incidence rates, with the highest concentrations observed among those below 5 years, whereas the elderly population (>90 years) exhibited the peak prevalence and disability-adjusted life years (DALYs) burden. Gender differences were observed, with males having a higher burden before age 15–19, and females afterward. In China, smoking emerged as a significant risk factor for men, while the risk associated with high body mass index (BMI) has increased notably in recent years. Both incidence and prevalence in China are projected to decrease in the future.

**Conclusion:**

This study finds 15–19 years is a key turning point for gender differences in asthma burden, pinpointing smoking, high BMI, and NO₂ as risk factors. From 2022–2030, asthma prevalence/incidence is set to decline overall but rise in 15–19-year-olds, highlighting adolescence as a new prevention focus and a call for better health education in China.

## 1 Introduction

Asthma, a heterogeneous condition primarily characterized by chronic airway inflammation, presents with symptoms such as wheezing, shortness of breath, chest tightness, and cough, which vary in intensity and are accompanied by expiratory airflow limitation (1). As a significant global health challenge, it affects individuals of all ages, with the burden varying across different regions, genders, and age groups, particularly impacting residents in low socioeconomic areas. Beyond premature mortality, asthma often coexists with comorbidities like allergic rhinitis, gastroesophageal reflux disease, obstructive sleep apnea, and anxiety disorders (2, 3), thereby substantially impairing quality of life and imposing economic burdens. While asthma prevalence has stabilized or declined in some regions, it continues to rise in others, potentially due to differing distributions of risk factors (4, 5). To explore the burden of asthma across different genders and age groups and to provide a solid foundation for implementing targeted and effective asthma prevention and control measures, the following research endeavors are essential: Firstly, it is crucial to quantify the characteristics of asthma burden stratified by age and gender, so as to gain a comprehensive understanding of the actual conditions of various populations affected by asthma. Secondly, a systematic and in-depth investigation should be conducted into the associations between asthma prevalence, incidence rates, and risk factors (such as smoking and BMI), clarifying the roles these risk factors play in the variations of asthma prevalence and incidence rates, thereby offering vital clues for formulating preventive strategies. Lastly, establishing a scientifically sound disease prediction model and utilizing it to simulate the future epidemic trends of asthma will provide a scientific basis for devising precise prevention strategies tailored to population characteristics, socioeconomic conditions, and regional specificities. The Global Burden of Disease (GBD) database provides incidence, prevalence, and disability-adjusted life-years (DALYs) data for hundreds of diseases and injuries, enabling age-, sex-, region-, and year-specific analyses (6–8). This study utilized GBD data from 1990 to 2021 to evaluate asthma’s global disease burden, assess the impacts of age, gender, and socio-demographic index (SDI), and explore the influence of risk factors in China. Additionally, asthma incidence and prevalence trends in 2030 were predicted to inform prevention and control strategies.

## 2 Materials and methods

### 2.1 Data sources

Data were obtained from the GBD database, with asthma diagnoses based on ICD-10 codes J45 and J46 (8). Years lived with disability (YLDs), DALYs, prevalence, and incidence data were extracted. Age groups ranged from 5 years to 95+ years in 5-year intervals. Five SDI groups were defined: high SDI (>0.81), high-medium SDI (0.70–0.81), medium SDI (0.60–0.70), low-medium SDI (0.46–0.60), and low SDI (≤0.46). Asthma-related risk factors were also retrieved (9). Ethical approval was granted by the Wuxi Children’s Hospital (Approval Number: WXCH2023-09-056).

### 2.2 Variables

Prevalence, incidence, YLDs, and DALYs were employed as indicators. Age-standardized rates (ASRs) per 100,000 population were calculated for incidence (ASIR), prevalence (ASPR), and YLDs (ASYR). Estimated annual percentage change (EAPC) was computed to track trends from 1990 to 2021.

### 2.3 Burden of asthma in 204 countries worldwide

Asthma incidence, prevalence, and YLD data (1990, 2021) were spatially analyzed using R, with queen-neighbor matrices for statistical modeling. Topographic maps visualized global case distributions via ggplot2 (v3.5.1). Temporal trends (1990–2021) were assessed by calculating EAPCs for incidence, prevalence, and YLDs, with results visualized globally using ggplot2.

### 2.4 Regional asthma trends and socioeconomic associations

Age-standardized rates (ASIR, ASPR, ASYR) for asthma were stratified by gender across 21 GBD regions. Spearman correlations (cor package v0.8.3) evaluated relationships between SDI and asthma metrics, applying thresholds (|cor| >0.3, *p* < 0.05). Results were visualized via ggplot2.

### 2.5 Gender and age-specific asthma burden (2021)

Global asthma incidence, prevalence, and DALYs were analyzed by five-year age cohorts (5–95+ years) and sex using GBD 2021 data. The R package ggplot2 was used to plot a heatmap of DALY rates for ages 0–100 (grouped in 5-year intervals).

### 2.6 SDI and age-stratified asthma burden (2021)

Asthma metrics were further stratified by SDI levels and age groups to assess disparities in 2021.

### 2.7 Risk factor dynamics for asthma in China (1990–2021)

From 1990 to 2021, smoking, occupational asthmatogens, high body mass index (BMI), and NO_2_ pollution were identified as common potential risk factors associated with asthma in the GBD study. To assess the rankings of these factors in the prevalence of asthma among different genders in China, and the changes in the rankings from 1990–2021, an online analysis was conducted in the GBD Compare database.^1^

### 2.8 Global and Chinese asthma projections (2022–2030)

The core advantage of the Bayesian age-period-cohort (BAPC) model lies in its ability to simultaneously separate and quantify the age effect (inherent risk differences among different age groups), the period effect (impacts of external factors that change over time, such as medical policies and environmental changes), and the cohort effect (specific exposure differences among individuals in the same birth cohort). This advantage was highly compatible with the data structure of this study, so we employed this model to predict the incidence and prevalence of (the disease) globally and in China from 2022 to 2030. In order to understand the trend of global asthma disease burden from 2022 to 2030, global and Chinese incidence and prevalence data from 1990–2021, downloaded from the GBD, were applied to construct a BAPC model using the “forecast” package (v 8.23.0) and “tseries” package (v 0.10-58). Subsequently, to explore the impact of potential future interventions or unexpected events on the prevalence of asthma, using the R package “forecast,” the BAPC model was employed to simulate the following two scenarios: optimistic (assuming that starting from 2023, the prevalence rate would decrease annually due to tobacco control, emission reduction, and obesity intervention) and pessimistic (assuming that starting from 2025, extreme climate events would occur, leading to an annual increase in the prevalence rate). Finally, using the R package “tseries,” data from 1990 to 2010 were used as the training set to fit the BAPC model. The trends from 2011 to 2021 were predicted, and errors were calculated against the actual data.

### 2.9 Statistical analysis

Temporal trends in asthma incidence, prevalence, and YLDs were represented by the EAPC, which was originated from a model based on ASR and calendar years. The model was $\log_{10}(\mathrm{ASR})=\alpha +\beta *(\text{calendar year})+\varepsilon$ . The EAPC was calculated using the formula $\text{EAPC}=100*(10^{\beta }-1)$ . The 95% CI for EAPC was calculated. All data analyses and visualizations were conducted in R (v 4.2.2).

## 3 Results

### 3.1 Asthma burden had a correlation with national population size

**Figure 1:**
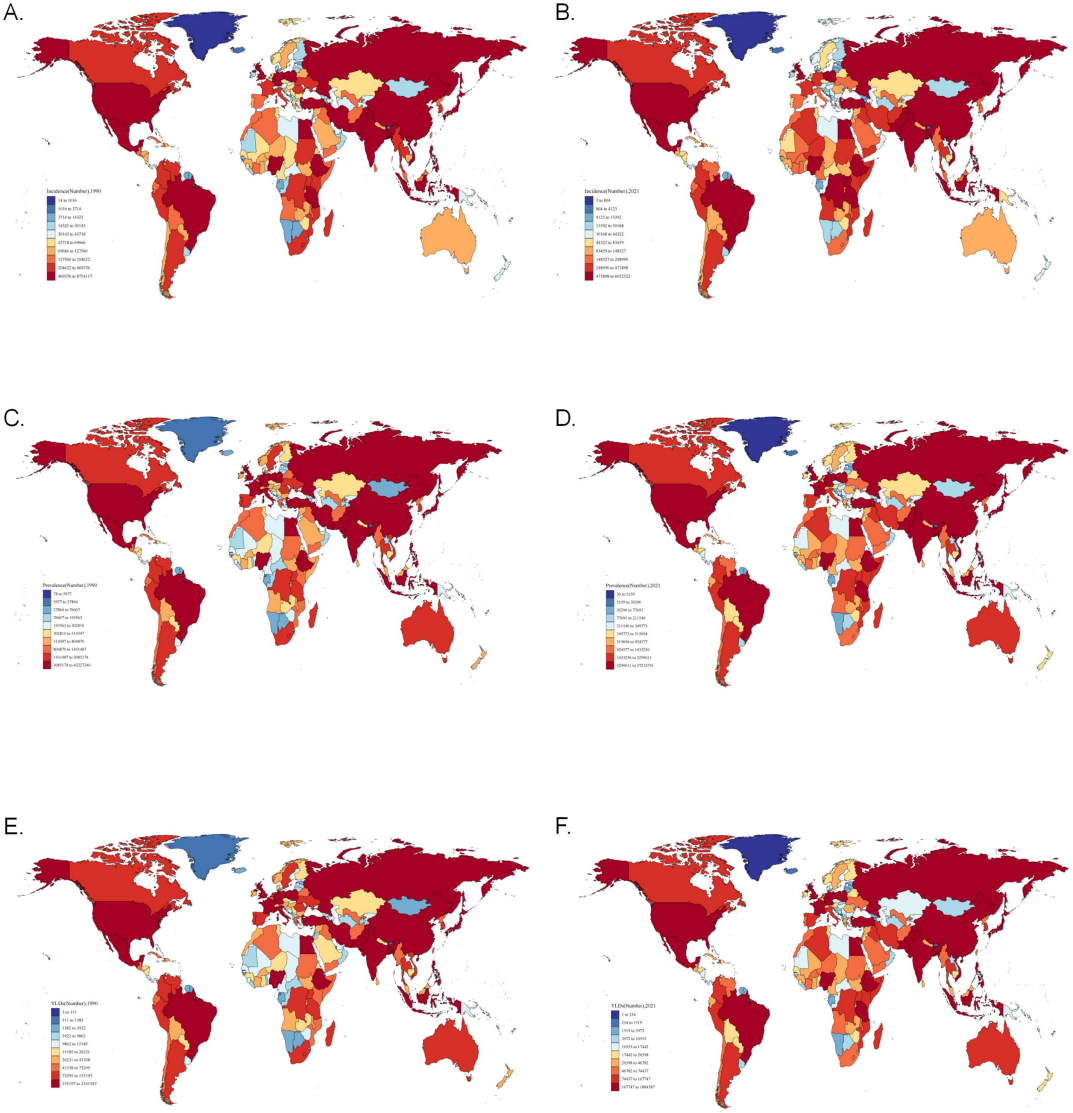
Asthma burden had a correlation with national population size. **(A)** Global incidence of asthma in 1990. **(B)** Global incidence of asthma in 2021. **(C)** Global prevalence of asthma in 1990. **(D)** Global prevalence of asthma in 2021. **(E)** Global YLDs of asthma in 1990. **(F)** Global YLDs of asthma in 2021. YLDs, incidence and years of disability.

Asthma incidence, prevalence, and YLDs correlated with national population size. India had the highest global caseloads in both 1990 [6.55 million (95% UI: 4.86–8.75)] and 2021 [4.90 million (4.06–6.05)], while Tokelau consistently reported the lowest (Figures 1A,B and Supplementary Tables 1, 2). For prevalence, the U.S. led in 2021 [33.33 million (29.84–37.21)], and India in 1990 [35.23 million (28.75–43.23)], with Tokelau again lowest (Figures 1C,D and Supplementary Tables 3, 4). YLDs followed similar patterns, with the U.S. highest in 2021 [1.28 million (0.84–1.88)] and India in 1990 [1.38 million (0.88–2.16)], and Tokelau lowest (Figures 1E,F and Supplementary Tables 5, 6).

Notably, while absolute case numbers aligned with population size, the most striking shifts in regional trends (map color gradients) occurred in Arab and African regions, indicating more pronounced changes in asthma burden compared to other areas.

### 3.2 Global ASPR for asthma showed an overall downward trend

#### 3.2.1 Prevalence (ASPR)

**Figure 2:**
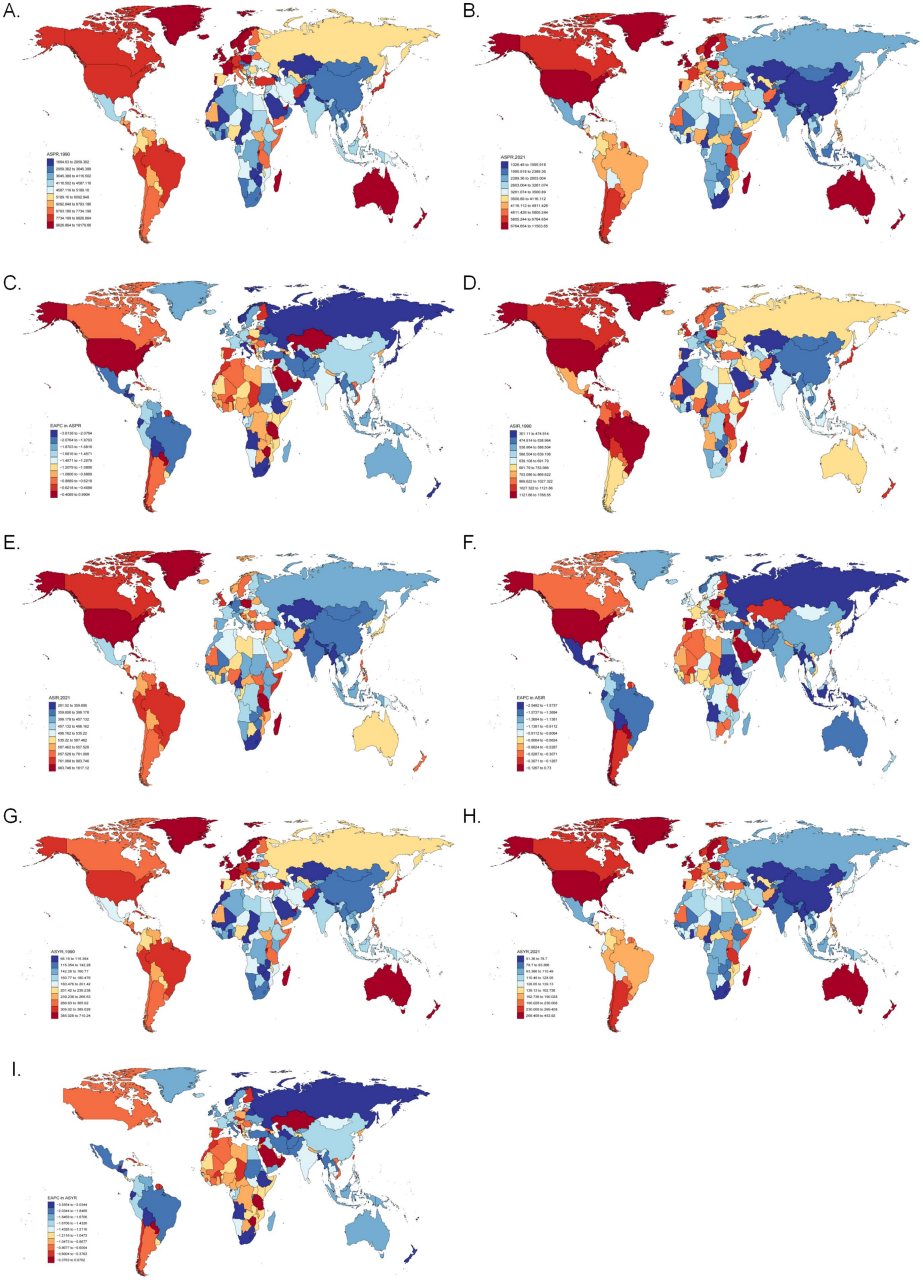
Global ASPR for asthma showed an overall downward trend. **(A)** Global ASPR for asthma in 1990. **(B)** Global ASPR for asthma in 2021. **(C)** Global EAPCs of prevalence for asthma. **(D)** Global ASIR for asthma in 1990. **(E)** Global ASIR for asthma in 2021. **(F)** Global EAPCs of incidence for asthma. **(G)** Global ASYR for asthma in 1990. **(H)** Global ASYR for asthma in 2021. **(I)** Global EAPCs of YLDs for asthma. ASPR, age-standardized prevalence rate; EAPC, estimated annual percentage change; ASIR, age-standardized incidence rate; ASYR, age-standardized YLDs rate; YLDs, incidence and years of disability.

New Zealand had the highest 1990 ASPR at 18179.66 (15448.46–21094.02) per 100,000, while Lesotho was lowest at 1664.63 (1481.95–1891.61) (Figure 2A and Supplementary Table 7). By 2021, Haiti reached the highest ASPR at 11503.65 (10476.15–12510.43), with Lesotho remaining lowest at 1326.48 (1182.91–1490.36) (Figure 2B). Globally, ASPR declined (EAPC trend), most steeply in Japan (−3.6138) and increased in the U.S. (0.99039) (Figure 2C and Supplementary Tables 8, 9).

#### 3.2.2 Incidence (ASIR)

Haiti led in 1990 ASIR at 1788.55 (1505.7–2159.51) per 100,000, with Kazakhstan lowest at 301.11 (249.15–379.11) (Figure 2D). By 2021, Haiti remained highest at 1617.12 (1355.96–1937.19), while Lesotho was lowest at 261.52 (224.56–304.25) (Figure 2E). ASIR declined globally, most notably in South Africa (−2.54823), but rose in the U.S. (0.72996) (Figure 2F and Supplementary Tables 10–12).

#### 3.2.3 YLDs (ASYR)

Mirroring ASPR patterns, New Zealand had the highest 1990 ASYR at 710.24 (456.95–1050.08) per 100,000, and Lesotho the lowest at 65.19 (42.56–96.98) (Figure 2G). By 2021, Haiti led at 453.92 (292.48–657.93), with Lesotho lowest at 51.36 (33.16–75.59) (Figure 2H). ASYR also declined globally, most steeply in Japan (−3.55544), but increased in the U. S. (0.97021) (Figure 2I and Supplementary Tables 13–15).

### 3.3 Asthma burden varied across gender populations in different GBD regions

#### 3.3.1 Prevalence (ASPR)

**Figure 3:**
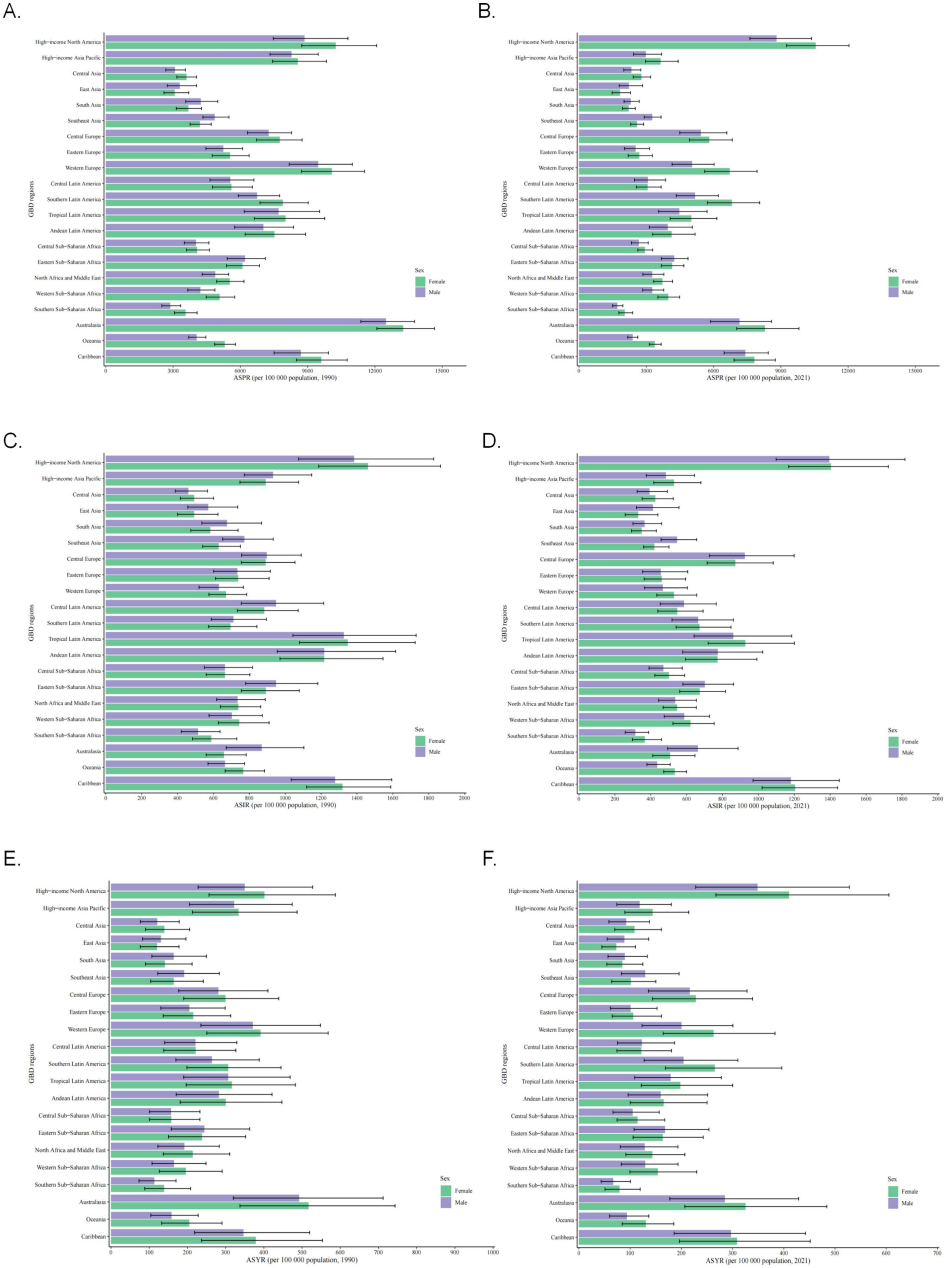
Asthma burden varied across gender populations in different GBD regions. **(A)** ASPR for asthma in different GBD regions in 1990. **(B)** ASPR for asthma in different GBD regions in 2021. **(C)** ASIR for asthma in different GBD regions in 1990. **(D)** ASIR for asthma in different GBD regions in 2021. **(E)** ASYR for asthma in different GBD regions in 1990. **(F)** ASYR for asthma in different GBD regions in 2021. ASPR, age-standardized prevalence rate; EAPC, estimated annual percentage change; ASIR, age-standardized incidence rate; ASYR, age-standardized YLDs rate; YLDs, incidence and years of disability.

In 1990, Australasia had the highest ASPR for both males and females, while high-income North America led in 2021 (Figures 3A,B and Supplementary Table 16). The lowest ASPR for males was consistently in Southern Sub-Saharan Africa, and for females, in East Asia across both periods.

#### 3.3.2 Incidence (ASIR)

High-income North America exhibited the highest ASIR for both genders in 1990 and 2021 (Figures 3C,D and Supplementary Table 17). The lowest ASIR for males shifted from Central Asia (1990) to Southern Sub-Saharan Africa (2021), while females remained lowest in East Asia.

#### 3.3.3 YLDs (ASYR)

Australasia had the highest ASYR for both genders in 1990, with high-income North America leading in 2021 (Figures 3E,F and Supplementary Table 18). The lowest ASYR for males was consistently in Southern Sub-Saharan Africa, and for females, in East Asia across both periods.

### 3.4 SDI and asthma burden correlation

**Figure 4:**
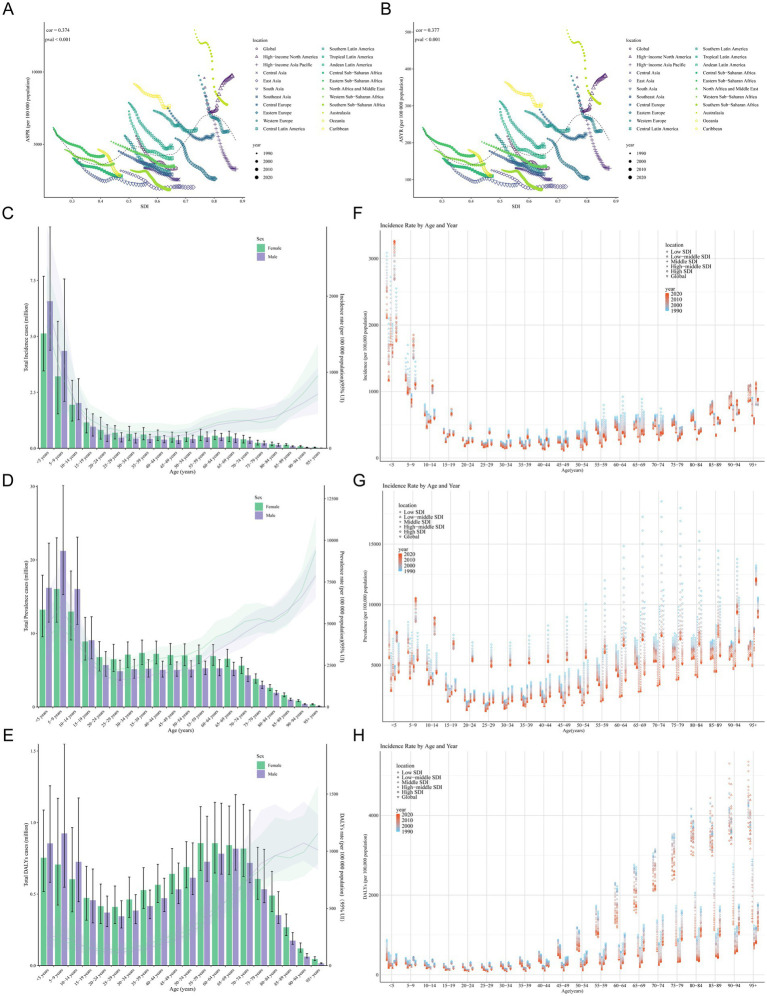
Asthma burden in different SDI regions and different age groups. **(A)** ASPR for asthma in different SDI regions. **(B)** ASYR for asthma in different SDI regions. **(C)** Incidence rate for asthma in different age groups. **(D)** Prevalence rate for asthma in different age groups. **(E)** DALYs for asthma in different age groups. **(F)** Incidence rate for asthma in different SDI regions by age and year. **(G)** Prevalence rate for asthma in different SDI regions by age and year. **(H)** DALYs for asthma in different SDI regions by age and year. ASPR, age-standardized prevalence rate; ASYR, age-standardized YLDs rate; SDI, socio-demographic index; DALYs, disability-adjusted life-years.

SDI exhibited a significant positive correlation (*p* < 0.001) with ASPR (cor = 0.374) and ASYR (cor = 0.377) (Figures 4A,B). As SDI increased, both ASPR and ASYR rose, underscoring the need for region-specific asthma management strategies tailored to varying SDI levels.

### 3.5 15–19 years of age was a critical time point

The prevalence is relatively high among those under 14 years old, with the highest rate observed in children under 5 (Figure 4C). Prevalence and DALYs peaked in older adults (>90 years). Notably, prevalence declined after age 5–9 and later resurged, while DALYs did not show early-life peaks (Figures 4D,E; Supplementary Figure 1). Age 15–19 emerged as a critical inflection point: males had higher prevalence, incidence, and DALYs before this age, with females surpassing males thereafter.

### 3.6 Asthma burden varied in different SDI regions by different age groups

In low-middle SDI regions, incidence peaked in the 75–94 age group, whereas other age groups showed higher incidence in high-SDI regions (Figure 4F). Prevalence was highest in high-SDI regions across all ages except under-5 years, where low-SDI regions led (Figure 4G). DALYs were highest in low-middle and low-SDI regions for those aged ≥35 years (Figure 4H).

### 3.7 The risk of high body mass index for the development of asthma risen significantly in recent years

**Figure 5:**
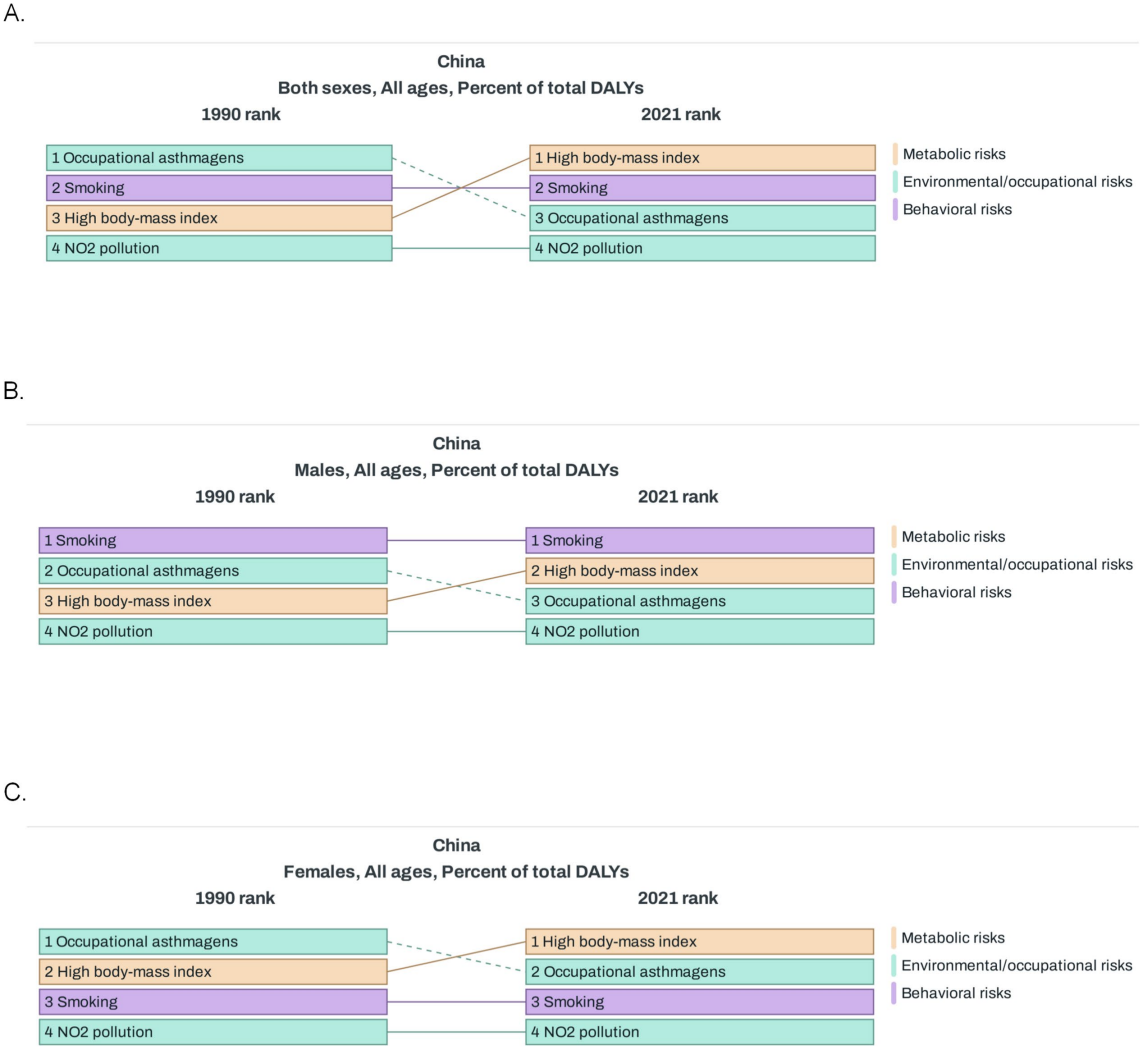
Risk factors of asthma in China. **(A)** Percent of total DALYs for risk factors of asthma in 1990 and 2021 (both sexes). **(B)** Percent of total DALYs for risk factors of asthma in 1990 and 2021 (males). **(C)** Percent of total DALYs for risk factors of asthma in 1990 and 2021 (females). DALYs, disability-adjusted life-years.

In China, smoking was pinpointed as the most prominent risk factor for asthma in both 1990 and 2021, playing a substantial role in the disease’s development (Figure 5A). Among Chinese men, when compared to the situation in 1990, high BMI climbed up the ranking of risk factors in 2021, overtaking occupational asthmagens (substances that can induce asthma) (Figure 5B). For Chinese women, smoking held the third position in the risk factor hierarchy in both 1990 and 2021. Similar to the trend observed in men, high BMI outstripped occupational asthmagens in 2021. Meanwhile, NO₂ pollution consistently occupied the fourth spot throughout this period (Figure 5C). Globally, compared with 1990, the risk of occupational asthmagens increased significantly in 2021 (Supplementary Figure 2A).

Overall, smoking played a particularly prominent role in the development of asthma in men, whereas the relative importance of high BMI and occupational asthmatogen changed over time, NO_2_ pollution and occupational asthmatogens remained stable, and the risk of high BMI has increased significantly in recent years. When looking at risk factors in regions with different SDI levels compared with 1990, it was found that the comparison results of risk factors in high SDI regions in 2021 were similar to those at the global level; in low SDI and low middle SDI regions, the risk associated with high BMI increased significantly; whereas in middle SDI and middle high SDI regions, the risk factors showed little change (Supplementary Figures 2B–F).

### 3.8 Projections showed a downward trend in both prevalence and incidence in the future

**Figure 6:**
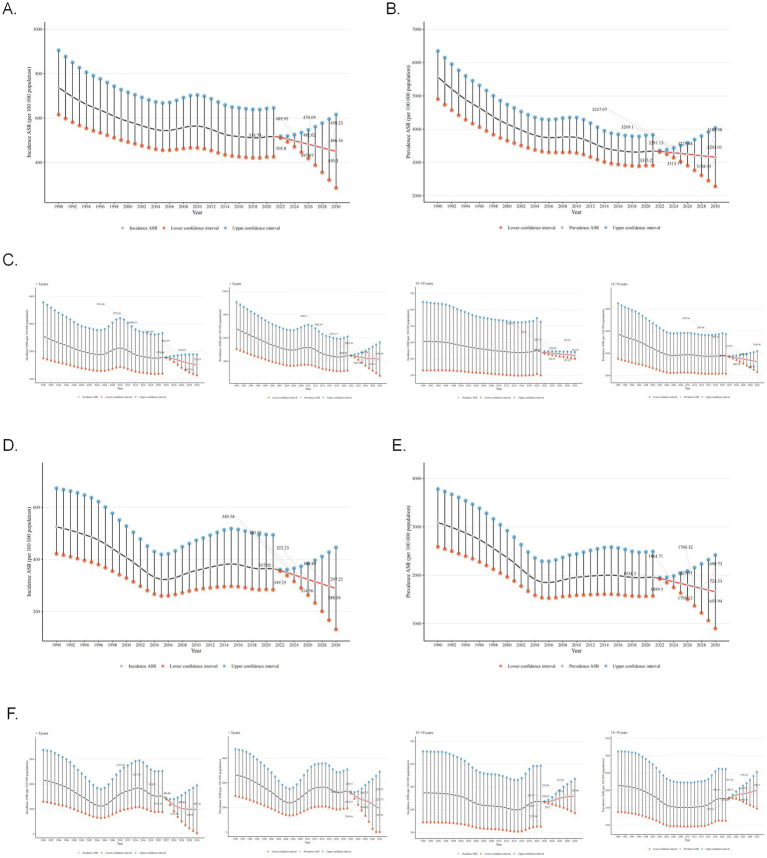
Projections of global and Chinese asthma incidence and prevalence from 2022 to 2030. **(A)** Projections of global ASIR for asthma from 2022 to 2030. **(B)** Projections of global ASPR for asthma from 2022 to 2030. **(C)** Projections of global ASIR and ASPR for asthma from 2022 to 2030 in age groups <5 years and 15–19 years. **(D)** Projections of ASIR for asthma in China from 2022 to 2030. **(E)** Projections of ASPR for asthma in China from 2022 to 2030. **(F)** Projections of ASIR and ASPR for asthma in China from 2022 to 2030 in age groups <5 years and 15–19 years. ASIR, age-standardized incidence rate; ASPR, age-standardized prevalence rate. The result area represents the 95% confidence interval (CI) for each scenario.

Globally, the future ASIR of asthma showed a steady downward trend and was projected to decrease from 513.73 per 100,000 population in 2022 to 450.3 per 100,000 in 2030 (Figure 6A). The ASPR was projected with a slight drop from 3335.2 in 2022 to 3203.01 per 100,000 population in 2030 (Figure 6B). However, the decreasing trend in incidence was more pronounced. Special attention was paid to the age groups <5 years and 15–19 years, and separate projections were made. The performance of the projections showed that while the prevalence and incidence of both age groups showed a downward trend in the future, the decreasing trend was more pronounced in the group younger than 5 years (Figure 6C).

For China, the prevalence and incidence of asthma also showed decreasing trend in the future. The ASIR of asthma showed a steady downward trend and was projected to decrease from 357.92 per 100,000 population in 2022 to 297.22 per 100,000 in 2030 (Figure 6D). The ASPR was projected with a slight drop from 1934.3 in 2022 to 1655.94 per 100,000 population in 2030 (Figure 6E). Meanwhile, the prevalence and incidence of asthma will decrease in the under-5 age group and increase in the 15–19 age group (Figure 6F). To explore the impact of future interventions or events on asthma prevalence, two scenarios were simulated: optimistic (from 2023, prevalence decreases annually due to tobacco control, emission reduction, and obesity intervention) and pessimistic (from 2025, extreme climate events cause annual increase). Simulation results showed prevalence rate changes in Supplementary Figure 3, from 2023, the green “Optimistic” line had a lower rate than the baseline; from 2025, the red “Pessimistic” line had a higher rate than the baseline. Finally, historical backtesting was performed for the period 2011–2021, with the model evaluated as excellent when the MAPE metric was below 5%. The analysis results showed (RMSE = 78.47 per 100,000, MAPE = 2.08%) that the BAPC model had extremely low prediction errors for the global asthma prevalence from 2011 to 2021, indicating that the model has robust and reliable predictive performance.

## 4 Discussion

Since 2019, researchers have gradually begun to leverage the GBD database to explore the global disease burden imposed by chronic respiratory diseases such as asthma. However, so far, no scholar has conducted a stratified analysis targeting different age groups within the entire population to clarify the incidence, prevalence, and disease burden of asthma in each age group, nor have they made predictions about the future trends in asthma incidence and prevalence.

Our study conducted a comprehensive analysis of asthma data from the GBD database spanning from 1990 to 2021, examining incidence, prevalence, YLDs, and DALYs. The findings revealed a downward trend in ASIR, ASPR, and ASYR, aligning with previous research trends (8). Although the age-standardized asthma burden rate has decreased over the past three decades, the overall asthma burden remains highly significant.

In terms of EAPC, the United States exhibits the most pronounced upward trend in ASPR and ASYR, while Japan shows the steepest decline, and China also demonstrates a notable decrease. As an economically developed country, the United States still faces a substantial asthma burden despite having abundant medical resources. Between 2001 and 2020, there were no signs of improvement in the proportion of adult asthma patients, the severity of their conditions, and the level of disease control in the United States (10). Research has found that smoking has a particularly prominent impact on asthma among African Americans and white Americans (11). Additionally, workers in the accommodation and food service, as well as food processing and related service industries, rank first in the number of emergency room visits due to asthma (12). It can thus be inferred that lifestyle habits and occupational characteristics are likely significant factors contributing to the high prevalence of asthma in the United States. The nationwide asthma prevalence surveys conducted in China (completed in the 2010s) revealed an upward trend in the number of asthma cases (13, 14). However, the declining trends observed in the ASPR and ASYR may be attributed to the deepening degree of population aging and advancements in asthma treatment. In recent years, Japan and China have successively released their respective national asthma guidelines and expert consensuses (15–20). These publications, which continuously update asthma prevention and control measures, further substantiate the gradually improving cognitive levels of asthma management in both countries.

Additionally, our study reveals a positive correlation between the SDI of a region and both the ASIR and APSR of asthma. When stratified by age, we observe that—except for the 75–94 age group, where the highest ASIR is reported in low-to-middle SDI regions—the highest incidence rates across all other age brackets predominantly cluster in areas with higher SDI levels. Regarding prevalence, only children under the age of five represent a striking exception, with their APSR peaking in low SDI regions, while other age groups generally exhibit higher prevalence rates in high SDI areas. This pattern may be attributed to the limited medical resources and heightened environmental pollution exposure in low SDI regions, which disproportionately affect young children and the elderly—groups with relatively weaker immune systems.

Furthermore, significant disparities in population size across different countries and regions substantially influence the distribution of asthma cases, incidence rates, and YLDs. For instance, populous nations such as India, China, and the United States consistently report high statistical figures in this regard. This trend stems not only from their large population bases but also from the variations in asthma prevention and control measures implemented across regions with differing SDI levels (7, 21, 22).

Age-stratified analyses have also revealed that the 15–19 age group represents a critical inflection point for asthma prevalence, incidence, and DALYs. Prior to this age range, males exhibit higher rates of asthma prevalence, incidence, and DALYs compared to females; however, beyond this stage, females begin to surpass males across these metrics. This finding underscores the importance of implementing asthma management strategies tailored to males under 15 and females over 19. Nevertheless, despite the established recognition of gender as a risk factor for asthma (23–25), significant challenges persist in developing population-level management strategies that account for gender disparities. Therefore, exploring additional key determinants—such as hormonal fluctuations, lifestyle behaviors, and environmental exposures—may offer crucial insights for enhancing asthma management approaches (26).

In our study, the highest asthma incidence occurred in children under 5, while prevalence and DALYs peaked in the elderly (>90 years), suggesting most asthma cases originate in early childhood but with cumulative harm persisting into old age. Literature identifies childhood as a critical asthma onset window, where early exposure to allergens, air pollution, and viral infections can trigger airway inflammation and exacerbations (27). However, few studies have compared asthma prevalence between adults and children in the same cross-sectional analysis. Our research robustly supports the need for enhanced asthma management in children under 5. Additionally, studies show that children diagnosed with asthma before age 5 maintain elevated airway hyperresponsiveness and inflammatory markers during long-term follow-up (28), implying irreversible airway structural changes and symptom persistence into adulthood and old age. Sears et al. tracked asthma patients for decades, finding that poorly controlled childhood-onset asthma led to a faster decline in lung function compared to healthy individuals, with increased comorbidities like COPD and ACOS (29), significantly raising disease burden and prevalence/DALYs among the elderly. These findings emphasize prioritizing early prevention and standardized treatment for childhood asthma, while also reminding clinicians and public health professionals of the need for comprehensive evaluation and management of elderly asthma patients. This approach can mitigate asthma’s long-term harm, improve quality of life, and reduce overall disease burden.

Our analysis of risk factors for asthma in China has found that smoking plays a particularly prominent role in the onset of asthma among males. Smoking has long been recognized as a risk factor for asthma (30–32), even including exposure to secondhand smoke and e-cigarettes (33–35). Tobacco exposure can increase the risk of developing asthma (31, 35, 36), and asthmatic patients who smoke face poorer asthma control (37, 38). However, ex-smokers can benefit in terms of asthma control, asthma exacerbations, lung function, and biomarkers 1 year after starting biologic therapy, similar to non-smoking asthmatic patients (39). This underscores the importance of smoking cessation campaigns.

Compared to 1990, the significance of a high BMI as a factor influencing asthma onset has increased markedly in 2021. In recent years, the global prevalence of obesity has been rising annually. As a developing country, China also faces an escalating prevalence of obesity (40). Studies have revealed that obese asthma patients experience more severe asthma exacerbations, poorer asthma control, impaired lung function, and reduced sensitivity to conventional treatments (41, 42). Due to these characteristics, the phenotype of “obese asthma” is gradually being recognized (43, 44). Our previous research on the mechanisms of obese asthma found that it differs from conventional allergic asthma, potentially exhibiting a neutrophil-predominant airway inflammation and increased M1 polarization of macrophages. These mechanisms contribute to the more severe airway inflammation and insensitivity to inhaled corticosteroid (ICS) treatment in the obese asthma phenotype. Weight loss can benefit this subset of asthma patients (45, 46). However, scientific weight loss approaches, which may prioritize the correction of poor dietary and sleep habits over blind dieting, are crucial. This study found that, compared with 1990, the significance of BMI as a factor influencing the onset of asthma has markedly increased. By 2021, it had surpassed occupational exposure to become the second-largest risk factor. This change reflects the accelerating impact of obesity on asthma, offers a targeted revelation of the association between lifestyle changes and asthma in the context of China’s urbanization process, and provides a new perspective for understanding the evolution of asthma risk factors in developing countries.

The impact of NO_2_ pollution on asthma incidence has remained stable between 1990 and 2021. NO_2_ in the atmosphere primarily originates from the combustion of fossil fuels, such as coal and oil, as well as vehicle emissions. Previous studies have found that there is a significant relationship between traffic-related nitrogen dioxide and the prevalence of asthma and its symptoms (47). With the reduction in NO_2_ exposure, asthma hospital admissions have also decreased accordingly (48). These findings underscore the hazards of environmental pollution on asthma. Moreover, this study revealed that NO_2_ has consistently served as a risk factor for asthma, exerting a stable influence throughout. As new energy vehicles become increasingly prevalent, we believe that the incidence of asthma is likely to decrease to some extent in the future, leading to notable improvements in environmental quality and public health levels.

Finally, we conducted predictive analyses on the incidence and prevalence rates of asthma globally and in China. The results indicate that both the global and Chinese asthma incidence and prevalence rates are projected to decline in the future, with the incidence rate experiencing a more pronounced reduction. This trend is closely associated with the gradually increasing public awareness of asthma. However, it is noteworthy that while the prevalence and incidence rates among children under 5 years old in China are expected to decrease, the incidence rate in the 15–19 age group may rise. This phenomenon serves as a reminder that the management of asthma patients during childhood and adolescence should remain a focal point of future efforts. However, our current model lacks the capacity to evaluate the impact of external factors such as air pollution, climate change, pandemics, and policy shifts on the predicted outcomes. Climate change may alter the distribution of seasonal allergens or exacerbate air pollution levels, thereby heightening the risk of acute asthma exacerbations. Conversely, stringent environmental regulations implemented by governments could mitigate asthma attacks. Similarly, while pandemics may trigger or worsen asthma symptoms through viral infections, measures like mask-wearing can concurrently reduce exposure to allergens.

Thus, despite our projections for asthma incidence and prevalence in 2030, it is imperative to adopt proactive environmental control strategies and enhance asthma management practices to genuinely alleviate the global burden of this disease.

### 4.1 Limitation

Firstly, while the study mentions the positive correlation between the SDI and the ASPR of asthma, it has not thoroughly explored the impact of socioeconomic factors on asthma incidence and prevalence rates. Secondly, this research primarily relies on data from the GBD study spanning 1990–2021. Although this dataset is relatively comprehensive, its estimates are model-derived and may be subject to bias in regions with limited surveillance infrastructure and resources, thereby affecting the accuracy and generalizability of the results. Additionally, this study employs the BAPC model to predict asthma trends from 2022 to 2030. However, such long-term forecasts cannot anticipate future policy interventions or unforeseen events that may alter these trends. These factors could influence asthma prevalence and incidence rates, thus limiting the comprehensiveness of the predictions. Furthermore, as this study solely focuses on risk factor analysis in China, differences in environmental conditions, cultural backgrounds, and healthcare systems across regions may affect the applicability of the findings elsewhere. Future research should aim to improve data collection and analytical methods, incorporating regional primary data to more accurately assess the global and regional burden of asthma. Simultaneously, more systematic studies should be conducted across diverse regions and encompass a broader range of risk factors (such as air quality, genetic predisposition, healthcare access, and seasonal variation), while considering local characteristics in the analysis of different regions. This will provide more valuable insights for developing more effective prevention and control strategies. Additionally, we will endeavor to construct multiple scenario-based prediction models, calculate predictive adjustments under different scenarios, incorporate uncertainty analysis, and introduce “fluctuation ranges” to enhance the reference value of our forecasts.

## 5 Conclusion

This study focuses on gender and age, revealing that the 15–19 age group is a key turning point for gender differences in asthma burden, providing a basis for tailored prevention strategies. It identifies smoking, high BMI, and NO₂ exposure as asthma risk factors. Smoking is the top risk for Chinese men, stressing the need for tobacco control. High BMI overtook occupational exposure as the second-largest risk in 2021, reflecting obesity’s growing impact and shedding light on lifestyle-asthma links during China’s urbanization, offering a new view on asthma risk evolution in developing nations.

Although asthma prevalence and incidence are expected to decline from 2022 to 2030, an increase is projected among 15–19-year-olds. This contrasts with the global downward trend, suggesting adolescence could be a new focus for asthma prevention in China, warning schools and communities to enhance targeted health education—a nuanced finding overlooked in previous global studies.

## Data Availability

The raw data supporting the conclusions of this article will be made available by the authors, without undue reservation.

## Footnotes

^1^ https://vizhub.healthdata.org/gbd-compare/

## References

[1] Miller RL Grayson MH Strothman K. Advances in asthma: new understandings of asthma’s natural history, risk factors, underlying mechanisms, and clinical management. J Allergy Clin Immunol. (2021) 148:1430–41. doi: 10.1016/j.jaci.2021.10.001, PMID: 34655640

[2] Althoff MD Ghincea A Wood LG Holguin F Sharma S. Asthma and three colinear comorbidities: obesity, OSA, and GERD. J Allergy Clin Immunol Pract. (2021) 9:3877–84. doi: 10.1016/j.jaip.2021.09.003, PMID: 34506967 PMC8578370

[3] Ye G Baldwin DS Hou R. Anxiety in asthma: a systematic review and meta-analysis. Psychol Med. (2021) 51:11–20. doi: 10.1017/S0033291720005097, PMID: 33431086

[4] Stern J Pier J Litonjua AA. Asthma epidemiology and risk factors. Semin Immunopathol. (2020) 42:5–15. doi: 10.1007/s00281-020-00785-1, PMID: 32020334

[5] Safiri S Carson-Chahhoud K Karamzad N Sullman MJM Nejadghaderi SA Taghizadieh A. Prevalence, deaths, and disability-adjusted life-years due to asthma and its attributable risk factors in 204 countries and territories, 1990–2019. Chest. (2022) 161:318–29. doi: 10.1016/j.chest.2021.09.042, PMID: 34699773

[6] Cheng F He L Deng D Zhang J Liu C. Analysis of asthma incidence and mortality rates among children aged 0–14 in 204 countries from 1990 to 2019. J Asthma. (2025) 62:45–55. doi: 10.1080/02770903.2024.2386442, PMID: 39074060

[7] Mendoza-Cano O Murillo-Zamora E. Assessing the asthma-related burden of disease in Latin American and Caribbean countries: a sociodemographic perspective. Public Health. (2024) 227:163–8. doi: 10.1016/j.puhe.2023.12.016, PMID: 38232564

[8] Yang W Li G Liu J. The incidence, prevalence, and health burden of hip fractures in China: data from the global burden of disease study 2019. Prev Med Rep. (2024) 38:102622. doi: 10.1016/j.pmedr.2024.102622, PMID: 38375171 PMC10874847

[9] Gustavsson EK Zhang D Reynolds RH Garcia-Ruiz S Ryten M. Ggtranscript: an R package for the visualization and interpretation of transcript isoforms using ggplot2. Bioinformatics. (2022) 38:3844–6. doi: 10.1093/bioinformatics/btac409, PMID: 35751589 PMC9344834

[10] Zhang F Du T Huang L Li M Li M Zhang X. Overall and subgroup prevalence of self-reported asthma in US adults: a nationally representative cross-sectional study. J Asthma. (2025) 62:36–44. doi: 10.1080/02770903.2024.2385985, PMID: 39082805

[11] Syamlal G Dodd KE Mazurek JM. Prevalence and burden of asthma among US working adults by industry and occupation-United States, 2020–2021. J Asthma. (2025) 62:73–83. doi: 10.1080/02770903.2024.2387751, PMID: 39087952 PMC11970257

[12] Nakamura Y Tamaoki J Nagase H Yamaguchi M Horiguchi T Hozawa S. Japanese guidelines for adult asthma 2020. Allergol Int. (2020) 69:519–48. doi: 10.1016/j.alit.2020.08.001, PMID: 32893125

[13] National Cooperative Group on Childhood Asthma, Institute of Environmental Health and Related Product Safety, Chinese Center for Disease Control and Prevention, Chinese Center for Disease Control and Prevention. Third nationwide survey of childhood asthma in urban areas of China. Zhonghua Er Ke Za Zhi. (2013) 51:729–35. doi: 10.3760/cma.j.issn.0578-1310.2013.10.003 24406223

[14] Ding B DiBonaventura M Karlsson N Ling X. A cross-sectional assessment of the prevalence and burden of mild asthma in urban China using the 2010, 2012, and 2013 China National Health and Wellness Surveys. J Asthma. (2017) 54:632–43. doi: 10.1080/02770903.2016.1255750, PMID: 28001469

[15] Hamasaki Y Kohno Y Ebisawa M Kondo N Nishima S Nishimuta T. Japanese guideline for childhood asthma 2014. Allergol Int. (2014) 63:335–56. doi: 10.2332/allergolint.14-RAI-0767 28942928

[16] Kakizoe T Tobisu K Takai K Tanaka Y Niizuma M. Total replacement of the bladder with an intestinal pouch for normal micturition after cystectomy. Jpn J Clin Oncol. (1989) 19:276–82. PMID: 2810824

[17] Wang C Bao Y Chen J Chen X Cheng L Guo YS. Chinese guideline on allergen immunotherapy for allergic rhinitis: the 2022 update. Allergy Asthma Immunol Res. (2022) 14:604–52. doi: 10.4168/aair.2022.14.6.604, PMID: 36426395 PMC9709690

[18] Lin J Xing B Chen P Huang M Zhou X Wu C. Chinese expert consensus-based guideline on assessment and management of asthma exacerbation. J Thorac Dis. (2019) 11:4918–35. doi: 10.21037/jtd.2019.12.67, PMID: 32030208 PMC6988075

[19] Hua W Huang H Shen H. Interpretation of 2016 *asthma management and prevention guideline*. Zhejiang Da Xue Xue Bao Yi Xue Ban. (2016) 45:447–52. doi: 10.3785/j.issn.1008-9292.2016.09.01 28087903 PMC10396884

[20] Sha L Shao M Liu C Li S Li Z Luo Y. The prevalence of asthma in children: a comparison between the year of 2010 and 2000 in urban China. Zhonghua Jie He He Hu Xi Za Zhi. (2015) 38:664–8. doi: 10.3760/cma.j.issn.1001-0939.2015.09.009 26703770

[21] Yang CH Lv JJ Li XY Yang XT Yin MY. Global burden of asthma in young adults in 204 countries and territories, 1990-2019: systematic analysis of the global burden of disease study 2019. Prev Med Rep. (2023) 37:102531. doi: 10.1016/j.pmedr.2023.102531 38162120 PMC10755496

[22] Shin YH Hwang J Kwon R Lee SW Kim MS GBD 2019 Allergic Disorders Collaborators. Global, regional, and national burden of allergic disorders and their risk factors in 204 countries and territories, from 1990 to 2019: a systematic analysis for the global burden of disease study 2019. Allergy. (2023) 78:2232–54. doi: 10.1111/all.15807, PMID: 37431853 PMC10529296

[23] Almqvist C Worm M Leynaert B working group of GA2LEN WP 2.5 Gender. Impact of gender on asthma in childhood and adolescence: a GA2LEN review. Allergy. (2008) 63:47–57. doi: 10.1111/j.1398-9995.2007.01524.x, PMID: 17822448

[24] Honkamäki J Piirilä P Hisinger-Mölkänen H Tuomisto LE Andersén H Huhtala H. Asthma remission by age at diagnosis and gender in a population-based study. J Allergy Clin Immunol Pract. (2021) 9:1950–1959.e4. doi: 10.1016/j.jaip.2020.12.015 33338683

[25] Treasure J Thompson P. Anorexia nervosa in childhood. Br J Hosp Med. (1988) 40:362–9. 3069170

[26] Zein JG Denson JL Wechsler ME. Asthma over the adult life course: gender and hormonal influences. Clin Chest Med. (2019) 40:149–61. doi: 10.1016/j.ccm.2018.10.009, PMID: 30691709

[27] Pijnenburg MW Frey U De Jongste JC Saglani S. Childhood asthma: pathogenesis and phenotypes. Eur Respir J. (2022) 59:2100731. doi: 10.1183/13993003.00731-2021, PMID: 34711541

[28] Kalliola S Malmberg LP Malmström K Pelkonen AS Mäkelä MJ. Airway hyperresponsiveness in young children with respiratory symptoms: a five-year follow-up. Ann Allergy Asthma Immunol. (2019) 122:492–7. doi: 10.1016/j.anai.2019.02.025, PMID: 30831260

[29] Sears MR Greene JM Willan AR Wiecek EM Taylor DR Flannery EM. A longitudinal, population-based, cohort study of childhood asthma followed to adulthood. N Engl J Med. (2003) 349:1414–22. doi: 10.1056/NEJMoa022363, PMID: 14534334

[30] McDonald VM Archbold G Beyene T Brew BK Franklin P Gibson PG. Asthma and landscape fire smoke: a Thoracic Society of Australia and New Zealand position statement. Respirology. (2023) 28:1023–35. doi: 10.1111/resp.14593, PMID: 37712340 PMC10946536

[31] Silvestri M Franchi S Pistorio A Petecchia L Rusconi F. Smoke exposure, wheezing, and asthma development: a systematic review and meta-analysis in unselected birth cohorts. Pediatr Pulmonol. (2015) 50:353–62. doi: 10.1002/ppul.23037, PMID: 24648197

[32] Balmes JR Holm SM. Increasing wildfire smoke from the climate crisis: impacts on asthma and allergies. J Allergy Clin Immunol. (2023) 152:1081–3. doi: 10.1016/j.jaci.2023.09.008, PMID: 37739070

[33] Lima LL Cruz CMS Fernandes AGO Pinheiro GP Souza-Machado C Lima VB. Exposure to secondhand smoke among patients with asthma: a cross-sectional study. Einstein. (2020) 18:eAO4781. doi: 10.31744/einstein_journal/2020AO4781, PMID: 31994604 PMC6986455

[34] Xian S Chen Y. E-cigarette users are associated with asthma disease: a meta-analysis. Clin Respir J. (2021) 15:457–66. doi: 10.1111/crj.13346, PMID: 33683790

[35] Ferrante G Antona R Malizia V Montalbano L Corsello G La Grutta S. Smoke exposure as a risk factor for asthma in childhood: a review of current evidence. Allergy Asthma Proc. (2014) 35:454–61. doi: 10.2500/aap.2014.35.3789, PMID: 25584912

[36] Burke H Leonardi-Bee J Hashim A Pine-Abata H Chen Y Cook DG. Prenatal and passive smoke exposure and incidence of asthma and wheeze: systematic review and meta-analysis. Pediatrics. (2012) 129:735–44. doi: 10.1542/peds.2011-2196, PMID: 22430451

[37] Thomson NC Polosa R Sin DD. Cigarette smoking and asthma. J Allergy Clin Immunol Pract. (2022) 10:2783–97. doi: 10.1016/j.jaip.2022.04.034, PMID: 35533997

[38] Alanazi AMM Alqahtani MM Alquaimi MM Alotaibi TF Algarni SS Alonizi KM. Epidemiological associations of asthma status and tobacco use, substance use, and substance misuse among adults in the United States, 2015–2019. J Asthma. (2023) 60:87–95. doi: 10.1080/02770903.2022.2029480, PMID: 35025703

[39] Stoshikj S Biener L Renner A Bal C Brugger J Krall C. Impact of smoking on biological treatment response in patients from the German Severe Asthma (GAN) Registry. J Allergy Clin Immunol Pract. (2025) 13:1125–1138.e4. doi: 10.1016/j.jaip.2025.01.005, PMID: 39800060

[40] Jaacks LM Vandevijvere S Pan A McGowan CJ Wallace C Imamura F. The obesity transition: stages of the global epidemic. Lancet Diabetes Endocrinol. (2019) 7:231–40. doi: 10.1016/S2213-8587(19)30026-9, PMID: 30704950 PMC7360432

[41] Ananth S Navarra A Vancheeswaran R. Obese, non-eosinophilic asthma: frequent exacerbators in a real-world setting. J Asthma. (2022) 59:2267–75. doi: 10.1080/02770903.2021.1996598, PMID: 34669527

[42] Dixon AE Poynter ME Garrow OJ Kaminsky DA Tharp WG Bates JHT. Peripheral airway dysfunction in obesity and obese asthma. Chest. (2023) 163:753–62. doi: 10.1016/j.chest.2022.12.030, PMID: 36610669 PMC10107055

[43] Bayless AK Wyatt TH Raynor H. Obese-asthma phenotype self-management: a literature review. J Pediatr Nurs. (2021) 60:154–63. doi: 10.1016/j.pedn.2021.04.027, PMID: 33989853

[44] Castro-Rodriguez JA. A new childhood asthma phenotype: obese with early menarche. Paediatr Respir Rev. (2016) 18:85–9. doi: 10.1016/j.prrv.2015.10.006, PMID: 26644272

[45] Bantulà M Tubita V Roca-Ferrer J Mullol J Valero A Bobolea I. Weight loss and vitamin D improve hyporesponsiveness to corticosteroids in obese asthma. J Investig Allergol Clin Immunol. (2023) 33:464–73. doi: 10.18176/jiaci.0861, PMID: 36098275

[46] Foer D Forno E Holguin F Cahill KN. Weight loss interventions for adults with obesity-related asthma. J Allergy Clin Immunol Pract. (2024) 12:840–7. doi: 10.1016/j.jaip.2023.12.041, PMID: 38159807 PMC10999349

[47] Studnicka M Hackl E Pischinger J Fangmeyer C Haschke N Kühr J. Traffic-related NO_2_ and the prevalence of asthma and respiratory symptoms in seven year olds. Eur Respir J. (1997) 10:2275–8. doi: 10.1183/09031936.97.10102275, PMID: 9387953

[48] Quintyne KI Kelly C Sheridan A Kenny P O'Dwyer M. Impact of COVID-19 lockdown restrictions: ambient NO_2_ and asthma hospital admissions. Ir Med J. (2021) 114:413. PMID: 34520648

