# Supplementary material for: Analysis of gender- and age-stratified asthma burden: forecasting prevalence trends in 2030
Source: Front Med (Lausanne). 2025 Aug 20;12:1612688. doi: 10.3389/fmed.2025.1612688 (PMC12404962; doi:10.3389/fmed.2025.1612688)
Supplement: Supplementary file 1 [file Supplementary_file_1.docx]

Supplementary Table 1: The number of global asthma incidence cases in 1990

| **location** | **val** | **upper** | **lower** | **Number_1990** |
| --- | --- | --- | --- | --- |
| Tokelau | 9.56 | 11.58 | 8.01 | 9.56 (8.01 to 11.58) |
| Niue | 12.09 | 14.78 | 10.1 | 12.09 (10.1 to 14.78) |
| Nauru | 69.41 | 84.83 | 58.11 | 69.41 (58.11 to 84.83) |
| Tuvalu | 75.48 | 90.11 | 64.61 | 75.48 (64.61 to 90.11) |
| Palau | 79.83 | 99.4 | 66.46 | 79.83 (66.46 to 99.4) |
| Cook Islands | 93.2 | 119.32 | 75.03 | 93.2 (75.03 to 119.32) |
| San Marino | 94.02 | 114.46 | 77.99 | 94.02 (77.99 to 114.46) |
| Monaco | 102.25 | 122.81 | 87.4 | 102.25 (87.4 to 122.81) |
| Northern Mariana Islands | 200.37 | 255.8 | 160.55 | 200.37 (160.55 to 255.8) |
| American Samoa | 249.15 | 314.27 | 203.29 | 249.15 (203.29 to 314.27) |
| Andorra | 270.33 | 320.39 | 226.76 | 270.33 (226.76 to 320.39) |
| Marshall Islands | 277.56 | 338.36 | 231.14 | 277.56 (231.14 to 338.36) |
| Seychelles | 327.55 | 412.12 | 265.92 | 327.55 (265.92 to 412.12) |
| Saint Kitts and Nevis | 465.22 | 595.26 | 374.92 | 465.22 (374.92 to 595.26) |
| Bermuda | 522.68 | 655.85 | 429.58 | 522.68 (429.58 to 655.85) |
| Antigua and Barbuda | 677.01 | 852.58 | 531.66 | 677.01 (531.66 to 852.58) |
| Kiribati | 682.08 | 795.98 | 588.22 | 682.08 (588.22 to 795.98) |
| Micronesia (Federated States of) | 692.48 | 833.04 | 579.21 | 692.48 (579.21 to 833.04) |
| Guam | 731.77 | 961.07 | 578.62 | 731.77 (578.62 to 961.07) |
| Tonga | 755.03 | 962.1 | 593.02 | 755.03 (593.02 to 962.1) |
| Greenland | 777.33 | 946.2 | 645.23 | 777.33 (645.23 to 946.2) |
| Dominica | 866.69 | 1095.93 | 701.62 | 866.69 (701.62 to 1095.93) |
| Samoa | 936.13 | 1144.2 | 758.15 | 936.13 (758.15 to 1144.2) |
| Vanuatu | 982.58 | 1176.46 | 827.86 | 982.58 (827.86 to 1176.46) |
| United States Virgin Islands | 1243.97 | 1585.11 | 987.97 | 1243.97 (987.97 to 1585.11) |
| Sao Tome and Principe | 1302.08 | 1631.16 | 1022.7 | 1302.08 (1022.7 to 1631.16) |
| Saint Vincent and the Grenadines | 1308.91 | 1678.24 | 1024.41 | 1308.91 (1024.41 to 1678.24) |
| Grenada | 1553 | 1975.48 | 1239.19 | 1553 (1239.19 to 1975.48) |
| Brunei Darussalam | 1710.03 | 2163.17 | 1366.11 | 1710.03 (1366.11 to 2163.17) |
| Maldives | 1920.19 | 2374.5 | 1554.56 | 1920.19 (1554.56 to 2374.5) |
| Saint Lucia | 1954.11 | 2418.72 | 1606.42 | 1954.11 (1606.42 to 2418.72) |
| Solomon Islands | 1974.31 | 2389.68 | 1653.43 | 1974.31 (1653.43 to 2389.68) |
| Iceland | 2064.65 | 2638.5 | 1694.62 | 2064.65 (1694.62 to 2638.5) |
| Luxembourg | 2101.49 | 2492.35 | 1794.07 | 2101.49 (1794.07 to 2492.35) |
| Malta | 2178.53 | 2573.99 | 1847.85 | 2178.53 (1847.85 to 2573.99) |
| Cabo Verde | 2502.74 | 3284.22 | 1941.79 | 2502.74 (1941.79 to 3284.22) |
| Barbados | 2617.43 | 3204.76 | 2143.25 | 2617.43 (2143.25 to 3204.76) |
| Bhutan | 2703.19 | 3377.55 | 2154.69 | 2703.19 (2154.69 to 3377.55) |
| Bahamas | 2799.12 | 3551.99 | 2248.5 | 2799.12 (2248.5 to 3551.99) |
| Qatar | 2832.33 | 3557.47 | 2302.57 | 2832.33 (2302.57 to 3557.47) |
| Belize | 2994.05 | 3753.32 | 2435.22 | 2994.05 (2435.22 to 3753.32) |
| Montenegro | 3513.61 | 4409.22 | 2868.8 | 3513.61 (2868.8 to 4409.22) |
| Bahrain | 3527.41 | 4299.07 | 2906.89 | 3527.41 (2906.89 to 4299.07) |
| Suriname | 4267 | 5470.46 | 3427.35 | 4267 (3427.35 to 5470.46) |
| Equatorial Guinea | 4273.25 | 5361.45 | 3479.05 | 4273.25 (3479.05 to 5361.45) |
| Cyprus | 4368.47 | 5408.78 | 3571.47 | 4368.47 (3571.47 to 5408.78) |
| Djibouti | 4678.24 | 5914.39 | 3754.51 | 4678.24 (3754.51 to 5914.39) |
| Fiji | 4872.48 | 5745.12 | 4061.53 | 4872.48 (4061.53 to 5745.12) |
| Comoros | 5974.45 | 7462.07 | 4640.26 | 5974.45 (4640.26 to 7462.07) |
| Lesotho | 5974.63 | 7341.86 | 4798.59 | 5974.63 (4798.59 to 7341.86) |
| Gambia | 7295.31 | 9418.14 | 5678.45 | 7295.31 (5678.45 to 9418.14) |
| Gabon | 7662.96 | 9720.35 | 6008.43 | 7662.96 (6008.43 to 9720.35) |
| Mauritius | 7871.68 | 9696.44 | 6598.1 | 7871.68 (6598.1 to 9696.44) |
| Estonia | 8100.02 | 9459.71 | 6948.14 | 8100.02 (6948.14 to 9459.71) |
| Timor-Leste | 8112.55 | 9953.42 | 6739.58 | 8112.55 (6739.58 to 9953.42) |
| Namibia | 9301.96 | 11822.53 | 7411 | 9301.96 (7411 to 11822.53) |
| Guyana | 9603.98 | 11979.91 | 7842.58 | 9603.98 (7842.58 to 11979.91) |
| Guinea-Bissau | 10206.68 | 12797.44 | 8111.34 | 10206.68 (8111.34 to 12797.44) |
| Trinidad and Tobago | 10702.27 | 13645.74 | 8616.38 | 10702.27 (8616.38 to 13645.74) |
| Botswana | 10762.38 | 13397.66 | 8464.26 | 10762.38 (8464.26 to 13397.66) |
| Eswatini | 11132.99 | 14155.36 | 8588.11 | 11132.99 (8588.11 to 14155.36) |
| Mongolia | 11790.88 | 15002.74 | 9482.54 | 11790.88 (9482.54 to 15002.74) |
| Kuwait | 12400.73 | 16017.94 | 9908.96 | 12400.73 (9908.96 to 16017.94) |
| Armenia | 12867.65 | 16706.87 | 10242.81 | 12867.65 (10242.81 to 16706.87) |
| Oman | 13061.37 | 16786.44 | 10335.92 | 13061.37 (10335.92 to 16786.44) |
| Latvia | 16066.71 | 19206.63 | 13571.99 | 16066.71 (13571.99 to 19206.63) |
| Slovenia | 16436.72 | 19588.03 | 13785.64 | 16436.72 (13785.64 to 19588.03) |
| Albania | 17630.48 | 22028.46 | 14605.54 | 17630.48 (14605.54 to 22028.46) |
| Palestine | 17694.19 | 22754.42 | 14160.41 | 17694.19 (14160.41 to 22754.42) |
| Lithuania | 18084.32 | 21905.91 | 15176.95 | 18084.32 (15176.95 to 21905.91) |
| Singapore | 19568.94 | 22776.47 | 16520.79 | 19568.94 (16520.79 to 22776.47) |
| North Macedonia | 19813.5 | 23336.69 | 17116.79 | 19813.5 (17116.79 to 23336.69) |
| United Arab Emirates | 20934.23 | 25495.41 | 17520.59 | 20934.23 (17520.59 to 25495.41) |
| Liberia | 21370.3 | 27730.55 | 16682.66 | 21370.3 (16682.66 to 27730.55) |
| Finland | 21382.57 | 25375.57 | 18089.03 | 21382.57 (18089.03 to 25375.57) |
| Georgia | 21864.63 | 26568.84 | 18133.81 | 21864.63 (18133.81 to 26568.84) |
| Uruguay | 21968.37 | 26105.56 | 18059.89 | 21968.37 (18059.89 to 26105.56) |
| Mauritania | 22871.36 | 29225.71 | 17721.11 | 22871.36 (17721.11 to 29225.71) |
| Congo | 22908.97 | 29000.64 | 18406.46 | 22908.97 (18406.46 to 29000.64) |
| Lebanon | 24538.36 | 30175.41 | 20137.97 | 24538.36 (20137.97 to 30175.41) |
| Israel | 25023.02 | 31027.5 | 20602.05 | 25023.02 (20602.05 to 31027.5) |
| Denmark | 25192.55 | 29776.5 | 21747.32 | 25192.55 (21747.32 to 29776.5) |
| Ireland | 25210.13 | 30093.33 | 20939.51 | 25210.13 (20939.51 to 30093.33) |
| Kyrgyzstan | 26453.1 | 33422.11 | 21441.94 | 26453.1 (21441.94 to 33422.11) |
| Slovakia | 26648.94 | 32989.72 | 21902.37 | 26648.94 (21902.37 to 32989.72) |
| Tajikistan | 27193.81 | 35542.6 | 21582.84 | 27193.81 (21582.84 to 35542.6) |
| Republic of Moldova | 27465.81 | 33170.05 | 22486.91 | 27465.81 (22486.91 to 33170.05) |
| Central African Republic | 28050.56 | 34897.77 | 23216.21 | 28050.56 (23216.21 to 34897.77) |
| Turkmenistan | 28244.14 | 35210.93 | 22803.61 | 28244.14 (22803.61 to 35210.93) |
| Jordan | 29673.33 | 37903.25 | 23639.17 | 29673.33 (23639.17 to 37903.25) |
| Panama | 30847.39 | 38879.38 | 24905.64 | 30847.39 (24905.64 to 38879.38) |
| Togo | 31243.93 | 41699.46 | 23570.74 | 31243.93 (23570.74 to 41699.46) |
| Libya | 31944.71 | 40318.93 | 25723.76 | 31944.71 (25723.76 to 40318.93) |
| New Zealand | 32707.33 | 40637.37 | 26424.16 | 32707.33 (26424.16 to 40637.37) |
| Bosnia and Herzegovina | 32897.98 | 40720.13 | 27508.43 | 32897.98 (27508.43 to 40720.13) |
| Lao People's Democratic Republic | 33058.73 | 41623.81 | 26824.77 | 33058.73 (26824.77 to 41623.81) |
| Switzerland | 33405.94 | 40217.32 | 28699.61 | 33405.94 (28699.61 to 40217.32) |
| Azerbaijan | 33874.62 | 42143.87 | 27843.52 | 33874.62 (27843.52 to 42143.87) |
| Jamaica | 34025.81 | 41949.47 | 27400.72 | 34025.81 (27400.72 to 41949.47) |
| Papua New Guinea | 35182.8 | 42289.93 | 29343.57 | 35182.8 (29343.57 to 42289.93) |
| Austria | 35907.81 | 42176.32 | 30798.21 | 35907.81 (30798.21 to 42176.32) |
| Croatia | 37134.12 | 43718.39 | 31498.65 | 37134.12 (31498.65 to 43718.39) |
| Norway | 38287.67 | 44203.83 | 33384.86 | 38287.67 (33384.86 to 44203.83) |
| Sierra Leone | 38674.04 | 48844.88 | 30696.59 | 38674.04 (30696.59 to 48844.88) |
| Costa Rica | 39948.69 | 48514.05 | 32539.12 | 39948.69 (32539.12 to 48514.05) |
| Chad | 42261.69 | 54667.39 | 33568.06 | 42261.69 (33568.06 to 54667.39) |
| Greece | 43379.3 | 52429.38 | 36401.29 | 43379.3 (36401.29 to 52429.38) |
| Benin | 46183.47 | 59409.93 | 36923.71 | 46183.47 (36923.71 to 59409.93) |
| Czechia | 49111.96 | 61059.73 | 40472.57 | 49111.96 (40472.57 to 61059.73) |
| Belgium | 49266.15 | 57893.94 | 42270.15 | 49266.15 (42270.15 to 57893.94) |
| Mali | 49406.88 | 62862.44 | 39073.51 | 49406.88 (39073.51 to 62862.44) |
| Eritrea | 50397.58 | 62857 | 41440.98 | 50397.58 (41440.98 to 62857) |
| Kazakhstan | 50564.96 | 64567.47 | 41230.57 | 50564.96 (41230.57 to 64567.47) |
| Netherlands | 51293.65 | 61014.52 | 43909.26 | 51293.65 (43909.26 to 61014.52) |
| Puerto Rico | 52423.68 | 65309.84 | 42879.7 | 52423.68 (42879.7 to 65309.84) |
| Zimbabwe | 52493.91 | 69580.62 | 40202.7 | 52493.91 (40202.7 to 69580.62) |
| Senegal | 52928.53 | 68611.46 | 42130.06 | 52928.53 (42130.06 to 68611.46) |
| Cameroon | 53465.04 | 69045 | 41047.23 | 53465.04 (41047.23 to 69045) |
| Paraguay | 54316.14 | 69794.4 | 43481.95 | 54316.14 (43481.95 to 69794.4) |
| Bulgaria | 55566.46 | 66940.4 | 46810.09 | 55566.46 (46810.09 to 66940.4) |
| Serbia | 56292.39 | 68613.61 | 47671.9 | 56292.39 (47671.9 to 68613.61) |
| Tunisia | 57083.06 | 69449.09 | 46900.53 | 57083.06 (46900.53 to 69449.09) |
| Hungary | 57551.65 | 69569.11 | 48745.11 | 57551.65 (48745.11 to 69569.11) |
| Guinea | 58126.38 | 76678.06 | 45153.05 | 58126.38 (45153.05 to 76678.06) |
| Zambia | 59656.19 | 74586.71 | 47699.21 | 59656.19 (47699.21 to 74586.71) |
| Nicaragua | 64385.43 | 82275.77 | 50662.96 | 64385.43 (50662.96 to 82275.77) |
| Nepal | 69250.38 | 86955.1 | 55919.83 | 69250.38 (55919.83 to 86955.1) |
| Cambodia | 69475.48 | 85133.37 | 57452.1 | 69475.48 (57452.1 to 85133.37) |
| Sweden | 71732.61 | 84163.62 | 61926.2 | 71732.61 (61926.2 to 84163.62) |
| Portugal | 73658.96 | 86727.03 | 62210.02 | 73658.96 (62210.02 to 86727.03) |
| Honduras | 76569.39 | 96831.11 | 61345.67 | 76569.39 (61345.67 to 96831.11) |
| Saudi Arabia | 84626.15 | 105737.27 | 68428.33 | 84626.15 (68428.33 to 105737.27) |
| Belarus | 84853.98 | 102814.74 | 70750.39 | 84853.98 (70750.39 to 102814.74) |
| Ghana | 86037.45 | 109546.9 | 68351.87 | 86037.45 (68351.87 to 109546.9) |
| Dominican Republic | 86795.03 | 105432.41 | 71964.48 | 86795.03 (71964.48 to 105432.41) |
| South Sudan | 87081.13 | 111120.38 | 70335.55 | 87081.13 (70335.55 to 111120.38) |
| Niger | 91240.1 | 115094.47 | 73402.74 | 91240.1 (73402.74 to 115094.47) |
| Burkina Faso | 91420.42 | 120741.61 | 71818.56 | 91420.42 (71818.56 to 120741.61) |
| Chile | 95010.91 | 116092.84 | 78807.22 | 95010.91 (78807.22 to 116092.84) |
| Cote d'Ivoire | 98471.8 | 128197.74 | 77469.58 | 98471.8 (77469.58 to 128197.74) |
| Burundi | 98712.73 | 125006.72 | 81143.14 | 98712.73 (81143.14 to 125006.72) |
| El Salvador | 100031.64 | 128515.13 | 78239.11 | 100031.64 (78239.11 to 128515.13) |
| Australia | 100273.48 | 121119.6 | 82165.29 | 100273.48 (82165.29 to 121119.6) |
| Syrian Arab Republic | 100957.31 | 124369.11 | 83293.16 | 100957.31 (83293.16 to 124369.11) |
| Bolivia (Plurinational State of) | 103293 | 133834.81 | 82100.97 | 103293 (82100.97 to 133834.81) |
| Malaysia | 107113.74 | 132371.04 | 90139.42 | 107113.74 (90139.42 to 132371.04) |
| Sri Lanka | 109405.15 | 130284.87 | 93924.26 | 109405.15 (93924.26 to 130284.87) |
| Angola | 110129.31 | 138610.24 | 89069.18 | 110129.31 (89069.18 to 138610.24) |
| Afghanistan | 119957.67 | 143492.75 | 102177.54 | 119957.67 (102177.54 to 143492.75) |
| Cuba | 121821.16 | 153793.08 | 98098.85 | 121821.16 (98098.85 to 153793.08) |
| Malawi | 127436.28 | 163099.61 | 101548.44 | 127436.28 (101548.44 to 163099.61) |
| Somalia | 129569.2 | 163178.84 | 105943.43 | 129569.2 (105943.43 to 163178.84) |
| Morocco | 131400.81 | 158930.28 | 109960.91 | 131400.81 (109960.91 to 158930.28) |
| Taiwan (Province of China) | 137920.91 | 170195.17 | 114488.54 | 137920.91 (114488.54 to 170195.17) |
| Guatemala | 141514.29 | 179556.81 | 113022.63 | 141514.29 (113022.63 to 179556.81) |
| Uzbekistan | 143199.72 | 178748.13 | 117581.44 | 143199.72 (117581.44 to 178748.13) |
| Spain | 146980.32 | 178215.95 | 122585.48 | 146980.32 (122585.48 to 178215.95) |
| Ecuador | 151530.13 | 202637.38 | 115316.54 | 151530.13 (115316.54 to 202637.38) |
| Yemen | 155516.08 | 190971.05 | 127943.76 | 155516.08 (127943.76 to 190971.05) |
| Iraq | 159955.46 | 196668.98 | 131073.48 | 159955.46 (131073.48 to 196668.98) |
| Democratic People's Republic of Korea | 161643.87 | 201723.68 | 133886.69 | 161643.87 (133886.69 to 201723.68) |
| Haiti | 162683.16 | 199658.34 | 136026.56 | 162683.16 (136026.56 to 199658.34) |
| Algeria | 166409.42 | 203511.87 | 134975.93 | 166409.42 (134975.93 to 203511.87) |
| Mozambique | 167972.71 | 214104.21 | 135436.55 | 167972.71 (135436.55 to 214104.21) |
| Rwanda | 169416.38 | 217409.05 | 134578.63 | 169416.38 (134578.63 to 217409.05) |
| Romania | 171253.39 | 209062.78 | 142581.09 | 171253.39 (142581.09 to 209062.78) |
| Sudan | 174832.58 | 216875.6 | 138375.01 | 174832.58 (138375.01 to 216875.6) |
| Kenya | 213058.5 | 279511.25 | 166774.49 | 213058.5 (166774.49 to 279511.25) |
| Republic of Korea | 215744.42 | 258414.1 | 182429.54 | 215744.42 (182429.54 to 258414.1) |
| Myanmar | 218725.6 | 261555.83 | 187362.58 | 218725.6 (187362.58 to 261555.83) |
| Argentina | 238014.76 | 295626.13 | 195425.64 | 238014.76 (195425.64 to 295626.13) |
| South Africa | 253309.88 | 321567.92 | 202036.9 | 253309.88 (202036.9 to 321567.92) |
| Venezuela (Bolivarian Republic of) | 255342.98 | 326448.68 | 204855.82 | 255342.98 (204855.82 to 326448.68) |
| Canada | 261271.72 | 312674.99 | 223113.58 | 261271.72 (223113.58 to 312674.99) |
| Madagascar | 261326.53 | 323506.06 | 205531.11 | 261326.53 (205531.11 to 323506.06) |
| Uganda | 287716.66 | 376670.13 | 231179.96 | 287716.66 (231179.96 to 376670.13) |
| Italy | 316323.68 | 368482.83 | 279134.25 | 316323.68 (279134.25 to 368482.83) |
| France | 328210.49 | 380848 | 282764.42 | 328210.49 (282764.42 to 380848) |
| Democratic Republic of the Congo | 330324.66 | 414169.29 | 262239.91 | 330324.66 (262239.91 to 414169.29) |
| Ukraine | 341153.47 | 427354.22 | 278927.16 | 341153.47 (278927.16 to 427354.22) |
| Colombia | 356451.7 | 453600.64 | 279938.7 | 356451.7 (279938.7 to 453600.64) |
| Thailand | 357272.28 | 435867.25 | 297087.46 | 357272.28 (297087.46 to 435867.25) |
| Peru | 359593.29 | 467703.78 | 286114.1 | 359593.29 (286114.1 to 467703.78) |
| Germany | 397017.68 | 470491.46 | 337741.77 | 397017.68 (337741.77 to 470491.46) |
| United Republic of Tanzania | 399330.68 | 515345.26 | 308969.89 | 399330.68 (308969.89 to 515345.26) |
| Viet Nam | 448618.95 | 569952.04 | 362148.46 | 448618.95 (362148.46 to 569952.04) |
| Egypt | 481650.9 | 602817.15 | 392341.67 | 481650.9 (392341.67 to 602817.15) |
| United Kingdom | 485205.33 | 574904.63 | 408106.09 | 485205.33 (408106.09 to 574904.63) |
| Iran (Islamic Republic of) | 496018.35 | 624524.83 | 396178.44 | 496018.35 (396178.44 to 624524.83) |
| Poland | 514560.63 | 594167.88 | 445629.55 | 514560.63 (445629.55 to 594167.88) |
| Pakistan | 522563.35 | 678068.47 | 408993.26 | 522563.35 (408993.26 to 678068.47) |
| Ethiopia | 552107.03 | 707844.32 | 446643.96 | 552107.03 (446643.96 to 707844.32) |
| Bangladesh | 624089.05 | 795558.78 | 500552.24 | 624089.05 (500552.24 to 795558.78) |
| Turkiye | 634781.99 | 765101.71 | 522971.68 | 634781.99 (522971.68 to 765101.71) |
| Philippines | 813917.2 | 1016857.12 | 668636.62 | 813917.2 (668636.62 to 1016857.12) |
| Mexico | 817717.63 | 1050391.38 | 642256.85 | 817717.63 (642256.85 to 1050391.38) |
| Nigeria | 1060608.43 | 1370597.82 | 843657.73 | 1060608.43 (843657.73 to 1370597.82) |
| Russian Federation | 1074311.63 | 1308329.21 | 911920.12 | 1074311.63 (911920.12 to 1308329.21) |
| Japan | 1134321.41 | 1345052.28 | 973017.85 | 1134321.41 (973017.85 to 1345052.28) |
| Indonesia | 1362402.56 | 1698637.55 | 1131867.99 | 1362402.56 (1131867.99 to 1698637.55) |
| Brazil | 2222918.28 | 2926614.44 | 1729243.39 | 2222918.28 (1729243.39 to 2926614.44) |
| United States of America | 3218353.47 | 4085740.28 | 2623241.26 | 3218353.47 (2623241.26 to 4085740.28) |
| China | 5826101.29 | 7504883.29 | 4640623.9 | 5826101.29 (4640623.9 to 7504883.29) |
| India | 6553912.15 | 8754116.4 | 4862942.47 | 6553912.15 (4862942.47 to 8754116.4) |

Supplementary Table 2: The number of global asthma incidence cases in 2021

| **location** | **val** | **upper** | **lower** | **Number_2021** |
| --- | --- | --- | --- | --- |
| Tokelau | 4.48 | 5.6 | 3.65 | 4.48 (3.65 to 5.6) |
| Niue | 4.7 | 5.65 | 3.99 | 4.7 (3.99 to 5.65) |
| Nauru | 41.6 | 49.53 | 34.78 | 41.6 (34.78 to 49.53) |
| Tuvalu | 42.67 | 50.92 | 36.1 | 42.67 (36.1 to 50.92) |
| Palau | 46.48 | 56.76 | 39.28 | 46.48 (39.28 to 56.76) |
| Cook Islands | 51.69 | 65.99 | 41.6 | 51.69 (41.6 to 65.99) |
| San Marino | 104.49 | 125.98 | 88.01 | 104.49 (88.01 to 125.98) |
| Monaco | 118.04 | 144.22 | 99.49 | 118.04 (99.49 to 144.22) |
| Northern Mariana Islands | 127.93 | 162.21 | 101.46 | 127.93 (101.46 to 162.21) |
| American Samoa | 141.54 | 177.23 | 113.91 | 141.54 (113.91 to 177.23) |
| Marshall Islands | 198.98 | 234.86 | 170.06 | 198.98 (170.06 to 234.86) |
| Andorra | 266.6 | 319.46 | 226.99 | 266.6 (226.99 to 319.46) |
| Seychelles | 327.83 | 399.84 | 275.3 | 327.83 (275.3 to 399.84) |
| Micronesia (Federated States of) | 368.37 | 435.32 | 316.77 | 368.37 (316.77 to 435.32) |
| Bermuda | 384.07 | 470.5 | 313.63 | 384.07 (313.63 to 470.5) |
| Saint Kitts and Nevis | 389.6 | 483.03 | 318.11 | 389.6 (318.11 to 483.03) |
| Guam | 439.7 | 576.25 | 348.55 | 439.7 (348.55 to 576.25) |
| Greenland | 524.1 | 641.18 | 439.69 | 524.1 (439.69 to 641.18) |
| Dominica | 534 | 656.58 | 440.35 | 534 (440.35 to 656.58) |
| United States Virgin Islands | 616.38 | 768.61 | 507.93 | 616.38 (507.93 to 768.61) |
| Tonga | 623.98 | 793.8 | 496.76 | 623.98 (496.76 to 793.8) |
| Kiribati | 660.74 | 756.11 | 577.51 | 660.74 (577.51 to 756.11) |
| Antigua and Barbuda | 661.5 | 819.89 | 536.14 | 661.5 (536.14 to 819.89) |
| Samoa | 842.46 | 1051.12 | 676.52 | 842.46 (676.52 to 1051.12) |
| Saint Vincent and the Grenadines | 876.28 | 1098.06 | 709.3 | 876.28 (709.3 to 1098.06) |
| Grenada | 1097.87 | 1362.73 | 876.77 | 1097.87 (876.77 to 1362.73) |
| Vanuatu | 1234.86 | 1451.91 | 1058.89 | 1234.86 (1058.89 to 1451.91) |
| Maldives | 1317.1 | 1653.86 | 1074.31 | 1317.1 (1074.31 to 1653.86) |
| Saint Lucia | 1321.83 | 1614.18 | 1108.24 | 1321.83 (1108.24 to 1614.18) |
| Sao Tome and Principe | 1377.3 | 1709.89 | 1118.48 | 1377.3 (1118.48 to 1709.89) |
| Malta | 1583.21 | 1935.4 | 1340.98 | 1583.21 (1340.98 to 1935.4) |
| Iceland | 1743.98 | 2194.81 | 1455.95 | 1743.98 (1455.95 to 2194.81) |
| Bhutan | 1896.18 | 2359.67 | 1577.03 | 1896.18 (1577.03 to 2359.67) |
| Cabo Verde | 2120.45 | 2730.95 | 1672.72 | 2120.45 (1672.72 to 2730.95) |
| Brunei Darussalam | 2138.92 | 2547.17 | 1826.88 | 2138.92 (1826.88 to 2547.17) |
| Luxembourg | 2446.97 | 3028.07 | 2062.07 | 2446.97 (2062.07 to 3028.07) |
| Barbados | 2534.64 | 3085.27 | 2119.16 | 2534.64 (2119.16 to 3085.27) |
| Solomon Islands | 2684.93 | 3236.31 | 2269.18 | 2684.93 (2269.18 to 3236.31) |
| Montenegro | 2781.52 | 3457.21 | 2297.31 | 2781.52 (2297.31 to 3457.21) |
| Bahamas | 2933.14 | 3649.62 | 2366.59 | 2933.14 (2366.59 to 3649.62) |
| Fiji | 3668.31 | 4241.29 | 3141.75 | 3668.31 (3141.75 to 4241.29) |
| Belize | 4220.32 | 5350.54 | 3361.81 | 4220.32 (3361.81 to 5350.54) |
| Suriname | 4660.18 | 5801.95 | 3737.23 | 4660.18 (3737.23 to 5801.95) |
| Estonia | 4688.13 | 5707.15 | 3954.17 | 4688.13 (3954.17 to 5707.15) |
| Mauritius | 4843.71 | 5635.34 | 4176.33 | 4843.71 (4176.33 to 5635.34) |
| Lesotho | 4970.25 | 5889.19 | 4133.29 | 4970.25 (4133.29 to 5889.19) |
| Cyprus | 5563.22 | 6844.78 | 4571.59 | 5563.22 (4571.59 to 6844.78) |
| Comoros | 5782.59 | 7204.19 | 4667.97 | 5782.59 (4667.97 to 7204.19) |
| Bahrain | 6272.45 | 7749.27 | 5199.61 | 6272.45 (5199.61 to 7749.27) |
| Trinidad and Tobago | 6854.41 | 8641.86 | 5567.78 | 6854.41 (5567.78 to 8641.86) |
| Latvia | 6987.08 | 8537.85 | 5855.71 | 6987.08 (5855.71 to 8537.85) |
| Guyana | 7209.83 | 8815.7 | 5907.42 | 7209.83 (5907.42 to 8815.7) |
| Armenia | 7413.68 | 9378.84 | 5875.88 | 7413.68 (5875.88 to 9378.84) |
| Equatorial Guinea | 7833.01 | 10216.91 | 6094.17 | 7833.01 (6094.17 to 10216.91) |
| Timor-Leste | 7844.26 | 9461.69 | 6555.43 | 7844.26 (6555.43 to 9461.69) |
| Djibouti | 8407.27 | 10579.25 | 6758.28 | 8407.27 (6758.28 to 10579.25) |
| Gabon | 9216.99 | 11842.11 | 7244.09 | 9216.99 (7244.09 to 11842.11) |
| Lithuania | 9226.05 | 11279.79 | 7651.98 | 9226.05 (7651.98 to 11279.79) |
| Albania | 10067.17 | 12144.19 | 8539.25 | 10067.17 (8539.25 to 12144.19) |
| Eswatini | 10656.28 | 13205.56 | 8556.43 | 10656.28 (8556.43 to 13205.56) |
| Georgia | 10741.55 | 13307.31 | 8825.6 | 10741.55 (8825.6 to 13307.31) |
| Namibia | 11139.68 | 13729.48 | 8950.94 | 11139.68 (8950.94 to 13729.48) |
| Republic of Moldova | 11247.99 | 13853.48 | 9239.02 | 11247.99 (9239.02 to 13853.48) |
| Qatar | 12001.92 | 14853.6 | 9771.75 | 12001.92 (9771.75 to 14853.6) |
| Slovenia | 12068.71 | 14492.18 | 10149.49 | 12068.71 (10149.49 to 14492.18) |
| Mongolia | 13002.98 | 16462.95 | 10408.95 | 13002.98 (10408.95 to 16462.95) |
| Gambia | 13257.33 | 16837.98 | 10504.04 | 13257.33 (10504.04 to 16837.98) |
| Botswana | 13358.46 | 16388.55 | 10865.1 | 13358.46 (10865.1 to 16388.55) |
| Guinea-Bissau | 13436.23 | 16674.3 | 11006.32 | 13436.23 (11006.32 to 16674.3) |
| North Macedonia | 14940.15 | 17449.1 | 12946.73 | 14940.15 (12946.73 to 17449.1) |
| Turkmenistan | 16901.11 | 21614.25 | 13441.23 | 16901.11 (13441.23 to 21614.25) |
| Uruguay | 17097.61 | 21124.93 | 13975.78 | 17097.61 (13975.78 to 21124.93) |
| Singapore | 17806.88 | 22259.61 | 14603.03 | 17806.88 (14603.03 to 22259.61) |
| Bosnia and Herzegovina | 19084.56 | 22987.64 | 16299.98 | 19084.56 (16299.98 to 22987.64) |
| Finland | 19377.79 | 23605.47 | 16740.76 | 19377.79 (16740.76 to 23605.47) |
| Denmark | 19494.41 | 23903.01 | 16188.15 | 19494.41 (16188.15 to 23903.01) |
| Kuwait | 19927.93 | 24213.98 | 16044.01 | 19927.93 (16044.01 to 24213.98) |
| Slovakia | 20264.78 | 24643.5 | 17117.67 | 20264.78 (17117.67 to 24643.5) |
| Croatia | 20407.99 | 24118.66 | 17384.01 | 20407.99 (17384.01 to 24118.66) |
| Ireland | 20432.69 | 25664.58 | 16524.34 | 20432.69 (16524.34 to 25664.58) |
| Jamaica | 22614.2 | 27606.59 | 18315.24 | 22614.2 (18315.24 to 27606.59) |
| Oman | 25814.75 | 32590.36 | 20658.28 | 25814.75 (20658.28 to 32590.36) |
| Norway | 26012.01 | 31996.89 | 21664.84 | 26012.01 (21664.84 to 31996.89) |
| Austria | 26067.74 | 31875.01 | 21875.4 | 26067.74 (21875.4 to 31875.01) |
| Puerto Rico | 26581.73 | 32545.62 | 21933.66 | 26581.73 (21933.66 to 32545.62) |
| Lao People's Democratic Republic | 26890.09 | 32254.04 | 22750.84 | 26890.09 (22750.84 to 32254.04) |
| Bulgaria | 27136.48 | 33466.47 | 22887.14 | 27136.48 (22887.14 to 33466.47) |
| Liberia | 27889.22 | 35844.82 | 21901.33 | 27889.22 (21901.33 to 35844.82) |
| New Zealand | 28062.99 | 35960.54 | 22253.6 | 28062.99 (22253.6 to 35960.54) |
| Greece | 28357.32 | 34632.7 | 23852.27 | 28357.32 (23852.27 to 34632.7) |
| Switzerland | 29411.14 | 36083.52 | 25005.12 | 29411.14 (25005.12 to 36083.52) |
| Libya | 30992.73 | 37809.87 | 26202.68 | 30992.73 (26202.68 to 37809.87) |
| Kyrgyzstan | 31009.24 | 39983.17 | 24800.75 | 31009.24 (24800.75 to 39983.17) |
| Panama | 32240.04 | 40510.25 | 25862.2 | 32240.04 (25862.2 to 40510.25) |
| Congo | 32528.16 | 40771.79 | 26071.47 | 32528.16 (26071.47 to 40771.79) |
| Palestine | 32563.56 | 41230.53 | 26037.41 | 32563.56 (26037.41 to 41230.53) |
| Lebanon | 33029.36 | 40275.99 | 27750.07 | 33029.36 (27750.07 to 40275.99) |
| Israel | 33917.79 | 43841.43 | 27517.68 | 33917.79 (27517.68 to 43841.43) |
| Belgium | 34175.25 | 42841.07 | 28816.38 | 34175.25 (28816.38 to 42841.07) |
| Azerbaijan | 35290.66 | 43696.29 | 29705.24 | 35290.66 (29705.24 to 43696.29) |
| Costa Rica | 35816.74 | 44679.83 | 29062.17 | 35816.74 (29062.17 to 44679.83) |
| Serbia | 36120.44 | 43709.46 | 30791.49 | 36120.44 (30791.49 to 43709.46) |
| Hungary | 36503.63 | 44321.96 | 31158.21 | 36503.63 (31158.21 to 44321.96) |
| Czechia | 38305.65 | 47589.91 | 31801.71 | 38305.65 (31801.71 to 47589.91) |
| Mauritania | 39336.49 | 51847.2 | 29826.84 | 39336.49 (29826.84 to 51847.2) |
| Tajikistan | 40338.4 | 52618.05 | 32052.06 | 40338.4 (32052.06 to 52618.05) |
| Central African Republic | 42830.39 | 50806.16 | 35888.78 | 42830.39 (35888.78 to 50806.16) |
| Netherlands | 43008.69 | 51734.32 | 36835.55 | 43008.69 (36835.55 to 51734.32) |
| Portugal | 45967.98 | 54796.36 | 40066.26 | 45967.98 (40066.26 to 54796.36) |
| Belarus | 47043.67 | 57653.28 | 38404.89 | 47043.67 (38404.89 to 57653.28) |
| Nicaragua | 47905.97 | 60016.18 | 38135.38 | 47905.97 (38135.38 to 60016.18) |
| Kazakhstan | 53601.75 | 67492.7 | 44256.22 | 53601.75 (44256.22 to 67492.7) |
| Sweden | 54740.81 | 67341.43 | 45464.11 | 54740.81 (45464.11 to 67341.43) |
| Eritrea | 55626.95 | 67753.27 | 45998.42 | 55626.95 (45998.42 to 67753.27) |
| Papua New Guinea | 55670.79 | 65752.38 | 47566.74 | 55670.79 (47566.74 to 65752.38) |
| Togo | 55727.05 | 73619.88 | 42846.75 | 55727.05 (42846.75 to 73619.88) |
| Tunisia | 56322.98 | 68891.71 | 47152.86 | 56322.98 (47152.86 to 68891.71) |
| El Salvador | 58902.82 | 74448.98 | 46038.16 | 58902.82 (46038.16 to 74448.98) |
| Sierra Leone | 61688.21 | 76520.67 | 49959.93 | 61688.21 (49959.93 to 76520.67) |
| Cambodia | 62754.61 | 76559.37 | 52324.6 | 62754.61 (52324.6 to 76559.37) |
| Zimbabwe | 65639.84 | 82895.8 | 52847.17 | 65639.84 (52847.17 to 82895.8) |
| Paraguay | 65899.98 | 84582.22 | 50716.96 | 65899.98 (50716.96 to 84582.22) |
| Honduras | 66074.29 | 82249.94 | 54053.07 | 66074.29 (54053.07 to 82249.94) |
| Jordan | 67207.16 | 84303.75 | 53863.07 | 67207.16 (53863.07 to 84303.75) |
| Dominican Republic | 70611.1 | 86333.97 | 58959.69 | 70611.1 (58959.69 to 86333.97) |
| United Arab Emirates | 70775.92 | 85693.29 | 57855.31 | 70775.92 (57855.31 to 85693.29) |
| Senegal | 73829.84 | 94660.69 | 57851.14 | 73829.84 (57851.14 to 94660.69) |
| Bolivia (Plurinational State of) | 78202.22 | 98858.83 | 61368 | 78202.22 (61368 to 98858.83) |
| Nepal | 83928.89 | 103854.65 | 69967.74 | 83928.89 (69967.74 to 103854.65) |
| Syrian Arab Republic | 84564.58 | 100602.55 | 71355.41 | 84564.58 (71355.41 to 100602.55) |
| Cuba | 85783 | 104709.69 | 71407.04 | 85783 (71407.04 to 104709.69) |
| Guatemala | 86176.92 | 107533.33 | 68426.5 | 86176.92 (68426.5 to 107533.33) |
| Benin | 92404.91 | 116971.42 | 75182.66 | 92404.91 (75182.66 to 116971.42) |
| Romania | 96306.81 | 118703.64 | 81297.58 | 96306.81 (81297.58 to 118703.64) |
| Zambia | 97007.91 | 121665.02 | 77210.39 | 97007.91 (77210.39 to 121665.02) |
| South Sudan | 100982.37 | 125625.16 | 83481.84 | 100982.37 (83481.84 to 125625.16) |
| Guinea | 101538.73 | 126277.82 | 81085.95 | 101538.73 (81085.95 to 126277.82) |
| Australia | 104411.04 | 133262.4 | 84585.8 | 104411.04 (84585.8 to 133262.4) |
| Chile | 112124.67 | 135190.86 | 93996.23 | 112124.67 (93996.23 to 135190.86) |
| Chad | 113790.06 | 143173.79 | 90793.44 | 113790.06 (90793.44 to 143173.79) |
| Ecuador | 115647.51 | 152157.1 | 87256.09 | 115647.51 (87256.09 to 152157.1) |
| Sri Lanka | 116791.58 | 135560.08 | 102990.88 | 116791.58 (102990.88 to 135560.08) |
| Malaysia | 119514.14 | 148634.1 | 97657.02 | 119514.14 (97657.02 to 148634.1) |
| Mali | 120522.1 | 160259.21 | 92869.77 | 120522.1 (92869.77 to 160259.21) |
| Taiwan (Province of China) | 122263.77 | 148097.94 | 104307.29 | 122263.77 (104307.29 to 148097.94) |
| Cameroon | 124452.57 | 158736.73 | 96676.48 | 124452.57 (96676.48 to 158736.73) |
| Democratic People's Republic of Korea | 128961.59 | 155121.83 | 111674.4 | 128961.59 (111674.4 to 155121.83) |
| Spain | 130285.48 | 159680.11 | 109438.48 | 130285.48 (109438.48 to 159680.11) |
| Ukraine | 139689.54 | 176907.06 | 111461.84 | 139689.54 (111461.84 to 176907.06) |
| Burundi | 140374.19 | 171439.86 | 114870.55 | 140374.19 (114870.55 to 171439.86) |
| Saudi Arabia | 144043.12 | 181332.75 | 117600.18 | 144043.12 (117600.18 to 181332.75) |
| Ghana | 144545.79 | 183717.52 | 117401.29 | 144545.79 (117401.29 to 183717.52) |
| Morocco | 145933.61 | 175956.12 | 122388.83 | 145933.61 (122388.83 to 175956.12) |
| Republic of Korea | 163854.24 | 195658.22 | 140986.78 | 163854.24 (140986.78 to 195658.22) |
| Malawi | 165476.99 | 210987.54 | 130833.67 | 165476.99 (130833.67 to 210987.54) |
| South Africa | 166063.87 | 212024.67 | 133274.16 | 166063.87 (133274.16 to 212024.67) |
| Italy | 166866.2 | 203191.71 | 140076.13 | 166866.2 (140076.13 to 203191.71) |
| Venezuela (Bolivarian Republic of) | 170009.39 | 212082.23 | 134940.73 | 170009.39 (134940.73 to 212082.23) |
| Myanmar | 178564.99 | 210341.77 | 153083.66 | 178564.99 (153083.66 to 210341.77) |
| Uzbekistan | 184078.83 | 226354.07 | 151251.68 | 184078.83 (151251.68 to 226354.07) |
| Cote d'Ivoire | 185468.44 | 235297.95 | 146700.13 | 185468.44 (146700.13 to 235297.95) |
| Burkina Faso | 193516.13 | 246383.47 | 153939.97 | 193516.13 (153939.97 to 246383.47) |
| Rwanda | 194607.24 | 243140.1 | 156534.45 | 194607.24 (156534.45 to 243140.1) |
| Angola | 205902.9 | 261858.04 | 165788.4 | 205902.9 (165788.4 to 261858.04) |
| Sudan | 211544.21 | 259416.25 | 172313.83 | 211544.21 (172313.83 to 259416.25) |
| Yemen | 216304.51 | 265193.86 | 180076.05 | 216304.51 (180076.05 to 265193.86) |
| Niger | 216702.53 | 273763.03 | 171981.37 | 216702.53 (171981.37 to 273763.03) |
| Algeria | 220138.29 | 273203.3 | 180258.3 | 220138.29 (180258.3 to 273203.3) |
| Iraq | 223349.71 | 283253.61 | 180975.01 | 223349.71 (180975.01 to 283253.61) |
| Germany | 227402.26 | 279401.65 | 194281.38 | 227402.26 (194281.38 to 279401.65) |
| Afghanistan | 228995.23 | 275682.89 | 189170.78 | 228995.23 (189170.78 to 275682.89) |
| Haiti | 239557.38 | 289256.98 | 200531.98 | 239557.38 (200531.98 to 289256.98) |
| France | 247134.24 | 303797.71 | 208756.9 | 247134.24 (208756.9 to 303797.71) |
| Argentina | 255880.79 | 325044.1 | 209196.99 | 255880.79 (209196.99 to 325044.1) |
| Thailand | 256413.86 | 306578.11 | 221710.26 | 256413.86 (221710.26 to 306578.11) |
| Colombia | 257443.17 | 325786.23 | 207431.96 | 257443.17 (207431.96 to 325786.23) |
| Somalia | 259275.65 | 315272.09 | 214904.73 | 259275.65 (214904.73 to 315272.09) |
| Kenya | 259279.28 | 329764.52 | 206178.34 | 259279.28 (206178.34 to 329764.52) |
| Mozambique | 261437.05 | 329107.17 | 210049.15 | 261437.05 (210049.15 to 329107.17) |
| Canada | 263764.95 | 329197.49 | 216213.1 | 263764.95 (216213.1 to 329197.49) |
| Peru | 293007.45 | 383500.21 | 222459.59 | 293007.45 (222459.59 to 383500.21) |
| Madagascar | 355967.86 | 438866.37 | 289367.22 | 355967.86 (289367.22 to 438866.37) |
| Iran (Islamic Republic of) | 365935.82 | 454695.83 | 300157.9 | 365935.82 (300157.9 to 454695.83) |
| United Kingdom | 398685.17 | 483365.85 | 334888.7 | 398685.17 (334888.7 to 483365.85) |
| Poland | 403697.36 | 493324.92 | 337639 | 403697.36 (337639 to 493324.92) |
| Viet Nam | 451332.56 | 551421.07 | 379760.96 | 451332.56 (379760.96 to 551421.07) |
| Russian Federation | 462778.87 | 583699.79 | 370941.46 | 462778.87 (370941.46 to 583699.79) |
| Japan | 474015.87 | 578243.09 | 401170.73 | 474015.87 (401170.73 to 578243.09) |
| Bangladesh | 485377.56 | 638807.96 | 387679.72 | 485377.56 (387679.72 to 638807.96) |
| Uganda | 504807.85 | 636491.59 | 397091.05 | 504807.85 (397091.05 to 636491.59) |
| Democratic Republic of the Congo | 516252.18 | 637226.72 | 416395.72 | 516252.18 (416395.72 to 637226.72) |
| Turkiye | 518748.83 | 630094.14 | 441775.89 | 518748.83 (441775.89 to 630094.14) |
| Mexico | 532367.47 | 700480.56 | 411444.2 | 532367.47 (411444.2 to 700480.56) |
| Egypt | 566339.21 | 695623.43 | 461170.42 | 566339.21 (461170.42 to 695623.43) |
| Ethiopia | 595237.26 | 769002.11 | 476451.39 | 595237.26 (476451.39 to 769002.11) |
| Pakistan | 689117.26 | 893893.88 | 544014.38 | 689117.26 (544014.38 to 893893.88) |
| United Republic of Tanzania | 755834.42 | 984397.41 | 580927.62 | 755834.42 (580927.62 to 984397.41) |
| Philippines | 840031.44 | 1010211.88 | 711624.82 | 840031.44 (711624.82 to 1010211.88) |
| Indonesia | 1009896.22 | 1198639.65 | 866092.29 | 1009896.22 (866092.29 to 1198639.65) |
| Brazil | 1595216.27 | 2089455.53 | 1238527.34 | 1595216.27 (1238527.34 to 2089455.53) |
| Nigeria | 2167163.54 | 2797155.25 | 1712783.59 | 2167163.54 (1712783.59 to 2797155.25) |
| United States of America | 3725882.23 | 4508860.57 | 3137859.36 | 3725882.23 (3137859.36 to 4508860.57) |
| China | 3934874.82 | 5136133.06 | 3183310.91 | 3934874.82 (3183310.91 to 5136133.06) |
| India | 4900531.8 | 6052521.19 | 4056256.53 | 4900531.8 (4056256.53 to 6052521.19) |

Supplementary Table 3: The number of global asthma prevalence cases in 1990

| **location** | **val** | **upper** | **lower** | **Number_1990** |
| --- | --- | --- | --- | --- |
| Tokelau | 56.47 | 63.83 | 50.21 | 56.47 (50.21 to 63.83) |
| Niue | 69.25 | 78.99 | 60.67 | 69.25 (60.67 to 78.99) |
| Nauru | 376.11 | 425.41 | 335.83 | 376.11 (335.83 to 425.41) |
| Tuvalu | 427.67 | 469.18 | 387.79 | 427.67 (387.79 to 469.18) |
| Palau | 469.08 | 546.69 | 406.89 | 469.08 (406.89 to 546.69) |
| Cook Islands | 499.23 | 599.66 | 421.95 | 499.23 (421.95 to 599.66) |
| Northern Mariana Islands | 1072.37 | 1273.74 | 898.57 | 1072.37 (898.57 to 1273.74) |
| American Samoa | 1230.09 | 1467.52 | 1057.3 | 1230.09 (1057.3 to 1467.52) |
| Marshall Islands | 1466.01 | 1664.12 | 1285.8 | 1466.01 (1285.8 to 1664.12) |
| San Marino | 1659.29 | 1972.12 | 1364.41 | 1659.29 (1364.41 to 1972.12) |
| Seychelles | 1865.06 | 2168.04 | 1589.36 | 1865.06 (1589.36 to 2168.04) |
| Monaco | 2195.3 | 2556.18 | 1866.65 | 2195.3 (1866.65 to 2556.18) |
| Saint Kitts and Nevis | 2684.63 | 3203.37 | 2241.77 | 2684.63 (2241.77 to 3203.37) |
| Bermuda | 3496.31 | 4142.12 | 2951.45 | 3496.31 (2951.45 to 4142.12) |
| Guam | 3841.91 | 4780.71 | 3096.45 | 3841.91 (3096.45 to 4780.71) |
| Micronesia (Federated States of) | 3900.12 | 4325.97 | 3492.82 | 3900.12 (3492.82 to 4325.97) |
| Antigua and Barbuda | 3980.61 | 4955.42 | 3239.46 | 3980.61 (3239.46 to 4955.42) |
| Kiribati | 4121.26 | 4490.44 | 3778.3 | 4121.26 (3778.3 to 4490.44) |
| Tonga | 4221.49 | 5116.13 | 3501.01 | 4221.49 (3501.01 to 5116.13) |
| Samoa | 4897.97 | 5718.89 | 4067.22 | 4897.97 (4067.22 to 5718.89) |
| Dominica | 5193.1 | 6134.46 | 4354.91 | 5193.1 (4354.91 to 6134.46) |
| Vanuatu | 5259.77 | 5871.58 | 4708.79 | 5259.77 (4708.79 to 5871.58) |
| Andorra | 5478.96 | 6427.4 | 4600.95 | 5478.96 (4600.95 to 6427.4) |
| Greenland | 5961.22 | 6737.1 | 5250.82 | 5961.22 (5250.82 to 6737.1) |
| Sao Tome and Principe | 6963.03 | 8165.37 | 5894.15 | 6963.03 (5894.15 to 8165.37) |
| Saint Vincent and the Grenadines | 7481.07 | 9019.74 | 6200.27 | 7481.07 (6200.27 to 9019.74) |
| United States Virgin Islands | 7698.37 | 9329.08 | 6354.44 | 7698.37 (6354.44 to 9329.08) |
| Maldives | 9601.95 | 11106.98 | 8258.91 | 9601.95 (8258.91 to 11106.98) |
| Grenada | 9889.55 | 11978.69 | 7930.6 | 9889.55 (7930.6 to 11978.69) |
| Solomon Islands | 10464.06 | 11866.19 | 9302.91 | 10464.06 (9302.91 to 11866.19) |
| Saint Lucia | 12263.24 | 14195.96 | 10396.19 | 12263.24 (10396.19 to 14195.96) |
| Brunei Darussalam | 12619.7 | 15269.67 | 10509.27 | 12619.7 (10509.27 to 15269.67) |
| Cabo Verde | 13150.89 | 15876.32 | 11025.98 | 13150.89 (11025.98 to 15876.32) |
| Bhutan | 15469.26 | 18524.73 | 13082.41 | 15469.26 (13082.41 to 18524.73) |
| Belize | 15958.1 | 18830.26 | 13301.41 | 15958.1 (13301.41 to 18830.26) |
| Qatar | 16661.82 | 19917.91 | 13842.23 | 16661.82 (13842.23 to 19917.91) |
| Bahamas | 16851.91 | 20444.28 | 13888.3 | 16851.91 (13888.3 to 20444.28) |
| Barbados | 19698.61 | 23015.92 | 16763.38 | 19698.61 (16763.38 to 23015.92) |
| Bahrain | 21604.18 | 24979.92 | 18659.78 | 21604.18 (18659.78 to 24979.92) |
| Equatorial Guinea | 21613.32 | 25012.54 | 18746.6 | 21613.32 (18746.6 to 25012.54) |
| Montenegro | 23318.76 | 28576.79 | 18927.48 | 23318.76 (18927.48 to 28576.79) |
| Suriname | 24455.82 | 28962.68 | 20552.99 | 24455.82 (20552.99 to 28962.68) |
| Djibouti | 24459.82 | 28596.95 | 20643.78 | 24459.82 (20643.78 to 28596.95) |
| Lesotho | 26686.81 | 31240.41 | 22806.08 | 26686.81 (22806.08 to 31240.41) |
| Fiji | 29106.5 | 32617.29 | 25483.18 | 29106.5 (25483.18 to 32617.29) |
| Iceland | 29850.35 | 34664.33 | 25352.43 | 29850.35 (25352.43 to 34664.33) |
| Comoros | 31704.71 | 37859.09 | 25850.45 | 31704.71 (25850.45 to 37859.09) |
| Gambia | 35031.35 | 41459.13 | 29588.99 | 35031.35 (29588.99 to 41459.13) |
| Malta | 38612.08 | 44791.15 | 32687.22 | 38612.08 (32687.22 to 44791.15) |
| Gabon | 40484.54 | 48136.91 | 33296.72 | 40484.54 (33296.72 to 48136.91) |
| Timor-Leste | 41333.91 | 47669.65 | 35620.8 | 41333.91 (35620.8 to 47669.65) |
| Namibia | 46896.65 | 55954.37 | 39829.82 | 46896.65 (39829.82 to 55954.37) |
| Luxembourg | 47354.45 | 55043 | 39440.6 | 47354.45 (39440.6 to 55043) |
| Mauritius | 51639.86 | 58509.04 | 44782.03 | 51639.86 (44782.03 to 58509.04) |
| Guyana | 52632.54 | 61664.54 | 44595.57 | 52632.54 (44595.57 to 61664.54) |
| Guinea-Bissau | 53530.28 | 60957.56 | 46573.43 | 53530.28 (46573.43 to 60957.56) |
| Botswana | 57558.81 | 68462.39 | 48706.23 | 57558.81 (48706.23 to 68462.39) |
| Trinidad and Tobago | 58418.87 | 70138.86 | 49138.02 | 58418.87 (49138.02 to 70138.86) |
| Mongolia | 61528.3 | 73529.09 | 52413.92 | 61528.3 (52413.92 to 73529.09) |
| Eswatini | 63616.46 | 74188.4 | 54482.06 | 63616.46 (54482.06 to 74188.4) |
| Estonia | 65363.62 | 73715.08 | 58053.19 | 65363.62 (58053.19 to 73715.08) |
| Oman | 68878.54 | 86279.7 | 55696.07 | 68878.54 (55696.07 to 86279.7) |
| Armenia | 71187.14 | 87705.52 | 58639.77 | 71187.14 (58639.77 to 87705.52) |
| Cyprus | 73840.73 | 91031.92 | 58598.19 | 73840.73 (58598.19 to 91031.92) |
| Kuwait | 75129.87 | 89643.58 | 62038.56 | 75129.87 (62038.56 to 89643.58) |
| Palestine | 93397.16 | 113263.01 | 76825.33 | 93397.16 (76825.33 to 113263.01) |
| Liberia | 98102.03 | 117786 | 81063.47 | 98102.03 (81063.47 to 117786) |
| Albania | 98847.23 | 117714.34 | 82778.43 | 98847.23 (82778.43 to 117714.34) |
| Congo | 126674.26 | 149021.56 | 106107.99 | 126674.26 (106107.99 to 149021.56) |
| Mauritania | 137093.49 | 162289.09 | 114038.12 | 137093.49 (114038.12 to 162289.09) |
| Lithuania | 138480.32 | 162485.3 | 120051.65 | 138480.32 (120051.65 to 162485.3) |
| Tajikistan | 141176.16 | 171845.29 | 116814.1 | 141176.16 (116814.1 to 171845.29) |
| Latvia | 142850.85 | 163539.91 | 125606.31 | 142850.85 (125606.31 to 163539.91) |
| Georgia | 145699.49 | 169110.09 | 126070.98 | 145699.49 (126070.98 to 169110.09) |
| Central African Republic | 147219.63 | 168883.86 | 128207.55 | 147219.63 (128207.55 to 168883.86) |
| Slovenia | 155753.37 | 179386.9 | 133764.7 | 155753.37 (133764.7 to 179386.9) |
| Togo | 156418.55 | 192705.84 | 124009.99 | 156418.55 (124009.99 to 192705.84) |
| Lebanon | 159907.1 | 185464.48 | 135316.79 | 159907.1 (135316.79 to 185464.48) |
| Jordan | 161471.39 | 196859.97 | 130149.9 | 161471.39 (130149.9 to 196859.97) |
| North Macedonia | 163437.82 | 185808.5 | 143998.21 | 163437.82 (143998.21 to 185808.5) |
| Turkmenistan | 163794.95 | 191775.51 | 138432.45 | 163794.95 (138432.45 to 191775.51) |
| Lao People's Democratic Republic | 166027.15 | 191393.78 | 141210.57 | 166027.15 (141210.57 to 191393.78) |
| Kyrgyzstan | 169255.01 | 199689.16 | 142111.05 | 169255.01 (142111.05 to 199689.16) |
| United Arab Emirates | 171164.5 | 194133.66 | 150476.29 | 171164.5 (150476.29 to 194133.66) |
| Libya | 180148.45 | 214069.66 | 151064.79 | 180148.45 (151064.79 to 214069.66) |
| Slovakia | 181526.65 | 214096.33 | 153115.92 | 181526.65 (153115.92 to 214096.33) |
| Sierra Leone | 187438.82 | 220079.12 | 159282.35 | 187438.82 (159282.35 to 220079.12) |
| Panama | 187675.47 | 222275.73 | 153371.82 | 187675.47 (153371.82 to 222275.73) |
| Chad | 189709.24 | 227210.31 | 158845.38 | 189709.24 (158845.38 to 227210.31) |
| Papua New Guinea | 189896.36 | 214913.2 | 169846.45 | 189896.36 (169846.45 to 214913.2) |
| Jamaica | 202471.93 | 237062.07 | 171350.51 | 202471.93 (171350.51 to 237062.07) |
| Azerbaijan | 203439.45 | 238509.03 | 174296.15 | 203439.45 (174296.15 to 238509.03) |
| Republic of Moldova | 203561.17 | 234775.69 | 176106 | 203561.17 (176106 to 234775.69) |
| Singapore | 219272.23 | 248076.33 | 189981.68 | 219272.23 (189981.68 to 248076.33) |
| Benin | 221801.95 | 258317.71 | 189584.38 | 221801.95 (189584.38 to 258317.71) |
| Mali | 230397.78 | 274889.29 | 192267.98 | 230397.78 (192267.98 to 274889.29) |
| Uruguay | 235204.66 | 262883.59 | 208849.9 | 235204.66 (208849.9 to 262883.59) |
| Cameroon | 240792.05 | 291239.44 | 194782.37 | 240792.05 (194782.37 to 291239.44) |
| Zimbabwe | 241920.12 | 302809.72 | 195892.98 | 241920.12 (195892.98 to 302809.72) |
| Bosnia and Herzegovina | 242686.96 | 287788.29 | 204454.93 | 242686.96 (204454.93 to 287788.29) |
| Senegal | 248032.1 | 297992.17 | 204003.82 | 248032.1 (204003.82 to 297992.17) |
| Costa Rica | 256306.3 | 299513.43 | 215690.81 | 256306.3 (215690.81 to 299513.43) |
| Zambia | 261878.17 | 314596.81 | 211112.5 | 261878.17 (211112.5 to 314596.81) |
| Eritrea | 271767.55 | 312426.16 | 231791.68 | 271767.55 (231791.68 to 312426.16) |
| Paraguay | 276380.7 | 332447.52 | 227674.94 | 276380.7 (227674.94 to 332447.52) |
| Kazakhstan | 278256.04 | 330855.11 | 235042.98 | 278256.04 (235042.98 to 330855.11) |
| Guinea | 289461.22 | 344232.75 | 236726.27 | 289461.22 (236726.27 to 344232.75) |
| Israel | 309318.9 | 357135.42 | 265838.63 | 309318.9 (265838.63 to 357135.42) |
| Croatia | 320463.4 | 365969.4 | 276505.26 | 320463.4 (276505.26 to 365969.4) |
| Tunisia | 331271.79 | 385711.52 | 284585.21 | 331271.79 (284585.21 to 385711.52) |
| Cambodia | 344116.19 | 397083.22 | 298048.09 | 344116.19 (298048.09 to 397083.22) |
| Nicaragua | 354376.86 | 425257.75 | 285957.85 | 354376.86 (285957.85 to 425257.75) |
| Czechia | 361095.1 | 424078.02 | 308090.26 | 361095.1 (308090.26 to 424078.02) |
| Nepal | 373782.13 | 439758.72 | 319967.68 | 373782.13 (319967.68 to 439758.72) |
| Ghana | 403265.74 | 481164.58 | 342250.3 | 403265.74 (342250.3 to 481164.58) |
| Denmark | 403320.14 | 455130.29 | 355960.27 | 403320.14 (355960.27 to 455130.29) |
| Serbia | 406100.4 | 466977.08 | 351920.18 | 406100.4 (351920.18 to 466977.08) |
| Finland | 410271.76 | 473060.15 | 348620.33 | 410271.76 (348620.33 to 473060.15) |
| Honduras | 411354.1 | 481688.24 | 343487.79 | 411354.1 (343487.79 to 481688.24) |
| Puerto Rico | 426682.28 | 497607.26 | 366152.8 | 426682.28 (366152.8 to 497607.26) |
| Saudi Arabia | 432611.9 | 518451.6 | 365169.9 | 432611.9 (365169.9 to 518451.6) |
| Niger | 433563.71 | 511299.62 | 367111.75 | 433563.71 (367111.75 to 511299.62) |
| Dominican Republic | 441086.98 | 512860.58 | 370670.5 | 441086.98 (370670.5 to 512860.58) |
| Burkina Faso | 446570.25 | 543650.08 | 366027.73 | 446570.25 (366027.73 to 543650.08) |
| South Sudan | 454617.99 | 538692.51 | 383802.95 | 454617.99 (383802.95 to 538692.51) |
| Ireland | 457637.8 | 524162.26 | 395787.26 | 457637.8 (395787.26 to 524162.26) |
| Hungary | 480162.43 | 547089.26 | 420025.53 | 480162.43 (420025.53 to 547089.26) |
| Bulgaria | 482714.95 | 559866.9 | 417418.82 | 482714.95 (417418.82 to 559866.9) |
| Cote d'Ivoire | 498203.54 | 588872.38 | 417053.07 | 498203.54 (417053.07 to 588872.38) |
| Bolivia (Plurinational State of) | 539461.83 | 645834.39 | 436555.33 | 539461.83 (436555.33 to 645834.39) |
| Syrian Arab Republic | 541721.57 | 625464.55 | 464018.19 | 541721.57 (464018.19 to 625464.55) |
| Angola | 549232.64 | 638430.14 | 468644.99 | 549232.64 (468644.99 to 638430.14) |
| Burundi | 552714.9 | 623787.45 | 479498.62 | 552714.9 (479498.62 to 623787.45) |
| Malawi | 624143.75 | 741788.65 | 507453.49 | 624143.75 (507453.49 to 741788.65) |
| New Zealand | 628959.16 | 728569.55 | 537572.7 | 628959.16 (537572.7 to 728569.55) |
| Norway | 635789.86 | 716930.87 | 552098.19 | 635789.86 (552098.19 to 716930.87) |
| Switzerland | 639822.14 | 741639.97 | 545627.95 | 639822.14 (545627.95 to 741639.97) |
| El Salvador | 650246.19 | 799107.41 | 511065.28 | 650246.19 (511065.28 to 799107.41) |
| Malaysia | 667942.59 | 755140.47 | 588038.12 | 667942.59 (588038.12 to 755140.47) |
| Austria | 693036.44 | 797110.57 | 581702.6 | 693036.44 (581702.6 to 797110.57) |
| Somalia | 694097.1 | 798445.29 | 589705.92 | 694097.1 (589705.92 to 798445.29) |
| Morocco | 697855.63 | 806310.53 | 602949.26 | 697855.63 (602949.26 to 806310.53) |
| Sri Lanka | 713333.34 | 794901.22 | 642559.97 | 713333.34 (642559.97 to 794901.22) |
| Guatemala | 742454.62 | 880554.53 | 613503.88 | 742454.62 (613503.88 to 880554.53) |
| Greece | 767296.79 | 911307.68 | 633411.69 | 767296.79 (633411.69 to 911307.68) |
| Afghanistan | 790739.85 | 867635.04 | 713570.24 | 790739.85 (713570.24 to 867635.04) |
| Ecuador | 826839.01 | 1007487.67 | 651440.25 | 826839.01 (651440.25 to 1007487.67) |
| Mozambique | 829155.56 | 982067.58 | 696145.3 | 829155.56 (696145.3 to 982067.58) |
| Belarus | 830714.39 | 969769.25 | 711343.39 | 830714.39 (711343.39 to 969769.25) |
| Chile | 861709.55 | 991529.8 | 750038.19 | 861709.55 (750038.19 to 991529.8) |
| Yemen | 870318.27 | 988746.58 | 755705.47 | 870318.27 (755705.47 to 988746.58) |
| Cuba | 876818.3 | 1047571.74 | 730898.01 | 876818.3 (730898.01 to 1047571.74) |
| Iraq | 891861.89 | 1037063.71 | 765702.67 | 891861.89 (765702.67 to 1037063.71) |
| Algeria | 914387.64 | 1076215.32 | 767930.14 | 914387.64 (767930.14 to 1076215.32) |
| Sudan | 923882.28 | 1088156.74 | 758828.65 | 923882.28 (758828.65 to 1088156.74) |
| Uzbekistan | 936014.57 | 1078676.22 | 797559.29 | 936014.57 (797559.29 to 1078676.22) |
| Belgium | 969291.47 | 1112832.31 | 808715.83 | 969291.47 (808715.83 to 1112832.31) |
| Kenya | 979839.05 | 1211444.8 | 796833.33 | 979839.05 (796833.33 to 1211444.8) |
| Democratic People's Republic of Korea | 1073413.95 | 1244928.44 | 923746.44 | 1073413.95 (923746.44 to 1244928.44) |
| Taiwan (Province of China) | 1087840.17 | 1282237.67 | 912369.36 | 1087840.17 (912369.36 to 1282237.67) |
| Haiti | 1128092.08 | 1265564.5 | 997735.25 | 1128092.08 (997735.25 to 1265564.5) |
| Myanmar | 1183228.27 | 1337771.1 | 1047590.41 | 1183228.27 (1047590.41 to 1337771.1) |
| Rwanda | 1206188.16 | 1405202.42 | 1032936.23 | 1206188.16 (1032936.23 to 1405202.42) |
| South Africa | 1312308.09 | 1568770.39 | 1101705.99 | 1312308.09 (1101705.99 to 1568770.39) |
| Sweden | 1346735.61 | 1536626.24 | 1152578.32 | 1346735.61 (1152578.32 to 1536626.24) |
| Venezuela (Bolivarian Republic of) | 1438132.88 | 1711804 | 1169370.09 | 1438132.88 (1169370.09 to 1711804) |
| Romania | 1445888.88 | 1682121.17 | 1218733.19 | 1445888.88 (1218733.19 to 1682121.17) |
| Uganda | 1470720.04 | 1737744.95 | 1203164.7 | 1470720.04 (1203164.7 to 1737744.95) |
| Democratic Republic of the Congo | 1566032.62 | 1874787.31 | 1293367.9 | 1566032.62 (1293367.9 to 1874787.31) |
| Netherlands | 1577979.93 | 1858961.65 | 1254794.66 | 1577979.93 (1254794.66 to 1858961.65) |
| Portugal | 1595615.55 | 1924652.03 | 1275553.88 | 1595615.55 (1275553.88 to 1924652.03) |
| Madagascar | 1743775.5 | 1963557.07 | 1531157.21 | 1743775.5 (1531157.21 to 1963557.07) |
| Republic of Korea | 1744273.18 | 2033017.84 | 1510625.59 | 1744273.18 (1510625.59 to 2033017.84) |
| Colombia | 1986441.61 | 2440337.2 | 1595930.33 | 1986441.61 (1595930.33 to 2440337.2) |
| Australia | 1991546.95 | 2185942.67 | 1832355.86 | 1991546.95 (1832355.86 to 2185942.67) |
| Canada | 2087056.54 | 2418984.98 | 1829339.3 | 2087056.54 (1829339.3 to 2418984.98) |
| Peru | 2116199.36 | 2551587.25 | 1743078.06 | 2116199.36 (1743078.06 to 2551587.25) |
| United Republic of Tanzania | 2130747.88 | 2577407.98 | 1666222.78 | 2130747.88 (1666222.78 to 2577407.98) |
| Spain | 2331162.72 | 2793201.21 | 1864383.67 | 2331162.72 (1864383.67 to 2793201.21) |
| Thailand | 2374837.04 | 2686831.89 | 2064664.04 | 2374837.04 (2064664.04 to 2686831.89) |
| Argentina | 2485800.29 | 2896493.49 | 2152480.43 | 2485800.29 (2152480.43 to 2896493.49) |
| Viet Nam | 2521081.78 | 2974761.45 | 2092652.4 | 2521081.78 (2092652.4 to 2974761.45) |
| Ukraine | 2612391.84 | 3052920.08 | 2213135.44 | 2612391.84 (2213135.44 to 3052920.08) |
| Ethiopia | 2631199.95 | 3173275.6 | 2187630.63 | 2631199.95 (2187630.63 to 3173275.6) |
| Egypt | 2689229.04 | 3106675.79 | 2231152.26 | 2689229.04 (2231152.26 to 3106675.79) |
| Iran (Islamic Republic of) | 2761369.48 | 3293606.07 | 2326442.54 | 2761369.48 (2326442.54 to 3293606.07) |
| Pakistan | 2803677.52 | 3399157.88 | 2357737.34 | 2803677.52 (2357737.34 to 3399157.88) |
| Bangladesh | 3650156.12 | 4241718.68 | 3115661.2 | 3650156.12 (3115661.2 to 4241718.68) |
| Mexico | 4265505.78 | 5290484.4 | 3433406.18 | 4265505.78 (3433406.18 to 5290484.4) |
| Turkiye | 4371813.98 | 5072757.03 | 3781227.95 | 4371813.98 (3781227.95 to 5072757.03) |
| Philippines | 4650480.86 | 5480297.68 | 3993968.47 | 4650480.86 (3993968.47 to 5480297.68) |
| Italy | 4801796.52 | 5503167.24 | 4136024.96 | 4801796.52 (4136024.96 to 5503167.24) |
| Poland | 5444514.05 | 6153988.53 | 4738041.49 | 5444514.05 (4738041.49 to 6153988.53) |
| Nigeria | 5515677.2 | 6738085.77 | 4583461.93 | 5515677.2 (4583461.93 to 6738085.77) |
| France | 6477536.06 | 7323752.58 | 5698883.34 | 6477536.06 (5698883.34 to 7323752.58) |
| Germany | 7736291.83 | 9031238.77 | 6285896.19 | 7736291.83 (6285896.19 to 9031238.77) |
| Indonesia | 7832405.46 | 9252877.58 | 6695243.19 | 7832405.46 (6695243.19 to 9252877.58) |
| Russian Federation | 8882006.69 | 10020580.56 | 7714257.71 | 8882006.69 (7714257.71 to 10020580.56) |
| United Kingdom | 9881245.9 | 11176703.17 | 8632251.46 | 9881245.9 (8632251.46 to 11176703.17) |
| Japan | 12773090.04 | 14593874.29 | 11224699.86 | 12773090.04 (11224699.86 to 14593874.29) |
| Brazil | 12981310.15 | 16295297.59 | 10269717.52 | 12981310.15 (10269717.52 to 16295297.59) |
| United States of America | 24187072.34 | 28342575.36 | 20802816.48 | 24187072.34 (20802816.48 to 28342575.36) |
| China | 33522406.49 | 41409092.76 | 27438105.26 | 33522406.49 (27438105.26 to 41409092.76) |
| India | 35232993.15 | 43227239.13 | 28747320.85 | 35232993.15 (28747320.85 to 43227239.13) |

Supplementary Table 4: The number of global asthma prevalence cases in 2021

| **location** | **val** | **upper** | **lower** | **Number_2021** |
| --- | --- | --- | --- | --- |
| Tokelau | 25.98 | 30.69 | 22.79 | 25.98 (22.79 to 30.69) |
| Niue | 26.84 | 30.72 | 23.88 | 26.84 (23.88 to 30.72) |
| Nauru | 220.47 | 248.81 | 196.79 | 220.47 (196.79 to 248.81) |
| Tuvalu | 239.27 | 269.73 | 212.16 | 239.27 (212.16 to 269.73) |
| Cook Islands | 290.1 | 355.53 | 244.4 | 290.1 (244.4 to 355.53) |
| Palau | 292.72 | 333.03 | 260.09 | 292.72 (260.09 to 333.03) |
| Northern Mariana Islands | 705.62 | 864.27 | 588.87 | 705.62 (588.87 to 864.27) |
| American Samoa | 775.4 | 935.88 | 658.56 | 775.4 (658.56 to 935.88) |
| Marshall Islands | 1170.77 | 1299.35 | 1057.54 | 1170.77 (1057.54 to 1299.35) |
| San Marino | 1976.6 | 2302.75 | 1682.11 | 1976.6 (1682.11 to 2302.75) |
| Seychelles | 1976.75 | 2270.67 | 1728.9 | 1976.75 (1728.9 to 2270.67) |
| Micronesia (Federated States of) | 2177.07 | 2440.75 | 1948.78 | 2177.07 (1948.78 to 2440.75) |
| Guam | 2262.04 | 2820.82 | 1869.24 | 2262.04 (1869.24 to 2820.82) |
| Monaco | 2278.77 | 2621.08 | 1952.94 | 2278.77 (1952.94 to 2621.08) |
| Saint Kitts and Nevis | 2557.95 | 2999.53 | 2144.32 | 2557.95 (2144.32 to 2999.53) |
| Bermuda | 2729.79 | 3252.34 | 2292.2 | 2729.79 (2292.2 to 3252.34) |
| Tonga | 3510.85 | 4190.36 | 2940.04 | 3510.85 (2940.04 to 4190.36) |
| Dominica | 3850.4 | 4435.35 | 3392.4 | 3850.4 (3392.4 to 4435.35) |
| Kiribati | 4031.85 | 4316.53 | 3744.2 | 4031.85 (3744.2 to 4316.53) |
| Antigua and Barbuda | 4241.9 | 5075.06 | 3529.42 | 4241.9 (3529.42 to 5075.06) |
| Greenland | 4313.29 | 4825 | 3833.44 | 4313.29 (3833.44 to 4825) |
| Samoa | 4431.61 | 5284.21 | 3745.72 | 4431.61 (3745.72 to 5284.21) |
| United States Virgin Islands | 4525.84 | 5316.53 | 3887.63 | 4525.84 (3887.63 to 5316.53) |
| Andorra | 5666.32 | 6579.85 | 4770.77 | 5666.32 (4770.77 to 6579.85) |
| Saint Vincent and the Grenadines | 5701.64 | 6687.38 | 4866.36 | 5701.64 (4866.36 to 6687.38) |
| Vanuatu | 6645.58 | 7352.15 | 5984.5 | 6645.58 (5984.5 to 7352.15) |
| Maldives | 7817 | 9509.44 | 6499.78 | 7817 (6499.78 to 9509.44) |
| Grenada | 7940.9 | 9578.78 | 6518.43 | 7940.9 (6518.43 to 9578.78) |
| Sao Tome and Principe | 8256.72 | 9400.12 | 7182.26 | 8256.72 (7182.26 to 9400.12) |
| Saint Lucia | 10498.61 | 11719.76 | 9294.85 | 10498.61 (9294.85 to 11719.76) |
| Bhutan | 11377.31 | 13301.14 | 9843.07 | 11377.31 (9843.07 to 13301.14) |
| Cabo Verde | 11748.2 | 14445.48 | 9749.03 | 11748.2 (9749.03 to 14445.48) |
| Solomon Islands | 14704.52 | 16452.4 | 13196.73 | 14704.52 (13196.73 to 16452.4) |
| Brunei Darussalam | 16753.02 | 19191.89 | 14614.2 | 16753.02 (14614.2 to 19191.89) |
| Bahamas | 19695.5 | 23328.11 | 16494.77 | 19695.5 (16494.77 to 23328.11) |
| Montenegro | 20060.42 | 24168.74 | 16819.37 | 20060.42 (16819.37 to 24168.74) |
| Barbados | 21144.7 | 24334.68 | 18374.56 | 21144.7 (18374.56 to 24334.68) |
| Fiji | 21585.66 | 23989.11 | 19224.06 | 21585.66 (19224.06 to 23989.11) |
| Lesotho | 24171.38 | 27607.53 | 21008.58 | 24171.38 (21008.58 to 27607.53) |
| Iceland | 25077.83 | 30057.15 | 20694.22 | 25077.83 (20694.22 to 30057.15) |
| Belize | 25765.33 | 30356.02 | 21784.46 | 25765.33 (21784.46 to 30356.02) |
| Suriname | 28066.88 | 33318.53 | 23888.59 | 28066.88 (23888.59 to 33318.53) |
| Malta | 30993.29 | 35983.87 | 26083.6 | 30993.29 (26083.6 to 35983.87) |
| Comoros | 33283.49 | 38413.34 | 28376.29 | 33283.49 (28376.29 to 38413.34) |
| Mauritius | 34136.13 | 38451.09 | 30167.88 | 34136.13 (30167.88 to 38451.09) |
| Estonia | 36062.91 | 41844.06 | 31525.32 | 36062.91 (31525.32 to 41844.06) |
| Equatorial Guinea | 38741 | 47872.36 | 31661.3 | 38741 (31661.3 to 47872.36) |
| Trinidad and Tobago | 40460.44 | 46911.36 | 34437.46 | 40460.44 (34437.46 to 46911.36) |
| Bahrain | 42022.18 | 49184.35 | 35681.02 | 42022.18 (35681.02 to 49184.35) |
| Timor-Leste | 42182.3 | 47711.34 | 37442.65 | 42182.3 (37442.65 to 47711.34) |
| Djibouti | 44142.24 | 51517.32 | 37934.08 | 44142.24 (37934.08 to 51517.32) |
| Guyana | 44806.94 | 51376.25 | 39153.24 | 44806.94 (39153.24 to 51376.25) |
| Armenia | 46769.75 | 57494.68 | 38432.72 | 46769.75 (38432.72 to 57494.68) |
| Luxembourg | 46871.07 | 55220.63 | 39749.01 | 46871.07 (39749.01 to 55220.63) |
| Gabon | 48014.59 | 58803.09 | 39665.29 | 48014.59 (39665.29 to 58803.09) |
| Latvia | 54580.4 | 62876.38 | 47244.55 | 54580.4 (47244.55 to 62876.38) |
| Namibia | 57918.54 | 68745.7 | 49232.46 | 57918.54 (49232.46 to 68745.7) |
| Gambia | 65290.97 | 76810.78 | 55312.65 | 65290.97 (55312.65 to 76810.78) |
| Eswatini | 65608.44 | 74945.5 | 56916.83 | 65608.44 (56916.83 to 74945.5) |
| Georgia | 66337.12 | 77817.89 | 56949.74 | 66337.12 (56949.74 to 77817.89) |
| Guinea-Bissau | 67302.12 | 77249.96 | 58433.73 | 67302.12 (58433.73 to 77249.96) |
| Albania | 68031.35 | 77659.19 | 58996.99 | 68031.35 (58996.99 to 77659.19) |
| Lithuania | 70482.43 | 82053.42 | 60723.87 | 70482.43 (60723.87 to 82053.42) |
| Mongolia | 71715.9 | 86282.25 | 60545.43 | 71715.9 (60545.43 to 86282.25) |
| Botswana | 77370.81 | 89563.09 | 67482.89 | 77370.81 (67482.89 to 89563.09) |
| Qatar | 81486.95 | 98986.76 | 66505.9 | 81486.95 (66505.9 to 98986.76) |
| Republic of Moldova | 85208.21 | 100536.42 | 72169.94 | 85208.21 (72169.94 to 100536.42) |
| Turkmenistan | 91343.01 | 111923.98 | 74878.06 | 91343.01 (74878.06 to 111923.98) |
| Cyprus | 107254.8 | 130586.58 | 86795.04 | 107254.8 (86795.04 to 130586.58) |
| Slovenia | 110591.28 | 127033.19 | 94886.23 | 110591.28 (94886.23 to 127033.19) |
| North Macedonia | 130636.26 | 148028.47 | 115715.29 | 130636.26 (115715.29 to 148028.47) |
| Liberia | 134958.59 | 161018.26 | 112642.03 | 134958.59 (112642.03 to 161018.26) |
| Kuwait | 140894.98 | 170104.22 | 116215.8 | 140894.98 (116215.8 to 170104.22) |
| Lao People's Democratic Republic | 144557.41 | 163142.36 | 126937.4 | 144557.41 (126937.4 to 163142.36) |
| Jamaica | 148443.55 | 172362.18 | 126423.06 | 148443.55 (126423.06 to 172362.18) |
| Slovakia | 150180.97 | 175529.73 | 128616.89 | 150180.97 (128616.89 to 175529.73) |
| Bosnia and Herzegovina | 162802.06 | 187501.23 | 141030.3 | 162802.06 (141030.3 to 187501.23) |
| Oman | 163192.52 | 202426.92 | 131238.82 | 163192.52 (131238.82 to 202426.92) |
| Croatia | 171419.81 | 195903.76 | 148588.34 | 171419.81 (148588.34 to 195903.76) |
| Kyrgyzstan | 177778.08 | 215322.03 | 146589.36 | 177778.08 (146589.36 to 215322.03) |
| Congo | 177995.13 | 211721.65 | 152001.87 | 177995.13 (152001.87 to 211721.65) |
| Singapore | 178748.33 | 209889.76 | 153312.42 | 178748.33 (153312.42 to 209889.76) |
| Uruguay | 185203.43 | 211266.99 | 162898.36 | 185203.43 (162898.36 to 211266.99) |
| Palestine | 185374.18 | 228810.43 | 149383.58 | 185374.18 (149383.58 to 228810.43) |
| Panama | 193524.06 | 230375.2 | 158871.01 | 193524.06 (158871.01 to 230375.2) |
| Tajikistan | 206043.59 | 252927.69 | 170950.84 | 206043.59 (170950.84 to 252927.69) |
| Libya | 210481.75 | 240891.92 | 184589.78 | 210481.75 (184589.78 to 240891.92) |
| Bulgaria | 219692.54 | 256753.22 | 185950.34 | 219692.54 (185950.34 to 256753.22) |
| Mauritania | 219755.59 | 269457.12 | 180129.35 | 219755.59 (180129.35 to 269457.12) |
| Central African Republic | 223755.63 | 251961.17 | 197205.81 | 223755.63 (197205.81 to 251961.17) |
| Azerbaijan | 224957.16 | 259920.47 | 193193.12 | 224957.16 (193193.12 to 259920.47) |
| Lebanon | 234181.59 | 276841.01 | 199120.92 | 234181.59 (199120.92 to 276841.01) |
| Puerto Rico | 245585.88 | 284907.22 | 209114.67 | 245585.88 (209114.67 to 284907.22) |
| Denmark | 261144.59 | 303391.97 | 220244.53 | 261144.59 (220244.53 to 303391.97) |
| Costa Rica | 265529.4 | 320816.18 | 218638.81 | 265529.4 (218638.81 to 320816.18) |
| Nicaragua | 271311.04 | 331165.9 | 222183.51 | 271311.04 (222183.51 to 331165.9) |
| Serbia | 278989.13 | 321013.38 | 245435.03 | 278989.13 (245435.03 to 321013.38) |
| Czechia | 282642.09 | 332841.75 | 242271.05 | 282642.09 (242271.05 to 332841.75) |
| Hungary | 284434.39 | 330738.49 | 247438.26 | 284434.39 (247438.26 to 330738.49) |
| Papua New Guinea | 291767.16 | 319889.37 | 265571.17 | 291767.16 (265571.17 to 319889.37) |
| Togo | 296488.37 | 361429.45 | 236603.29 | 296488.37 (236603.29 to 361429.45) |
| Eritrea | 297885.81 | 338804.7 | 258895.29 | 297885.81 (258895.29 to 338804.7) |
| Kazakhstan | 298686.18 | 351161.97 | 255607.34 | 298686.18 (255607.34 to 351161.97) |
| Sierra Leone | 304034.54 | 349773.44 | 263421.32 | 304034.54 (263421.32 to 349773.44) |
| Norway | 322345.93 | 377799.75 | 271892.7 | 322345.93 (271892.7 to 377799.75) |
| Zimbabwe | 326782.88 | 386127.39 | 277482.63 | 326782.88 (277482.63 to 386127.39) |
| Ireland | 335299.09 | 394707.88 | 284060.83 | 335299.09 (284060.83 to 394707.88) |
| Cambodia | 350490.73 | 398280.7 | 308724.9 | 350490.73 (308724.9 to 398280.7) |
| Honduras | 353515.69 | 416809.9 | 297120.45 | 353515.69 (297120.45 to 416809.9) |
| Senegal | 360067.24 | 433052.58 | 301869.31 | 360067.24 (301869.31 to 433052.58) |
| Israel | 360753.74 | 425802.18 | 303238.14 | 360753.74 (303238.14 to 425802.18) |
| New Zealand | 363457.49 | 417078.26 | 314965.96 | 363457.49 (314965.96 to 417078.26) |
| Finland | 365763.05 | 419311.27 | 314278.5 | 365763.05 (314278.5 to 419311.27) |
| Tunisia | 372830.95 | 430068.26 | 327075.23 | 372830.95 (327075.23 to 430068.26) |
| Paraguay | 375545.38 | 466053.38 | 298681.55 | 375545.38 (298681.55 to 466053.38) |
| El Salvador | 382455.93 | 464962.03 | 313818.15 | 382455.93 (313818.15 to 464962.03) |
| Dominican Republic | 383351.85 | 439321.14 | 330877.78 | 383351.85 (330877.78 to 439321.14) |
| Bolivia (Plurinational State of) | 396709.08 | 486790.48 | 327606.24 | 396709.08 (327606.24 to 486790.48) |
| Jordan | 400498.6 | 490798.12 | 327345.5 | 400498.6 (327345.5 to 490798.12) |
| Belarus | 402787.81 | 466336.99 | 341222.04 | 402787.81 (341222.04 to 466336.99) |
| Austria | 426035.02 | 497250.38 | 357010.59 | 426035.02 (357010.59 to 497250.38) |
| Benin | 432180.95 | 504997.22 | 369909.97 | 432180.95 (369909.97 to 504997.22) |
| Zambia | 442651.49 | 531739.3 | 367314.94 | 442651.49 (367314.94 to 531739.3) |
| Guatemala | 451521.16 | 560282.12 | 370047.22 | 451521.16 (370047.22 to 560282.12) |
| Nepal | 485135.5 | 565987.96 | 424931.27 | 485135.5 (424931.27 to 565987.96) |
| Greece | 485298 | 568208.7 | 409487.18 | 485298 (409487.18 to 568208.7) |
| South Sudan | 490750.49 | 572983.21 | 414500.82 | 490750.49 (414500.82 to 572983.21) |
| Chad | 491473.2 | 587146.52 | 409518.2 | 491473.2 (409518.2 to 587146.52) |
| Guinea | 499002.3 | 594566.84 | 415976.1 | 499002.3 (415976.1 to 594566.84) |
| Switzerland | 501424.04 | 592281.05 | 414361.06 | 501424.04 (414361.06 to 592281.05) |
| Belgium | 518847.3 | 603270.29 | 447793.28 | 518847.3 (447793.28 to 603270.29) |
| Mali | 537787.24 | 659280.68 | 438133.43 | 537787.24 (438133.43 to 659280.68) |
| Syrian Arab Republic | 560223.4 | 634481.63 | 488742.62 | 560223.4 (488742.62 to 634481.63) |
| Cameroon | 564274.61 | 687436.22 | 458120.04 | 564274.61 (458120.04 to 687436.22) |
| Ecuador | 599833.22 | 774783.67 | 480800.12 | 599833.22 (480800.12 to 774783.67) |
| Cuba | 640611.03 | 761999.7 | 543284.94 | 640611.03 (543284.94 to 761999.7) |
| Ghana | 699441.58 | 833131.07 | 593131 | 699441.58 (593131 to 833131.07) |
| Burundi | 724825.24 | 827070.5 | 640048.37 | 724825.24 (640048.37 to 827070.5) |
| Malaysia | 747217.94 | 868425.38 | 637322.22 | 747217.94 (637322.22 to 868425.38) |
| Sweden | 790009.36 | 923930.35 | 664613.14 | 790009.36 (664613.14 to 923930.35) |
| Romania | 791568.96 | 922752.46 | 675347.64 | 791568.96 (675347.64 to 922752.46) |
| Sri Lanka | 808085.91 | 900231.71 | 724092.27 | 808085.91 (724092.27 to 900231.71) |
| United Arab Emirates | 817564.46 | 924738.25 | 721608.89 | 817564.46 (721608.89 to 924738.25) |
| Malawi | 867181.41 | 1031961.96 | 714469.24 | 867181.41 (714469.24 to 1031961.96) |
| Morocco | 878175.41 | 1010863.23 | 764202.94 | 878175.41 (764202.94 to 1010863.23) |
| South Africa | 922549.64 | 1080751 | 798404.74 | 922549.64 (798404.74 to 1080751) |
| Saudi Arabia | 931326.68 | 1096047.45 | 794363.7 | 931326.68 (794363.7 to 1096047.45) |
| Burkina Faso | 933166.65 | 1109155.69 | 779704.92 | 933166.65 (779704.92 to 1109155.69) |
| Cote d'Ivoire | 945390.42 | 1120918.58 | 798522.64 | 945390.42 (798522.64 to 1120918.58) |
| Angola | 956747.78 | 1141366.42 | 808456.42 | 956747.78 (808456.42 to 1141366.42) |
| Myanmar | 982998.33 | 1114480.03 | 873706.94 | 982998.33 (873706.94 to 1114480.03) |
| Niger | 986351.69 | 1149192.46 | 834822.85 | 986351.69 (834822.85 to 1149192.46) |
| Ukraine | 1029856.29 | 1231628.97 | 854257.66 | 1029856.29 (854257.66 to 1231628.97) |
| Venezuela (Bolivarian Republic of) | 1035139.15 | 1239537.53 | 866419.38 | 1035139.15 (866419.38 to 1239537.53) |
| Democratic People's Republic of Korea | 1068931.78 | 1200839.99 | 952970.36 | 1068931.78 (952970.36 to 1200839.99) |
| Portugal | 1082621.81 | 1263504.74 | 919805.06 | 1082621.81 (919805.06 to 1263504.74) |
| Taiwan (Province of China) | 1093185.77 | 1285484.05 | 962526.07 | 1093185.77 (962526.07 to 1285484.05) |
| Sudan | 1111282.91 | 1309057.38 | 935566.37 | 1111282.91 (935566.37 to 1309057.38) |
| Netherlands | 1161085.57 | 1340509.24 | 988268.02 | 1161085.57 (988268.02 to 1340509.24) |
| Yemen | 1226866.05 | 1409944.81 | 1072954.11 | 1226866.05 (1072954.11 to 1409944.81) |
| Uzbekistan | 1233829.73 | 1411716.02 | 1069361.38 | 1233829.73 (1069361.38 to 1411716.02) |
| Chile | 1234063.81 | 1426376.25 | 1074246.68 | 1234063.81 (1074246.68 to 1426376.25) |
| Afghanistan | 1258963.72 | 1420429.97 | 1122508.69 | 1258963.72 (1122508.69 to 1420429.97) |
| Mozambique | 1272448.17 | 1496939.72 | 1085151.91 | 1272448.17 (1085151.91 to 1496939.72) |
| Somalia | 1289512.9 | 1460772.83 | 1137058.37 | 1289512.9 (1137058.37 to 1460772.83) |
| Rwanda | 1289676.56 | 1501025.91 | 1092998.27 | 1289676.56 (1092998.27 to 1501025.91) |
| Iraq | 1293149.36 | 1533617.19 | 1084134.61 | 1293149.36 (1084134.61 to 1533617.19) |
| Algeria | 1304205.95 | 1565302.03 | 1103790.9 | 1304205.95 (1103790.9 to 1565302.03) |
| Kenya | 1326566.12 | 1583411.99 | 1117751.49 | 1326566.12 (1117751.49 to 1583411.99) |
| Republic of Korea | 1497054.32 | 1735538.73 | 1310903 | 1497054.32 (1310903 to 1735538.73) |
| Colombia | 1545590.56 | 1894948.87 | 1251859.25 | 1545590.56 (1251859.25 to 1894948.87) |
| Haiti | 1605068.59 | 1754327.09 | 1450586.07 | 1605068.59 (1450586.07 to 1754327.09) |
| Peru | 1610227.37 | 2029034.12 | 1276356.78 | 1610227.37 (1276356.78 to 2029034.12) |
| Australia | 1891455.5 | 2162497.98 | 1615478.16 | 1891455.5 (1615478.16 to 2162497.98) |
| Thailand | 1977603.47 | 2247844.74 | 1758316.67 | 1977603.47 (1758316.67 to 2247844.74) |
| Canada | 1980778.36 | 2299108.79 | 1696599.64 | 1980778.36 (1696599.64 to 2299108.79) |
| Spain | 2002159.89 | 2302025.66 | 1715501.1 | 2002159.89 (1715501.1 to 2302025.66) |
| Madagascar | 2177976.14 | 2438875.83 | 1938819.19 | 2177976.14 (1938819.19 to 2438875.83) |
| Italy | 2215451.12 | 2616370.53 | 1852903.6 | 2215451.12 (1852903.6 to 2616370.53) |
| Iran (Islamic Republic of) | 2311547.23 | 2675699.35 | 1994962.54 | 2311547.23 (1994962.54 to 2675699.35) |
| Democratic Republic of the Congo | 2544658.14 | 3011473.02 | 2163633.7 | 2544658.14 (2163633.7 to 3011473.02) |
| Uganda | 2622505.52 | 3090994.4 | 2143998.36 | 2622505.52 (2143998.36 to 3090994.4) |
| Argentina | 2754216.61 | 3257775.42 | 2339848.06 | 2754216.61 (2339848.06 to 3257775.42) |
| Ethiopia | 2793527.82 | 3369142.1 | 2328796.41 | 2793527.82 (2328796.41 to 3369142.1) |
| Bangladesh | 2869001.4 | 3437304.32 | 2431749.95 | 2869001.4 (2431749.95 to 3437304.32) |
| Viet Nam | 2909596.62 | 3326501.66 | 2527150.62 | 2909596.62 (2527150.62 to 3326501.66) |
| Mexico | 2930938 | 3691675.86 | 2350427.05 | 2930938 (2350427.05 to 3691675.86) |
| Russian Federation | 3195040.1 | 3778497.29 | 2673037.67 | 3195040.1 (2673037.67 to 3778497.29) |
| Egypt | 3228376.26 | 3797510.93 | 2751479 | 3228376.26 (2751479 to 3797510.93) |
| Poland | 3359392.22 | 3813600.24 | 2948448.63 | 3359392.22 (2948448.63 to 3813600.24) |
| Germany | 3545438.79 | 4091324.15 | 3019749.11 | 3545438.79 (3019749.11 to 4091324.15) |
| Pakistan | 3589530.35 | 4303358.23 | 3035828.37 | 3589530.35 (3035828.37 to 4303358.23) |
| Turkiye | 3976397.92 | 4593714.28 | 3461690.45 | 3976397.92 (3461690.45 to 4593714.28) |
| United Republic of Tanzania | 4184506.2 | 5049834.35 | 3280327.52 | 4184506.2 (3280327.52 to 5049834.35) |
| France | 4208128.21 | 4847310.22 | 3580292.95 | 4208128.21 (3580292.95 to 4847310.22) |
| Japan | 4315281.16 | 4942772.3 | 3742172.5 | 4315281.16 (3742172.5 to 4942772.3) |
| Philippines | 5175229.52 | 5818700.6 | 4617803.88 | 5175229.52 (4617803.88 to 5818700.6) |
| Indonesia | 6333479.18 | 7046859.49 | 5651616.88 | 6333479.18 (5651616.88 to 7046859.49) |
| United Kingdom | 6690900.21 | 7640079.03 | 5780039.94 | 6690900.21 (5780039.94 to 7640079.03) |
| Brazil | 9179529.71 | 11228159.36 | 7516873.48 | 9179529.71 (7516873.48 to 11228159.36) |
| Nigeria | 11105770.43 | 13439541.42 | 9195594.42 | 11105770.43 (9195594.42 to 13439541.42) |
| China | 25015668.03 | 30448108.18 | 20898413.74 | 25015668.03 (20898413.74 to 30448108.18) |
| India | 32054679.86 | 36883861.75 | 27775103.58 | 32054679.86 (27775103.58 to 36883861.75) |
| United States of America | 33328621.57 | 37213730.47 | 29835744.05 | 33328621.57 (29835744.05 to 37213730.47) |

Supplementary Table 5: The number of global asthma YLDs cases in 1990

| **location** | **val** | **upper** | **lower** | **Number_1990** |
| --- | --- | --- | --- | --- |
| Tokelau | 2.22 | 3.32 | 1.44 | 2.22 (1.44 to 3.32) |
| Niue | 2.72 | 4.12 | 1.76 | 2.72 (1.76 to 4.12) |
| Nauru | 14.97 | 22.13 | 9.67 | 14.97 (9.67 to 22.13) |
| Tuvalu | 16.9 | 24.22 | 11.07 | 16.9 (11.07 to 24.22) |
| Palau | 18.55 | 28.13 | 11.89 | 18.55 (11.89 to 28.13) |
| Cook Islands | 19.86 | 30.29 | 12.54 | 19.86 (12.54 to 30.29) |
| Northern Mariana Islands | 42.87 | 65.05 | 26.94 | 42.87 (26.94 to 65.05) |
| American Samoa | 49.07 | 75.07 | 30.87 | 49.07 (30.87 to 75.07) |
| Marshall Islands | 58.17 | 87.45 | 37.39 | 58.17 (37.39 to 87.45) |
| San Marino | 64.84 | 95.76 | 41.58 | 64.84 (41.58 to 95.76) |
| Seychelles | 74 | 112.71 | 46.8 | 74 (46.8 to 112.71) |
| Monaco | 85.29 | 125.83 | 54.85 | 85.29 (54.85 to 125.83) |
| Saint Kitts and Nevis | 106.87 | 162.76 | 66.83 | 106.87 (66.83 to 162.76) |
| Bermuda | 139.04 | 207.27 | 86.59 | 139.04 (86.59 to 207.27) |
| Guam | 153.57 | 237.54 | 93.73 | 153.57 (93.73 to 237.54) |
| Micronesia (Federated States of) | 154.39 | 231.39 | 101.22 | 154.39 (101.22 to 231.39) |
| Antigua and Barbuda | 159.14 | 246.76 | 98.81 | 159.14 (98.81 to 246.76) |
| Kiribati | 162.02 | 235.02 | 104.88 | 162.02 (104.88 to 235.02) |
| Tonga | 167.42 | 252.7 | 105.9 | 167.42 (105.9 to 252.7) |
| Samoa | 194.23 | 294.75 | 119.96 | 194.23 (119.96 to 294.75) |
| Dominica | 207.11 | 317.46 | 130.51 | 207.11 (130.51 to 317.46) |
| Vanuatu | 208.66 | 308.34 | 135.81 | 208.66 (135.81 to 308.34) |
| Andorra | 215.24 | 314.7 | 133.86 | 215.24 (133.86 to 314.7) |
| Greenland | 233.32 | 345.76 | 149.98 | 233.32 (149.98 to 345.76) |
| Sao Tome and Principe | 276.69 | 409.28 | 173.19 | 276.69 (173.19 to 409.28) |
| Saint Vincent and the Grenadines | 299.22 | 454.42 | 183.06 | 299.22 (183.06 to 454.42) |
| United States Virgin Islands | 307.2 | 469.87 | 189.85 | 307.2 (189.85 to 469.87) |
| Maldives | 382.25 | 580.22 | 238.69 | 382.25 (238.69 to 580.22) |
| Grenada | 394.84 | 609.67 | 238.64 | 394.84 (238.64 to 609.67) |
| Solomon Islands | 413.94 | 623.4 | 265.58 | 413.94 (265.58 to 623.4) |
| Saint Lucia | 488.39 | 729.56 | 302.38 | 488.39 (302.38 to 729.56) |
| Brunei Darussalam | 500.52 | 771.59 | 313.65 | 500.52 (313.65 to 771.59) |
| Cabo Verde | 522.18 | 801.49 | 330.34 | 522.18 (330.34 to 801.49) |
| Bhutan | 609.79 | 927.9 | 384.48 | 609.79 (384.48 to 927.9) |
| Belize | 641.8 | 962.67 | 395.44 | 641.8 (395.44 to 962.67) |
| Qatar | 663.92 | 991.6 | 418.4 | 663.92 (418.4 to 991.6) |
| Bahamas | 674.86 | 1013.78 | 411.9 | 674.86 (411.9 to 1013.78) |
| Barbados | 777.35 | 1199.55 | 481.5 | 777.35 (481.5 to 1199.55) |
| Equatorial Guinea | 850.33 | 1286.29 | 548.39 | 850.33 (548.39 to 1286.29) |
| Bahrain | 857.77 | 1255.01 | 544.4 | 857.77 (544.4 to 1255.01) |
| Montenegro | 923.53 | 1406.18 | 568.27 | 923.53 (568.27 to 1406.18) |
| Suriname | 975.91 | 1468.63 | 595.14 | 975.91 (595.14 to 1468.63) |
| Djibouti | 978.33 | 1473.44 | 593.37 | 978.33 (593.37 to 1473.44) |
| Lesotho | 1057.93 | 1583.27 | 681.19 | 1057.93 (681.19 to 1583.27) |
| Fiji | 1148.03 | 1700.02 | 744.53 | 1148.03 (744.53 to 1700.02) |
| Iceland | 1172.12 | 1764.69 | 742.5 | 1172.12 (742.5 to 1764.69) |
| Comoros | 1262.99 | 1921.33 | 782.41 | 1262.99 (782.41 to 1921.33) |
| Gambia | 1390.6 | 2128.64 | 875.45 | 1390.6 (875.45 to 2128.64) |
| Malta | 1504.94 | 2196.41 | 957.35 | 1504.94 (957.35 to 2196.41) |
| Gabon | 1595.77 | 2412.49 | 986.32 | 1595.77 (986.32 to 2412.49) |
| Timor-Leste | 1645.7 | 2451.09 | 1026.45 | 1645.7 (1026.45 to 2451.09) |
| Luxembourg | 1845.3 | 2735.63 | 1179.45 | 1845.3 (1179.45 to 2735.63) |
| Namibia | 1858.33 | 2840.43 | 1154.05 | 1858.33 (1154.05 to 2840.43) |
| Mauritius | 2035.01 | 3039.82 | 1274.18 | 2035.01 (1274.18 to 3039.82) |
| Guyana | 2091.56 | 3165.83 | 1295.82 | 2091.56 (1295.82 to 3165.83) |
| Guinea-Bissau | 2115.4 | 3160.15 | 1372.92 | 2115.4 (1372.92 to 3160.15) |
| Botswana | 2284.09 | 3522.31 | 1482.05 | 2284.09 (1482.05 to 3522.31) |
| Trinidad and Tobago | 2335.9 | 3577.23 | 1432.93 | 2335.9 (1432.93 to 3577.23) |
| Mongolia | 2431.94 | 3750.53 | 1546.46 | 2431.94 (1546.46 to 3750.53) |
| Estonia | 2530.09 | 3656.06 | 1654.25 | 2530.09 (1654.25 to 3656.06) |
| Eswatini | 2538.47 | 3865.36 | 1635.96 | 2538.47 (1635.96 to 3865.36) |
| Oman | 2746.63 | 4150.5 | 1684.23 | 2746.63 (1684.23 to 4150.5) |
| Armenia | 2818.68 | 4253.53 | 1746.27 | 2818.68 (1746.27 to 4253.53) |
| Cyprus | 2883.48 | 4296.81 | 1778.62 | 2883.48 (1778.62 to 4296.81) |
| Kuwait | 3002.34 | 4430.64 | 1890.87 | 3002.34 (1890.87 to 4430.64) |
| Palestine | 3714.16 | 5588.51 | 2240.52 | 3714.16 (2240.52 to 5588.51) |
| Liberia | 3868.16 | 5797.23 | 2413 | 3868.16 (2413 to 5797.23) |
| Albania | 3907.2 | 5764.7 | 2454.5 | 3907.2 (2454.5 to 5764.7) |
| Congo | 5016.12 | 7699.64 | 3145.71 | 5016.12 (3145.71 to 7699.64) |
| Lithuania | 5387.23 | 7880.81 | 3413.68 | 5387.23 (3413.68 to 7880.81) |
| Mauritania | 5427.85 | 8236.02 | 3436.44 | 5427.85 (3436.44 to 8236.02) |
| Latvia | 5515.1 | 8078.81 | 3489.49 | 5515.1 (3489.49 to 8078.81) |
| Tajikistan | 5575.12 | 8378.83 | 3495.03 | 5575.12 (3495.03 to 8378.83) |
| Georgia | 5710.36 | 8467.72 | 3587.54 | 5710.36 (3587.54 to 8467.72) |
| Central African Republic | 5793.26 | 8745.28 | 3730.98 | 5793.26 (3730.98 to 8745.28) |
| Slovenia | 6002.68 | 8627.8 | 3861.01 | 6002.68 (3861.01 to 8627.8) |
| Togo | 6185.77 | 9489.11 | 3791.05 | 6185.77 (3791.05 to 9489.11) |
| Lebanon | 6273.45 | 9093.95 | 4012.3 | 6273.45 (4012.3 to 9093.95) |
| North Macedonia | 6305.97 | 9078.33 | 4036.83 | 6305.97 (4036.83 to 9078.33) |
| Jordan | 6441.21 | 9535.95 | 3985.62 | 6441.21 (3985.62 to 9535.95) |
| Turkmenistan | 6495.12 | 9854.22 | 4038.63 | 6495.12 (4038.63 to 9854.22) |
| Lao People's Democratic Republic | 6614.05 | 9931.57 | 4164.48 | 6614.05 (4164.48 to 9931.57) |
| Kyrgyzstan | 6668.82 | 9867.11 | 4144.3 | 6668.82 (4144.3 to 9867.11) |
| United Arab Emirates | 6785.5 | 10150.52 | 4345.59 | 6785.5 (4345.59 to 10150.52) |
| Slovakia | 7131.57 | 10564.24 | 4421.69 | 7131.57 (4421.69 to 10564.24) |
| Libya | 7160.17 | 10624.23 | 4494.8 | 7160.17 (4494.8 to 10624.23) |
| Sierra Leone | 7413.03 | 11162 | 4702.51 | 7413.03 (4702.51 to 11162) |
| Papua New Guinea | 7502.07 | 11071.01 | 4909.12 | 7502.07 (4909.12 to 11071.01) |
| Chad | 7518.06 | 11587.12 | 4695.77 | 7518.06 (4695.77 to 11587.12) |
| Panama | 7542.33 | 11255.46 | 4585.87 | 7542.33 (4585.87 to 11255.46) |
| Republic of Moldova | 7951.2 | 11756.13 | 5069.25 | 7951.2 (5069.25 to 11756.13) |
| Azerbaijan | 8006.8 | 12100.63 | 5146.38 | 8006.8 (5146.38 to 12100.63) |
| Jamaica | 8115.2 | 12223.12 | 5010.35 | 8115.2 (5010.35 to 12223.12) |
| Singapore | 8657.6 | 12863.66 | 5518.23 | 8657.6 (5518.23 to 12863.66) |
| Benin | 8796.12 | 13212.69 | 5586.26 | 8796.12 (5586.26 to 13212.69) |
| Mali | 9072.14 | 13579.11 | 5674.18 | 9072.14 (5674.18 to 13579.11) |
| Uruguay | 9162.24 | 13452.03 | 5971.23 | 9162.24 (5971.23 to 13452.03) |
| Bosnia and Herzegovina | 9498.42 | 13718.52 | 5957.45 | 9498.42 (5957.45 to 13718.52) |
| Cameroon | 9543.24 | 14585.94 | 5836.39 | 9543.24 (5836.39 to 14585.94) |
| Zimbabwe | 9613.44 | 14799.11 | 6009.39 | 9613.44 (6009.39 to 14799.11) |
| Senegal | 9799.59 | 15049.78 | 6149.63 | 9799.59 (6149.63 to 15049.78) |
| Costa Rica | 10233.05 | 15184.93 | 6541.86 | 10233.05 (6541.86 to 15184.93) |
| Zambia | 10389.56 | 15808.86 | 6387.7 | 10389.56 (6387.7 to 15808.86) |
| Eritrea | 10773.99 | 16342.9 | 6871.74 | 10773.99 (6871.74 to 16342.9) |
| Kazakhstan | 10915.79 | 16372.72 | 6929.09 | 10915.79 (6929.09 to 16372.72) |
| Paraguay | 11068.02 | 16494.41 | 6879.72 | 11068.02 (6879.72 to 16494.41) |
| Guinea | 11448.91 | 17598.51 | 7199.83 | 11448.91 (7199.83 to 17598.51) |
| Israel | 12169.07 | 17970.93 | 7742.1 | 12169.07 (7742.1 to 17970.93) |
| Croatia | 12309.3 | 17888.06 | 7805.45 | 12309.3 (7805.45 to 17888.06) |
| Tunisia | 13123.92 | 19284.53 | 8230.61 | 13123.92 (8230.61 to 19284.53) |
| Cambodia | 13637.52 | 20651.49 | 8658.06 | 13637.52 (8658.06 to 20651.49) |
| Czechia | 14133.88 | 20836.36 | 8784.43 | 14133.88 (8784.43 to 20836.36) |
| Nicaragua | 14242.24 | 21338.83 | 8524.53 | 14242.24 (8524.53 to 21338.83) |
| Nepal | 14563.56 | 22021.18 | 9226.57 | 14563.56 (9226.57 to 22021.18) |
| Denmark | 15598.43 | 22754.31 | 9998.15 | 15598.43 (9998.15 to 22754.31) |
| Serbia | 15875.07 | 22946.1 | 10080.18 | 15875.07 (10080.18 to 22946.1) |
| Finland | 15908.02 | 23460.06 | 10309.81 | 15908.02 (10309.81 to 23460.06) |
| Ghana | 15990.88 | 24585 | 10164.96 | 15990.88 (10164.96 to 24585) |
| Honduras | 16555.85 | 25020.19 | 10172.73 | 16555.85 (10172.73 to 25020.19) |
| Puerto Rico | 16929.33 | 25700.6 | 10736.83 | 16929.33 (10736.83 to 25700.6) |
| Niger | 17174.19 | 26097.03 | 10940.28 | 17174.19 (10940.28 to 26097.03) |
| Saudi Arabia | 17220.05 | 25984.2 | 10604.42 | 17220.05 (10604.42 to 25984.2) |
| Burkina Faso | 17600.28 | 26431.15 | 11189.54 | 17600.28 (11189.54 to 26431.15) |
| Dominican Republic | 17690.3 | 26770.75 | 10787.56 | 17690.3 (10787.56 to 26770.75) |
| Ireland | 17945.77 | 26583.11 | 11707.37 | 17945.77 (11707.37 to 26583.11) |
| South Sudan | 18018.69 | 27471.92 | 11228.88 | 18018.69 (11228.88 to 27471.92) |
| Hungary | 18617.01 | 27307.05 | 11839.46 | 18617.01 (11839.46 to 27307.05) |
| Bulgaria | 18649.87 | 27261.36 | 11927.35 | 18649.87 (11927.35 to 27261.36) |
| Cote d'Ivoire | 19661.06 | 30044.72 | 12383.31 | 19661.06 (12383.31 to 30044.72) |
| Syrian Arab Republic | 21575.69 | 31813.39 | 13423.99 | 21575.69 (13423.99 to 31813.39) |
| Bolivia (Plurinational State of) | 21627.51 | 32611.13 | 13148.17 | 21627.51 (13148.17 to 32611.13) |
| Angola | 21825.98 | 33031.98 | 13817.35 | 21825.98 (13817.35 to 33031.98) |
| Burundi | 21958.22 | 33093.96 | 13908.94 | 21958.22 (13908.94 to 33093.96) |
| Norway | 24362.45 | 34786.05 | 15573.65 | 24362.45 (15573.65 to 34786.05) |
| New Zealand | 24493.82 | 35801.1 | 15692.04 | 24493.82 (15692.04 to 35801.1) |
| Malawi | 24657.28 | 37563.65 | 15092.67 | 24657.28 (15092.67 to 37563.65) |
| Switzerland | 24730.77 | 36599.96 | 15888.78 | 24730.77 (15888.78 to 36599.96) |
| El Salvador | 26018.16 | 39409.44 | 16038.68 | 26018.16 (16038.68 to 39409.44) |
| Malaysia | 26409.4 | 40017.99 | 16637.74 | 26409.4 (16637.74 to 40017.99) |
| Austria | 26771.15 | 39212.99 | 16962.47 | 26771.15 (16962.47 to 39212.99) |
| Somalia | 27567.15 | 41493.51 | 17376.73 | 27567.15 (17376.73 to 41493.51) |
| Morocco | 27597.03 | 40629.78 | 17448.88 | 27597.03 (17448.88 to 40629.78) |
| Sri Lanka | 27948.67 | 40717.02 | 18240.44 | 27948.67 (18240.44 to 40717.02) |
| Greece | 29711.82 | 43648.74 | 18413.14 | 29711.82 (18413.14 to 43648.74) |
| Guatemala | 29769.6 | 45424.79 | 17918.32 | 29769.6 (17918.32 to 45424.79) |
| Afghanistan | 30907.05 | 45222.85 | 19842 | 30907.05 (19842 to 45222.85) |
| Belarus | 32169.39 | 46236.49 | 20456.12 | 32169.39 (20456.12 to 46236.49) |
| Mozambique | 32825.98 | 50026.22 | 20239.52 | 32825.98 (20239.52 to 50026.22) |
| Ecuador | 33380.09 | 50371.79 | 19586.3 | 33380.09 (19586.3 to 50371.79) |
| Chile | 33627.25 | 48486.54 | 21908.48 | 33627.25 (21908.48 to 48486.54) |
| Yemen | 34242.76 | 51165.34 | 21496.86 | 34242.76 (21496.86 to 51165.34) |
| Cuba | 34887.58 | 52656.79 | 21533.72 | 34887.58 (21533.72 to 52656.79) |
| Iraq | 35314.35 | 53024.23 | 22650.56 | 35314.35 (22650.56 to 53024.23) |
| Algeria | 36326.41 | 55691.89 | 22807.56 | 36326.41 (22807.56 to 55691.89) |
| Sudan | 36571.39 | 55494.41 | 22288.22 | 36571.39 (22288.22 to 55494.41) |
| Uzbekistan | 36697.31 | 54537.97 | 23456.6 | 36697.31 (23456.6 to 54537.97) |
| Belgium | 37559.81 | 55586.49 | 23842.67 | 37559.81 (23842.67 to 55586.49) |
| Kenya | 39161.08 | 60877.42 | 24504.92 | 39161.08 (24504.92 to 60877.42) |
| Democratic People's Republic of Korea | 42496.93 | 63714.08 | 27214.64 | 42496.93 (27214.64 to 63714.08) |
| Taiwan (Province of China) | 42704.36 | 63036.52 | 26584.31 | 42704.36 (26584.31 to 63036.52) |
| Haiti | 44761.1 | 66772.26 | 28403.76 | 44761.1 (28403.76 to 66772.26) |
| Myanmar | 46676.29 | 69790.27 | 29455.94 | 46676.29 (29455.94 to 69790.27) |
| Rwanda | 48033.44 | 73039.56 | 30537.34 | 48033.44 (30537.34 to 73039.56) |
| Sweden | 51901.83 | 74317.4 | 32584.33 | 51901.83 (32584.33 to 74317.4) |
| South Africa | 51944.39 | 78575.85 | 33155.69 | 51944.39 (33155.69 to 78575.85) |
| Romania | 56089.88 | 82541.9 | 35483.87 | 56089.88 (35483.87 to 82541.9) |
| Venezuela (Bolivarian Republic of) | 57865.14 | 88667.02 | 35492.67 | 57865.14 (35492.67 to 88667.02) |
| Uganda | 58288.56 | 88597.36 | 35780.61 | 58288.56 (35780.61 to 88597.36) |
| Netherlands | 61709.26 | 91516.39 | 38498.42 | 61709.26 (38498.42 to 91516.39) |
| Portugal | 61773.44 | 93409.09 | 38097.38 | 61773.44 (38097.38 to 93409.09) |
| Democratic Republic of the Congo | 61796.83 | 93530.14 | 38896.78 | 61796.83 (38896.78 to 93530.14) |
| Republic of Korea | 68278.5 | 98137.95 | 43508.8 | 68278.5 (43508.8 to 98137.95) |
| Madagascar | 69346.52 | 102873.16 | 44198.25 | 69346.52 (44198.25 to 102873.16) |
| Australia | 77804.1 | 111366.78 | 50569.88 | 77804.1 (50569.88 to 111366.78) |
| Colombia | 79408.31 | 121496.5 | 49509.91 | 79408.31 (49509.91 to 121496.5) |
| Canada | 81473.17 | 121073.13 | 52000.18 | 81473.17 (52000.18 to 121073.13) |
| United Republic of Tanzania | 84016.81 | 129582.9 | 49966.15 | 84016.81 (49966.15 to 129582.9) |
| Peru | 84994.88 | 128018.93 | 52085.73 | 84994.88 (52085.73 to 128018.93) |
| Spain | 90703.33 | 135382.73 | 57330.25 | 90703.33 (57330.25 to 135382.73) |
| Thailand | 93773.14 | 139152.26 | 59295.42 | 93773.14 (59295.42 to 139152.26) |
| Argentina | 97231.86 | 141241.82 | 62725.87 | 97231.86 (62725.87 to 141241.82) |
| Viet Nam | 100021.32 | 151887.62 | 63713.41 | 100021.32 (63713.41 to 151887.62) |
| Ukraine | 101844.45 | 149528.71 | 64314.58 | 101844.45 (64314.58 to 149528.71) |
| Ethiopia | 104393.35 | 157402.9 | 66150.06 | 104393.35 (66150.06 to 157402.9) |
| Egypt | 106837.92 | 159973.16 | 65812.81 | 106837.92 (65812.81 to 159973.16) |
| Iran (Islamic Republic of) | 109393.95 | 165105.86 | 69259.68 | 109393.95 (69259.68 to 165105.86) |
| Pakistan | 110073.1 | 166040.65 | 69714.1 | 110073.1 (69714.1 to 166040.65) |
| Bangladesh | 144233.69 | 215451.94 | 89219.95 | 144233.69 (89219.95 to 215451.94) |
| Mexico | 170678.05 | 258379.32 | 105992.98 | 170678.05 (105992.98 to 258379.32) |
| Turkiye | 173261.29 | 252751.61 | 108205.05 | 173261.29 (108205.05 to 252751.61) |
| Italy | 182926.6 | 266426.66 | 117959.93 | 182926.6 (117959.93 to 266426.66) |
| Philippines | 185093.19 | 276760.53 | 118807.33 | 185093.19 (118807.33 to 276760.53) |
| Poland | 207752.9 | 302132.01 | 133335.46 | 207752.9 (133335.46 to 302132.01) |
| Nigeria | 216780.82 | 327640.05 | 138541.56 | 216780.82 (138541.56 to 327640.05) |
| France | 250729.51 | 370609.42 | 161647.27 | 250729.51 (161647.27 to 370609.42) |
| Germany | 298438.74 | 440416.97 | 188724.67 | 298438.74 (188724.67 to 440416.97) |
| Indonesia | 311703.66 | 467860.58 | 198881.75 | 311703.66 (198881.75 to 467860.58) |
| Russian Federation | 343246.85 | 492718.79 | 219616.97 | 343246.85 (219616.97 to 492718.79) |
| United Kingdom | 384890.16 | 546421.31 | 248243.77 | 384890.16 (248243.77 to 546421.31) |
| Japan | 494474.01 | 720233.63 | 316295.11 | 494474.01 (316295.11 to 720233.63) |
| Brazil | 517903.58 | 790276.26 | 317422.44 | 517903.58 (317422.44 to 790276.26) |
| United States of America | 943688.11 | 1393828.52 | 609933.61 | 943688.11 (609933.61 to 1393828.52) |
| China | 1332012.07 | 2013102.35 | 840069.92 | 1332012.07 (840069.92 to 2013102.35) |
| India | 1380306.77 | 2161546.35 | 880424.9 | 1380306.77 (880424.9 to 2161546.35) |

Supplementary Table 6: The number of global asthma YLDs cases in 2021

| **location** | **val** | **upper** | **lower** | **Number_2021** |
| --- | --- | --- | --- | --- |
| Tokelau | 1.02 | 1.52 | 0.65 | 1.02 (0.65 to 1.52) |
| Niue | 1.05 | 1.57 | 0.68 | 1.05 (0.68 to 1.57) |
| Nauru | 8.73 | 12.86 | 5.67 | 8.73 (5.67 to 12.86) |
| Tuvalu | 9.43 | 14.02 | 6.06 | 9.43 (6.06 to 14.02) |
| Cook Islands | 11.43 | 17.5 | 7.21 | 11.43 (7.21 to 17.5) |
| Palau | 11.45 | 17.02 | 7.23 | 11.45 (7.23 to 17.02) |
| Northern Mariana Islands | 28.05 | 42.63 | 17.41 | 28.05 (17.41 to 42.63) |
| American Samoa | 30.61 | 46.36 | 19.15 | 30.61 (19.15 to 46.36) |
| Marshall Islands | 45.98 | 68.85 | 29.8 | 45.98 (29.8 to 68.85) |
| San Marino | 76.55 | 112.56 | 48.52 | 76.55 (48.52 to 112.56) |
| Seychelles | 77.84 | 118.45 | 49.22 | 77.84 (49.22 to 118.45) |
| Micronesia (Federated States of) | 85.64 | 127.58 | 54.84 | 85.64 (54.84 to 127.58) |
| Monaco | 88.24 | 129.61 | 56.13 | 88.24 (56.13 to 129.61) |
| Guam | 90.09 | 137.47 | 56.04 | 90.09 (56.04 to 137.47) |
| Saint Kitts and Nevis | 101.56 | 151.53 | 62.61 | 101.56 (62.61 to 151.53) |
| Bermuda | 108.15 | 161.82 | 66.81 | 108.15 (66.81 to 161.82) |
| Tonga | 138.2 | 208.34 | 86.99 | 138.2 (86.99 to 208.34) |
| Dominica | 152.03 | 228.52 | 97.14 | 152.03 (97.14 to 228.52) |
| Kiribati | 157.95 | 225.38 | 104.28 | 157.95 (104.28 to 225.38) |
| Greenland | 167.87 | 244.89 | 110.31 | 167.87 (110.31 to 244.89) |
| Antigua and Barbuda | 168.21 | 253.14 | 104.55 | 168.21 (104.55 to 253.14) |
| Samoa | 174.75 | 254.77 | 107.81 | 174.75 (107.81 to 254.77) |
| United States Virgin Islands | 177.51 | 265.18 | 109.25 | 177.51 (109.25 to 265.18) |
| Andorra | 220.14 | 325.13 | 140.26 | 220.14 (140.26 to 325.13) |
| Saint Vincent and the Grenadines | 225.86 | 340.06 | 142.93 | 225.86 (142.93 to 340.06) |
| Vanuatu | 262.71 | 380.6 | 169.01 | 262.71 (169.01 to 380.6) |
| Maldives | 311.33 | 470.89 | 190.96 | 311.33 (190.96 to 470.89) |
| Grenada | 314.83 | 475.05 | 192.82 | 314.83 (192.82 to 475.05) |
| Sao Tome and Principe | 326.7 | 495.98 | 210.69 | 326.7 (210.69 to 495.98) |
| Saint Lucia | 413.3 | 608.14 | 266.08 | 413.3 (266.08 to 608.14) |
| Bhutan | 444.33 | 674.02 | 275.91 | 444.33 (275.91 to 674.02) |
| Cabo Verde | 466.18 | 717.51 | 291.63 | 466.18 (291.63 to 717.51) |
| Solomon Islands | 580.07 | 850.48 | 369.49 | 580.07 (369.49 to 850.48) |
| Brunei Darussalam | 649.7 | 944.18 | 413.78 | 649.7 (413.78 to 944.18) |
| Bahamas | 784.4 | 1191.51 | 479.93 | 784.4 (479.93 to 1191.51) |
| Montenegro | 788.76 | 1153.79 | 490.2 | 788.76 (490.2 to 1153.79) |
| Barbados | 828.11 | 1203.12 | 522.07 | 828.11 (522.07 to 1203.12) |
| Fiji | 842.65 | 1248.07 | 546.54 | 842.65 (546.54 to 1248.07) |
| Lesotho | 947.18 | 1413.44 | 607.27 | 947.18 (607.27 to 1413.44) |
| Iceland | 983.55 | 1450.83 | 612.56 | 983.55 (612.56 to 1450.83) |
| Belize | 1029.19 | 1536.44 | 635.32 | 1029.19 (635.32 to 1536.44) |
| Suriname | 1113.43 | 1687.91 | 697.68 | 1113.43 (697.68 to 1687.91) |
| Malta | 1198.1 | 1758.98 | 767.37 | 1198.1 (767.37 to 1758.98) |
| Comoros | 1323.38 | 1997.76 | 835.88 | 1323.38 (835.88 to 1997.76) |
| Mauritius | 1324.37 | 1928.55 | 860.2 | 1324.37 (860.2 to 1928.55) |
| Estonia | 1397.21 | 2023.4 | 885.72 | 1397.21 (885.72 to 2023.4) |
| Equatorial Guinea | 1545.12 | 2387.57 | 940.41 | 1545.12 (940.41 to 2387.57) |
| Trinidad and Tobago | 1606.9 | 2392.9 | 988.44 | 1606.9 (988.44 to 2392.9) |
| Bahrain | 1660.7 | 2464.63 | 1046.38 | 1660.7 (1046.38 to 2464.63) |
| Timor-Leste | 1675.48 | 2518.36 | 1063.98 | 1675.48 (1063.98 to 2518.36) |
| Djibouti | 1763.17 | 2708.92 | 1110.97 | 1763.17 (1110.97 to 2708.92) |
| Guyana | 1773.61 | 2678.09 | 1126.47 | 1773.61 (1126.47 to 2678.09) |
| Luxembourg | 1825.99 | 2706.5 | 1165.68 | 1825.99 (1165.68 to 2706.5) |
| Armenia | 1852.7 | 2726.28 | 1122.85 | 1852.7 (1122.85 to 2726.28) |
| Gabon | 1900.98 | 2851.77 | 1189.41 | 1900.98 (1189.41 to 2851.77) |
| Latvia | 2118.63 | 3073.85 | 1367.59 | 2118.63 (1367.59 to 3073.85) |
| Namibia | 2291.56 | 3462.78 | 1458.55 | 2291.56 (1458.55 to 3462.78) |
| Eswatini | 2583.82 | 3872.61 | 1652.52 | 2583.82 (1652.52 to 3872.61) |
| Gambia | 2586.43 | 3991.65 | 1627.1 | 2586.43 (1627.1 to 3991.65) |
| Georgia | 2611.02 | 3916.37 | 1621.99 | 2611.02 (1621.99 to 3916.37) |
| Albania | 2650.22 | 3854.97 | 1647.4 | 2650.22 (1647.4 to 3854.97) |
| Guinea-Bissau | 2671.78 | 4029.81 | 1714.2 | 2671.78 (1714.2 to 4029.81) |
| Lithuania | 2735.93 | 3968.89 | 1721.94 | 2735.93 (1721.94 to 3968.89) |
| Mongolia | 2829 | 4207.88 | 1789.1 | 2829 (1789.1 to 4207.88) |
| Botswana | 3046.02 | 4603.88 | 1948.78 | 3046.02 (1948.78 to 4603.88) |
| Qatar | 3229.49 | 4858.65 | 1964.06 | 3229.49 (1964.06 to 4858.65) |
| Republic of Moldova | 3344.47 | 4856.16 | 2060.43 | 3344.47 (2060.43 to 4856.16) |
| Turkmenistan | 3640.58 | 5502.7 | 2213.88 | 3640.58 (2213.88 to 5502.7) |
| Cyprus | 4164.36 | 6182.43 | 2536.6 | 4164.36 (2536.6 to 6182.43) |
| Slovenia | 4264.13 | 6213.33 | 2731 | 4264.13 (2731 to 6213.33) |
| North Macedonia | 4993.41 | 7212.07 | 3199.7 | 4993.41 (3199.7 to 7212.07) |
| Liberia | 5314.8 | 8109.57 | 3336.59 | 5314.8 (3336.59 to 8109.57) |
| Kuwait | 5567.74 | 8186.82 | 3439.87 | 5567.74 (3439.87 to 8186.82) |
| Lao People's Democratic Republic | 5752.91 | 8508.71 | 3641.76 | 5752.91 (3641.76 to 8508.71) |
| Slovakia | 5864.91 | 8519.88 | 3647.17 | 5864.91 (3647.17 to 8519.88) |
| Jamaica | 5902.96 | 8656.63 | 3671.79 | 5902.96 (3671.79 to 8656.63) |
| Bosnia and Herzegovina | 6267.19 | 8900.37 | 3990.66 | 6267.19 (3990.66 to 8900.37) |
| Oman | 6500.69 | 9593.91 | 3936.69 | 6500.69 (3936.69 to 9593.91) |
| Croatia | 6588.88 | 9408.25 | 4147.01 | 6588.88 (4147.01 to 9408.25) |
| Singapore | 7023.74 | 10126.01 | 4448.3 | 7023.74 (4448.3 to 10126.01) |
| Congo | 7049.82 | 10593.06 | 4452.56 | 7049.82 (4452.56 to 10593.06) |
| Kyrgyzstan | 7067.73 | 10705.57 | 4388.82 | 7067.73 (4388.82 to 10705.57) |
| Uruguay | 7166.43 | 10493.56 | 4606.43 | 7166.43 (4606.43 to 10493.56) |
| Palestine | 7356.45 | 10973.36 | 4471.45 | 7356.45 (4471.45 to 10973.36) |
| Panama | 7745.83 | 11806.88 | 4705.14 | 7745.83 (4705.14 to 11806.88) |
| Tajikistan | 8175.86 | 12298.54 | 5071.47 | 8175.86 (5071.47 to 12298.54) |
| Libya | 8235.23 | 11938.29 | 5149.23 | 8235.23 (5149.23 to 11938.29) |
| Bulgaria | 8530.27 | 12342.85 | 5397.63 | 8530.27 (5397.63 to 12342.85) |
| Mauritania | 8742.93 | 13405.46 | 5486.16 | 8742.93 (5486.16 to 13405.46) |
| Azerbaijan | 8826.57 | 13133.95 | 5665.19 | 8826.57 (5665.19 to 13133.95) |
| Central African Republic | 8851.38 | 13353.58 | 5661.04 | 8851.38 (5661.04 to 13353.58) |
| Lebanon | 9084.65 | 13292.8 | 5707.54 | 9084.65 (5707.54 to 13292.8) |
| Puerto Rico | 9652.65 | 14280.84 | 6141.39 | 9652.65 (6141.39 to 14280.84) |
| Denmark | 10171.22 | 14628.07 | 6523.69 | 10171.22 (6523.69 to 14628.07) |
| Costa Rica | 10511.88 | 15546.9 | 6527.68 | 10511.88 (6527.68 to 15546.9) |
| Serbia | 10790.49 | 15624.49 | 6808.44 | 10790.49 (6808.44 to 15624.49) |
| Nicaragua | 10836.07 | 16597.48 | 6546.67 | 10836.07 (6546.67 to 16597.48) |
| Czechia | 11031.63 | 15918.71 | 6872.23 | 11031.63 (6872.23 to 15918.71) |
| Hungary | 11071.88 | 16027.01 | 6917.23 | 11071.88 (6917.23 to 16027.01) |
| Papua New Guinea | 11522.15 | 16689.72 | 7351.32 | 11522.15 (7351.32 to 16689.72) |
| Kazakhstan | 11686.64 | 17245.12 | 7351.31 | 11686.64 (7351.31 to 17245.12) |
| Togo | 11723.91 | 17941.04 | 7194.59 | 11723.91 (7194.59 to 17941.04) |
| Eritrea | 11855.2 | 17442.1 | 7545.35 | 11855.2 (7545.35 to 17442.1) |
| Sierra Leone | 12076.53 | 18323.19 | 7734.63 | 12076.53 (7734.63 to 18323.19) |
| Norway | 12553.27 | 18336.23 | 8015.94 | 12553.27 (8015.94 to 18336.23) |
| Zimbabwe | 12914.56 | 19932.21 | 8273.39 | 12914.56 (8273.39 to 19932.21) |
| Ireland | 13117.29 | 19022.77 | 8172.73 | 13117.29 (8172.73 to 19022.77) |
| Cambodia | 13859.41 | 21018 | 8773.78 | 13859.41 (8773.78 to 21018) |
| Finland | 14031.04 | 20109.1 | 9092.16 | 14031.04 (9092.16 to 20109.1) |
| Honduras | 14117.59 | 21496.11 | 8528.73 | 14117.59 (8528.73 to 21496.11) |
| New Zealand | 14205.27 | 21374.65 | 9164.13 | 14205.27 (9164.13 to 21374.65) |
| Israel | 14223.59 | 20764.45 | 8744.05 | 14223.59 (8744.05 to 20764.45) |
| Senegal | 14224.92 | 21458.12 | 8852.69 | 14224.92 (8852.69 to 21458.12) |
| Tunisia | 14524.79 | 20957.17 | 9230.22 | 14524.79 (9230.22 to 20957.17) |
| Paraguay | 14974.64 | 22878.2 | 9036.03 | 14974.64 (9036.03 to 22878.2) |
| El Salvador | 15159.21 | 22464.46 | 9168.34 | 15159.21 (9168.34 to 22464.46) |
| Dominican Republic | 15251.45 | 23160.46 | 9614.53 | 15251.45 (9614.53 to 23160.46) |
| Belarus | 15590.35 | 22319.1 | 10072.39 | 15590.35 (10072.39 to 22319.1) |
| Bolivia (Plurinational State of) | 15900.66 | 24414.41 | 9813.53 | 15900.66 (9813.53 to 24414.41) |
| Jordan | 15956.3 | 23674.45 | 9862.75 | 15956.3 (9862.75 to 23674.45) |
| Austria | 16557.23 | 24262.21 | 10481.72 | 16557.23 (10481.72 to 24262.21) |
| Benin | 17245.39 | 26370.41 | 10885.4 | 17245.39 (10885.4 to 26370.41) |
| Zambia | 17636.47 | 26940.6 | 10894.22 | 17636.47 (10894.22 to 26940.6) |
| Guatemala | 18014.73 | 27220.89 | 11075.13 | 18014.73 (11075.13 to 27220.89) |
| Greece | 18762.22 | 27150.07 | 11851.46 | 18762.22 (11851.46 to 27150.07) |
| Nepal | 18805.51 | 28538.81 | 12028.51 | 18805.51 (12028.51 to 28538.81) |
| Switzerland | 19419.77 | 28423.29 | 12195.06 | 19419.77 (12195.06 to 28423.29) |
| South Sudan | 19546.19 | 29216.9 | 12246.39 | 19546.19 (12246.39 to 29216.9) |
| Chad | 19592.7 | 30191.99 | 12383.31 | 19592.7 (12383.31 to 30191.99) |
| Guinea | 19821.78 | 30396.19 | 12342.37 | 19821.78 (12342.37 to 30396.19) |
| Belgium | 20142.49 | 29015.25 | 12884.93 | 20142.49 (12884.93 to 29015.25) |
| Mali | 21280.66 | 32030.22 | 13176.84 | 21280.66 (13176.84 to 32030.22) |
| Syrian Arab Republic | 21933.56 | 32212.7 | 14119.5 | 21933.56 (14119.5 to 32212.7) |
| Cameroon | 22533.79 | 35256.88 | 13641.05 | 22533.79 (13641.05 to 35256.88) |
| Ecuador | 24151.55 | 37319.23 | 14303.27 | 24151.55 (14303.27 to 37319.23) |
| Cuba | 25366.91 | 37972.55 | 15766.07 | 25366.91 (15766.07 to 37972.55) |
| Ghana | 27790.44 | 42430.57 | 17737.05 | 27790.44 (17737.05 to 42430.57) |
| Burundi | 28960.69 | 42922.43 | 18659.71 | 28960.69 (18659.71 to 42922.43) |
| Malaysia | 29624.71 | 44964.76 | 18683.17 | 29624.71 (18683.17 to 44964.76) |
| Sweden | 30630.97 | 44834.37 | 19820.03 | 30630.97 (19820.03 to 44834.37) |
| Sri Lanka | 30786.47 | 43834.4 | 19901.95 | 30786.47 (19901.95 to 43834.4) |
| Romania | 30833.76 | 44656.06 | 19606.52 | 30833.76 (19606.52 to 44656.06) |
| United Arab Emirates | 32047.84 | 47236.69 | 20317.08 | 32047.84 (20317.08 to 47236.69) |
| Morocco | 34219.27 | 49817.74 | 21854.21 | 34219.27 (21854.21 to 49817.74) |
| Malawi | 34556.5 | 52639.21 | 21539.51 | 34556.5 (21539.51 to 52639.21) |
| South Africa | 36058.7 | 53745.15 | 22981 | 36058.7 (22981 to 53745.15) |
| Saudi Arabia | 36811.22 | 56613.57 | 23422.43 | 36811.22 (23422.43 to 56613.57) |
| Burkina Faso | 37099.02 | 56307.06 | 23109.22 | 37099.02 (23109.22 to 56307.06) |
| Cote d'Ivoire | 37547.77 | 57575.55 | 23699.26 | 37547.77 (23699.26 to 57575.55) |
| Angola | 38318.48 | 58066.28 | 23675.91 | 38318.48 (23675.91 to 58066.28) |
| Myanmar | 38760.94 | 56550.37 | 24840.4 | 38760.94 (24840.4 to 56550.37) |
| Niger | 39351.12 | 59003.69 | 25285.35 | 39351.12 (25285.35 to 59003.69) |
| Ukraine | 40512.74 | 60326.04 | 25271.35 | 40512.74 (25271.35 to 60326.04) |
| Venezuela (Bolivarian Republic of) | 41202.51 | 63141.29 | 24946.44 | 41202.51 (24946.44 to 63141.29) |
| Portugal | 41598.87 | 60721.72 | 26800.88 | 41598.87 (26800.88 to 60721.72) |
| Democratic People's Republic of Korea | 41809.75 | 60857.56 | 27000.22 | 41809.75 (27000.22 to 60857.56) |
| Taiwan (Province of China) | 42369.39 | 63162.01 | 27494.81 | 42369.39 (27494.81 to 63162.01) |
| Sudan | 44122.7 | 67953.36 | 27560.6 | 44122.7 (27560.6 to 67953.36) |
| Netherlands | 45030.62 | 65695.5 | 28417.35 | 45030.62 (28417.35 to 65695.5) |
| Chile | 47413.1 | 68806.76 | 30320.96 | 47413.1 (30320.96 to 68806.76) |
| Yemen | 48102.58 | 71623.4 | 30173.81 | 48102.58 (30173.81 to 71623.4) |
| Uzbekistan | 48271.49 | 71162.36 | 31220.73 | 48271.49 (31220.73 to 71162.36) |
| Afghanistan | 49558.54 | 74336.18 | 31788.49 | 49558.54 (31788.49 to 74336.18) |
| Mozambique | 50628.97 | 75796.55 | 31757.55 | 50628.97 (31757.55 to 75796.55) |
| Iraq | 51112.17 | 74842.29 | 31107.04 | 51112.17 (31107.04 to 74842.29) |
| Algeria | 51344.93 | 75589 | 31741.71 | 51344.93 (31741.71 to 75589) |
| Somalia | 51353.34 | 75619.1 | 33047.64 | 51353.34 (33047.64 to 75619.1) |
| Rwanda | 51500.51 | 78146.28 | 32491.97 | 51500.51 (32491.97 to 78146.28) |
| Kenya | 52906.81 | 80873.4 | 33331.05 | 52906.81 (33331.05 to 80873.4) |
| Republic of Korea | 57402.14 | 82535.83 | 36419.39 | 57402.14 (36419.39 to 82535.83) |
| Colombia | 61601.54 | 91880.54 | 37358.08 | 61601.54 (37358.08 to 91880.54) |
| Haiti | 63638.12 | 92594.68 | 40641.77 | 63638.12 (40641.77 to 92594.68) |
| Peru | 64645.55 | 101452.16 | 38657.41 | 64645.55 (38657.41 to 101452.16) |
| Australia | 73759.7 | 109702.45 | 47643.2 | 73759.7 (47643.2 to 109702.45) |
| Thailand | 76937.91 | 114482.83 | 50198.59 | 76937.91 (50198.59 to 114482.83) |
| Canada | 77177.99 | 112649.67 | 49667.72 | 77177.99 (49667.72 to 112649.67) |
| Spain | 77368.18 | 112464.67 | 49719.16 | 77368.18 (49719.16 to 112464.67) |
| Italy | 85423.07 | 126390.37 | 54216.15 | 85423.07 (54216.15 to 126390.37) |
| Madagascar | 86868.75 | 130951.42 | 56486.71 | 86868.75 (56486.71 to 130951.42) |
| Iran (Islamic Republic of) | 90505.52 | 133243.61 | 57473.88 | 90505.52 (57473.88 to 133243.61) |
| Democratic Republic of the Congo | 100936.58 | 152764.66 | 63398.52 | 100936.58 (63398.52 to 152764.66) |
| Uganda | 105030 | 162294.65 | 64438.54 | 105030 (64438.54 to 162294.65) |
| Argentina | 107316.29 | 158698.84 | 68154.34 | 107316.29 (68154.34 to 158698.84) |
| Ethiopia | 111658.59 | 172243.44 | 69285.18 | 111658.59 (69285.18 to 172243.44) |
| Bangladesh | 112528.29 | 171382.67 | 70754.7 | 112528.29 (70754.7 to 171382.67) |
| Viet Nam | 114692.79 | 174652.43 | 71861.73 | 114692.79 (71861.73 to 174652.43) |
| Mexico | 116898.77 | 177614.09 | 70993.96 | 116898.77 (70993.96 to 177614.09) |
| Russian Federation | 124861.03 | 184457.09 | 78599.47 | 124861.03 (78599.47 to 184457.09) |
| Egypt | 127473.58 | 188979.24 | 78943.94 | 127473.58 (78943.94 to 188979.24) |
| Poland | 130003.73 | 190059.68 | 84509.29 | 130003.73 (84509.29 to 190059.68) |
| Germany | 137320.22 | 196851.05 | 87445.99 | 137320.22 (87445.99 to 196851.05) |
| Pakistan | 141253.57 | 217157.9 | 88766.85 | 141253.57 (88766.85 to 217157.9) |
| Turkiye | 154329.91 | 228728.54 | 96714.08 | 154329.91 (96714.08 to 228728.54) |
| France | 162925.16 | 235186.46 | 104737.42 | 162925.16 (104737.42 to 235186.46) |
| Japan | 165549.58 | 243193.86 | 105143.04 | 165549.58 (105143.04 to 243193.86) |
| United Republic of Tanzania | 167138.13 | 260431.81 | 102442.42 | 167138.13 (102442.42 to 260431.81) |
| Philippines | 205578.53 | 303906.23 | 131121.43 | 205578.53 (131121.43 to 303906.23) |
| Indonesia | 249930.79 | 374128.68 | 158639.04 | 249930.79 (158639.04 to 374128.68) |
| United Kingdom | 259804.31 | 374479.81 | 166991.21 | 259804.31 (166991.21 to 374479.81) |
| Brazil | 363162.04 | 554864.91 | 224756.04 | 363162.04 (224756.04 to 554864.91) |
| Nigeria | 440241.66 | 677936.78 | 281248.36 | 440241.66 (281248.36 to 677936.78) |
| China | 985793.83 | 1477413.01 | 618691.5 | 985793.83 (618691.5 to 1477413.01) |
| India | 1236376.56 | 1838910.13 | 793887.92 | 1236376.56 (793887.92 to 1838910.13) |
| United States of America | 1282331.24 | 1884585.5 | 837258.83 | 1282331.24 (837258.83 to 1884585.5) |

Supplementary Table 7: Global ASPR for asthma in 1990

| **location** | **val** | **upper** | **lower** | **Rate_1990** |
| --- | --- | --- | --- | --- |
| Lesotho | 1664.63 | 1891.61 | 1481.95 | 1664.63 (1481.95 to 1891.61) |
| Kazakhstan | 1761.98 | 2067.05 | 1509.96 | 1761.98 (1509.96 to 2067.05) |
| Armenia | 2088.04 | 2546.19 | 1733.21 | 2088.04 (1733.21 to 2546.19) |
| Cameroon | 2190.88 | 2559.87 | 1862.09 | 2190.88 (1862.09 to 2559.87) |
| Nepal | 2289.53 | 2595.33 | 2041.62 | 2289.53 (2041.62 to 2595.33) |
| Zimbabwe | 2362.19 | 2761.69 | 2040.82 | 2362.19 (2040.82 to 2761.69) |
| Ghana | 2520.45 | 2860.52 | 2199.1 | 2520.45 (2199.1 to 2860.52) |
| Seychelles | 2596.25 | 2964.76 | 2259.76 | 2596.25 (2259.76 to 2964.76) |
| Mali | 2605.79 | 2973.38 | 2273.98 | 2605.79 (2273.98 to 2973.38) |
| Georgia | 2612.98 | 3061.36 | 2248.54 | 2612.98 (2248.54 to 3061.36) |
| American Samoa | 2653.05 | 3029.36 | 2339.97 | 2653.05 (2339.97 to 3029.36) |
| Northern Mariana Islands | 2656.54 | 3151.85 | 2252.34 | 2656.54 (2252.34 to 3151.85) |
| Cook Islands | 2657 | 3101.17 | 2314.01 | 2657 (2314.01 to 3101.17) |
| Pakistan | 2674.28 | 3054.49 | 2325.26 | 2674.28 (2325.26 to 3054.49) |
| Zambia | 2701.77 | 3149.9 | 2293.1 | 2701.77 (2293.1 to 3149.9) |
| Bhutan | 2721.25 | 3072.47 | 2399.62 | 2721.25 (2399.62 to 3072.47) |
| Chad | 2793.8 | 3205.06 | 2434.92 | 2793.8 (2434.92 to 3205.06) |
| Saudi Arabia | 2826.72 | 3228.11 | 2476.49 | 2826.72 (2476.49 to 3228.11) |
| Morocco | 2832.01 | 3178.34 | 2502.75 | 2832.01 (2502.75 to 3178.34) |
| Niue | 2910.06 | 3260.6 | 2590.09 | 2910.06 (2590.09 to 3260.6) |
| Senegal | 2955.35 | 3387.05 | 2569.54 | 2955.35 (2569.54 to 3387.05) |
| Guam | 2965.43 | 3623.74 | 2419.02 | 2965.43 (2419.02 to 3623.74) |
| Tajikistan | 2972.69 | 3411.52 | 2589.28 | 2972.69 (2589.28 to 3411.52) |
| Myanmar | 3009.41 | 3338.74 | 2711.21 | 3009.41 (2711.21 to 3338.74) |
| Azerbaijan | 3022.13 | 3461.36 | 2652.38 | 3022.13 (2652.38 to 3461.36) |
| China | 3087.89 | 3782.05 | 2583.55 | 3087.89 (2583.55 to 3782.05) |
| Albania | 3152.73 | 3672.8 | 2719.9 | 3152.73 (2719.9 to 3672.8) |
| Samoa | 3169 | 3647.6 | 2728.76 | 3169 (2728.76 to 3647.6) |
| Mongolia | 3177.3 | 3612.26 | 2822.43 | 3177.3 (2822.43 to 3612.26) |
| Cambodia | 3179.24 | 3543.38 | 2873.65 | 3179.24 (2873.65 to 3543.38) |
| Liberia | 3296.74 | 3819.99 | 2841.5 | 3296.74 (2841.5 to 3819.99) |
| Palau | 3319.32 | 3804.75 | 2906.04 | 3319.32 (2906.04 to 3804.75) |
| Namibia | 3406.2 | 3844.15 | 3014.18 | 3406.2 (3014.18 to 3844.15) |
| South Africa | 3407.5 | 3951.48 | 2940.09 | 3407.5 (2940.09 to 3951.48) |
| Bangladesh | 3421.43 | 3824.67 | 3054.23 | 3421.43 (3054.23 to 3824.67) |
| Gambia | 3434.61 | 3895.23 | 3024.18 | 3434.61 (3024.18 to 3895.23) |
| Slovakia | 3469.35 | 4148.8 | 2899.92 | 3469.35 (2899.92 to 4148.8) |
| Oman | 3488.97 | 4175.92 | 2925.64 | 3488.97 (2925.64 to 4175.92) |
| Tokelau | 3583.65 | 3959.23 | 3239.78 | 3583.65 (3239.78 to 3959.23) |
| Czechia | 3587.16 | 4303.92 | 2993.7 | 3587.16 (2993.7 to 4303.92) |
| Cabo Verde | 3592.23 | 4119.36 | 3147.73 | 3592.23 (3147.73 to 4119.36) |
| Lao People's Democratic Republic | 3613.58 | 4055.96 | 3189.52 | 3613.58 (3189.52 to 4055.96) |
| Solomon Islands | 3653.34 | 4009.55 | 3348.45 | 3653.34 (3348.45 to 4009.55) |
| Kenya | 3654.63 | 4299.87 | 3160.6 | 3654.63 (3160.6 to 4299.87) |
| Lithuania | 3664.53 | 4314.64 | 3149.98 | 3664.53 (3149.98 to 4314.64) |
| Marshall Islands | 3666.1 | 3991.38 | 3357.99 | 3666.1 (3357.99 to 3991.38) |
| Democratic Republic of the Congo | 3681.51 | 4245.08 | 3192.98 | 3681.51 (3192.98 to 4245.08) |
| Algeria | 3737.2 | 4272.99 | 3207.68 | 3737.2 (3207.68 to 4272.99) |
| Viet Nam | 3764.39 | 4328.06 | 3259.05 | 3764.39 (3259.05 to 4328.06) |
| Maldives | 3783.67 | 4242.62 | 3369.06 | 3783.67 (3369.06 to 4242.62) |
| Montenegro | 3785.45 | 4661.4 | 3070.7 | 3785.45 (3070.7 to 4661.4) |
| Nauru | 3809.91 | 4193.78 | 3468.13 | 3809.91 (3468.13 to 4193.78) |
| Vanuatu | 3919.74 | 4265.24 | 3576.6 | 3919.74 (3576.6 to 4265.24) |
| Estonia | 3935.67 | 4471.78 | 3453.75 | 3935.67 (3453.75 to 4471.78) |
| Gabon | 3944.91 | 4543.79 | 3368.54 | 3944.91 (3368.54 to 4543.79) |
| Cote d'Ivoire | 3999.16 | 4564.79 | 3506.82 | 3999.16 (3506.82 to 4564.79) |
| Burkina Faso | 3999.25 | 4645.1 | 3485.37 | 3999.25 (3485.37 to 4645.1) |
| Togo | 4016.55 | 4740 | 3353.01 | 4016.55 (3353.01 to 4740) |
| Qatar | 4068.43 | 4765.67 | 3439.5 | 4068.43 (3439.5 to 4765.67) |
| Tunisia | 4075.71 | 4605.15 | 3592.66 | 4075.71 (3592.66 to 4605.15) |
| Sierra Leone | 4089.96 | 4638.31 | 3580.94 | 4089.96 (3580.94 to 4638.31) |
| Benin | 4100.75 | 4612.23 | 3673.91 | 4100.75 (3673.91 to 4612.23) |
| Kyrgyzstan | 4105.69 | 4759.93 | 3531.83 | 4105.69 (3531.83 to 4759.93) |
| Micronesia (Federated States of) | 4159.75 | 4530.07 | 3833.58 | 4159.75 (3833.58 to 4530.07) |
| Malaysia | 4172.27 | 4583.05 | 3765.3 | 4172.27 (3765.3 to 4583.05) |
| Syrian Arab Republic | 4175.43 | 4659.32 | 3730.96 | 4175.43 (3730.96 to 4659.32) |
| Indonesia | 4228.76 | 4877.28 | 3747.81 | 4228.76 (3747.81 to 4877.28) |
| Serbia | 4250.2 | 4943.58 | 3598.27 | 4250.2 (3598.27 to 4943.58) |
| India | 4260.73 | 5037.05 | 3575.78 | 4260.73 (3575.78 to 5037.05) |
| Guinea | 4319.77 | 4966.84 | 3684.57 | 4319.77 (3684.57 to 4966.84) |
| Fiji | 4406.61 | 4866.91 | 3949.49 | 4406.61 (3949.49 to 4866.91) |
| Jordan | 4416.74 | 5151.3 | 3758.78 | 4416.74 (3758.78 to 5151.3) |
| Ethiopia | 4452.03 | 5162.25 | 3868.31 | 4452.03 (3868.31 to 5162.25) |
| Botswana | 4465.91 | 5098.23 | 3935.4 | 4465.91 (3935.4 to 5098.23) |
| Trinidad and Tobago | 4485.96 | 5311.15 | 3812.07 | 4485.96 (3812.07 to 5311.15) |
| Hungary | 4492.21 | 5297.2 | 3847.07 | 4492.21 (3847.07 to 5297.2) |
| Libya | 4503.71 | 5132.87 | 3967.39 | 4503.71 (3967.39 to 5132.87) |
| Republic of Moldova | 4510.13 | 5224.39 | 3912.24 | 4510.13 (3912.24 to 5224.39) |
| Sudan | 4518.22 | 5182.87 | 3903.87 | 4518.22 (3903.87 to 5182.87) |
| Turkmenistan | 4547.9 | 5110.16 | 4011.49 | 4547.9 (4011.49 to 5110.16) |
| Tonga | 4557.12 | 5309.67 | 3890.83 | 4557.12 (3890.83 to 5309.67) |
| Kuwait | 4557.36 | 5317.82 | 3886.73 | 4557.36 (3886.73 to 5317.82) |
| Mexico | 4580.75 | 5497.32 | 3817.91 | 4580.75 (3817.91 to 5497.32) |
| Tuvalu | 4586.26 | 4992.13 | 4204.16 | 4586.26 (4204.16 to 4992.13) |
| Thailand | 4591.36 | 5144.55 | 4035.11 | 4591.36 (4035.11 to 5144.55) |
| Niger | 4640.3 | 5225.81 | 4121.78 | 4640.3 (4121.78 to 5225.81) |
| Republic of Korea | 4711.7 | 5461.57 | 4109.13 | 4711.7 (4109.13 to 5461.57) |
| Equatorial Guinea | 4746.53 | 5302.71 | 4255.63 | 4746.53 (4255.63 to 5302.71) |
| Palestine | 4770.34 | 5525.02 | 4133.86 | 4770.34 (4133.86 to 5525.02) |
| Sri Lanka | 4791.2 | 5290.77 | 4359.72 | 4791.2 (4359.72 to 5290.77) |
| Egypt | 4793.52 | 5445.99 | 4121.08 | 4793.52 (4121.08 to 5445.99) |
| Angola | 4835.92 | 5405.64 | 4292.78 | 4835.92 (4292.78 to 5405.64) |
| Timor-Leste | 4884.28 | 5471.52 | 4375.03 | 4884.28 (4375.03 to 5471.52) |
| Ukraine | 4929.84 | 5926.65 | 4094.65 | 4929.84 (4094.65 to 5926.65) |
| Bahrain | 4932.66 | 5614.14 | 4378.95 | 4932.66 (4378.95 to 5614.14) |
| Latvia | 4970.15 | 5715.13 | 4299.89 | 4970.15 (4299.89 to 5715.13) |
| Mozambique | 5004.8 | 5735.7 | 4336.67 | 5004.8 (4336.67 to 5735.7) |
| Papua New Guinea | 5017.02 | 5476.87 | 4620.89 | 5017.02 (4620.89 to 5476.87) |
| Iraq | 5032.44 | 5632.93 | 4500.25 | 5032.44 (4500.25 to 5632.93) |
| Iran (Islamic Republic of) | 5033.77 | 5702.39 | 4404.74 | 5033.77 (4404.74 to 5702.39) |
| Guinea-Bissau | 5050.66 | 5563.16 | 4543.12 | 5050.66 (4543.12 to 5563.16) |
| Mauritius | 5116.75 | 5792.5 | 4493.14 | 5116.75 (4493.14 to 5792.5) |
| Congo | 5131.67 | 5824.7 | 4505.83 | 5131.67 (4505.83 to 5824.7) |
| Bulgaria | 5157.26 | 6124.7 | 4380.22 | 5157.26 (4380.22 to 6124.7) |
| Central African Republic | 5189.16 | 5780.65 | 4653.33 | 5189.16 (4653.33 to 5780.65) |
| Dominican Republic | 5265.32 | 6058.71 | 4519.72 | 5265.32 (4519.72 to 6058.71) |
| Djibouti | 5307.57 | 6033.8 | 4636.68 | 5307.57 (4636.68 to 6033.8) |
| Malawi | 5308.26 | 6107.24 | 4524.35 | 5308.26 (4524.35 to 6107.24) |
| Sao Tome and Principe | 5380.06 | 6075.46 | 4730.75 | 5380.06 (4730.75 to 6075.46) |
| Uzbekistan | 5406.93 | 6098.97 | 4729.16 | 5406.93 (4729.16 to 6098.97) |
| Democratic People's Republic of Korea | 5511.75 | 6362.86 | 4776.93 | 5511.75 (4776.93 to 6362.86) |
| Lebanon | 5544.3 | 6366.27 | 4799.64 | 5544.3 (4799.64 to 6366.27) |
| Spain | 5565.85 | 6735.14 | 4360.26 | 5565.85 (4360.26 to 6735.14) |
| Russian Federation | 5569.65 | 6413.96 | 4776.22 | 5569.65 (4776.22 to 6413.96) |
| Paraguay | 5605.99 | 6656.26 | 4746.61 | 5605.99 (4746.61 to 6656.26) |
| Bosnia and Herzegovina | 5647.26 | 6663.02 | 4791.37 | 5647.26 (4791.37 to 6663.02) |
| Colombia | 5806.83 | 6969.02 | 4811.03 | 5806.83 (4811.03 to 6969.02) |
| Suriname | 5863.03 | 6844.06 | 5003.82 | 5863.03 (5003.82 to 6844.06) |
| Taiwan (Province of China) | 5894.05 | 6905.85 | 5004.46 | 5894.05 (5004.46 to 6905.85) |
| Nigeria | 5946.26 | 6888.55 | 5156.94 | 5946.26 (5156.94 to 6888.55) |
| Romania | 5995.09 | 7048.82 | 5031.96 | 5995.09 (5031.96 to 7048.82) |
| Saint Kitts and Nevis | 6013.56 | 7068.65 | 5084.26 | 6013.56 (5084.26 to 7068.65) |
| Comoros | 6069.18 | 6983.17 | 5202.26 | 6069.18 (5202.26 to 6983.17) |
| Brunei Darussalam | 6087.24 | 7041.34 | 5258.69 | 6087.24 (5258.69 to 7041.34) |
| Saint Vincent and the Grenadines | 6101.26 | 7219.67 | 5199.01 | 6101.26 (5199.01 to 7219.67) |
| Kiribati | 6195 | 6681.49 | 5741.61 | 6195 (5741.61 to 6681.49) |
| Croatia | 6230.86 | 7285.82 | 5362.7 | 6230.86 (5362.7 to 7285.82) |
| Bahamas | 6272.09 | 7488.44 | 5237.37 | 6272.09 (5237.37 to 7488.44) |
| Israel | 6284.21 | 7213.95 | 5425.88 | 6284.21 (5425.88 to 7213.95) |
| Bolivia (Plurinational State of) | 6323.82 | 7514.92 | 5224.46 | 6323.82 (5224.46 to 7514.92) |
| Guyana | 6333.97 | 7252.99 | 5501.22 | 6333.97 (5501.22 to 7252.99) |
| Antigua and Barbuda | 6389.93 | 7922.92 | 5224.75 | 6389.93 (5224.75 to 7922.92) |
| South Sudan | 6414.6 | 7348.36 | 5616.16 | 6414.6 (5616.16 to 7348.36) |
| Guatemala | 6439.82 | 7487.3 | 5487.42 | 6439.82 (5487.42 to 7487.3) |
| Honduras | 6530.04 | 7503.87 | 5579.89 | 6530.04 (5579.89 to 7503.87) |
| Venezuela (Bolivarian Republic of) | 6586.32 | 7697.18 | 5460.29 | 6586.32 (5460.29 to 7697.18) |
| Ecuador | 6602.31 | 7949.46 | 5313.04 | 6602.31 (5313.04 to 7949.46) |
| Bermuda | 6676.7 | 8073.15 | 5521.71 | 6676.7 (5521.71 to 8073.15) |
| Greece | 6695.34 | 8019.5 | 5460.34 | 6695.34 (5460.34 to 8019.5) |
| Dominica | 6750.79 | 7860.51 | 5756.65 | 6750.79 (5756.65 to 7860.51) |
| Mauritania | 6764.8 | 7686.57 | 5907.93 | 6764.8 (5907.93 to 7686.57) |
| San Marino | 6767.49 | 8134.14 | 5525.7 | 6767.49 (5525.7 to 8134.14) |
| Belize | 6799.61 | 7828.95 | 5833.13 | 6799.61 (5833.13 to 7828.95) |
| Italy | 6888.88 | 7845.13 | 5928.62 | 6888.88 (5928.62 to 7845.13) |
| Yemen | 6894.06 | 7536.57 | 6272.18 | 6894.06 (6272.18 to 7536.57) |
| United Republic of Tanzania | 6976.83 | 8145.77 | 5780.78 | 6976.83 (5780.78 to 8145.77) |
| Eritrea | 6985.23 | 7766.18 | 6218.37 | 6985.23 (6218.37 to 7766.18) |
| Monaco | 7004.3 | 8512.4 | 5793.11 | 7004.3 (5793.11 to 8512.4) |
| Chile | 7067.48 | 8004.57 | 6211.47 | 7067.48 (6211.47 to 8004.57) |
| Panama | 7076.85 | 8299.63 | 5898.95 | 7076.85 (5898.95 to 8299.63) |
| Uganda | 7088.23 | 8132.7 | 6114.71 | 7088.23 (6114.71 to 8132.7) |
| Nicaragua | 7113.64 | 8336.12 | 5925.69 | 7113.64 (5925.69 to 8336.12) |
| Uruguay | 7162.85 | 8057.76 | 6336.87 | 7162.85 (6336.87 to 8057.76) |
| United States Virgin Islands | 7187.45 | 8653.1 | 5973.67 | 7187.45 (5973.67 to 8653.1) |
| Denmark | 7196.78 | 8241.67 | 6246.86 | 7196.78 (6246.86 to 8241.67) |
| Philippines | 7203.37 | 8256.66 | 6364.11 | 7203.37 (6364.11 to 8256.66) |
| Belarus | 7415.2 | 8648.35 | 6312.37 | 7415.2 (6312.37 to 8648.35) |
| Slovenia | 7514.46 | 8754.67 | 6448.3 | 7514.46 (6448.3 to 8754.67) |
| Argentina | 7527.12 | 8721.22 | 6522.05 | 7527.12 (6522.05 to 8721.22) |
| Somalia | 7587.1 | 8499.58 | 6769.95 | 7587.1 (6769.95 to 8499.58) |
| Singapore | 7645.56 | 8542.57 | 6730.58 | 7645.56 (6730.58 to 8542.57) |
| Finland | 7690.34 | 8899.96 | 6499.98 | 7690.34 (6499.98 to 8899.96) |
| Jamaica | 7730.56 | 8908.62 | 6632.48 | 7730.56 (6632.48 to 8908.62) |
| Canada | 7748.75 | 8969.42 | 6711.47 | 7748.75 (6711.47 to 8969.42) |
| Eswatini | 7818.51 | 8777.72 | 7002.09 | 7818.51 (7002.09 to 8777.72) |
| Turkiye | 7827.51 | 8933.23 | 6923.65 | 7827.51 (6923.65 to 8933.23) |
| Peru | 7886.96 | 9353.21 | 6570.6 | 7886.96 (6570.6 to 9353.21) |
| Brazil | 7942.37 | 9796.94 | 6460.55 | 7942.37 (6460.55 to 9796.94) |
| Barbados | 7967.73 | 9416.28 | 6702.5 | 7967.73 (6702.5 to 9416.28) |
| Austria | 7986.53 | 9298.81 | 6645.58 | 7986.53 (6645.58 to 9298.81) |
| Costa Rica | 8025.49 | 9230.76 | 6889.47 | 8025.49 (6889.47 to 9230.76) |
| Afghanistan | 8146.6 | 8836.95 | 7478.36 | 8146.6 (7478.36 to 8836.95) |
| Cuba | 8347.51 | 9979.51 | 6896.32 | 8347.51 (6896.32 to 9979.51) |
| Saint Lucia | 8348.54 | 9447.68 | 7266.93 | 8348.54 (7266.93 to 9447.68) |
| Burundi | 8435.28 | 9326.72 | 7604.27 | 8435.28 (7604.27 to 9326.72) |
| Germany | 8438.13 | 9955.46 | 6793.87 | 8438.13 (6793.87 to 9955.46) |
| North Macedonia | 8560.08 | 9651.56 | 7548.15 | 8560.08 (7548.15 to 9651.56) |
| Switzerland | 8616.41 | 10096.01 | 7254.83 | 8616.41 (7254.83 to 10096.01) |
| Belgium | 8727.23 | 10206.29 | 7185.35 | 8727.23 (7185.35 to 10206.29) |
| Cyprus | 9282.82 | 11460.72 | 7408.41 | 9282.82 (7408.41 to 11460.72) |
| Japan | 9638.63 | 11237.05 | 8362.66 | 9638.63 (8362.66 to 11237.05) |
| Andorra | 9717.88 | 11492.48 | 8124.37 | 9717.88 (8124.37 to 11492.48) |
| United States of America | 9780.16 | 11819.88 | 8270.46 | 9780.16 (8270.46 to 11819.88) |
| Malta | 9858 | 11472.76 | 8319.79 | 9858 (8319.79 to 11472.76) |
| Netherlands | 9954.39 | 11788.51 | 7959.82 | 9954.39 (7959.82 to 11788.51) |
| Grenada | 10180.78 | 12141.64 | 8373.12 | 10180.78 (8373.12 to 12141.64) |
| France | 10288.2 | 11863.11 | 8920.18 | 10288.2 (8920.18 to 11863.11) |
| El Salvador | 10583.6 | 12746.93 | 8598.54 | 10583.6 (8598.54 to 12746.93) |
| United Arab Emirates | 11050.54 | 12272.09 | 9927.53 | 11050.54 (9927.53 to 12272.09) |
| Luxembourg | 11670.58 | 13856.93 | 9661.87 | 11670.58 (9661.87 to 13856.93) |
| Iceland | 11707.14 | 13605.53 | 9908.07 | 11707.14 (9908.07 to 13605.53) |
| Australia | 11831.99 | 12922.86 | 10885.64 | 11831.99 (10885.64 to 12922.86) |
| Madagascar | 11894.84 | 13092.88 | 10686.7 | 11894.84 (10686.7 to 13092.88) |
| Puerto Rico | 11922.85 | 13947.93 | 10245.46 | 11922.85 (10245.46 to 13947.93) |
| Greenland | 12179.85 | 13616.44 | 10880.66 | 12179.85 (10880.66 to 13616.44) |
| Ireland | 12538.1 | 14383.33 | 10810.38 | 12538.1 (10810.38 to 14383.33) |
| Norway | 12979.94 | 14789.92 | 11287.84 | 12979.94 (11287.84 to 14789.92) |
| Poland | 13239.74 | 14956.48 | 11533.39 | 13239.74 (11533.39 to 14956.48) |
| Sweden | 13432.23 | 15791 | 11212.64 | 13432.23 (11212.64 to 15791) |
| Portugal | 14563.01 | 17765.14 | 11431.6 | 14563.01 (11431.6 to 17765.14) |
| Haiti | 14648.1 | 16213.03 | 13212.44 | 14648.1 (13212.44 to 16213.03) |
| Rwanda | 14673.28 | 16671.06 | 12934.45 | 14673.28 (12934.45 to 16671.06) |
| United Kingdom | 16628.61 | 19048.33 | 14434.36 | 16628.61 (14434.36 to 19048.33) |
| New Zealand | 18179.66 | 21094.02 | 15448.46 | 18179.66 (15448.46 to 21094.02) |

Supplementary Table 8: Global ASPR for asthma in 2021

| **location** | **val** | **upper** | **lower** | **Rate_2021** |
| --- | --- | --- | --- | --- |
| Lesotho | 1326.48 | 1490.36 | 1182.91 | 1326.48 (1182.91 to 1490.36) |
| American Samoa | 1557.22 | 1863.67 | 1325.83 | 1557.22 (1325.83 to 1863.67) |
| Guam | 1577 | 2012.32 | 1271.79 | 1577 (1271.79 to 2012.32) |
| Pakistan | 1597.08 | 1839.2 | 1395.85 | 1597.08 (1395.85 to 1839.2) |
| Kazakhstan | 1599.8 | 1888.63 | 1360.98 | 1599.8 (1360.98 to 1888.63) |
| Northern Mariana Islands | 1601.28 | 2006.59 | 1318.07 | 1601.28 (1318.07 to 2006.59) |
| Niue | 1645.15 | 1919.1 | 1434.28 | 1645.15 (1434.28 to 1919.1) |
| Bhutan | 1652.78 | 1927.98 | 1435.09 | 1652.78 (1435.09 to 1927.98) |
| Cameroon | 1652.81 | 1952.35 | 1387.55 | 1652.81 (1387.55 to 1952.35) |
| South Africa | 1678.76 | 1970.05 | 1452.95 | 1678.76 (1452.95 to 1970.05) |
| Nepal | 1722.92 | 1965.57 | 1530.5 | 1722.92 (1530.5 to 1965.57) |
| Turkmenistan | 1749.99 | 2138.72 | 1442.25 | 1749.99 (1442.25 to 2138.72) |
| Armenia | 1753.85 | 2210 | 1411.32 | 1753.85 (1411.32 to 2210) |
| Palau | 1766.32 | 2100.53 | 1522.41 | 1766.32 (1522.41 to 2100.53) |
| Bangladesh | 1798.28 | 2137.8 | 1538.95 | 1798.28 (1538.95 to 2137.8) |
| Maldives | 1801.66 | 2227.8 | 1507.99 | 1801.66 (1507.99 to 2227.8) |
| Myanmar | 1802.92 | 2039.93 | 1607.84 | 1802.92 (1607.84 to 2039.93) |
| Cook Islands | 1803.83 | 2268.97 | 1475.85 | 1803.83 (1475.85 to 2268.97) |
| Tokelau | 1859.74 | 2183.88 | 1633.24 | 1859.74 (1633.24 to 2183.88) |
| China | 1956.49 | 2491.87 | 1566.68 | 1956.49 (1566.68 to 2491.87) |
| Tuvalu | 1981.87 | 2216.66 | 1773.12 | 1981.87 (1773.12 to 2216.66) |
| Seychelles | 1985.47 | 2338.1 | 1711.86 | 1985.47 (1711.86 to 2338.1) |
| Ghana | 1987.93 | 2301.48 | 1744.31 | 1987.93 (1744.31 to 2301.48) |
| Zambia | 2007.9 | 2331.61 | 1724.32 | 2007.9 (1724.32 to 2331.61) |
| Georgia | 2016.73 | 2434.46 | 1680.68 | 2016.73 (1680.68 to 2434.46) |
| Lao People's Democratic Republic | 2033.3 | 2289.68 | 1808.6 | 2033.3 (1808.6 to 2289.68) |
| Nauru | 2081.66 | 2306.24 | 1894.88 | 2081.66 (1894.88 to 2306.24) |
| Mali | 2138.73 | 2467.18 | 1846.21 | 2138.73 (1846.21 to 2467.18) |
| Cambodia | 2150.44 | 2421.96 | 1917.81 | 2150.44 (1917.81 to 2421.96) |
| Samoa | 2155.54 | 2529.8 | 1850.82 | 2155.54 (1850.82 to 2529.8) |
| Tajikistan | 2170.6 | 2590.1 | 1869.76 | 2170.6 (1869.76 to 2590.1) |
| Marshall Islands | 2170.8 | 2388.11 | 1985.23 | 2170.8 (1985.23 to 2388.11) |
| Senegal | 2212.01 | 2574.32 | 1930.63 | 2212.01 (1930.63 to 2574.32) |
| Cabo Verde | 2214.37 | 2740.46 | 1841.64 | 2214.37 (1841.64 to 2740.46) |
| Micronesia (Federated States of) | 2222.56 | 2469.78 | 2010 | 2222.56 (2010 to 2469.78) |
| Zimbabwe | 2232.32 | 2539.75 | 1980.32 | 2232.32 (1980.32 to 2539.75) |
| Mongolia | 2235.53 | 2625.22 | 1917.93 | 2235.53 (1917.93 to 2625.22) |
| Azerbaijan | 2247.67 | 2633.38 | 1909.25 | 2247.67 (1909.25 to 2633.38) |
| Vanuatu | 2271.98 | 2472.79 | 2085.24 | 2271.98 (2085.24 to 2472.79) |
| Ethiopia | 2317.77 | 2703.37 | 2006.9 | 2317.77 (2006.9 to 2703.37) |
| Liberia | 2324.69 | 2681.11 | 2029.94 | 2324.69 (2029.94 to 2681.11) |
| Chad | 2371.63 | 2710.45 | 2074.95 | 2371.63 (2074.95 to 2710.45) |
| Indonesia | 2384.08 | 2666.38 | 2124.17 | 2384.08 (2124.17 to 2666.38) |
| Mexico | 2390.68 | 3007.39 | 1923.9 | 2390.68 (1923.9 to 3007.39) |
| Solomon Islands | 2402.41 | 2622.8 | 2196 | 2402.41 (2196 to 2622.8) |
| Fiji | 2412.44 | 2688.38 | 2163.83 | 2412.44 (2163.83 to 2688.38) |
| India | 2414.64 | 2767.53 | 2111.34 | 2414.64 (2111.34 to 2767.53) |
| Russian Federation | 2418.87 | 2967.85 | 1960.76 | 2418.87 (1960.76 to 2967.85) |
| Morocco | 2429.48 | 2808.87 | 2110.03 | 2429.48 (2110.03 to 2808.87) |
| Namibia | 2442.26 | 2823.28 | 2138.67 | 2442.26 (2138.67 to 2823.28) |
| Malaysia | 2461.83 | 2897.64 | 2094.09 | 2461.83 (2094.09 to 2897.64) |
| Equatorial Guinea | 2467.85 | 2911.12 | 2114.98 | 2467.85 (2114.98 to 2911.12) |
| Kenya | 2537.61 | 2930.42 | 2216.85 | 2537.61 (2216.85 to 2930.42) |
| Kyrgyzstan | 2572.86 | 3076.02 | 2149.37 | 2572.86 (2149.37 to 3076.02) |
| Gabon | 2601.6 | 3136.64 | 2204.78 | 2601.6 (2204.78 to 3136.64) |
| Sudan | 2606.09 | 3000.89 | 2270.43 | 2606.09 (2270.43 to 3000.89) |
| Angola | 2637.31 | 3007.47 | 2337.6 | 2637.31 (2337.6 to 3007.47) |
| Albania | 2687.17 | 3207.86 | 2243.97 | 2687.17 (2243.97 to 3207.86) |
| Republic of Moldova | 2692.8 | 3279.19 | 2203.88 | 2692.8 (2203.88 to 3279.19) |
| Guatemala | 2712.08 | 3329.68 | 2267.27 | 2712.08 (2267.27 to 3329.68) |
| Gambia | 2736 | 3096.28 | 2414.48 | 2736 (2414.48 to 3096.28) |
| Democratic Republic of the Congo | 2769.46 | 3148.06 | 2450.47 | 2769.46 (2450.47 to 3148.06) |
| Estonia | 2789.26 | 3387.63 | 2330.47 | 2789.26 (2330.47 to 3387.63) |
| Saudi Arabia | 2798.86 | 3332.94 | 2377.25 | 2798.86 (2377.25 to 3332.94) |
| Lithuania | 2819.58 | 3408.32 | 2331.76 | 2819.58 (2331.76 to 3408.32) |
| Iran (Islamic Republic of) | 2861.9 | 3360.58 | 2449.38 | 2861.9 (2449.38 to 3360.58) |
| Mauritius | 2886.61 | 3302.38 | 2479.38 | 2886.61 (2479.38 to 3302.38) |
| Benin | 2901.49 | 3273.11 | 2566.47 | 2901.49 (2566.47 to 3273.11) |
| Ukraine | 2970.93 | 3690.81 | 2345.25 | 2970.93 (2345.25 to 3690.81) |
| Timor-Leste | 2979.79 | 3308.46 | 2705.85 | 2979.79 (2705.85 to 3308.46) |
| Algeria | 3004.73 | 3571.56 | 2563.49 | 3004.73 (2563.49 to 3571.56) |
| Viet Nam | 3011.36 | 3475.18 | 2596.92 | 3011.36 (2596.92 to 3475.18) |
| Czechia | 3043.34 | 3715.82 | 2488.67 | 3043.34 (2488.67 to 3715.82) |
| Republic of Korea | 3044.51 | 3705.27 | 2533.95 | 3044.51 (2533.95 to 3705.27) |
| Slovakia | 3060.79 | 3742.63 | 2533.73 | 3060.79 (2533.73 to 3742.63) |
| Papua New Guinea | 3076.95 | 3318.64 | 2854.29 | 3076.95 (2854.29 to 3318.64) |
| Latvia | 3094.58 | 3753.24 | 2578.23 | 3094.58 (2578.23 to 3753.24) |
| Thailand | 3110.55 | 3640.56 | 2674.14 | 3110.55 (2674.14 to 3640.56) |
| Iraq | 3164.68 | 3673.86 | 2728.84 | 3164.68 (2728.84 to 3673.86) |
| Egypt | 3171.16 | 3646.71 | 2751.34 | 3171.16 (2751.34 to 3646.71) |
| Bahrain | 3198.46 | 3750.24 | 2707.81 | 3198.46 (2707.81 to 3750.24) |
| Sierra Leone | 3199.15 | 3592.96 | 2856.06 | 3199.15 (2856.06 to 3592.96) |
| Tunisia | 3220.88 | 3775.62 | 2785.92 | 3220.88 (2785.92 to 3775.62) |
| Serbia | 3234.92 | 3871.51 | 2734.73 | 3234.92 (2734.73 to 3871.51) |
| Guinea-Bissau | 3278.51 | 3630.68 | 2950.88 | 3278.51 (2950.88 to 3630.68) |
| Jordan | 3281.58 | 3998.73 | 2717.67 | 3281.58 (2717.67 to 3998.73) |
| Hungary | 3282.85 | 3982.4 | 2700.83 | 3282.85 (2700.83 to 3982.4) |
| Cote d'Ivoire | 3289.2 | 3782.69 | 2899 | 3289.2 (2899 to 3782.69) |
| Bolivia (Plurinational State of) | 3305.48 | 4042.07 | 2741.49 | 3305.48 (2741.49 to 4042.07) |
| Libya | 3327.55 | 3823.49 | 2894.44 | 3327.55 (2894.44 to 3823.49) |
| Congo | 3328.28 | 3865.32 | 2912.32 | 3328.28 (2912.32 to 3865.32) |
| Honduras | 3339.28 | 3916.6 | 2858.4 | 3339.28 (2858.4 to 3916.6) |
| Tonga | 3350.98 | 3931.63 | 2836.99 | 3350.98 (2836.99 to 3931.63) |
| Singapore | 3352.79 | 4116.4 | 2767.69 | 3352.79 (2767.69 to 4116.4) |
| Ecuador | 3353.73 | 4331.89 | 2701.34 | 3353.73 (2701.34 to 4331.89) |
| Guinea | 3356.15 | 3833.1 | 2906.44 | 3356.15 (2906.44 to 3833.1) |
| Niger | 3363.81 | 3759.59 | 2985.05 | 3363.81 (2985.05 to 3759.59) |
| Qatar | 3370.78 | 4039.03 | 2799.62 | 3370.78 (2799.62 to 4039.03) |
| Botswana | 3386.57 | 3880.35 | 2976.55 | 3386.57 (2976.55 to 3880.35) |
| Sri Lanka | 3417.39 | 3856.61 | 3042.09 | 3417.39 (3042.09 to 3856.61) |
| Kuwait | 3446.68 | 4169.47 | 2841.54 | 3446.68 (2841.54 to 4169.47) |
| Japan | 3456.79 | 4307.2 | 2818.67 | 3456.79 (2818.67 to 4307.2) |
| Djibouti | 3467.51 | 3993.23 | 3025.96 | 3467.51 (3025.96 to 3993.23) |
| Togo | 3478.8 | 4114.95 | 2894.5 | 3478.8 (2894.5 to 4114.95) |
| Trinidad and Tobago | 3500.89 | 4128.68 | 2918.61 | 3500.89 (2918.61 to 4128.68) |
| Colombia | 3513.93 | 4349.93 | 2816.31 | 3513.93 (2816.31 to 4349.93) |
| Burkina Faso | 3538.99 | 4041.03 | 3054.52 | 3538.99 (3054.52 to 4041.03) |
| Dominican Republic | 3553.45 | 4089.46 | 3067.97 | 3553.45 (3067.97 to 4089.46) |
| Montenegro | 3594.64 | 4497.29 | 2887.67 | 3594.64 (2887.67 to 4497.29) |
| Bulgaria | 3606.59 | 4410.88 | 2945.82 | 3606.59 (2945.82 to 4410.88) |
| Mozambique | 3621.88 | 4102.42 | 3198.38 | 3621.88 (3198.38 to 4102.42) |
| Oman | 3649.21 | 4504.61 | 3019.41 | 3649.21 (3019.41 to 4504.61) |
| Palestine | 3697.79 | 4401.54 | 3108.53 | 3697.79 (3108.53 to 4401.54) |
| Kiribati | 3713.08 | 3950.78 | 3469.18 | 3713.08 (3469.18 to 3950.78) |
| Italy | 3722.92 | 4568.97 | 2995.21 | 3722.92 (2995.21 to 4568.97) |
| Israel | 3765.64 | 4472.43 | 3130.59 | 3765.64 (3130.59 to 4472.43) |
| Uzbekistan | 3791.57 | 4312.93 | 3314.24 | 3791.57 (3314.24 to 4312.93) |
| Central African Republic | 3973.92 | 4350.07 | 3596.9 | 3973.92 (3596.9 to 4350.07) |
| Democratic People's Republic of Korea | 4019.71 | 4646.22 | 3493.25 | 4019.71 (3493.25 to 4646.22) |
| Yemen | 4034.87 | 4463.16 | 3650.12 | 4034.87 (3650.12 to 4463.16) |
| Malawi | 4035.97 | 4648.09 | 3509.68 | 4035.97 (3509.68 to 4648.09) |
| Nicaragua | 4046.45 | 4906.54 | 3336.28 | 4046.45 (3336.28 to 4906.54) |
| Sao Tome and Principe | 4067.5 | 4492.86 | 3648.94 | 4067.5 (3648.94 to 4492.86) |
| Croatia | 4070.44 | 4977.02 | 3362.34 | 4070.44 (3362.34 to 4977.02) |
| Syrian Arab Republic | 4099.88 | 4638.46 | 3581.16 | 4099.88 (3581.16 to 4638.46) |
| Venezuela (Bolivarian Republic of) | 4140.46 | 4995.77 | 3414.15 | 4140.46 (3414.15 to 4995.77) |
| Belarus | 4210.13 | 5059.38 | 3487.35 | 4210.13 (3487.35 to 5059.38) |
| Germany | 4262.24 | 5050.34 | 3529.51 | 4262.24 (3529.51 to 5050.34) |
| South Sudan | 4317.76 | 4906.9 | 3761.66 | 4317.76 (3761.66 to 4906.9) |
| Spain | 4332.58 | 5189.57 | 3602.39 | 4332.58 (3602.39 to 5189.57) |
| Eritrea | 4342.26 | 4816.13 | 3860.44 | 4342.26 (3860.44 to 4816.13) |
| Lebanon | 4366.55 | 5192.83 | 3681.76 | 4366.55 (3681.76 to 5192.83) |
| Comoros | 4409.89 | 5035.73 | 3826.57 | 4409.89 (3826.57 to 5035.73) |
| Romania | 4526.57 | 5513.63 | 3745.4 | 4526.57 (3745.4 to 5513.63) |
| Belgium | 4618.84 | 5517.63 | 3855.26 | 4618.84 (3855.26 to 5517.63) |
| Denmark | 4631.28 | 5507.16 | 3772.2 | 4631.28 (3772.2 to 5507.16) |
| Panama | 4634.67 | 5530.5 | 3799.54 | 4634.67 (3799.54 to 5530.5) |
| Philippines | 4646.81 | 5203.85 | 4182.43 | 4646.81 (4182.43 to 5203.85) |
| Peru | 4673.82 | 5893.1 | 3672.17 | 4673.82 (3672.17 to 5893.1) |
| Nigeria | 4718.45 | 5408.68 | 4145.01 | 4718.45 (4145.01 to 5408.68) |
| Brazil | 4732.79 | 5904.6 | 3807.07 | 4732.79 (3807.07 to 5904.6) |
| Brunei Darussalam | 4777.33 | 5443.18 | 4231.9 | 4777.33 (4231.9 to 5443.18) |
| Greece | 4781.94 | 5721.54 | 3914.27 | 4781.94 (3914.27 to 5721.54) |
| Austria | 4788.8 | 5796.41 | 3942.24 | 4788.8 (3942.24 to 5796.41) |
| Taiwan (Province of China) | 4805.89 | 5886.49 | 3982.15 | 4805.89 (3982.15 to 5886.49) |
| Afghanistan | 4812.81 | 5227.24 | 4424.63 | 4812.81 (4424.63 to 5227.24) |
| Turkiye | 4888.71 | 5680.4 | 4199.55 | 4888.71 (4199.55 to 5680.4) |
| Burundi | 4900.82 | 5420.11 | 4446.7 | 4900.82 (4446.7 to 5420.11) |
| Mauritania | 4925.61 | 5760.88 | 4282.57 | 4925.61 (4282.57 to 5760.88) |
| Bosnia and Herzegovina | 4972.16 | 6036.3 | 4133.29 | 4972.16 (4133.29 to 6036.3) |
| Suriname | 5156.87 | 6167.98 | 4355.26 | 5156.87 (4355.26 to 6167.98) |
| Uganda | 5210.14 | 5925.89 | 4469.69 | 5210.14 (4469.69 to 5925.89) |
| Uruguay | 5234.25 | 6134.54 | 4491.65 | 5234.25 (4491.65 to 6134.54) |
| Paraguay | 5298.28 | 6594.41 | 4246.15 | 5298.28 (4246.15 to 6594.41) |
| Somalia | 5358.81 | 5878.62 | 4893.38 | 5358.81 (4893.38 to 5878.62) |
| Slovenia | 5374.83 | 6479.25 | 4469.26 | 5374.83 (4469.26 to 6479.25) |
| Saint Vincent and the Grenadines | 5521.46 | 6598.29 | 4671.17 | 5521.46 (4671.17 to 6598.29) |
| Saint Kitts and Nevis | 5556.28 | 6764.94 | 4527.48 | 5556.28 (4527.48 to 6764.94) |
| North Macedonia | 5654.92 | 6652.95 | 4836.49 | 5654.92 (4836.49 to 6652.95) |
| Switzerland | 5655.46 | 6839.17 | 4526.97 | 5655.46 (4526.97 to 6839.17) |
| Antigua and Barbuda | 5708.16 | 7064.44 | 4641.03 | 5708.16 (4641.03 to 7064.44) |
| Belize | 5785.52 | 6781.32 | 4918.69 | 5785.52 (4918.69 to 6781.32) |
| Bahamas | 5803.81 | 7016.97 | 4796.26 | 5803.81 (4796.26 to 7016.97) |
| Guyana | 5810.98 | 6636.48 | 5083.99 | 5810.98 (5083.99 to 6636.48) |
| Canada | 5838.1 | 7145.53 | 4757.48 | 5838.1 (4757.48 to 7145.53) |
| Eswatini | 5909.5 | 6594.26 | 5284.79 | 5909.5 (5284.79 to 6594.26) |
| Jamaica | 5924.68 | 6969.86 | 4995.19 | 5924.68 (4995.19 to 6969.86) |
| El Salvador | 5933.93 | 7197.78 | 4897.27 | 5933.93 (4897.27 to 7197.78) |
| Argentina | 5987.5 | 7193.79 | 5010.83 | 5987.5 (5010.83 to 7193.79) |
| San Marino | 5999.89 | 7191.1 | 4970.88 | 5999.89 (4970.88 to 7191.1) |
| Costa Rica | 6022.58 | 7365.48 | 4935.34 | 6022.58 (4935.34 to 7365.48) |
| Norway | 6050.41 | 7293.18 | 4949.49 | 6050.41 (4949.49 to 7293.18) |
| Monaco | 6054.68 | 7148.92 | 4990.98 | 6054.68 (4990.98 to 7148.92) |
| Finland | 6162.98 | 7331.01 | 5111.36 | 6162.98 (5111.36 to 7331.01) |
| Dominica | 6226.6 | 7247.35 | 5435.42 | 6226.6 (5435.42 to 7247.35) |
| France | 6244.64 | 7435.83 | 5192.67 | 6244.64 (5192.67 to 7435.83) |
| Chile | 6255.93 | 7396.13 | 5349.84 | 6255.93 (5349.84 to 7396.13) |
| United Republic of Tanzania | 6320.39 | 7437.85 | 5193.79 | 6320.39 (5193.79 to 7437.85) |
| Netherlands | 6507.06 | 7766.07 | 5455.32 | 6507.06 (5455.32 to 7766.07) |
| Bermuda | 6525.87 | 8040.69 | 5161.78 | 6525.87 (5161.78 to 8040.69) |
| Andorra | 6536.57 | 7774.29 | 5337.15 | 6536.57 (5337.15 to 7774.29) |
| Malta | 6700.26 | 8081.96 | 5456 | 6700.26 (5456 to 8081.96) |
| United States Virgin Islands | 6756.44 | 8321.07 | 5488.98 | 6756.44 (5488.98 to 8321.07) |
| Saint Lucia | 6770.13 | 7760.05 | 5879.99 | 6770.13 (5879.99 to 7760.05) |
| Madagascar | 6900.99 | 7585.06 | 6255.93 | 6900.99 (6255.93 to 7585.06) |
| Ireland | 6914.52 | 8351.52 | 5760.14 | 6914.52 (5760.14 to 8351.52) |
| Luxembourg | 7102.28 | 8441.86 | 5964.05 | 7102.28 (5964.05 to 8441.86) |
| Iceland | 7330.46 | 8914.07 | 5957.62 | 7330.46 (5957.62 to 8914.07) |
| Cyprus | 7411.92 | 9009.13 | 6003.54 | 7411.92 (6003.54 to 9009.13) |
| Cuba | 7433.3 | 9228.13 | 6051.11 | 7433.3 (6051.11 to 9228.13) |
| Sweden | 7522.65 | 9106.14 | 6140.78 | 7522.65 (6140.78 to 9106.14) |
| New Zealand | 7597.42 | 8916.68 | 6397.68 | 7597.42 (6397.68 to 8916.68) |
| Greenland | 7632.22 | 8851.03 | 6612.87 | 7632.22 (6612.87 to 8851.03) |
| Australia | 7777.1 | 9110.65 | 6479.26 | 7777.1 (6479.26 to 9110.65) |
| United Arab Emirates | 8133.42 | 9175.19 | 7189.98 | 8133.42 (7189.98 to 9175.19) |
| Barbados | 8184.46 | 9718.55 | 6834.9 | 8184.46 (6834.9 to 9718.55) |
| Grenada | 8686.09 | 10626.03 | 7053.5 | 8686.09 (7053.5 to 10626.03) |
| Rwanda | 9222.69 | 10486.56 | 8050.61 | 9222.69 (8050.61 to 10486.56) |
| Poland | 9329.08 | 11200.28 | 7839.39 | 9329.08 (7839.39 to 11200.28) |
| Portugal | 9465.39 | 11402.82 | 7816.92 | 9465.39 (7816.92 to 11402.82) |
| Puerto Rico | 9990.18 | 12063.23 | 8137.16 | 9990.18 (8137.16 to 12063.23) |
| United Kingdom | 10029.77 | 11747.37 | 8450.19 | 10029.77 (8450.19 to 11747.37) |
| United States of America | 10149.92 | 11676.86 | 8887.4 | 10149.92 (8887.4 to 11676.86) |
| Haiti | 11503.65 | 12510.43 | 10476.15 | 11503.65 (10476.15 to 12510.43) |

Supplementary Table 9: Global EAPCs of prevalence for asthma

| **location** | **EAPC** | **UCI** | **LCI** | **EAPC_CI** | **val** |
| --- | --- | --- | --- | --- | --- |
| Japan | -3.6138 | -3.34028 | -3.88655 | -3.6138% (-3.88655 to -3.34028) | -3.6138 |
| Turkmenistan | -3.15585 | -3.00515 | -3.30631 | -3.15585% (-3.30631 to -3.00515) | -3.15585 |
| Russian Federation | -3.03393 | -2.92366 | -3.14407 | -3.03393% (-3.14407 to -2.92366) | -3.03393 |
| Guatemala | -2.85711 | -2.58014 | -3.1333 | -2.85711% (-3.1333 to -2.58014) | -2.85711 |
| Tuvalu | -2.84784 | -2.78432 | -2.91133 | -2.84784% (-2.91133 to -2.78432) | -2.84784 |
| South Africa | -2.76675 | -2.46331 | -3.06926 | -2.76675% (-3.06926 to -2.46331) | -2.76675 |
| New Zealand | -2.67312 | -2.36946 | -2.97583 | -2.67312% (-2.97583 to -2.36946) | -2.67312 |
| Norway | -2.65074 | -2.55115 | -2.75024 | -2.65074% (-2.75024 to -2.55115) | -2.65074 |
| Singapore | -2.6387 | -2.37939 | -2.89732 | -2.6387% (-2.89732 to -2.37939) | -2.6387 |
| Maldives | -2.4434 | -2.33695 | -2.54972 | -2.4434% (-2.54972 to -2.33695) | -2.4434 |
| Honduras | -2.41735 | -2.32751 | -2.5071 | -2.41735% (-2.5071 to -2.32751) | -2.41735 |
| Bangladesh | -2.40312 | -2.29643 | -2.5097 | -2.40312% (-2.5097 to -2.29643) | -2.40312 |
| Equatorial Guinea | -2.33654 | -2.24608 | -2.42692 | -2.33654% (-2.42692 to -2.24608) | -2.33654 |
| Tokelau | -2.26836 | -2.15207 | -2.3845 | -2.26836% (-2.3845 to -2.15207) | -2.26836 |
| Fiji | -2.26781 | -2.13908 | -2.39637 | -2.26781% (-2.39637 to -2.13908) | -2.26781 |
| Ethiopia | -2.26125 | -2.10638 | -2.41586 | -2.26125% (-2.41586 to -2.10638) | -2.26125 |
| Nauru | -2.24068 | -1.977 | -2.50365 | -2.24068% (-2.50365 to -1.977) | -2.24068 |
| Bolivia (Plurinational State of) | -2.21347 | -2.13697 | -2.28992 | -2.21347% (-2.28992 to -2.13697) | -2.21347 |
| Angola | -2.21283 | -2.03537 | -2.38997 | -2.21283% (-2.38997 to -2.03537) | -2.21283 |
| Ecuador | -2.17022 | -1.83652 | -2.50279 | -2.17022% (-2.50279 to -1.83652) | -2.17022 |
| Belarus | -2.12551 | -1.91391 | -2.33665 | -2.12551% (-2.33665 to -1.91391) | -2.12551 |
| Micronesia (Federated States of) | -2.09656 | -2.04612 | -2.14697 | -2.09656% (-2.14697 to -2.04612) | -2.09656 |
| Italy | -2.0937 | -1.92442 | -2.26268 | -2.0937% (-2.26268 to -1.92442) | -2.0937 |
| El Salvador | -2.05057 | -1.92628 | -2.1747 | -2.05057% (-2.1747 to -1.92628) | -2.05057 |
| Mexico | -2.04793 | -1.7906 | -2.30459 | -2.04793% (-2.30459 to -1.7906) | -2.04793 |
| Niue | -2.02512 | -1.82628 | -2.22356 | -2.02512% (-2.22356 to -1.82628) | -2.02512 |
| Ireland | -2.01392 | -1.90074 | -2.12696 | -2.01392% (-2.12696 to -1.90074) | -2.01392 |
| Mauritius | -2.01151 | -1.91461 | -2.10831 | -2.01151% (-2.10831 to -1.91461) | -2.01151 |
| Lao People's Democratic Republic | -1.99973 | -1.93347 | -2.06594 | -1.99973% (-2.06594 to -1.93347) | -1.99973 |
| Guam | -1.99689 | -1.54381 | -2.44789 | -1.99689% (-2.44789 to -1.54381) | -1.99689 |
| Iran (Islamic Republic of) | -1.98784 | -1.90466 | -2.07094 | -1.98784% (-2.07094 to -1.90466) | -1.98784 |
| Vanuatu | -1.98061 | -1.84468 | -2.11636 | -1.98061% (-2.11636 to -1.84468) | -1.98061 |
| Palau | -1.97836 | -1.7187 | -2.23733 | -1.97836% (-2.23733 to -1.7187) | -1.97836 |
| Sudan | -1.96976 | -1.83156 | -2.10776 | -1.96976% (-2.10776 to -1.83156) | -1.96976 |
| Nicaragua | -1.96699 | -1.88086 | -2.05305 | -1.96699% (-2.05305 to -1.88086) | -1.96699 |
| Afghanistan | -1.9385 | -1.83417 | -2.04272 | -1.9385% (-2.04272 to -1.83417) | -1.9385 |
| Brazil | -1.91157 | -1.78564 | -2.03733 | -1.91157% (-2.03733 to -1.78564) | -1.91157 |
| Pakistan | -1.89362 | -1.70641 | -2.08048 | -1.89362% (-2.08048 to -1.70641) | -1.89362 |
| Ukraine | -1.89313 | -1.80736 | -1.97882 | -1.89313% (-1.97882 to -1.80736) | -1.89313 |
| Burundi | -1.89306 | -1.82839 | -1.95769 | -1.89306% (-1.95769 to -1.82839) | -1.89306 |
| Turkiye | -1.88952 | -1.67495 | -2.10362 | -1.88952% (-2.10362 to -1.67495) | -1.88952 |
| Kiribati | -1.88313 | -1.77449 | -1.99165 | -1.88313% (-1.99165 to -1.77449) | -1.88313 |
| Myanmar | -1.87598 | -1.78869 | -1.9632 | -1.87598% (-1.9632 to -1.78869) | -1.87598 |
| Marshall Islands | -1.86893 | -1.71585 | -2.02177 | -1.86893% (-2.02177 to -1.71585) | -1.86893 |
| Belgium | -1.8681 | -1.56727 | -2.16802 | -1.8681% (-2.16802 to -1.56727) | -1.8681 |
| Greenland | -1.86325 | -1.76201 | -1.96439 | -1.86325% (-1.96439 to -1.76201) | -1.86325 |
| Madagascar | -1.85763 | -1.77597 | -1.93922 | -1.85763% (-1.93922 to -1.77597) | -1.85763 |
| Malaysia | -1.84015 | -1.74803 | -1.93219 | -1.84015% (-1.93219 to -1.74803) | -1.84015 |
| Indonesia | -1.83242 | -1.70453 | -1.96014 | -1.83242% (-1.96014 to -1.70453) | -1.83242 |
| Bhutan | -1.82851 | -1.75198 | -1.90499 | -1.82851% (-1.90499 to -1.75198) | -1.82851 |
| Israel | -1.81946 | -1.67422 | -1.96449 | -1.81946% (-1.96449 to -1.67422) | -1.81946 |
| Republic of Moldova | -1.81849 | -1.76045 | -1.8765 | -1.81849% (-1.8765 to -1.76045) | -1.81849 |
| Sweden | -1.81824 | -1.40837 | -2.22641 | -1.81824% (-2.22641 to -1.40837) | -1.81824 |
| Yemen | -1.80055 | -1.76307 | -1.83801 | -1.80055% (-1.83801 to -1.76307) | -1.80055 |
| Rwanda | -1.78461 | -1.68694 | -1.88219 | -1.78461% (-1.88219 to -1.68694) | -1.78461 |
| Luxembourg | -1.78071 | -1.68744 | -1.8739 | -1.78071% (-1.8739 to -1.68744) | -1.78071 |
| Kyrgyzstan | -1.76142 | -1.67773 | -1.84503 | -1.76142% (-1.84503 to -1.67773) | -1.76142 |
| Australia | -1.75406 | -1.46566 | -2.04162 | -1.75406% (-2.04162 to -1.46566) | -1.75406 |
| Papua New Guinea | -1.73664 | -1.63347 | -1.83971 | -1.73664% (-1.83971 to -1.63347) | -1.73664 |
| Eritrea | -1.71494 | -1.64438 | -1.78546 | -1.71494% (-1.78546 to -1.64438) | -1.71494 |
| American Samoa | -1.70685 | -1.45069 | -1.96234 | -1.70685% (-1.96234 to -1.45069) | -1.70685 |
| Venezuela (Bolivarian Republic of) | -1.70054 | -1.47623 | -1.92433 | -1.70054% (-1.92433 to -1.47623) | -1.70054 |
| Timor-Leste | -1.69696 | -1.60069 | -1.79315 | -1.69696% (-1.79315 to -1.60069) | -1.69696 |
| United Kingdom | -1.68514 | -1.49743 | -1.8725 | -1.68514% (-1.8725 to -1.49743) | -1.68514 |
| Iraq | -1.66739 | -1.56047 | -1.77419 | -1.66739% (-1.77419 to -1.56047) | -1.66739 |
| Peru | -1.6654 | -1.47861 | -1.85184 | -1.6654% (-1.85184 to -1.47861) | -1.6654 |
| Iceland | -1.6501 | -1.56102 | -1.73909 | -1.6501% (-1.73909 to -1.56102) | -1.6501 |
| Austria | -1.61641 | -1.48436 | -1.74828 | -1.61641% (-1.74828 to -1.48436) | -1.61641 |
| Northern Mariana Islands | -1.61063 | -1.12182 | -2.09703 | -1.61063% (-2.09703 to -1.12182) | -1.61063 |
| Congo | -1.59631 | -1.50773 | -1.68481 | -1.59631% (-1.68481 to -1.50773) | -1.59631 |
| Djibouti | -1.59292 | -1.50905 | -1.67673 | -1.59292% (-1.67673 to -1.50905) | -1.59292 |
| Germany | -1.58594 | -1.21152 | -1.95895 | -1.58594% (-1.95895 to -1.21152) | -1.58594 |
| Bahrain | -1.5835 | -1.46107 | -1.70577 | -1.5835% (-1.70577 to -1.46107) | -1.5835 |
| France | -1.57493 | -1.41261 | -1.73698 | -1.57493% (-1.73698 to -1.41261) | -1.57493 |
| China | -1.56827 | -1.21911 | -1.91619 | -1.56827% (-1.91619 to -1.21911) | -1.56827 |
| Latvia | -1.54851 | -1.4114 | -1.68544 | -1.54851% (-1.68544 to -1.4114) | -1.54851 |
| Colombia | -1.54098 | -1.2485 | -1.8326 | -1.54098% (-1.8326 to -1.2485) | -1.54098 |
| Croatia | -1.50616 | -1.33162 | -1.68039 | -1.50616% (-1.68039 to -1.33162) | -1.50616 |
| North Macedonia | -1.50009 | -1.27867 | -1.72102 | -1.50009% (-1.72102 to -1.27867) | -1.50009 |
| Guinea-Bissau | -1.48725 | -1.43613 | -1.53834 | -1.48725% (-1.53834 to -1.43613) | -1.48725 |
| Uzbekistan | -1.48679 | -1.34637 | -1.627 | -1.48679% (-1.627 to -1.34637) | -1.48679 |
| Denmark | -1.47821 | -1.26146 | -1.69448 | -1.47821% (-1.69448 to -1.26146) | -1.47821 |
| Republic of Korea | -1.47207 | -1.35855 | -1.58546 | -1.47207% (-1.58546 to -1.35855) | -1.47207 |
| Solomon Islands | -1.46589 | -1.35751 | -1.57416 | -1.46589% (-1.57416 to -1.35751) | -1.46589 |
| Andorra | -1.45126 | -1.34908 | -1.55333 | -1.45126% (-1.55333 to -1.34908) | -1.45126 |
| Thailand | -1.4488 | -1.30711 | -1.59028 | -1.4488% (-1.59028 to -1.30711) | -1.4488 |
| Egypt | -1.44199 | -1.36917 | -1.51475 | -1.44199% (-1.51475 to -1.36917) | -1.44199 |
| Switzerland | -1.4235 | -1.37341 | -1.47357 | -1.4235% (-1.47357 to -1.37341) | -1.4235 |
| Cabo Verde | -1.41797 | -1.26998 | -1.56575 | -1.41797% (-1.56575 to -1.26998) | -1.41797 |
| India | -1.41776 | -1.09405 | -1.74041 | -1.41776% (-1.74041 to -1.09405) | -1.41776 |
| Netherlands | -1.38661 | -1.10825 | -1.6642 | -1.38661% (-1.6642 to -1.10825) | -1.38661 |
| Panama | -1.35554 | -1.28431 | -1.42672 | -1.35554% (-1.42672 to -1.28431) | -1.35554 |
| South Sudan | -1.33609 | -1.26 | -1.41213 | -1.33609% (-1.41213 to -1.26) | -1.33609 |
| Liberia | -1.33445 | -1.27252 | -1.39634 | -1.33445% (-1.39634 to -1.27252) | -1.33445 |
| Cambodia | -1.33394 | -1.26016 | -1.40767 | -1.33394% (-1.40767 to -1.26016) | -1.33394 |
| Estonia | -1.33236 | -1.20195 | -1.46261 | -1.33236% (-1.46261 to -1.20195) | -1.33236 |
| Malta | -1.3263 | -1.259 | -1.39355 | -1.3263% (-1.39355 to -1.259) | -1.3263 |
| Gabon | -1.3114 | -1.25695 | -1.36583 | -1.3114% (-1.36583 to -1.25695) | -1.3114 |
| Philippines | -1.29024 | -1.19496 | -1.38543 | -1.29024% (-1.38543 to -1.19496) | -1.29024 |
| Bulgaria | -1.27656 | -1.18492 | -1.36812 | -1.27656% (-1.36812 to -1.18492) | -1.27656 |
| Mongolia | -1.27071 | -1.20214 | -1.33923 | -1.27071% (-1.33923 to -1.20214) | -1.27071 |
| Namibia | -1.23962 | -1.17723 | -1.30197 | -1.23962% (-1.30197 to -1.17723) | -1.23962 |
| Comoros | -1.21921 | -1.14663 | -1.29175 | -1.21921% (-1.29175 to -1.14663) | -1.21921 |
| Sao Tome and Principe | -1.20843 | -1.12829 | -1.28852 | -1.20843% (-1.28852 to -1.12829) | -1.20843 |
| Samoa | -1.20793 | -1.15437 | -1.26146 | -1.20793% (-1.26146 to -1.15437) | -1.20793 |
| Benin | -1.20201 | -1.15333 | -1.25068 | -1.20201% (-1.25068 to -1.15333) | -1.20201 |
| Sri Lanka | -1.19973 | -1.12826 | -1.27115 | -1.19973% (-1.27115 to -1.12826) | -1.19973 |
| Kenya | -1.19821 | -1.07947 | -1.31681 | -1.19821% (-1.31681 to -1.07947) | -1.19821 |
| Uganda | -1.18801 | -1.11575 | -1.26023 | -1.18801% (-1.26023 to -1.11575) | -1.18801 |
| Costa Rica | -1.17124 | -1.01821 | -1.32404 | -1.17124% (-1.32404 to -1.01821) | -1.17124 |
| Slovenia | -1.17115 | -1.1158 | -1.22646 | -1.17115% (-1.22646 to -1.1158) | -1.17115 |
| Mozambique | -1.1687 | -1.10316 | -1.23418 | -1.1687% (-1.23418 to -1.10316) | -1.1687 |
| Greece | -1.16832 | -1.04177 | -1.2947 | -1.16832% (-1.2947 to -1.04177) | -1.16832 |
| Mauritania | -1.15785 | -1.0863 | -1.22935 | -1.15785% (-1.22935 to -1.0863) | -1.15785 |
| Somalia | -1.15486 | -1.1085 | -1.20119 | -1.15486% (-1.20119 to -1.1085) | -1.15486 |
| Azerbaijan | -1.1504 | -1.07873 | -1.22202 | -1.1504% (-1.22202 to -1.07873) | -1.1504 |
| Tajikistan | -1.14512 | -1.09949 | -1.19073 | -1.14512% (-1.19073 to -1.09949) | -1.14512 |
| Poland | -1.14201 | -0.71888 | -1.56334 | -1.14201% (-1.56334 to -0.71888) | -1.14201 |
| Cook Islands | -1.12214 | -0.66587 | -1.57632 | -1.12214% (-1.57632 to -0.66587) | -1.12214 |
| Niger | -1.1151 | -1.05387 | -1.1763 | -1.1151% (-1.1763 to -1.05387) | -1.1151 |
| Uruguay | -1.10976 | -1.04622 | -1.17326 | -1.10976% (-1.17326 to -1.04622) | -1.10976 |
| Portugal | -1.10635 | -0.7634 | -1.4481 | -1.10635% (-1.4481 to -0.7634) | -1.10635 |
| Tonga | -1.0954 | -0.97198 | -1.21867 | -1.0954% (-1.21867 to -0.97198) | -1.0954 |
| United Arab Emirates | -1.08546 | -0.99951 | -1.17134 | -1.08546% (-1.17134 to -0.99951) | -1.08546 |
| Malawi | -1.08303 | -1.01374 | -1.15227 | -1.08303% (-1.15227 to -1.01374) | -1.08303 |
| Dominican Republic | -1.07707 | -0.91458 | -1.23929 | -1.07707% (-1.23929 to -0.91458) | -1.07707 |
| Zambia | -1.07268 | -0.99545 | -1.14986 | -1.07268% (-1.14986 to -0.99545) | -1.07268 |
| Hungary | -1.04518 | -0.91372 | -1.17647 | -1.04518% (-1.17647 to -0.91372) | -1.04518 |
| Senegal | -1.04295 | -0.96328 | -1.12255 | -1.04295% (-1.12255 to -0.96328) | -1.04295 |
| Nepal | -1.014 | -0.95461 | -1.07335 | -1.014% (-1.07335 to -0.95461) | -1.014 |
| Seychelles | -1.00329 | -0.86506 | -1.14132 | -1.00329% (-1.14132 to -0.86506) | -1.00329 |
| Jamaica | -0.99885 | -0.85607 | -1.14143 | -0.99885% (-1.14143 to -0.85607) | -0.99885 |
| Democratic Republic of the Congo | -0.98216 | -0.91119 | -1.05308 | -0.98216% (-1.05308 to -0.91119) | -0.98216 |
| Eswatini | -0.96854 | -0.88623 | -1.05078 | -0.96854% (-1.05078 to -0.88623) | -0.96854 |
| Democratic People's Republic of Korea | -0.95855 | -0.89022 | -1.02684 | -0.95855% (-1.02684 to -0.89022) | -0.95855 |
| Libya | -0.95295 | -0.89597 | -1.0099 | -0.95295% (-1.0099 to -0.89597) | -0.95295 |
| Botswana | -0.95199 | -0.89572 | -1.00822 | -0.95199% (-1.00822 to -0.89572) | -0.95199 |
| Nigeria | -0.94258 | -0.87303 | -1.01208 | -0.94258% (-1.01208 to -0.87303) | -0.94258 |
| Ghana | -0.93435 | -0.87911 | -0.98955 | -0.93435% (-0.98955 to -0.87911) | -0.93435 |
| Guinea | -0.92276 | -0.87401 | -0.97148 | -0.92276% (-0.97148 to -0.87401) | -0.92276 |
| Central African Republic | -0.91454 | -0.83185 | -0.99717 | -0.91454% (-0.99717 to -0.83185) | -0.91454 |
| Jordan | -0.90744 | -0.76537 | -1.0493 | -0.90744% (-1.0493 to -0.76537) | -0.90744 |
| Cameroon | -0.90519 | -0.84349 | -0.96686 | -0.90519% (-0.96686 to -0.84349) | -0.90519 |
| Serbia | -0.90264 | -0.76771 | -1.0374 | -0.90264% (-1.0374 to -0.76771) | -0.90264 |
| Georgia | -0.90171 | -0.79952 | -1.00379 | -0.90171% (-1.00379 to -0.79952) | -0.90171 |
| Argentina | -0.88574 | -0.83636 | -0.93509 | -0.88574% (-0.93509 to -0.83636) | -0.88574 |
| Gambia | -0.8801 | -0.82686 | -0.9333 | -0.8801% (-0.9333 to -0.82686) | -0.8801 |
| Romania | -0.87334 | -0.74075 | -1.00575 | -0.87334% (-1.00575 to -0.74075) | -0.87334 |
| Haiti | -0.86813 | -0.7969 | -0.9393 | -0.86813% (-0.9393 to -0.7969) | -0.86813 |
| Viet Nam | -0.86744 | -0.741 | -0.99371 | -0.86744% (-0.99371 to -0.741) | -0.86744 |
| Puerto Rico | -0.84584 | -0.67961 | -1.01179 | -0.84584% (-1.01179 to -0.67961) | -0.84584 |
| Sierra Leone | -0.82647 | -0.77938 | -0.87353 | -0.82647% (-0.87353 to -0.77938) | -0.82647 |
| Brunei Darussalam | -0.82551 | -0.76183 | -0.88916 | -0.82551% (-0.88916 to -0.76183) | -0.82551 |
| Tunisia | -0.81596 | -0.74841 | -0.88347 | -0.81596% (-0.88347 to -0.74841) | -0.81596 |
| Lebanon | -0.81169 | -0.7652 | -0.85815 | -0.81169% (-0.85815 to -0.7652) | -0.81169 |
| Saint Lucia | -0.79635 | -0.66998 | -0.92256 | -0.79635% (-0.92256 to -0.66998) | -0.79635 |
| Qatar | -0.79544 | -0.73341 | -0.85742 | -0.79544% (-0.85742 to -0.73341) | -0.79544 |
| Kuwait | -0.77765 | -0.65819 | -0.89697 | -0.77765% (-0.89697 to -0.65819) | -0.77765 |
| Cote d'Ivoire | -0.7403 | -0.66684 | -0.8137 | -0.7403% (-0.8137 to -0.66684) | -0.7403 |
| Algeria | -0.73677 | -0.68591 | -0.78761 | -0.73677% (-0.78761 to -0.68591) | -0.73677 |
| Mali | -0.7156 | -0.65356 | -0.77759 | -0.7156% (-0.77759 to -0.65356) | -0.7156 |
| Lithuania | -0.68982 | -0.59743 | -0.78212 | -0.68982% (-0.78212 to -0.59743) | -0.68982 |
| Palestine | -0.66228 | -0.55888 | -0.76557 | -0.66228% (-0.76557 to -0.55888) | -0.66228 |
| Lesotho | -0.64597 | -0.58856 | -0.70335 | -0.64597% (-0.70335 to -0.58856) | -0.64597 |
| Cyprus | -0.63947 | -0.55731 | -0.72156 | -0.63947% (-0.72156 to -0.55731) | -0.63947 |
| Canada | -0.62795 | -0.43249 | -0.82302 | -0.62795% (-0.82302 to -0.43249) | -0.62795 |
| Finland | -0.59706 | -0.53832 | -0.65576 | -0.59706% (-0.65576 to -0.53832) | -0.59706 |
| Belize | -0.58202 | -0.45122 | -0.71266 | -0.58202% (-0.71266 to -0.45122) | -0.58202 |
| Taiwan (Province of China) | -0.56806 | -0.30999 | -0.82546 | -0.56806% (-0.82546 to -0.30999) | -0.56806 |
| Trinidad and Tobago | -0.56735 | -0.00315 | -1.12837 | -0.56735% (-1.12837 to -0.00315) | -0.56735 |
| Chad | -0.56005 | -0.51541 | -0.60468 | -0.56005% (-0.60468 to -0.51541) | -0.56005 |
| Grenada | -0.55562 | -0.47535 | -0.63583 | -0.55562% (-0.63583 to -0.47535) | -0.55562 |
| Czechia | -0.55152 | -0.47699 | -0.62599 | -0.55152% (-0.62599 to -0.47699) | -0.55152 |
| Morocco | -0.55115 | -0.47229 | -0.62994 | -0.55115% (-0.62994 to -0.47229) | -0.55115 |
| Spain | -0.52815 | -0.32149 | -0.73438 | -0.52815% (-0.73438 to -0.32149) | -0.52815 |
| Guyana | -0.51703 | -0.35448 | -0.67932 | -0.51703% (-0.67932 to -0.35448) | -0.51703 |
| Togo | -0.50641 | -0.45474 | -0.55805 | -0.50641% (-0.55805 to -0.45474) | -0.50641 |
| Monaco | -0.47084 | -0.42219 | -0.51946 | -0.47084% (-0.51946 to -0.42219) | -0.47084 |
| Burkina Faso | -0.46231 | -0.41156 | -0.51303 | -0.46231% (-0.51303 to -0.41156) | -0.46231 |
| Antigua and Barbuda | -0.46091 | -0.24967 | -0.67171 | -0.46091% (-0.67171 to -0.24967) | -0.46091 |
| Chile | -0.45079 | -0.33323 | -0.56821 | -0.45079% (-0.56821 to -0.33323) | -0.45079 |
| Dominica | -0.42677 | -0.2084 | -0.64467 | -0.42677% (-0.64467 to -0.2084) | -0.42677 |
| Slovakia | -0.41511 | -0.35766 | -0.47252 | -0.41511% (-0.47252 to -0.35766) | -0.41511 |
| Cuba | -0.412 | -0.29959 | -0.52427 | -0.412% (-0.52427 to -0.29959) | -0.412 |
| Suriname | -0.4089 | -0.25967 | -0.55791 | -0.4089% (-0.55791 to -0.25967) | -0.4089 |
| Armenia | -0.40888 | -0.15004 | -0.66704 | -0.40888% (-0.66704 to -0.15004) | -0.40888 |
| Albania | -0.40604 | -0.26598 | -0.5459 | -0.40604% (-0.5459 to -0.26598) | -0.40604 |
| United Republic of Tanzania | -0.36977 | -0.30236 | -0.43714 | -0.36977% (-0.43714 to -0.30236) | -0.36977 |
| Saint Vincent and the Grenadines | -0.33973 | -0.17059 | -0.50858 | -0.33973% (-0.50858 to -0.17059) | -0.33973 |
| Bosnia and Herzegovina | -0.33409 | -0.27272 | -0.39542 | -0.33409% (-0.39542 to -0.27272) | -0.33409 |
| Kazakhstan | -0.32368 | -0.23528 | -0.41199 | -0.32368% (-0.41199 to -0.23528) | -0.32368 |
| San Marino | -0.31987 | -0.28542 | -0.3543 | -0.31987% (-0.3543 to -0.28542) | -0.31987 |
| Saint Kitts and Nevis | -0.29924 | -0.08841 | -0.50962 | -0.29924% (-0.50962 to -0.08841) | -0.29924 |
| United States Virgin Islands | -0.299 | -0.16937 | -0.42847 | -0.299% (-0.42847 to -0.16937) | -0.299 |
| Bahamas | -0.21821 | 0.0064 | -0.44233 | -0.21821% (-0.44233 to 0.0064) | -0.21821 |
| Bermuda | -0.1722 | 0.04094 | -0.38489 | -0.1722% (-0.38489 to 0.04094) | -0.1722 |
| Zimbabwe | -0.16651 | -0.11468 | -0.21831 | -0.16651% (-0.21831 to -0.11468) | -0.16651 |
| Montenegro | -0.14514 | -0.07218 | -0.21805 | -0.14514% (-0.21805 to -0.07218) | -0.14514 |
| Paraguay | -0.00827 | 0.07636 | -0.09282 | -0.00827% (-0.09282 to 0.07636) | -0.00827 |
| Saudi Arabia | 0.09626 | 0.21437 | -0.02171 | 0.09626% (-0.02171 to 0.21437) | 0.09626 |
| Barbados | 0.14062 | 0.26909 | 0.01231 | 0.14062% (0.01231 to 0.26909) | 0.14062 |
| Syrian Arab Republic | 0.1696 | 0.41381 | -0.07403 | 0.1696% (-0.07403 to 0.41381) | 0.1696 |
| Oman | 0.3107 | 0.43141 | 0.19014 | 0.3107% (0.19014 to 0.43141) | 0.3107 |
| United States of America | 0.99039 | 1.36049 | 0.62164 | 0.99039% (0.62164 to 1.36049) | 0.99039 |

Supplementary Table 10: Global ASIR for asthma in 1990

| **location** | **val** | **upper** | **lower** | **Rate_1990** |
| --- | --- | --- | --- | --- |
| Kazakhstan | 301.11 | 379.11 | 249.15 | 301.11 (249.15 to 379.11) |
| Lesotho | 324.97 | 387.61 | 274.42 | 324.97 (274.42 to 387.61) |
| Nepal | 355.32 | 421.38 | 305.98 | 355.32 (305.98 to 421.38) |
| Armenia | 359.76 | 463.39 | 288.13 | 359.76 (288.13 to 463.39) |
| Cameroon | 394.91 | 486.46 | 318.64 | 394.91 (318.64 to 486.46) |
| Bhutan | 398.66 | 476.15 | 336.12 | 398.66 (336.12 to 476.15) |
| Netherlands | 405.66 | 495.31 | 337.69 | 405.66 (337.69 to 495.31) |
| Zimbabwe | 411.88 | 517.27 | 334.61 | 411.88 (334.61 to 517.27) |
| Georgia | 413.51 | 511.3 | 339.09 | 413.51 (339.09 to 511.3) |
| Pakistan | 414.48 | 512.06 | 348.83 | 414.48 (348.83 to 512.06) |
| Spain | 424.47 | 529.72 | 349.91 | 424.47 (349.91 to 529.72) |
| Seychelles | 429.16 | 533.43 | 352.51 | 429.16 (352.51 to 533.43) |
| Mali | 442.31 | 538.59 | 369.02 | 442.31 (369.02 to 538.59) |
| Ghana | 445.54 | 543.95 | 369.27 | 445.54 (369.27 to 543.95) |
| Azerbaijan | 447.86 | 548.95 | 377.11 | 447.86 (377.11 to 548.95) |
| Tajikistan | 448.34 | 554.93 | 373.68 | 448.34 (373.68 to 554.93) |
| American Samoa | 457.15 | 559.16 | 381.85 | 457.15 (381.85 to 559.16) |
| Cook Islands | 462.69 | 578.56 | 384.15 | 462.69 (384.15 to 578.56) |
| Saudi Arabia | 463.41 | 569.14 | 387.46 | 463.41 (387.46 to 569.14) |
| Morocco | 464.29 | 546.02 | 398.01 | 464.29 (398.01 to 546.02) |
| Northern Mariana Islands | 474.41 | 603.24 | 382.14 | 474.41 (382.14 to 603.24) |
| Greece | 475.67 | 590.84 | 395.14 | 475.67 (395.14 to 590.84) |
| Bangladesh | 476.2 | 585.18 | 404.02 | 476.2 (404.02 to 585.18) |
| Niue | 485.6 | 580.69 | 415.88 | 485.6 (415.88 to 580.69) |
| Mongolia | 490.07 | 595.95 | 414.32 | 490.07 (414.32 to 595.95) |
| Israel | 492.06 | 605.99 | 409.61 | 492.06 (409.61 to 605.99) |
| Chad | 495.57 | 613.81 | 409.03 | 495.57 (409.03 to 613.81) |
| San Marino | 502.5 | 631.55 | 403.07 | 502.5 (403.07 to 631.55) |
| Senegal | 505.87 | 627.4 | 419.05 | 505.87 (419.05 to 627.4) |
| Zambia | 507.57 | 619.44 | 416.32 | 507.57 (416.32 to 619.44) |
| Finland | 507.68 | 606.87 | 416.72 | 507.68 (416.72 to 606.87) |
| Albania | 508.9 | 621.43 | 430.07 | 508.9 (430.07 to 621.43) |
| Samoa | 509.38 | 602.07 | 429.3 | 509.38 (429.3 to 602.07) |
| Monaco | 513.02 | 655.64 | 411.64 | 513.02 (411.64 to 655.64) |
| Myanmar | 521.38 | 610.19 | 454.34 | 521.38 (454.34 to 610.19) |
| Cambodia | 523.81 | 620.94 | 448.57 | 523.81 (448.57 to 620.94) |
| China | 524.81 | 672.76 | 421.31 | 524.81 (421.31 to 672.76) |
| Guam | 526.24 | 681.4 | 417.41 | 526.24 (417.41 to 681.4) |
| Lithuania | 527.42 | 651.71 | 434.2 | 527.42 (434.2 to 651.71) |
| Austria | 531.8 | 640.4 | 447.89 | 531.8 (447.89 to 640.4) |
| Solomon Islands | 538.58 | 618.74 | 466.34 | 538.58 (466.34 to 618.74) |
| Oman | 539.06 | 670.24 | 440.34 | 539.06 (440.34 to 670.24) |
| Kyrgyzstan | 543.69 | 670.65 | 449.03 | 543.69 (449.03 to 670.65) |
| Palau | 547.79 | 675.16 | 459.21 | 547.79 (459.21 to 675.16) |
| Slovakia | 547.91 | 688.32 | 443.54 | 547.91 (443.54 to 688.32) |
| Marshall Islands | 548.97 | 635.6 | 476.46 | 548.97 (476.46 to 635.6) |
| Estonia | 550.64 | 651.89 | 468.94 | 550.64 (468.94 to 651.89) |
| Cabo Verde | 551.07 | 694.73 | 446.65 | 551.07 (446.65 to 694.73) |
| Tokelau | 551.58 | 646.82 | 473.2 | 551.58 (473.2 to 646.82) |
| Namibia | 556.52 | 681.62 | 464.99 | 556.52 (464.99 to 681.62) |
| Czechia | 558.52 | 703.24 | 454.11 | 558.52 (454.11 to 703.24) |
| Gambia | 562.63 | 696.07 | 462.98 | 562.63 (462.98 to 696.07) |
| Belgium | 566.2 | 685.35 | 467.47 | 566.2 (467.47 to 685.35) |
| Malaysia | 567.15 | 676.96 | 488.56 | 567.15 (488.56 to 676.96) |
| Germany | 568.47 | 687.18 | 477.13 | 568.47 (477.13 to 687.18) |
| Italy | 570.5 | 670.87 | 486.63 | 570.5 (486.63 to 670.87) |
| Algeria | 572.87 | 679.64 | 482.79 | 572.87 (482.79 to 679.64) |
| Republic of Korea | 574.31 | 690.53 | 478.25 | 574.31 (478.25 to 690.53) |
| Viet Nam | 582.47 | 716.63 | 481.53 | 582.47 (481.53 to 716.63) |
| Vanuatu | 584.1 | 668.73 | 512.35 | 584.1 (512.35 to 668.73) |
| Denmark | 586.61 | 712.87 | 487.84 | 586.61 (487.84 to 712.87) |
| Liberia | 586.77 | 733.93 | 479.21 | 586.77 (479.21 to 733.93) |
| Switzerland | 587.94 | 724.8 | 489.83 | 587.94 (489.83 to 724.8) |
| Nauru | 590.76 | 701.61 | 512.79 | 590.76 (512.79 to 701.61) |
| South Africa | 591.98 | 737.49 | 482.79 | 591.98 (482.79 to 737.49) |
| Cyprus | 593.01 | 737.85 | 480.73 | 593.01 (480.73 to 737.85) |
| Montenegro | 598.8 | 755.37 | 485.57 | 598.8 (485.57 to 755.37) |
| Micronesia (Federated States of) | 610.37 | 707.52 | 530.46 | 610.37 (530.46 to 707.52) |
| Cote d'Ivoire | 614.16 | 770.73 | 506.47 | 614.16 (506.47 to 770.73) |
| Lao People's Democratic Republic | 615.02 | 749.53 | 517.43 | 615.02 (517.43 to 749.53) |
| Maldives | 616.83 | 737.37 | 522.23 | 616.83 (522.23 to 737.37) |
| Andorra | 617.83 | 754.7 | 507.15 | 617.83 (507.15 to 754.7) |
| Republic of Moldova | 618.12 | 752.96 | 505.63 | 618.12 (505.63 to 752.96) |
| Democratic Republic of the Congo | 622.98 | 765.11 | 514.7 | 622.98 (514.7 to 765.11) |
| Malta | 623.91 | 739.42 | 527.83 | 623.91 (527.83 to 739.42) |
| Tunisia | 624.01 | 746.59 | 524.94 | 624.01 (524.94 to 746.59) |
| France | 627.48 | 744.87 | 527.39 | 627.48 (527.39 to 744.87) |
| Gabon | 628.31 | 772.46 | 508.12 | 628.31 (508.12 to 772.46) |
| Syrian Arab Republic | 628.55 | 744.5 | 537.84 | 628.55 (537.84 to 744.5) |
| Togo | 631.18 | 788.82 | 505.19 | 631.18 (505.19 to 788.82) |
| Turkmenistan | 633.04 | 770.34 | 529.33 | 633.04 (529.33 to 770.34) |
| Hungary | 636.84 | 794.22 | 526.6 | 636.84 (526.6 to 794.22) |
| Latvia | 638.04 | 772.06 | 535.49 | 638.04 (535.49 to 772.06) |
| Serbia | 639.82 | 794.75 | 531.85 | 639.82 (531.85 to 794.75) |
| Qatar | 642.82 | 803.17 | 528.31 | 642.82 (528.31 to 803.17) |
| Kenya | 644.11 | 819.55 | 525.49 | 644.11 (525.49 to 819.55) |
| Uzbekistan | 645.17 | 774.52 | 543.56 | 645.17 (543.56 to 774.52) |
| Burkina Faso | 649.17 | 811.6 | 538.25 | 649.17 (538.25 to 811.6) |
| Libya | 650.99 | 789.85 | 547.91 | 650.99 (547.91 to 789.85) |
| Jordan | 651.27 | 803.63 | 542.01 | 651.27 (542.01 to 803.63) |
| Thailand | 660.52 | 794.56 | 556.09 | 660.52 (556.09 to 794.56) |
| Benin | 662.12 | 815.3 | 553.27 | 662.12 (553.27 to 815.3) |
| Brunei Darussalam | 665.49 | 812.39 | 556.64 | 665.49 (556.64 to 812.39) |
| Fiji | 668.9 | 764.64 | 572.57 | 668.9 (572.57 to 764.64) |
| Botswana | 670.07 | 811.9 | 560.1 | 670.07 (560.1 to 811.9) |
| Tonga | 674.62 | 824.62 | 556.54 | 674.62 (556.54 to 824.62) |
| Indonesia | 679.04 | 824.42 | 573.03 | 679.04 (573.03 to 824.42) |
| Palestine | 679.04 | 835.84 | 563.47 | 679.04 (563.47 to 835.84) |
| Sierra Leone | 679.93 | 840.19 | 555.43 | 679.93 (555.43 to 840.19) |
| Luxembourg | 683.2 | 849.57 | 565.63 | 683.2 (565.63 to 849.57) |
| Kuwait | 689.33 | 872.58 | 561.59 | 689.33 (561.59 to 872.58) |
| Sri Lanka | 689.64 | 803.48 | 602.74 | 689.64 (602.74 to 803.48) |
| India | 690.55 | 887.57 | 547.48 | 690.55 (547.48 to 887.57) |
| Sudan | 691.79 | 829.36 | 566.86 | 691.79 (566.86 to 829.36) |
| Guinea | 693.89 | 874.86 | 559.54 | 693.89 (559.54 to 874.86) |
| Bahrain | 700.25 | 850.4 | 589.18 | 700.25 (589.18 to 850.4) |
| Tuvalu | 701.28 | 818.63 | 612.39 | 701.28 (612.39 to 818.63) |
| Bulgaria | 701.55 | 869.88 | 579.61 | 701.55 (579.61 to 869.88) |
| Australia | 704.14 | 873.13 | 572.39 | 704.14 (572.39 to 873.13) |
| Argentina | 704.48 | 872.62 | 582.35 | 704.48 (582.35 to 872.62) |
| Iraq | 706.98 | 843.31 | 602.89 | 706.98 (602.89 to 843.31) |
| Chile | 713.48 | 865.78 | 594.92 | 713.48 (594.92 to 865.78) |
| Singapore | 717.43 | 863.41 | 599.62 | 717.43 (599.62 to 863.41) |
| Egypt | 723.99 | 877.47 | 600.92 | 723.99 (600.92 to 877.47) |
| Ireland | 728.51 | 874.8 | 607.62 | 728.51 (607.62 to 874.8) |
| Ukraine | 731.55 | 938.39 | 586.72 | 731.55 (586.72 to 938.39) |
| Uruguay | 731.89 | 881.5 | 593.05 | 731.89 (593.05 to 881.5) |
| Taiwan (Province of China) | 734.83 | 907.21 | 609.57 | 734.83 (609.57 to 907.21) |
| Equatorial Guinea | 736.05 | 890.46 | 617.35 | 736.05 (617.35 to 890.46) |
| Russian Federation | 745.15 | 923.05 | 619.41 | 745.15 (619.41 to 923.05) |
| Mauritius | 750.22 | 915.79 | 632.71 | 750.22 (632.71 to 915.79) |
| Iran (Islamic Republic of) | 750.58 | 899.11 | 632.68 | 750.58 (632.68 to 899.11) |
| Niger | 751.12 | 921.36 | 626 | 751.12 (626 to 921.36) |
| Ethiopia | 751.35 | 917.89 | 630.42 | 751.35 (630.42 to 917.89) |
| Guinea-Bissau | 751.63 | 908.25 | 633.15 | 751.63 (633.15 to 908.25) |
| Congo | 755.27 | 931.69 | 625.84 | 755.27 (625.84 to 931.69) |
| Lebanon | 757.86 | 913.47 | 630.75 | 757.86 (630.75 to 913.47) |
| Timor-Leste | 761.3 | 915.42 | 653.34 | 761.3 (653.34 to 915.42) |
| Angola | 765.11 | 942.08 | 642.39 | 765.11 (642.39 to 942.08) |
| Democratic People's Republic of Korea | 765.44 | 954.29 | 634.94 | 765.44 (634.94 to 954.29) |
| Papua New Guinea | 767.2 | 893.47 | 664.67 | 767.2 (664.67 to 893.47) |
| Romania | 776.4 | 968.42 | 639.37 | 776.4 (639.37 to 968.42) |
| Central African Republic | 780.91 | 942.47 | 667.94 | 780.91 (667.94 to 942.47) |
| Mexico | 789.81 | 999.02 | 636.35 | 789.81 (636.35 to 999.02) |
| Bosnia and Herzegovina | 791.04 | 990.08 | 649.34 | 791.04 (649.34 to 990.08) |
| Trinidad and Tobago | 801.52 | 1008.89 | 656.1 | 801.52 (656.1 to 1008.89) |
| Croatia | 808.18 | 983.92 | 677.32 | 808.18 (677.32 to 983.92) |
| Portugal | 811.26 | 972.37 | 667.36 | 811.26 (667.36 to 972.37) |
| Sao Tome and Principe | 818 | 992.05 | 673.71 | 818 (673.71 to 992.05) |
| Belarus | 844.56 | 1035.85 | 696.6 | 844.56 (696.6 to 1035.85) |
| Mozambique | 851.39 | 1059.76 | 706.74 | 851.39 (706.74 to 1059.76) |
| Malawi | 854.56 | 1062.71 | 707.14 | 854.56 (707.14 to 1062.71) |
| Kiribati | 856.03 | 986.39 | 753.7 | 856.03 (753.7 to 986.39) |
| Djibouti | 860.49 | 1065.65 | 713.02 | 860.49 (713.02 to 1065.65) |
| Iceland | 867.91 | 1144.33 | 698.6 | 867.91 (698.6 to 1144.33) |
| Mauritania | 870.05 | 1075.14 | 706.75 | 870.05 (706.75 to 1075.14) |
| Yemen | 884.35 | 1036.13 | 767.71 | 884.35 (767.71 to 1036.13) |
| Nigeria | 906.78 | 1123.08 | 747.62 | 906.78 (747.62 to 1123.08) |
| Slovenia | 916.92 | 1121.81 | 762.82 | 916.92 (762.82 to 1121.81) |
| Sweden | 918.49 | 1103.09 | 772.7 | 918.49 (772.7 to 1103.09) |
| Comoros | 921.41 | 1121.94 | 737.78 | 921.41 (737.78 to 1121.94) |
| Colombia | 935.67 | 1181.86 | 746.14 | 935.67 (746.14 to 1181.86) |
| Dominican Republic | 944.8 | 1139.87 | 789.9 | 944.8 (789.9 to 1139.87) |
| Suriname | 976.69 | 1240.44 | 795.06 | 976.69 (795.06 to 1240.44) |
| Paraguay | 990.69 | 1247.3 | 807.06 | 990.69 (807.06 to 1247.3) |
| Afghanistan | 995.28 | 1158.13 | 868.15 | 995.28 (868.15 to 1158.13) |
| Saint Kitts and Nevis | 996.89 | 1267.89 | 806.76 | 996.89 (806.76 to 1267.89) |
| Guyana | 1001.84 | 1240.77 | 830.45 | 1001.84 (830.45 to 1240.77) |
| Saint Vincent and the Grenadines | 1006.7 | 1267.93 | 811.57 | 1006.7 (811.57 to 1267.93) |
| Norway | 1009.71 | 1189.49 | 853.16 | 1009.71 (853.16 to 1189.49) |
| Turkiye | 1018.81 | 1212.92 | 856.12 | 1018.81 (856.12 to 1212.92) |
| North Macedonia | 1022.6 | 1217.23 | 877.01 | 1022.6 (877.01 to 1217.23) |
| South Sudan | 1023.31 | 1287.41 | 855.49 | 1023.31 (855.49 to 1287.41) |
| Eritrea | 1026.47 | 1254.22 | 863.31 | 1026.47 (863.31 to 1254.22) |
| Bahamas | 1030.73 | 1304.34 | 827.38 | 1030.73 (827.38 to 1304.34) |
| Eswatini | 1040.84 | 1279.59 | 859.14 | 1040.84 (859.14 to 1279.59) |
| United Republic of Tanzania | 1042.62 | 1303.12 | 819.38 | 1042.62 (819.38 to 1303.12) |
| United Kingdom | 1049.62 | 1276.74 | 864.14 | 1049.62 (864.14 to 1276.74) |
| Dominica | 1050.84 | 1321.51 | 856.43 | 1050.84 (856.43 to 1321.51) |
| Uganda | 1052.24 | 1322.12 | 864.34 | 1052.24 (864.34 to 1322.12) |
| Japan | 1056.02 | 1310.29 | 866.69 | 1056.02 (866.69 to 1310.29) |
| Honduras | 1066.52 | 1331.63 | 876.13 | 1066.52 (876.13 to 1331.63) |
| Guatemala | 1067.33 | 1328.15 | 874.93 | 1067.33 (874.93 to 1328.15) |
| New Zealand | 1073.88 | 1368.02 | 852.58 | 1073.88 (852.58 to 1368.02) |
| Venezuela (Bolivarian Republic of) | 1075.23 | 1363.24 | 873.69 | 1075.23 (873.69 to 1363.24) |
| Philippines | 1076.15 | 1303.75 | 908.56 | 1076.15 (908.56 to 1303.75) |
| Somalia | 1087.41 | 1343.36 | 916.48 | 1087.41 (916.48 to 1343.36) |
| Antigua and Barbuda | 1090.29 | 1371.84 | 862.04 | 1090.29 (862.04 to 1371.84) |
| Costa Rica | 1099.32 | 1316.68 | 909.14 | 1099.32 (909.14 to 1316.68) |
| Canada | 1103.78 | 1348.06 | 923.5 | 1103.78 (923.5 to 1348.06) |
| Bermuda | 1106.79 | 1409.72 | 887.08 | 1106.79 (887.08 to 1409.72) |
| Bolivia (Plurinational State of) | 1107.57 | 1428.98 | 895.22 | 1107.57 (895.22 to 1428.98) |
| Panama | 1113.32 | 1395.6 | 904.52 | 1113.32 (904.52 to 1395.6) |
| United Arab Emirates | 1127.22 | 1313.8 | 965.14 | 1127.22 (965.14 to 1313.8) |
| Belize | 1129.14 | 1396.59 | 928.23 | 1129.14 (928.23 to 1396.59) |
| Nicaragua | 1131.13 | 1404.61 | 911.4 | 1131.13 (911.4 to 1404.61) |
| United States Virgin Islands | 1143.02 | 1457.9 | 906.26 | 1143.02 (906.26 to 1457.9) |
| Barbados | 1146.07 | 1420.63 | 924.69 | 1146.07 (924.69 to 1420.63) |
| Ecuador | 1166.14 | 1553.66 | 896.54 | 1166.14 (896.54 to 1553.66) |
| Burundi | 1181.08 | 1452.66 | 994.2 | 1181.08 (994.2 to 1452.66) |
| Saint Lucia | 1191.81 | 1453.88 | 985.95 | 1191.81 (985.95 to 1453.88) |
| Cuba | 1228.47 | 1564.82 | 974.01 | 1228.47 (974.01 to 1564.82) |
| Jamaica | 1237.44 | 1520.76 | 1006.82 | 1237.44 (1006.82 to 1520.76) |
| Peru | 1278.86 | 1647.22 | 1024.71 | 1278.86 (1024.71 to 1647.22) |
| Brazil | 1351.58 | 1747.43 | 1071.59 | 1351.58 (1071.59 to 1747.43) |
| Poland | 1355.72 | 1570.95 | 1164.15 | 1355.72 (1164.15 to 1570.95) |
| Grenada | 1429.3 | 1799.53 | 1165.08 | 1429.3 (1165.08 to 1799.53) |
| Greenland | 1434.55 | 1761.61 | 1195.02 | 1434.55 (1195.02 to 1761.61) |
| El Salvador | 1439.6 | 1822.24 | 1145.18 | 1439.6 (1145.18 to 1822.24) |
| Madagascar | 1449.98 | 1761.22 | 1172.14 | 1449.98 (1172.14 to 1761.22) |
| United States of America | 1457.35 | 1911.79 | 1158.29 | 1457.35 (1158.29 to 1911.79) |
| Puerto Rico | 1514.97 | 1898.09 | 1236.59 | 1514.97 (1236.59 to 1898.09) |
| Rwanda | 1580.65 | 1961.15 | 1287.91 | 1580.65 (1287.91 to 1961.15) |
| Haiti | 1788.55 | 2159.51 | 1505.7 | 1788.55 (1505.7 to 2159.51) |

Supplementary Table 11: Global ASIR for asthma in 2021

| **location** | **val** | **upper** | **lower** | **Rate_2021** |
| --- | --- | --- | --- | --- |
| Lesotho | 261.52 | 304.25 | 224.56 | 261.52 (224.56 to 304.25) |
| Pakistan | 278.77 | 347.45 | 227.64 | 278.77 (227.64 to 347.45) |
| Bhutan | 283.35 | 353.21 | 233.61 | 283.35 (233.61 to 353.21) |
| Kazakhstan | 284.4 | 357.91 | 233.75 | 284.4 (233.75 to 357.91) |
| Nepal | 292.65 | 359.34 | 247.93 | 292.65 (247.93 to 359.34) |
| American Samoa | 304.95 | 385.19 | 246.96 | 304.95 (246.96 to 385.19) |
| Bangladesh | 307.88 | 402.6 | 246.97 | 307.88 (246.97 to 402.6) |
| South Africa | 311.37 | 397.5 | 248.33 | 311.37 (248.33 to 397.5) |
| Niue | 314.96 | 388.06 | 261.86 | 314.96 (261.86 to 388.06) |
| Cameroon | 316.79 | 391.44 | 253.01 | 316.79 (253.01 to 391.44) |
| Turkmenistan | 318.89 | 407.45 | 254.13 | 318.89 (254.13 to 407.45) |
| Guam | 321.8 | 429.36 | 249.92 | 321.8 (249.92 to 429.36) |
| Armenia | 322.89 | 419.04 | 250.73 | 322.89 (250.73 to 419.04) |
| Northern Mariana Islands | 322.96 | 416.79 | 254.17 | 322.96 (254.17 to 416.79) |
| Myanmar | 332.72 | 390.58 | 285.5 | 332.72 (285.5 to 390.58) |
| Palau | 334.74 | 427.39 | 272.01 | 334.74 (272.01 to 427.39) |
| Netherlands | 339.04 | 419.64 | 280.69 | 339.04 (280.69 to 419.64) |
| Tokelau | 339.87 | 419.32 | 278.55 | 339.87 (278.55 to 419.32) |
| Maldives | 344.42 | 435.64 | 276.53 | 344.42 (276.53 to 435.64) |
| Tuvalu | 346.88 | 410.88 | 295.35 | 346.88 (295.35 to 410.88) |
| Cook Islands | 358.42 | 476.76 | 280.3 | 358.42 (280.3 to 476.76) |
| Nauru | 359.02 | 417.93 | 307.99 | 359.02 (307.99 to 417.93) |
| Seychelles | 359.11 | 445.47 | 295.52 | 359.11 (295.52 to 445.47) |
| Lao People's Democratic Republic | 360.35 | 429.74 | 308.35 | 360.35 (308.35 to 429.74) |
| Marshall Islands | 363.53 | 424.41 | 314.82 | 363.53 (314.82 to 424.41) |
| China | 364.17 | 494.1 | 283.22 | 364.17 (283.22 to 494.1) |
| Israel | 365.5 | 472.88 | 294.43 | 365.5 (294.43 to 472.88) |
| Samoa | 367.08 | 447.16 | 303.64 | 367.08 (303.64 to 447.16) |
| Ghana | 369.17 | 456.22 | 306.88 | 369.17 (306.88 to 456.22) |
| Georgia | 370.44 | 470.75 | 296.84 | 370.44 (296.84 to 470.75) |
| Cambodia | 373.28 | 451.24 | 313.36 | 373.28 (313.36 to 451.24) |
| Micronesia (Federated States of) | 373.79 | 437.35 | 323.29 | 373.79 (323.29 to 437.35) |
| Zimbabwe | 376.81 | 457.76 | 315.86 | 376.81 (315.86 to 457.76) |
| Mongolia | 377.28 | 467.55 | 307.58 | 377.28 (307.58 to 467.55) |
| India | 378.41 | 469.02 | 313.15 | 378.41 (313.15 to 469.02) |
| Tajikistan | 378.71 | 476.49 | 311.71 | 378.71 (311.71 to 476.49) |
| Azerbaijan | 379.87 | 480.02 | 310.86 | 379.87 (310.86 to 480.02) |
| Mali | 380.86 | 474.09 | 315.48 | 380.86 (315.48 to 474.09) |
| Vanuatu | 381.55 | 440.06 | 334.78 | 381.55 (334.78 to 440.06) |
| Solomon Islands | 387.08 | 450.26 | 335.38 | 387.08 (335.38 to 450.26) |
| Zambia | 387.82 | 471.12 | 318.44 | 387.82 (318.44 to 471.12) |
| Germany | 388.84 | 498.4 | 315.78 | 388.84 (315.78 to 498.4) |
| Senegal | 395.01 | 493.39 | 321.34 | 395.01 (321.34 to 493.39) |
| Indonesia | 400.22 | 480.38 | 339.45 | 400.22 (339.45 to 480.38) |
| Spain | 402.73 | 512.86 | 322.25 | 402.73 (322.25 to 512.86) |
| Belgium | 407.01 | 530.57 | 329.62 | 407.01 (329.62 to 530.57) |
| Greece | 411.54 | 522.67 | 328.35 | 411.54 (328.35 to 522.67) |
| Fiji | 413.52 | 474.4 | 357.76 | 413.52 (357.76 to 474.4) |
| Morocco | 415 | 500.81 | 345.54 | 415 (345.54 to 500.81) |
| Italy | 415.14 | 540.13 | 323.39 | 415.14 (323.39 to 540.13) |
| Austria | 416.63 | 532.73 | 339.86 | 416.63 (339.86 to 532.73) |
| Malaysia | 418.46 | 529.76 | 335.26 | 418.46 (335.26 to 529.76) |
| Cabo Verde | 421.06 | 546.87 | 328.96 | 421.06 (328.96 to 546.87) |
| Kyrgyzstan | 421.49 | 536.07 | 343.37 | 421.49 (343.37 to 536.07) |
| Liberia | 425.01 | 527.38 | 346.95 | 425.01 (346.95 to 527.38) |
| Russian Federation | 431.11 | 565.93 | 337.88 | 431.11 (337.88 to 565.93) |
| Namibia | 433.53 | 529.63 | 354.73 | 433.53 (354.73 to 529.63) |
| Ethiopia | 436.2 | 551.89 | 354.79 | 436.2 (354.79 to 551.89) |
| Chad | 440.57 | 525.69 | 365.19 | 440.57 (365.19 to 525.69) |
| Sudan | 441.82 | 531.86 | 369.78 | 441.82 (369.78 to 531.86) |
| Equatorial Guinea | 450.46 | 565.66 | 367.53 | 450.46 (367.53 to 565.66) |
| Republic of Korea | 451.45 | 574.87 | 361.36 | 451.45 (361.36 to 574.87) |
| Singapore | 453.34 | 591.27 | 356.34 | 453.34 (356.34 to 591.27) |
| Kenya | 456.3 | 569.95 | 371.4 | 456.3 (371.4 to 569.95) |
| Denmark | 460.46 | 582.51 | 371.68 | 460.46 (371.68 to 582.51) |
| Gabon | 462.48 | 581.44 | 373.94 | 462.48 (373.94 to 581.44) |
| Finland | 462.62 | 586.93 | 378.33 | 462.62 (378.33 to 586.93) |
| Estonia | 462.96 | 591.7 | 372.9 | 462.96 (372.9 to 591.7) |
| Republic of Moldova | 464.54 | 598.21 | 366.5 | 464.54 (366.5 to 598.21) |
| Switzerland | 465.75 | 604.17 | 375.8 | 465.75 (375.8 to 604.17) |
| Mexico | 466.58 | 618.79 | 360.69 | 466.58 (360.69 to 618.79) |
| Gambia | 467.58 | 571.9 | 386.03 | 467.58 (386.03 to 571.9) |
| Albania | 471.16 | 589.65 | 382.88 | 471.16 (382.88 to 589.65) |
| Angola | 471.7 | 581.46 | 397.45 | 471.7 (397.45 to 581.46) |
| San Marino | 474.16 | 600.02 | 382.8 | 474.16 (382.8 to 600.02) |
| Monaco | 474.57 | 605.76 | 376.4 | 474.57 (376.4 to 605.76) |
| Lithuania | 476.46 | 609.44 | 381.41 | 476.46 (381.41 to 609.44) |
| Saudi Arabia | 477.24 | 611.37 | 383.88 | 477.24 (383.88 to 611.37) |
| Democratic Republic of the Congo | 478.87 | 574.22 | 398.6 | 478.87 (398.6 to 574.22) |
| France | 484.64 | 611.23 | 392.33 | 484.64 (392.33 to 611.23) |
| Mauritius | 488.52 | 603.08 | 405.68 | 488.52 (405.68 to 603.08) |
| Viet Nam | 489.61 | 602.5 | 406.17 | 489.61 (406.17 to 602.5) |
| Iran (Islamic Republic of) | 492.05 | 614.85 | 401.86 | 492.05 (401.86 to 614.85) |
| Andorra | 493.99 | 626.48 | 401.19 | 493.99 (401.19 to 626.48) |
| Timor-Leste | 497.61 | 585.44 | 426.64 | 497.61 (426.64 to 585.44) |
| Algeria | 498.51 | 615.62 | 411.37 | 498.51 (411.37 to 615.62) |
| Malta | 502.08 | 644.33 | 403.99 | 502.08 (403.99 to 644.33) |
| Egypt | 506.22 | 609.09 | 421.46 | 506.22 (421.46 to 609.09) |
| Latvia | 510.1 | 647.25 | 408.52 | 510.1 (408.52 to 647.25) |
| Thailand | 510.19 | 633.12 | 415.81 | 510.19 (415.81 to 633.12) |
| Benin | 514.33 | 630.32 | 432 | 514.33 (432 to 630.32) |
| Luxembourg | 514.34 | 662.01 | 417.42 | 514.34 (417.42 to 662.01) |
| Papua New Guinea | 514.61 | 587.26 | 451.71 | 514.61 (451.71 to 587.26) |
| Slovakia | 515.62 | 651.95 | 414.65 | 515.62 (414.65 to 651.95) |
| Czechia | 517.41 | 665.98 | 413.27 | 517.41 (413.27 to 665.98) |
| Guatemala | 517.84 | 648.65 | 414.78 | 517.84 (414.78 to 648.65) |
| Iraq | 522.27 | 654.02 | 428.68 | 522.27 (428.68 to 654.02) |
| Cyprus | 524.56 | 661.2 | 417.5 | 524.56 (417.5 to 661.2) |
| Ireland | 525.49 | 670.53 | 417.61 | 525.49 (417.61 to 670.53) |
| Tunisia | 529.5 | 654.79 | 436.9 | 529.5 (436.9 to 654.79) |
| Uzbekistan | 532.75 | 652.67 | 441.48 | 532.75 (441.48 to 652.67) |
| Ukraine | 534.18 | 709.46 | 408.19 | 534.18 (408.19 to 709.46) |
| Guinea-Bissau | 534.53 | 639.21 | 453.83 | 534.53 (453.83 to 639.21) |
| Sri Lanka | 535.17 | 635.17 | 462.82 | 535.17 (462.82 to 635.17) |
| Tonga | 535.22 | 666.13 | 433.07 | 535.22 (433.07 to 666.13) |
| Serbia | 536.9 | 675.93 | 434.49 | 536.9 (434.49 to 675.93) |
| Libya | 536.97 | 659.14 | 452.84 | 536.97 (452.84 to 659.14) |
| Japan | 537.52 | 711.38 | 418.28 | 537.52 (418.28 to 711.38) |
| Bahrain | 537.79 | 673.53 | 437.95 | 537.79 (437.95 to 673.53) |
| Cote d'Ivoire | 542.47 | 665.82 | 445.48 | 542.47 (445.48 to 665.82) |
| Hungary | 544.21 | 696.54 | 437.93 | 544.21 (437.93 to 696.54) |
| Congo | 555.28 | 681.09 | 456.7 | 555.28 (456.7 to 681.09) |
| Australia | 556.15 | 729.6 | 434.5 | 556.15 (434.5 to 729.6) |
| Jordan | 557.19 | 698.51 | 454.37 | 557.19 (454.37 to 698.51) |
| Sierra Leone | 561.18 | 680.08 | 469.78 | 561.18 (469.78 to 680.08) |
| Togo | 567.76 | 724.05 | 451.7 | 567.76 (451.7 to 724.05) |
| Botswana | 568.25 | 692.49 | 466.02 | 568.25 (466.02 to 692.49) |
| Qatar | 568.37 | 713.57 | 460.91 | 568.37 (460.91 to 713.57) |
| Kiribati | 569.81 | 642.05 | 508.13 | 569.81 (508.13 to 642.05) |
| Guinea | 573.06 | 689.83 | 470.54 | 573.06 (470.54 to 689.83) |
| Kuwait | 575.19 | 713.27 | 457.8 | 575.19 (457.8 to 713.27) |
| Democratic People's Republic of Korea | 575.3 | 721.34 | 479.19 | 575.3 (479.19 to 721.34) |
| Niger | 583.22 | 713.74 | 480.17 | 583.22 (480.17 to 713.74) |
| Palestine | 586.1 | 726.27 | 478.16 | 586.1 (478.16 to 726.27) |
| Bulgaria | 587.17 | 744.39 | 470.33 | 587.17 (470.33 to 744.39) |
| Yemen | 587.9 | 698.18 | 506.53 | 587.9 (506.53 to 698.18) |
| Montenegro | 591.64 | 753.53 | 473.4 | 591.64 (473.4 to 753.53) |
| Oman | 594.39 | 740.85 | 481.51 | 594.39 (481.51 to 740.85) |
| Burkina Faso | 603.48 | 752.26 | 493.23 | 603.48 (493.23 to 752.26) |
| Honduras | 604.06 | 748.93 | 497.83 | 604.06 (497.83 to 748.93) |
| Sao Tome and Principe | 604.25 | 732.16 | 509.35 | 604.25 (509.35 to 732.16) |
| Djibouti | 619.74 | 771.02 | 507.64 | 619.74 (507.64 to 771.02) |
| Mozambique | 620.41 | 761.3 | 513.31 | 620.41 (513.31 to 761.3) |
| Uruguay | 621.2 | 799.27 | 491.46 | 621.2 (491.46 to 799.27) |
| Brunei Darussalam | 621.37 | 744.08 | 531.41 | 621.37 (531.41 to 744.08) |
| Portugal | 621.82 | 792.42 | 514.87 | 621.82 (514.87 to 792.42) |
| Belarus | 627.76 | 793.03 | 494.75 | 627.76 (494.75 to 793.03) |
| Iceland | 634.64 | 817.33 | 511.84 | 634.64 (511.84 to 817.33) |
| Croatia | 639.26 | 806.35 | 520.05 | 639.26 (520.05 to 806.35) |
| Colombia | 642.63 | 821.61 | 508.02 | 642.63 (508.02 to 821.61) |
| Norway | 644.29 | 823.58 | 511 | 644.29 (511 to 823.58) |
| Central African Republic | 645.17 | 752.96 | 555.98 | 645.17 (555.98 to 752.96) |
| Afghanistan | 649.85 | 757.78 | 557.66 | 649.85 (557.66 to 757.78) |
| Bolivia (Plurinational State of) | 653.14 | 823.89 | 513.89 | 653.14 (513.89 to 823.89) |
| Syrian Arab Republic | 655.68 | 781.15 | 552.16 | 655.68 (552.16 to 781.15) |
| Argentina | 657.99 | 852.8 | 523.06 | 657.99 (523.06 to 852.8) |
| Ecuador | 666.45 | 876.74 | 503.97 | 666.45 (503.97 to 876.74) |
| Dominican Republic | 666.98 | 815.61 | 554.93 | 666.98 (554.93 to 815.61) |
| Lebanon | 670.53 | 829.09 | 554.84 | 670.53 (554.84 to 829.09) |
| Malawi | 678.16 | 844.22 | 552.51 | 678.16 (552.51 to 844.22) |
| Trinidad and Tobago | 679.47 | 868.6 | 547.24 | 679.47 (547.24 to 868.6) |
| Sweden | 679.64 | 872.59 | 541.37 | 679.64 (541.37 to 872.59) |
| Romania | 699.72 | 881.37 | 560.36 | 699.72 (560.36 to 881.37) |
| Chile | 701.91 | 892.05 | 565.01 | 701.91 (565.01 to 892.05) |
| Eritrea | 706.29 | 846.77 | 595.64 | 706.29 (595.64 to 846.77) |
| Taiwan (Province of China) | 710.21 | 939.34 | 560.2 | 710.21 (560.2 to 939.34) |
| Turkiye | 711.27 | 869.36 | 593.5 | 711.27 (593.5 to 869.36) |
| Nicaragua | 717.65 | 895.19 | 573.92 | 717.65 (573.92 to 895.19) |
| Venezuela (Bolivarian Republic of) | 720.6 | 906.61 | 572.08 | 720.6 (572.08 to 906.61) |
| Comoros | 732.03 | 903.4 | 594.07 | 732.03 (594.07 to 903.4) |
| Mauritania | 734.48 | 928.59 | 585.41 | 734.48 (585.41 to 928.59) |
| New Zealand | 744.86 | 987.23 | 570.25 | 744.86 (570.25 to 987.23) |
| Philippines | 744.95 | 891.77 | 632.85 | 744.95 (632.85 to 891.77) |
| Bosnia and Herzegovina | 746.27 | 943.37 | 605.77 | 746.27 (605.77 to 943.37) |
| South Sudan | 753.8 | 922.4 | 633.42 | 753.8 (633.42 to 922.4) |
| Nigeria | 757 | 937.91 | 627.56 | 757 (627.56 to 937.91) |
| Slovenia | 777.44 | 977.49 | 631.09 | 777.44 (631.09 to 977.49) |
| United Kingdom | 793.69 | 998.97 | 643.07 | 793.69 (643.07 to 998.97) |
| Burundi | 794.26 | 953.81 | 664.98 | 794.26 (664.98 to 953.81) |
| North Macedonia | 796.35 | 986.17 | 663.83 | 796.35 (663.83 to 986.17) |
| Panama | 807.89 | 1017.18 | 648.7 | 807.89 (648.7 to 1017.18) |
| Uganda | 835.6 | 1025.52 | 683.81 | 835.6 (683.81 to 1025.52) |
| Somalia | 839.02 | 1001.41 | 718.73 | 839.02 (718.73 to 1001.41) |
| Eswatini | 842.5 | 1021.57 | 694.95 | 842.5 (694.95 to 1021.57) |
| Peru | 870.54 | 1143.55 | 656.45 | 870.54 (656.45 to 1143.55) |
| Brazil | 892.5 | 1189.9 | 680.92 | 892.5 (680.92 to 1189.9) |
| Suriname | 915.48 | 1151.32 | 733.94 | 915.48 (733.94 to 1151.32) |
| El Salvador | 930.04 | 1176.03 | 728.8 | 930.04 (728.8 to 1176.03) |
| Costa Rica | 930.17 | 1173.41 | 742.78 | 930.17 (742.78 to 1173.41) |
| Guyana | 943.3 | 1159.05 | 767.19 | 943.3 (767.19 to 1159.05) |
| United Arab Emirates | 949.61 | 1114.34 | 797.69 | 949.61 (797.69 to 1114.34) |
| Paraguay | 955.28 | 1228.2 | 738.61 | 955.28 (738.61 to 1228.2) |
| Saint Vincent and the Grenadines | 960.95 | 1220.83 | 776.78 | 960.95 (776.78 to 1220.83) |
| Belize | 977.88 | 1242.16 | 781.83 | 977.88 (781.83 to 1242.16) |
| Canada | 983.59 | 1288.1 | 760.26 | 983.59 (760.26 to 1288.1) |
| Madagascar | 983.85 | 1190.93 | 818.54 | 983.85 (818.54 to 1190.93) |
| United Republic of Tanzania | 987.55 | 1254.28 | 776.52 | 987.55 (776.52 to 1254.28) |
| Saint Kitts and Nevis | 1001.71 | 1276.1 | 793.69 | 1001.71 (793.69 to 1276.1) |
| Bahamas | 1012.47 | 1280.72 | 808.29 | 1012.47 (808.29 to 1280.72) |
| Dominica | 1012.95 | 1277.33 | 824.03 | 1012.95 (824.03 to 1277.33) |
| Antigua and Barbuda | 1020.4 | 1293.8 | 804.74 | 1020.4 (804.74 to 1293.8) |
| Jamaica | 1029.65 | 1282.3 | 823.76 | 1029.65 (823.76 to 1282.3) |
| Saint Lucia | 1056.76 | 1324.93 | 859.09 | 1056.76 (859.09 to 1324.93) |
| Greenland | 1067.74 | 1350.25 | 865.62 | 1067.74 (865.62 to 1350.25) |
| Bermuda | 1139.78 | 1438.4 | 892.46 | 1139.78 (892.46 to 1438.4) |
| United States Virgin Islands | 1148.76 | 1474.09 | 905.18 | 1148.76 (905.18 to 1474.09) |
| Cuba | 1220.75 | 1532.01 | 970.5 | 1220.75 (970.5 to 1532.01) |
| Rwanda | 1236.28 | 1529.96 | 1005.6 | 1236.28 (1005.6 to 1529.96) |
| Barbados | 1251.59 | 1584.49 | 1008.04 | 1251.59 (1008.04 to 1584.49) |
| Grenada | 1344.97 | 1678.37 | 1065.89 | 1344.97 (1065.89 to 1678.37) |
| United States of America | 1448.72 | 1815.96 | 1176.3 | 1448.72 (1176.3 to 1815.96) |
| Puerto Rico | 1463.6 | 1846.82 | 1174.35 | 1463.6 (1174.35 to 1846.82) |
| Poland | 1467.91 | 1879.79 | 1163.39 | 1467.91 (1163.39 to 1879.79) |
| Haiti | 1617.12 | 1937.19 | 1355.96 | 1617.12 (1355.96 to 1937.19) |

Supplementary Table 12: Global EAPCs of incidence for asthma

| **location** | **EAPC** | **UCI** | **LCI** | **EAPC_CI** | **val** |
| --- | --- | --- | --- | --- | --- |
| South Africa | -2.54823 | -2.23247 | -2.86298 | -2.54823% (-2.86298 to -2.23247) | -2.54823 |
| Japan | -2.45473 | -2.11244 | -2.79583 | -2.45473% (-2.79583 to -2.11244) | -2.45473 |
| Guatemala | -2.41075 | -2.17664 | -2.64429 | -2.41075% (-2.64429 to -2.17664) | -2.41075 |
| Tuvalu | -2.36426 | -2.30757 | -2.42092 | -2.36426% (-2.42092 to -2.30757) | -2.36426 |
| Turkmenistan | -2.23353 | -2.11231 | -2.35459 | -2.23353% (-2.35459 to -2.11231) | -2.23353 |
| Russian Federation | -2.04597 | -1.95296 | -2.13889 | -2.04597% (-2.13889 to -1.95296) | -2.04597 |
| Honduras | -2.04026 | -1.95638 | -2.12408 | -2.04026% (-2.12408 to -1.95638) | -2.04026 |
| Maldives | -1.9066 | -1.81898 | -1.99413 | -1.9066% (-1.99413 to -1.81898) | -1.9066 |
| Fiji | -1.86416 | -1.72744 | -2.00069 | -1.86416% (-2.00069 to -1.72744) | -1.86416 |
| Nauru | -1.8557 | -1.60655 | -2.10422 | -1.8557% (-2.10422 to -1.60655) | -1.8557 |
| Ethiopia | -1.84194 | -1.68761 | -1.99602 | -1.84194% (-1.99602 to -1.68761) | -1.84194 |
| Lao People's Democratic Republic | -1.83774 | -1.78186 | -1.8936 | -1.83774% (-1.8936 to -1.78186) | -1.83774 |
| Ecuador | -1.81164 | -1.53747 | -2.08505 | -1.81164% (-2.08505 to -1.53747) | -1.81164 |
| Bolivia (Plurinational State of) | -1.81163 | -1.75149 | -1.87173 | -1.81163% (-1.87173 to -1.75149) | -1.81163 |
| Equatorial Guinea | -1.77874 | -1.69816 | -1.85926 | -1.77874% (-1.85926 to -1.69816) | -1.77874 |
| Angola | -1.7537 | -1.58287 | -1.92422 | -1.7537% (-1.92422 to -1.58287) | -1.7537 |
| Tokelau | -1.66739 | -1.55401 | -1.78064 | -1.66739% (-1.78064 to -1.55401) | -1.66739 |
| Indonesia | -1.66323 | -1.54258 | -1.78373 | -1.66323% (-1.78373 to -1.54258) | -1.66323 |
| Micronesia (Federated States of) | -1.6415 | -1.58146 | -1.70151 | -1.6415% (-1.70151 to -1.58146) | -1.6415 |
| Bangladesh | -1.63828 | -1.56968 | -1.70684 | -1.63828% (-1.70684 to -1.56968) | -1.63828 |
| Myanmar | -1.63736 | -1.55546 | -1.71918 | -1.63736% (-1.71918 to -1.55546) | -1.63736 |
| Sudan | -1.60157 | -1.47105 | -1.73192 | -1.60157% (-1.73192 to -1.47105) | -1.60157 |
| Mexico | -1.57391 | -1.25413 | -1.89265 | -1.57391% (-1.89265 to -1.25413) | -1.57391 |
| Nicaragua | -1.57333 | -1.49564 | -1.65095 | -1.57333% (-1.65095 to -1.49564) | -1.57333 |
| Guam | -1.56189 | -1.20745 | -1.91506 | -1.56189% (-1.91506 to -1.20745) | -1.56189 |
| El Salvador | -1.55175 | -1.46595 | -1.63748 | -1.55175% (-1.63748 to -1.46595) | -1.55175 |
| Afghanistan | -1.54171 | -1.45936 | -1.62399 | -1.54171% (-1.62399 to -1.45936) | -1.54171 |
| Palau | -1.54046 | -1.31314 | -1.76726 | -1.54046% (-1.76726 to -1.31314) | -1.54046 |
| Niue | -1.5388 | -1.34593 | -1.7313 | -1.5388% (-1.7313 to -1.34593) | -1.5388 |
| Vanuatu | -1.53771 | -1.40521 | -1.67003 | -1.53771% (-1.67003 to -1.40521) | -1.53771 |
| Kiribati | -1.49974 | -1.40377 | -1.59561 | -1.49974% (-1.59561 to -1.40377) | -1.49974 |
| Norway | -1.49126 | -1.38197 | -1.60042 | -1.49126% (-1.60042 to -1.38197) | -1.49126 |
| Timor-Leste | -1.46425 | -1.39637 | -1.53207 | -1.46425% (-1.53207 to -1.39637) | -1.46425 |
| Venezuela (Bolivarian Republic of) | -1.46096 | -1.28123 | -1.64035 | -1.46096% (-1.64035 to -1.28123) | -1.46096 |
| Marshall Islands | -1.4593 | -1.30811 | -1.61026 | -1.4593% (-1.61026 to -1.30811) | -1.4593 |
| Brazil | -1.45558 | -1.31943 | -1.59153 | -1.45558% (-1.59153 to -1.31943) | -1.45558 |
| Mauritius | -1.45333 | -1.38401 | -1.52261 | -1.45333% (-1.52261 to -1.38401) | -1.45333 |
| Iran (Islamic Republic of) | -1.45057 | -1.38512 | -1.51597 | -1.45057% (-1.51597 to -1.38512) | -1.45057 |
| Pakistan | -1.41541 | -1.23346 | -1.59702 | -1.41541% (-1.59702 to -1.23346) | -1.41541 |
| Singapore | -1.40666 | -1.20013 | -1.61275 | -1.40666% (-1.61275 to -1.20013) | -1.40666 |
| Turkiye | -1.40535 | -1.23783 | -1.57259 | -1.40535% (-1.57259 to -1.23783) | -1.40535 |
| Papua New Guinea | -1.40402 | -1.3088 | -1.49916 | -1.40402% (-1.49916 to -1.3088) | -1.40402 |
| Australia | -1.39108 | -1.10608 | -1.67525 | -1.39108% (-1.67525 to -1.10608) | -1.39108 |
| Yemen | -1.36277 | -1.33402 | -1.3915 | -1.36277% (-1.3915 to -1.33402) | -1.36277 |
| Burundi | -1.34598 | -1.29802 | -1.39392 | -1.34598% (-1.39392 to -1.29802) | -1.34598 |
| Madagascar | -1.34209 | -1.2886 | -1.39554 | -1.34209% (-1.39554 to -1.2886) | -1.34209 |
| Eritrea | -1.32623 | -1.25792 | -1.39449 | -1.32623% (-1.39449 to -1.25792) | -1.32623 |
| American Samoa | -1.27107 | -1.02403 | -1.51749 | -1.27107% (-1.51749 to -1.02403) | -1.27107 |
| India | -1.26421 | -0.83893 | -1.68767 | -1.26421% (-1.68767 to -0.83893) | -1.26421 |
| Peru | -1.26067 | -1.1298 | -1.39137 | -1.26067% (-1.39137 to -1.1298) | -1.26067 |
| Bhutan | -1.25322 | -1.19336 | -1.31304 | -1.25322% (-1.31304 to -1.19336) | -1.25322 |
| Greenland | -1.24927 | -1.15112 | -1.34731 | -1.24927% (-1.34731 to -1.15112) | -1.24927 |
| Egypt | -1.23719 | -1.16597 | -1.30836 | -1.23719% (-1.30836 to -1.16597) | -1.23719 |
| Northern Mariana Islands | -1.22875 | -0.83136 | -1.62455 | -1.22875% (-1.62455 to -0.83136) | -1.22875 |
| Belarus | -1.22293 | -1.02345 | -1.42201 | -1.22293% (-1.42201 to -1.02345) | -1.22293 |
| Guinea-Bissau | -1.21491 | -1.17563 | -1.25418 | -1.21491% (-1.25418 to -1.17563) | -1.21491 |
| Liberia | -1.2023 | -1.15021 | -1.25435 | -1.2023% (-1.25435 to -1.15021) | -1.2023 |
| Djibouti | -1.20123 | -1.11997 | -1.28242 | -1.20123% (-1.28242 to -1.11997) | -1.20123 |
| China | -1.16966 | -0.77972 | -1.55807 | -1.16966% (-1.55807 to -0.77972) | -1.16966 |
| Ukraine | -1.1568 | -1.1058 | -1.20778 | -1.1568% (-1.20778 to -1.1058) | -1.1568 |
| Sao Tome and Principe | -1.15641 | -1.09517 | -1.21761 | -1.15641% (-1.21761 to -1.09517) | -1.15641 |
| Cambodia | -1.15094 | -1.08101 | -1.22081 | -1.15094% (-1.22081 to -1.08101) | -1.15094 |
| Congo | -1.14874 | -1.04721 | -1.25016 | -1.14874% (-1.25016 to -1.04721) | -1.14874 |
| Solomon Islands | -1.13948 | -1.02595 | -1.25289 | -1.13948% (-1.25289 to -1.02595) | -1.13948 |
| Mozambique | -1.13265 | -1.07638 | -1.18889 | -1.13265% (-1.18889 to -1.07638) | -1.13265 |
| Kenya | -1.13225 | -1.01334 | -1.25101 | -1.13225% (-1.25101 to -1.01334) | -1.13225 |
| Iceland | -1.1236 | -1.04552 | -1.20162 | -1.1236% (-1.20162 to -1.04552) | -1.1236 |
| Colombia | -1.11682 | -0.8195 | -1.41324 | -1.11682% (-1.41324 to -0.8195) | -1.11682 |
| Iraq | -1.08057 | -1.00716 | -1.15394 | -1.08057% (-1.15394 to -1.00716) | -1.08057 |
| Philippines | -1.05802 | -0.95489 | -1.16104 | -1.05802% (-1.16104 to -0.95489) | -1.05802 |
| Republic of Moldova | -1.01956 | -0.98026 | -1.05886 | -1.01956% (-1.05886 to -0.98026) | -1.01956 |
| Panama | -1.01716 | -0.955 | -1.07929 | -1.01716% (-1.07929 to -0.955) | -1.01716 |
| Ireland | -1.01677 | -0.94389 | -1.08959 | -1.01677% (-1.08959 to -0.94389) | -1.01677 |
| South Sudan | -1.01533 | -0.95367 | -1.07695 | -1.01533% (-1.07695 to -0.95367) | -1.01533 |
| Samoa | -1.00351 | -0.94476 | -1.06222 | -1.00351% (-1.06222 to -0.94476) | -1.00351 |
| New Zealand | -1.00347 | -0.76484 | -1.24152 | -1.00347% (-1.24152 to -0.76484) | -1.00347 |
| Bahrain | -0.95986 | -0.87289 | -1.04675 | -0.95986% (-1.04675 to -0.87289) | -0.95986 |
| Luxembourg | -0.9584 | -0.90492 | -1.01186 | -0.9584% (-1.01186 to -0.90492) | -0.9584 |
| Malaysia | -0.95018 | -0.83796 | -1.06228 | -0.95018% (-1.06228 to -0.83796) | -0.95018 |
| Zambia | -0.94233 | -0.86974 | -1.01485 | -0.94233% (-1.01485 to -0.86974) | -0.94233 |
| Rwanda | -0.93779 | -0.88249 | -0.99306 | -0.93779% (-0.99306 to -0.88249) | -0.93779 |
| Kyrgyzstan | -0.93328 | -0.84971 | -1.01678 | -0.93328% (-1.01678 to -0.84971) | -0.93328 |
| Israel | -0.92742 | -0.82696 | -1.02777 | -0.92742% (-1.02777 to -0.82696) | -0.92742 |
| Dominican Republic | -0.91307 | -0.75784 | -1.06805 | -0.91307% (-1.06805 to -0.75784) | -0.91307 |
| Thailand | -0.90998 | -0.75902 | -1.0607 | -0.90998% (-1.0607 to -0.75902) | -0.90998 |
| Senegal | -0.9097 | -0.85171 | -0.96767 | -0.9097% (-0.96767 to -0.85171) | -0.9097 |
| Gabon | -0.90902 | -0.84377 | -0.97423 | -0.90902% (-0.97423 to -0.84377) | -0.90902 |
| Niger | -0.9017 | -0.85937 | -0.944 | -0.9017% (-0.944 to -0.85937) | -0.9017 |
| Mongolia | -0.89661 | -0.85635 | -0.93686 | -0.89661% (-0.93686 to -0.85635) | -0.89661 |
| North Macedonia | -0.89557 | -0.70852 | -1.08226 | -0.89557% (-1.08226 to -0.70852) | -0.89557 |
| Democratic Republic of the Congo | -0.89452 | -0.82171 | -0.96728 | -0.89452% (-0.96728 to -0.82171) | -0.89452 |
| Benin | -0.89178 | -0.84739 | -0.93616 | -0.89178% (-0.93616 to -0.84739) | -0.89178 |
| Germany | -0.8807 | -0.62079 | -1.13993 | -0.8807% (-1.13993 to -0.62079) | -0.8807 |
| Malawi | -0.87817 | -0.82132 | -0.93499 | -0.87817% (-0.93499 to -0.82132) | -0.87817 |
| Croatia | -0.87156 | -0.7007 | -1.04213 | -0.87156% (-1.04213 to -0.7007) | -0.87156 |
| Comoros | -0.87049 | -0.81156 | -0.92938 | -0.87049% (-0.92938 to -0.81156) | -0.87049 |
| United Kingdom | -0.86642 | -0.7282 | -1.00445 | -0.86642% (-1.00445 to -0.7282) | -0.86642 |
| Namibia | -0.86351 | -0.8116 | -0.9154 | -0.86351% (-0.9154 to -0.8116) | -0.86351 |
| Sri Lanka | -0.86151 | -0.77054 | -0.9524 | -0.86151% (-0.9524 to -0.77054) | -0.86151 |
| Italy | -0.85447 | -0.63457 | -1.07388 | -0.85447% (-1.07388 to -0.63457) | -0.85447 |
| Sweden | -0.83839 | -0.41262 | -1.26235 | -0.83839% (-1.26235 to -0.41262) | -0.83839 |
| Somalia | -0.83684 | -0.80008 | -0.8736 | -0.83684% (-0.8736 to -0.80008) | -0.83684 |
| Tonga | -0.82805 | -0.6945 | -0.96143 | -0.82805% (-0.96143 to -0.6945) | -0.82805 |
| Uzbekistan | -0.8187 | -0.74038 | -0.89694 | -0.8187% (-0.89694 to -0.74038) | -0.8187 |
| Uganda | -0.80639 | -0.74796 | -0.8648 | -0.80639% (-0.8648 to -0.74796) | -0.80639 |
| Belgium | -0.79146 | -0.52905 | -1.05318 | -0.79146% (-1.05318 to -0.52905) | -0.79146 |
| France | -0.78681 | -0.66259 | -0.91088 | -0.78681% (-0.91088 to -0.66259) | -0.78681 |
| Democratic People's Republic of Korea | -0.78653 | -0.70515 | -0.86784 | -0.78653% (-0.86784 to -0.70515) | -0.78653 |
| Cabo Verde | -0.7644 | -0.68083 | -0.8479 | -0.7644% (-0.8479 to -0.68083) | -0.7644 |
| Andorra | -0.7407 | -0.67078 | -0.81058 | -0.7407% (-0.81058 to -0.67078) | -0.7407 |
| Cook Islands | -0.73758 | -0.37146 | -1.10236 | -0.73758% (-1.10236 to -0.37146) | -0.73758 |
| Republic of Korea | -0.73411 | -0.67134 | -0.79685 | -0.73411% (-0.79685 to -0.67134) | -0.73411 |
| Nigeria | -0.73402 | -0.67048 | -0.79752 | -0.73402% (-0.79752 to -0.67048) | -0.73402 |
| Gambia | -0.73136 | -0.66507 | -0.7976 | -0.73136% (-0.7976 to -0.66507) | -0.73136 |
| Guinea | -0.72829 | -0.67913 | -0.77742 | -0.72829% (-0.77742 to -0.67913) | -0.72829 |
| Switzerland | -0.71761 | -0.67376 | -0.76144 | -0.71761% (-0.76144 to -0.67376) | -0.71761 |
| Ghana | -0.71447 | -0.66792 | -0.761 | -0.71447% (-0.761 to -0.66792) | -0.71447 |
| Estonia | -0.71224 | -0.6297 | -0.7947 | -0.71224% (-0.7947 to -0.6297) | -0.71224 |
| Costa Rica | -0.70771 | -0.55949 | -0.8557 | -0.70771% (-0.8557 to -0.55949) | -0.70771 |
| Cameroon | -0.69905 | -0.63617 | -0.7619 | -0.69905% (-0.7619 to -0.63617) | -0.69905 |
| Viet Nam | -0.69879 | -0.51722 | -0.88002 | -0.69879% (-0.88002 to -0.51722) | -0.69879 |
| Sierra Leone | -0.68439 | -0.64782 | -0.72095 | -0.68439% (-0.72095 to -0.64782) | -0.68439 |
| Denmark | -0.6652 | -0.55629 | -0.77399 | -0.6652% (-0.77399 to -0.55629) | -0.6652 |
| Seychelles | -0.66494 | -0.53836 | -0.79137 | -0.66494% (-0.79137 to -0.53836) | -0.66494 |
| Eswatini | -0.66259 | -0.58598 | -0.73915 | -0.66259% (-0.73915 to -0.58598) | -0.66259 |
| Mauritania | -0.66206 | -0.61898 | -0.70513 | -0.66206% (-0.70513 to -0.61898) | -0.66206 |
| Nepal | -0.6587 | -0.62289 | -0.69449 | -0.6587% (-0.69449 to -0.62289) | -0.6587 |
| Azerbaijan | -0.64465 | -0.60487 | -0.68442 | -0.64465% (-0.68442 to -0.60487) | -0.64465 |
| Latvia | -0.6427 | -0.54288 | -0.74242 | -0.6427% (-0.74242 to -0.54288) | -0.6427 |
| Portugal | -0.63464 | -0.39377 | -0.87493 | -0.63464% (-0.87493 to -0.39377) | -0.63464 |
| Malta | -0.6309 | -0.54801 | -0.71372 | -0.6309% (-0.71372 to -0.54801) | -0.6309 |
| Austria | -0.62855 | -0.49893 | -0.75799 | -0.62855% (-0.75799 to -0.49893) | -0.62855 |
| Bulgaria | -0.62771 | -0.52651 | -0.7288 | -0.62771% (-0.7288 to -0.52651) | -0.62771 |
| Central African Republic | -0.6274 | -0.5483 | -0.70644 | -0.6274% (-0.70644 to -0.5483) | -0.6274 |
| Lesotho | -0.62135 | -0.56608 | -0.6766 | -0.62135% (-0.6766 to -0.56608) | -0.62135 |
| Netherlands | -0.62026 | -0.4493 | -0.79093 | -0.62026% (-0.79093 to -0.4493) | -0.62026 |
| Jamaica | -0.61701 | -0.51006 | -0.72384 | -0.61701% (-0.72384 to -0.51006) | -0.61701 |
| Tajikistan | -0.61102 | -0.57855 | -0.64347 | -0.61102% (-0.64347 to -0.57855) | -0.61102 |
| Libya | -0.60791 | -0.54688 | -0.6689 | -0.60791% (-0.6689 to -0.54688) | -0.60791 |
| Slovenia | -0.5924 | -0.55827 | -0.62653 | -0.5924% (-0.62653 to -0.55827) | -0.5924 |
| Uruguay | -0.5882 | -0.55164 | -0.62476 | -0.5882% (-0.62476 to -0.55164) | -0.5882 |
| Qatar | -0.57498 | -0.51751 | -0.63242 | -0.57498% (-0.63242 to -0.51751) | -0.57498 |
| Tunisia | -0.5637 | -0.48644 | -0.64091 | -0.5637% (-0.64091 to -0.48644) | -0.5637 |
| United Arab Emirates | -0.56327 | -0.47498 | -0.65148 | -0.56327% (-0.65148 to -0.47498) | -0.56327 |
| Serbia | -0.54426 | -0.40768 | -0.68066 | -0.54426% (-0.68066 to -0.40768) | -0.54426 |
| Cote d'Ivoire | -0.52475 | -0.43165 | -0.61776 | -0.52475% (-0.61776 to -0.43165) | -0.52475 |
| Mali | -0.52068 | -0.46719 | -0.57413 | -0.52068% (-0.57413 to -0.46719) | -0.52068 |
| Hungary | -0.47822 | -0.30338 | -0.65275 | -0.47822% (-0.65275 to -0.30338) | -0.47822 |
| Belize | -0.47555 | -0.37962 | -0.57139 | -0.47555% (-0.57139 to -0.37962) | -0.47555 |
| Jordan | -0.47212 | -0.35649 | -0.58763 | -0.47212% (-0.58763 to -0.35649) | -0.47212 |
| Saint Lucia | -0.46262 | -0.34006 | -0.58502 | -0.46262% (-0.58502 to -0.34006) | -0.46262 |
| Botswana | -0.44956 | -0.37636 | -0.52271 | -0.44956% (-0.52271 to -0.37636) | -0.44956 |
| Kuwait | -0.44816 | -0.35023 | -0.546 | -0.44816% (-0.546 to -0.35023) | -0.44816 |
| Algeria | -0.42969 | -0.37577 | -0.48358 | -0.42969% (-0.48358 to -0.37577) | -0.42969 |
| Lebanon | -0.42296 | -0.38372 | -0.4622 | -0.42296% (-0.4622 to -0.38372) | -0.42296 |
| Chad | -0.4199 | -0.38705 | -0.45274 | -0.4199% (-0.45274 to -0.38705) | -0.4199 |
| Togo | -0.40587 | -0.3424 | -0.46929 | -0.40587% (-0.46929 to -0.3424) | -0.40587 |
| Morocco | -0.40573 | -0.32876 | -0.48265 | -0.40573% (-0.48265 to -0.32876) | -0.40573 |
| Haiti | -0.3589 | -0.32624 | -0.39155 | -0.3589% (-0.39155 to -0.32624) | -0.3589 |
| Georgia | -0.34706 | -0.21487 | -0.47907 | -0.34706% (-0.47907 to -0.21487) | -0.34706 |
| Trinidad and Tobago | -0.33348 | 0.139 | -0.80372 | -0.33348% (-0.80372 to 0.139) | -0.33348 |
| Romania | -0.32356 | -0.18203 | -0.46489 | -0.32356% (-0.46489 to -0.18203) | -0.32356 |
| Canada | -0.3183 | -0.18357 | -0.45285 | -0.3183% (-0.45285 to -0.18357) | -0.3183 |
| Zimbabwe | -0.31339 | -0.24867 | -0.37807 | -0.31339% (-0.37807 to -0.24867) | -0.31339 |
| Guyana | -0.31135 | -0.17769 | -0.44484 | -0.31135% (-0.44484 to -0.17769) | -0.31135 |
| Greece | -0.31082 | -0.21033 | -0.41121 | -0.31082% (-0.41121 to -0.21033) | -0.31082 |
| Antigua and Barbuda | -0.29221 | -0.12559 | -0.45855 | -0.29221% (-0.45855 to -0.12559) | -0.29221 |
| Burkina Faso | -0.2903 | -0.25562 | -0.32497 | -0.2903% (-0.32497 to -0.25562) | -0.2903 |
| Czechia | -0.28078 | -0.22598 | -0.33555 | -0.28078% (-0.33555 to -0.22598) | -0.28078 |
| Argentina | -0.25268 | -0.16879 | -0.3365 | -0.25268% (-0.3365 to -0.16879) | -0.25268 |
| Armenia | -0.24996 | -0.07458 | -0.42504 | -0.24996% (-0.42504 to -0.07458) | -0.24996 |
| Palestine | -0.24583 | -0.13721 | -0.35432 | -0.24583% (-0.35432 to -0.13721) | -0.24583 |
| Grenada | -0.2264 | -0.15637 | -0.29637 | -0.2264% (-0.29637 to -0.15637) | -0.2264 |
| Puerto Rico | -0.218 | -0.10061 | -0.33525 | -0.218% (-0.33525 to -0.10061) | -0.218 |
| Finland | -0.21621 | -0.13351 | -0.29885 | -0.21621% (-0.29885 to -0.13351) | -0.21621 |
| Cyprus | -0.21519 | -0.11734 | -0.31295 | -0.21519% (-0.31295 to -0.11734) | -0.21519 |
| Brunei Darussalam | -0.21482 | -0.11098 | -0.31855 | -0.21482% (-0.31855 to -0.11098) | -0.21482 |
| Slovakia | -0.21047 | -0.17042 | -0.25051 | -0.21047% (-0.25051 to -0.17042) | -0.21047 |
| Monaco | -0.20153 | -0.17364 | -0.2294 | -0.20153% (-0.2294 to -0.17364) | -0.20153 |
| United Republic of Tanzania | -0.18547 | -0.12245 | -0.24845 | -0.18547% (-0.24845 to -0.12245) | -0.18547 |
| Kazakhstan | -0.17997 | -0.10913 | -0.25076 | -0.17997% (-0.25076 to -0.10913) | -0.17997 |
| Dominica | -0.17823 | 0.00188 | -0.35801 | -0.17823% (-0.35801 to 0.00188) | -0.17823 |
| Suriname | -0.17756 | -0.04108 | -0.31386 | -0.17756% (-0.31386 to -0.04108) | -0.17756 |
| Bosnia and Herzegovina | -0.16285 | -0.12392 | -0.20176 | -0.16285% (-0.20176 to -0.12392) | -0.16285 |
| Saint Vincent and the Grenadines | -0.12668 | 0.03128 | -0.28439 | -0.12668% (-0.28439 to 0.03128) | -0.12668 |
| San Marino | -0.1021 | -0.07602 | -0.12818 | -0.1021% (-0.12818 to -0.07602) | -0.1021 |
| Albania | -0.08908 | 0.037 | -0.215 | -0.08908% (-0.215 to 0.037) | -0.08908 |
| Lithuania | -0.08253 | 0.03773 | -0.20263 | -0.08253% (-0.20263 to 0.03773) | -0.08253 |
| Chile | -0.04957 | 0.02924 | -0.12832 | -0.04957% (-0.12832 to 0.02924) | -0.04957 |
| Montenegro | -0.04544 | 0.00341 | -0.09428 | -0.04544% (-0.09428 to 0.00341) | -0.04544 |
| United States Virgin Islands | -0.04082 | 0.06417 | -0.1457 | -0.04082% (-0.1457 to 0.06417) | -0.04082 |
| Bahamas | -0.0237 | 0.16589 | -0.21294 | -0.0237% (-0.21294 to 0.16589) | -0.0237 |
| Taiwan (Province of China) | -0.01959 | 0.26943 | -0.30779 | -0.01959% (-0.30779 to 0.26943) | -0.01959 |
| Saint Kitts and Nevis | -0.00241 | 0.18496 | -0.18944 | -0.00241% (-0.18944 to 0.18496) | -0.00241 |
| Cuba | 0.01477 | 0.12691 | -0.09725 | 0.01477% (-0.09725 to 0.12691) | 0.01477 |
| Bermuda | 0.01916 | 0.18228 | -0.1437 | 0.01916% (-0.1437 to 0.18228) | 0.01916 |
| Paraguay | 0.03356 | 0.09986 | -0.03269 | 0.03356% (-0.03269 to 0.09986) | 0.03356 |
| Spain | 0.19384 | 0.37623 | 0.01179 | 0.19384% (0.01179 to 0.37623) | 0.19384 |
| Saudi Arabia | 0.26354 | 0.38565 | 0.14157 | 0.26354% (0.14157 to 0.38565) | 0.26354 |
| Poland | 0.28689 | 0.71401 | -0.13841 | 0.28689% (-0.13841 to 0.71401) | 0.28689 |
| Syrian Arab Republic | 0.35611 | 0.62173 | 0.09119 | 0.35611% (0.09119 to 0.62173) | 0.35611 |
| Barbados | 0.43869 | 0.57492 | 0.30264 | 0.43869% (0.30264 to 0.57492) | 0.43869 |
| Oman | 0.48423 | 0.6085 | 0.3601 | 0.48423% (0.3601 to 0.6085) | 0.48423 |
| United States of America | 0.72996 | 1.05646 | 0.40451 | 0.72996% (0.40451 to 1.05646) | 0.72996 |

Supplementary Table 13: Global ASYR for asthma in 1990

| **location** | **val** | **upper** | **lower** | **Rate_1990** |
| --- | --- | --- | --- | --- |
| Lesotho | 65.19 | 96.98 | 42.56 | 65.19 (42.56 to 96.98) |
| Kazakhstan | 68.69 | 101.91 | 43.6 | 68.69 (43.6 to 101.91) |
| Armenia | 82.37 | 123.26 | 50.92 | 82.37 (50.92 to 123.26) |
| Cameroon | 85.53 | 127.91 | 54.13 | 85.53 (54.13 to 127.91) |
| Nepal | 87.4 | 129.61 | 56.9 | 87.4 (56.9 to 129.61) |
| Zimbabwe | 92.25 | 139.18 | 58.67 | 92.25 (58.67 to 139.18) |
| Ghana | 98.72 | 149.83 | 63.74 | 98.72 (63.74 to 149.83) |
| Mali | 101.47 | 149.81 | 64.18 | 101.47 (64.18 to 149.81) |
| Seychelles | 102.5 | 156.12 | 65.12 | 102.5 (65.12 to 156.12) |
| Georgia | 102.74 | 152.37 | 64.27 | 102.74 (64.27 to 152.37) |
| Pakistan | 103.31 | 153.29 | 66.14 | 103.31 (66.14 to 153.29) |
| American Samoa | 104.14 | 156.28 | 67.57 | 104.14 (67.57 to 156.28) |
| Cook Islands | 104.6 | 158.22 | 66.47 | 104.6 (66.47 to 158.22) |
| Northern Mariana Islands | 105.04 | 158.21 | 67.04 | 105.04 (67.04 to 158.21) |
| Bhutan | 105.46 | 156.3 | 67.81 | 105.46 (67.81 to 156.3) |
| Zambia | 106.07 | 157.32 | 67.16 | 106.07 (67.16 to 157.32) |
| Chad | 109.32 | 163.69 | 70.14 | 109.32 (70.14 to 163.69) |
| Saudi Arabia | 110.32 | 160.43 | 69.56 | 110.32 (69.56 to 160.43) |
| Morocco | 110.57 | 161.15 | 71.06 | 110.57 (71.06 to 161.15) |
| Niue | 114.16 | 168.06 | 74.54 | 114.16 (74.54 to 168.06) |
| Senegal | 115.21 | 175.29 | 74.19 | 115.21 (74.19 to 175.29) |
| Tajikistan | 115.57 | 169.17 | 74.48 | 115.57 (74.48 to 169.17) |
| Guam | 117.46 | 178.37 | 72.23 | 117.46 (72.23 to 178.37) |
| Myanmar | 117.52 | 172.22 | 75.1 | 117.52 (75.1 to 172.22) |
| Azerbaijan | 117.97 | 174.13 | 75.7 | 117.97 (75.7 to 174.13) |
| China | 121.82 | 182.02 | 77.1 | 121.82 (77.1 to 182.02) |
| Albania | 123.16 | 179.61 | 77.4 | 123.16 (77.4 to 179.61) |
| Mongolia | 123.49 | 184.27 | 78.8 | 123.49 (78.8 to 184.27) |
| Samoa | 123.62 | 184.1 | 77.89 | 123.62 (77.89 to 184.1) |
| Cambodia | 124.18 | 183 | 79.26 | 124.18 (79.26 to 183) |
| Liberia | 128.29 | 190.31 | 81.28 | 128.29 (81.28 to 190.31) |
| Palau | 130.06 | 194.07 | 83.36 | 130.06 (83.36 to 194.07) |
| Bangladesh | 132.97 | 196.67 | 84.96 | 132.97 (84.96 to 196.67) |
| Namibia | 133.06 | 199.85 | 86.19 | 133.06 (86.19 to 199.85) |
| South Africa | 133.73 | 201.11 | 85.22 | 133.73 (85.22 to 201.11) |
| Gambia | 134.28 | 202.93 | 85.84 | 134.28 (85.84 to 202.93) |
| Oman | 136.75 | 200.33 | 85.87 | 136.75 (85.87 to 200.33) |
| Slovakia | 136.79 | 205.08 | 84.79 | 136.79 (84.79 to 205.08) |
| Tokelau | 140.13 | 205.57 | 90.27 | 140.13 (90.27 to 205.57) |
| Cabo Verde | 141.32 | 213.69 | 91.25 | 141.32 (91.25 to 213.69) |
| Czechia | 141.5 | 207.8 | 87.66 | 141.5 (87.66 to 207.8) |
| Solomon Islands | 142.08 | 205.58 | 92.69 | 142.08 (92.69 to 205.58) |
| Lao People's Democratic Republic | 142.33 | 209.12 | 90.8 | 142.33 (90.8 to 209.12) |
| Marshall Islands | 142.65 | 208.55 | 93.92 | 142.65 (93.92 to 208.55) |
| Democratic Republic of the Congo | 142.92 | 213.07 | 90.27 | 142.92 (90.27 to 213.07) |
| Lithuania | 143.43 | 210.91 | 90.21 | 143.43 (90.21 to 210.91) |
| Kenya | 143.9 | 217.79 | 91.82 | 143.9 (91.82 to 217.79) |
| Algeria | 146.11 | 220.9 | 94.28 | 146.11 (94.28 to 220.9) |
| Viet Nam | 147.63 | 220.12 | 93.66 | 147.63 (93.66 to 220.12) |
| Maldives | 148.67 | 218.67 | 93.95 | 148.67 (93.95 to 218.67) |
| Nauru | 149.45 | 216.37 | 97.45 | 149.45 (97.45 to 216.37) |
| Montenegro | 150.07 | 227.62 | 92.52 | 150.07 (92.52 to 227.62) |
| Vanuatu | 152.86 | 224.03 | 99.6 | 152.86 (99.6 to 224.03) |
| Estonia | 153.51 | 223.23 | 99.33 | 153.51 (99.33 to 223.23) |
| Gabon | 153.77 | 226.9 | 98 | 153.77 (98 to 226.9) |
| Cote d'Ivoire | 154.89 | 234.37 | 100.5 | 154.89 (100.5 to 234.37) |
| Burkina Faso | 155.76 | 229.46 | 100.38 | 155.76 (100.38 to 229.46) |
| Togo | 156.63 | 238.77 | 99.38 | 156.63 (99.38 to 238.77) |
| Tunisia | 159.5 | 231.45 | 100.53 | 159.5 (100.53 to 231.45) |
| Sierra Leone | 159.89 | 237.31 | 103.32 | 159.89 (103.32 to 237.31) |
| Benin | 160.1 | 238.51 | 101.92 | 160.1 (101.92 to 238.51) |
| Qatar | 160.15 | 238.78 | 101.67 | 160.15 (101.67 to 238.78) |
| Kyrgyzstan | 160.43 | 234.72 | 102.18 | 160.43 (102.18 to 234.72) |
| Micronesia (Federated States of) | 162.13 | 235.18 | 105.81 | 162.13 (105.81 to 235.18) |
| Malaysia | 162.82 | 243.05 | 105.45 | 162.82 (105.45 to 243.05) |
| Syrian Arab Republic | 163.5 | 237.02 | 103.57 | 163.5 (103.57 to 237.02) |
| India | 164.14 | 252.65 | 106.78 | 164.14 (106.78 to 252.65) |
| Indonesia | 166.42 | 247.59 | 106.06 | 166.42 (106.06 to 247.59) |
| Serbia | 166.95 | 242.71 | 106.37 | 166.95 (106.37 to 242.71) |
| Guinea | 169.11 | 256.84 | 107.42 | 169.11 (107.42 to 256.84) |
| Fiji | 170.86 | 247.14 | 110.97 | 170.86 (110.97 to 247.14) |
| Jordan | 173.11 | 254.17 | 110.45 | 173.11 (110.45 to 254.17) |
| Ethiopia | 174.27 | 258.31 | 112.09 | 174.27 (112.09 to 258.31) |
| Botswana | 174.51 | 267.31 | 113.92 | 174.51 (113.92 to 267.31) |
| Libya | 176.02 | 255.84 | 111.82 | 176.02 (111.82 to 255.84) |
| Hungary | 176.04 | 260.19 | 110.34 | 176.04 (110.34 to 260.19) |
| Republic of Moldova | 176.26 | 260.28 | 111.93 | 176.26 (111.93 to 260.28) |
| Sudan | 176.3 | 263.72 | 111.2 | 176.3 (111.2 to 263.72) |
| Tonga | 177.94 | 265.8 | 113 | 177.94 (113 to 265.8) |
| Turkmenistan | 178.27 | 267.52 | 113.87 | 178.27 (113.87 to 267.52) |
| Trinidad and Tobago | 178.58 | 273.04 | 110.33 | 178.58 (110.33 to 273.04) |
| Thailand | 179.53 | 260.03 | 114.16 | 179.53 (114.16 to 260.03) |
| Tuvalu | 179.72 | 256.59 | 116.76 | 179.72 (116.76 to 256.59) |
| Kuwait | 179.93 | 265.22 | 113.16 | 179.93 (113.16 to 265.22) |
| Mexico | 180.84 | 269.25 | 114.61 | 180.84 (114.61 to 269.25) |
| Niger | 181.43 | 267.22 | 116.39 | 181.43 (116.39 to 267.22) |
| Republic of Korea | 181.82 | 262.76 | 117.01 | 181.82 (117.01 to 262.76) |
| Equatorial Guinea | 183.88 | 271.71 | 120.43 | 183.88 (120.43 to 271.71) |
| Sri Lanka | 185.27 | 265.86 | 120.87 | 185.27 (120.87 to 265.86) |
| Palestine | 186.08 | 271.31 | 116.81 | 186.08 (116.81 to 271.31) |
| Egypt | 187.97 | 278.63 | 118.88 | 187.97 (118.88 to 278.63) |
| Angola | 189.34 | 283.05 | 122.96 | 189.34 (122.96 to 283.05) |
| Timor-Leste | 191.6 | 273.98 | 120.65 | 191.6 (120.65 to 273.98) |
| Bahrain | 192.47 | 283.74 | 123.36 | 192.47 (123.36 to 283.74) |
| Latvia | 193.55 | 288.31 | 121.63 | 193.55 (121.63 to 288.31) |
| Ukraine | 193.86 | 290.82 | 121.62 | 193.86 (121.62 to 290.82) |
| Papua New Guinea | 194.47 | 278.13 | 125.96 | 194.47 (125.96 to 278.13) |
| Iran (Islamic Republic of) | 195.46 | 285.14 | 124.24 | 195.46 (124.24 to 285.14) |
| Mozambique | 195.94 | 293.62 | 123.49 | 195.94 (123.49 to 293.62) |
| Iraq | 195.97 | 288.09 | 126.55 | 195.97 (126.55 to 288.09) |
| Guinea-Bissau | 196.88 | 290.32 | 128.79 | 196.88 (128.79 to 290.32) |
| Mauritius | 199.94 | 296.97 | 125.79 | 199.94 (125.79 to 296.97) |
| Congo | 200.27 | 303.78 | 127.51 | 200.27 (127.51 to 303.78) |
| Central African Republic | 200.94 | 298.74 | 131.05 | 200.94 (131.05 to 298.74) |
| Bulgaria | 201.42 | 299.14 | 127.57 | 201.42 (127.57 to 299.14) |
| Malawi | 207.41 | 309.67 | 130.84 | 207.41 (130.84 to 309.67) |
| Uzbekistan | 209.47 | 306.87 | 134.91 | 209.47 (134.91 to 306.87) |
| Djibouti | 209.86 | 313.22 | 132.51 | 209.86 (132.51 to 313.22) |
| Dominican Republic | 210.03 | 313.14 | 129.86 | 210.03 (129.86 to 313.14) |
| Sao Tome and Principe | 210.98 | 306.24 | 133.73 | 210.98 (133.73 to 306.24) |
| Lebanon | 215.57 | 312.38 | 139.31 | 215.57 (139.31 to 312.38) |
| Russian Federation | 216.46 | 314.07 | 138.34 | 216.46 (138.34 to 314.07) |
| Democratic People's Republic of Korea | 216.96 | 323.78 | 139.04 | 216.96 (139.04 to 323.78) |
| Spain | 218.2 | 331.02 | 136.53 | 218.2 (136.53 to 331.02) |
| Bosnia and Herzegovina | 220.61 | 319.16 | 138.72 | 220.61 (138.72 to 319.16) |
| Paraguay | 222.81 | 330.94 | 139.34 | 222.81 (139.34 to 330.94) |
| Taiwan (Province of China) | 229.67 | 339.63 | 142.06 | 229.67 (142.06 to 339.63) |
| Colombia | 230.1 | 348.04 | 144.34 | 230.1 (144.34 to 348.04) |
| Nigeria | 230.51 | 345.91 | 150.95 | 230.51 (150.95 to 345.91) |
| Suriname | 232.95 | 349.03 | 144.95 | 232.95 (144.95 to 349.03) |
| Romania | 233.82 | 343.48 | 146.44 | 233.82 (146.44 to 343.48) |
| Brunei Darussalam | 235.68 | 353.12 | 149.75 | 235.68 (149.75 to 353.12) |
| Saint Kitts and Nevis | 238.54 | 359.29 | 149.62 | 238.54 (149.62 to 359.29) |
| Comoros | 238.91 | 357.67 | 151.6 | 238.91 (151.6 to 357.67) |
| Kiribati | 239.73 | 342.12 | 154.18 | 239.73 (154.18 to 342.12) |
| Croatia | 240.97 | 348.67 | 152.89 | 240.97 (152.89 to 348.67) |
| Saint Vincent and the Grenadines | 242.55 | 366.17 | 150.42 | 242.55 (150.42 to 366.17) |
| Israel | 246.94 | 362.97 | 157.19 | 246.94 (157.19 to 362.97) |
| Guyana | 249.21 | 377.32 | 156.98 | 249.21 (156.98 to 377.32) |
| Bahamas | 250.21 | 377.15 | 153.59 | 250.21 (153.59 to 377.15) |
| South Sudan | 251.47 | 375.35 | 158.65 | 251.47 (158.65 to 375.35) |
| Bolivia (Plurinational State of) | 252.34 | 377.9 | 155.71 | 252.34 (155.71 to 377.9) |
| Antigua and Barbuda | 255.08 | 393.56 | 158.68 | 255.08 (158.68 to 393.56) |
| Guatemala | 255.87 | 386.27 | 155.2 | 255.87 (155.2 to 386.27) |
| Honduras | 260.81 | 385.95 | 163.36 | 260.81 (163.36 to 385.95) |
| Greece | 261.39 | 388.66 | 160.89 | 261.39 (160.89 to 388.66) |
| Venezuela (Bolivarian Republic of) | 263.47 | 404.1 | 163.12 | 263.47 (163.12 to 404.1) |
| Mauritania | 264.53 | 394.87 | 171.83 | 264.53 (171.83 to 394.87) |
| Italy | 265.54 | 387.16 | 168.83 | 265.54 (168.83 to 387.16) |
| Ecuador | 265.59 | 398.28 | 156.28 | 265.59 (156.28 to 398.28) |
| Yemen | 265.86 | 383.63 | 171.49 | 265.86 (171.49 to 383.63) |
| San Marino | 266.59 | 399.69 | 167.05 | 266.59 (167.05 to 399.69) |
| Bermuda | 266.64 | 399.32 | 165.97 | 266.64 (165.97 to 399.32) |
| Dominica | 268.18 | 407.56 | 168.39 | 268.18 (168.39 to 407.56) |
| Belize | 271.75 | 407.69 | 169.96 | 271.75 (169.96 to 407.69) |
| United Republic of Tanzania | 272.09 | 412.91 | 168.19 | 272.09 (168.19 to 412.91) |
| Eritrea | 273.48 | 403.24 | 175.47 | 273.48 (175.47 to 403.24) |
| Chile | 273.86 | 395.56 | 178.38 | 273.86 (178.38 to 395.56) |
| Monaco | 276.69 | 415.05 | 172.65 | 276.69 (172.65 to 415.05) |
| Uganda | 277.37 | 418.42 | 175.08 | 277.37 (175.08 to 418.42) |
| Uruguay | 280.44 | 414.48 | 182.13 | 280.44 (182.13 to 414.48) |
| Denmark | 281.15 | 412.93 | 179.74 | 281.15 (179.74 to 412.93) |
| Nicaragua | 282.58 | 421.03 | 172.2 | 282.58 (172.2 to 421.03) |
| Philippines | 282.63 | 416.94 | 181.3 | 282.63 (181.3 to 416.94) |
| Panama | 283.21 | 421.09 | 174.78 | 283.21 (174.78 to 421.09) |
| United States Virgin Islands | 286.25 | 436.46 | 177.61 | 286.25 (177.61 to 436.46) |
| Belarus | 288.83 | 419.3 | 184.29 | 288.83 (184.29 to 419.3) |
| Slovenia | 291.89 | 422.04 | 186.04 | 291.89 (186.04 to 422.04) |
| Argentina | 294.1 | 427.01 | 189.86 | 294.1 (189.86 to 427.01) |
| Somalia | 297.39 | 442.41 | 190.07 | 297.39 (190.07 to 442.41) |
| Singapore | 300.73 | 441.98 | 194.81 | 300.73 (194.81 to 441.98) |
| Finland | 300.76 | 448 | 193.09 | 300.76 (193.09 to 448) |
| Canada | 304.59 | 451.3 | 192.68 | 304.59 (192.68 to 451.3) |
| Eswatini | 306.74 | 460.76 | 200.97 | 306.74 (200.97 to 460.76) |
| Turkiye | 306.9 | 450.57 | 193.56 | 306.9 (193.56 to 450.57) |
| Jamaica | 308.67 | 463.83 | 193.66 | 308.67 (193.66 to 463.83) |
| Austria | 311.28 | 468.26 | 195.42 | 311.28 (195.42 to 468.26) |
| Afghanistan | 314.53 | 452.29 | 202.74 | 314.53 (202.74 to 452.29) |
| Brazil | 314.98 | 477.63 | 194.98 | 314.98 (194.98 to 477.63) |
| Barbados | 315.36 | 482.05 | 195.18 | 315.36 (195.18 to 482.05) |
| Peru | 315.74 | 473.36 | 194.88 | 315.74 (194.88 to 473.36) |
| Costa Rica | 318.03 | 473.17 | 203.89 | 318.03 (203.89 to 473.17) |
| Germany | 328.71 | 492.92 | 209.82 | 328.71 (209.82 to 492.92) |
| North Macedonia | 329.26 | 471.67 | 211.11 | 329.26 (211.11 to 471.67) |
| Saint Lucia | 330.05 | 482.65 | 206.78 | 330.05 (206.78 to 482.65) |
| Burundi | 331.83 | 493.23 | 213.31 | 331.83 (213.31 to 493.23) |
| Cuba | 332.54 | 502.98 | 205.85 | 332.54 (205.85 to 502.98) |
| Switzerland | 336.41 | 505.11 | 214.52 | 336.41 (214.52 to 505.11) |
| Belgium | 341.04 | 511.05 | 215.27 | 341.04 (215.27 to 511.05) |
| Cyprus | 362.88 | 543.17 | 224.64 | 362.88 (224.64 to 543.17) |
| Japan | 376.62 | 552.57 | 241.01 | 376.62 (241.01 to 552.57) |
| Andorra | 382.9 | 563.67 | 240.91 | 382.9 (240.91 to 563.67) |
| United States of America | 384.62 | 573.85 | 246.72 | 384.62 (246.72 to 573.85) |
| Malta | 385.3 | 568.77 | 244.98 | 385.3 (244.98 to 568.77) |
| Netherlands | 391.47 | 584.74 | 245.98 | 391.47 (245.98 to 584.74) |
| France | 401.1 | 598.06 | 256.31 | 401.1 (256.31 to 598.06) |
| Grenada | 404.39 | 614.3 | 251.28 | 404.39 (251.28 to 614.3) |
| El Salvador | 419.97 | 634.62 | 261.15 | 419.97 (261.15 to 634.62) |
| United Arab Emirates | 428.91 | 627.37 | 278.69 | 428.91 (278.69 to 627.37) |
| Luxembourg | 458.21 | 692.15 | 288.02 | 458.21 (288.02 to 692.15) |
| Iceland | 460.77 | 690.51 | 294.22 | 460.77 (294.22 to 690.51) |
| Australia | 464.23 | 662.52 | 301.39 | 464.23 (301.39 to 662.52) |
| Madagascar | 469.54 | 690.24 | 300.88 | 469.54 (300.88 to 690.24) |
| Greenland | 472.82 | 695 | 308.07 | 472.82 (308.07 to 695) |
| Puerto Rico | 473.25 | 713.8 | 299.55 | 473.25 (299.55 to 713.8) |
| Ireland | 492.39 | 729.23 | 320.39 | 492.39 (320.39 to 729.23) |
| Norway | 503.28 | 723.33 | 318.79 | 503.28 (318.79 to 723.33) |
| Poland | 507.04 | 736.42 | 322.69 | 507.04 (322.69 to 736.42) |
| Sweden | 523.75 | 765.1 | 329.16 | 523.75 (329.16 to 765.1) |
| Portugal | 567.33 | 862.45 | 346.53 | 567.33 (346.53 to 862.45) |
| Haiti | 576.86 | 838.35 | 368.38 | 576.86 (368.38 to 838.35) |
| Rwanda | 577.64 | 861.38 | 371.6 | 577.64 (371.6 to 861.38) |
| United Kingdom | 652.79 | 941.37 | 423.81 | 652.79 (423.81 to 941.37) |
| New Zealand | 710.24 | 1050.08 | 456.95 | 710.24 (456.95 to 1050.08) |

Supplementary Table 14: Global ASYR for asthma in 2021

| **location** | **val** | **upper** | **lower** | **Rate_2021** |
| --- | --- | --- | --- | --- |
| Lesotho | 51.36 | 75.59 | 33.16 | 51.36 (33.16 to 75.59) |
| American Samoa | 61.36 | 93.15 | 38.58 | 61.36 (38.58 to 93.15) |
| Pakistan | 61.95 | 93.19 | 39.44 | 61.95 (39.44 to 93.19) |
| Kazakhstan | 62.51 | 91.89 | 39.12 | 62.51 (39.12 to 91.89) |
| Guam | 63.15 | 98.33 | 38.62 | 63.15 (38.62 to 98.33) |
| Northern Mariana Islands | 63.76 | 97.24 | 39.27 | 63.76 (39.27 to 97.24) |
| Bhutan | 64.29 | 97.26 | 40.09 | 64.29 (40.09 to 97.26) |
| Niue | 64.89 | 98.08 | 41.15 | 64.89 (41.15 to 98.08) |
| Cameroon | 65.09 | 98.41 | 39.99 | 65.09 (39.99 to 98.41) |
| South Africa | 65.43 | 97.26 | 41.55 | 65.43 (41.55 to 97.26) |
| Nepal | 66.18 | 98.61 | 42.98 | 66.18 (42.98 to 98.61) |
| Turkmenistan | 69.61 | 105.16 | 42.49 | 69.61 (42.49 to 105.16) |
| Palau | 69.68 | 105.08 | 43.9 | 69.68 (43.9 to 105.08) |
| Armenia | 69.96 | 103.57 | 41.81 | 69.96 (41.81 to 103.57) |
| Bangladesh | 70.27 | 106.08 | 44.2 | 70.27 (44.2 to 106.08) |
| Myanmar | 70.86 | 103.5 | 45.31 | 70.86 (45.31 to 103.5) |
| Maldives | 71.64 | 108.58 | 44.13 | 71.64 (44.13 to 108.58) |
| Cook Islands | 71.82 | 111.69 | 44.72 | 71.82 (44.72 to 111.69) |
| Tokelau | 73 | 108.19 | 46.22 | 73 (46.22 to 108.19) |
| Tuvalu | 77.68 | 115.62 | 50.11 | 77.68 (50.11 to 115.62) |
| China | 78 | 119.9 | 48.1 | 78 (48.1 to 119.9) |
| Ghana | 78.15 | 117.39 | 49.88 | 78.15 (49.88 to 117.39) |
| Seychelles | 78.5 | 118.93 | 49.11 | 78.5 (49.11 to 118.93) |
| Zambia | 79 | 118.37 | 50.05 | 79 (50.05 to 118.37) |
| Lao People's Democratic Republic | 80.28 | 117.25 | 51.42 | 80.28 (51.42 to 117.25) |
| Georgia | 80.3 | 121.91 | 49.62 | 80.3 (49.62 to 121.91) |
| Nauru | 81.32 | 116.84 | 53.16 | 81.32 (53.16 to 116.84) |
| Mali | 83.47 | 123.9 | 53.04 | 83.47 (53.04 to 123.9) |
| Samoa | 83.99 | 121.38 | 53.35 | 83.99 (53.35 to 121.38) |
| Cambodia | 84.38 | 127.05 | 53.95 | 84.38 (53.95 to 127.05) |
| Marshall Islands | 84.4 | 124.45 | 55.29 | 84.4 (55.29 to 124.45) |
| Tajikistan | 84.95 | 124.98 | 53.35 | 84.95 (53.35 to 124.98) |
| Senegal | 86.31 | 130.4 | 54.6 | 86.31 (54.6 to 130.4) |
| Micronesia (Federated States of) | 86.69 | 127.74 | 55.73 | 86.69 (55.73 to 127.74) |
| Zimbabwe | 86.71 | 129.78 | 56.66 | 86.71 (56.66 to 129.78) |
| Mongolia | 87.47 | 129.04 | 55.66 | 87.47 (55.66 to 129.04) |
| Cabo Verde | 87.74 | 135.18 | 54.98 | 87.74 (54.98 to 135.18) |
| Azerbaijan | 88.25 | 132.41 | 55.9 | 88.25 (55.9 to 132.41) |
| Vanuatu | 88.71 | 128.14 | 57.62 | 88.71 (57.62 to 128.14) |
| Liberia | 90.23 | 134.59 | 58.21 | 90.23 (58.21 to 134.59) |
| Ethiopia | 91.45 | 138.12 | 58.24 | 91.45 (58.24 to 138.12) |
| India | 92.62 | 137.59 | 59.27 | 92.62 (59.27 to 137.59) |
| Chad | 93.11 | 139.1 | 60.28 | 93.11 (60.28 to 139.1) |
| Solomon Islands | 93.43 | 132.48 | 59.9 | 93.43 (59.9 to 132.48) |
| Fiji | 93.57 | 137.95 | 60.82 | 93.57 (60.82 to 137.95) |
| Indonesia | 93.85 | 140.29 | 59.69 | 93.85 (59.69 to 140.29) |
| Morocco | 94.56 | 138.11 | 60.21 | 94.56 (60.21 to 138.11) |
| Mexico | 95.48 | 144.74 | 58.04 | 95.48 (58.04 to 144.74) |
| Namibia | 95.54 | 143.46 | 62.01 | 95.54 (62.01 to 143.46) |
| Russian Federation | 95.88 | 145.43 | 59.09 | 95.88 (59.09 to 145.43) |
| Equatorial Guinea | 96.73 | 145.21 | 60.83 | 96.73 (60.83 to 145.21) |
| Malaysia | 97.58 | 149.64 | 61.15 | 97.58 (61.15 to 149.64) |
| Kenya | 99.98 | 149.6 | 63.73 | 99.98 (63.73 to 149.6) |
| Kyrgyzstan | 101.75 | 153.36 | 63.57 | 101.75 (63.57 to 153.36) |
| Gabon | 101.9 | 151.18 | 64.52 | 101.9 (64.52 to 151.18) |
| Sudan | 101.98 | 155.15 | 64.64 | 101.98 (64.64 to 155.15) |
| Angola | 103.73 | 154.23 | 66.02 | 103.73 (66.02 to 154.23) |
| Albania | 106.37 | 159.41 | 65.8 | 106.37 (65.8 to 159.41) |
| Gambia | 106.73 | 162.16 | 68.15 | 106.73 (68.15 to 162.16) |
| Republic of Moldova | 107.16 | 161.54 | 65.28 | 107.16 (65.28 to 161.54) |
| Guatemala | 107.75 | 161.62 | 66.35 | 107.75 (66.35 to 161.62) |
| Democratic Republic of the Congo | 108.01 | 159.53 | 69.68 | 108.01 (69.68 to 159.53) |
| Saudi Arabia | 109.99 | 167.71 | 68.8 | 109.99 (68.8 to 167.71) |
| Estonia | 110.2 | 162.66 | 67.77 | 110.2 (67.77 to 162.66) |
| Lithuania | 111.6 | 165.78 | 67.94 | 111.6 (67.94 to 165.78) |
| Iran (Islamic Republic of) | 112.07 | 166.65 | 71.4 | 112.07 (71.4 to 166.65) |
| Mauritius | 113.69 | 169.29 | 72.31 | 113.69 (72.31 to 169.29) |
| Benin | 114.12 | 171.37 | 73.35 | 114.12 (73.35 to 171.37) |
| Timor-Leste | 116.99 | 172.77 | 75.83 | 116.99 (75.83 to 172.77) |
| Algeria | 117.71 | 171.68 | 73.07 | 117.71 (73.07 to 171.68) |
| Ukraine | 118.51 | 175.84 | 71.66 | 118.51 (71.66 to 175.84) |
| Viet Nam | 118.68 | 181.74 | 74.09 | 118.68 (74.09 to 181.74) |
| Papua New Guinea | 119.33 | 169.67 | 76.57 | 119.33 (76.57 to 169.67) |
| Republic of Korea | 119.76 | 178.3 | 74.1 | 119.76 (74.1 to 178.3) |
| Czechia | 120.95 | 179.3 | 72.47 | 120.95 (72.47 to 179.3) |
| Slovakia | 121.49 | 181.91 | 74.27 | 121.49 (74.27 to 181.91) |
| Latvia | 122.55 | 183.18 | 76.14 | 122.55 (76.14 to 183.18) |
| Thailand | 123.13 | 188.27 | 77.89 | 123.13 (77.89 to 188.27) |
| Egypt | 123.74 | 180.69 | 77.64 | 123.74 (77.64 to 180.69) |
| Iraq | 123.8 | 181.42 | 76.62 | 123.8 (76.62 to 181.42) |
| Sierra Leone | 125.58 | 186.63 | 81.19 | 125.58 (81.19 to 186.63) |
| Bahrain | 125.78 | 186.74 | 79.33 | 125.78 (79.33 to 186.74) |
| Tunisia | 126.05 | 184.75 | 79.36 | 126.05 (79.36 to 184.75) |
| Serbia | 127.81 | 191.1 | 79.79 | 127.81 (79.79 to 191.1) |
| Guinea-Bissau | 128.21 | 189.58 | 83.88 | 128.21 (83.88 to 189.58) |
| Cote d'Ivoire | 128.79 | 192.37 | 82.21 | 128.79 (82.21 to 192.37) |
| Libya | 129.91 | 188.37 | 80.49 | 129.91 (80.49 to 188.37) |
| Jordan | 129.92 | 192.35 | 80.38 | 129.92 (80.38 to 192.35) |
| Congo | 130.16 | 196.47 | 82.9 | 130.16 (82.9 to 196.47) |
| Hungary | 130.26 | 195.79 | 79.23 | 130.26 (79.23 to 195.79) |
| Tonga | 130.69 | 195.37 | 84.29 | 130.69 (84.29 to 195.37) |
| Sri Lanka | 130.94 | 188.45 | 84.05 | 130.94 (84.05 to 188.45) |
| Guinea | 131.7 | 195.37 | 83.67 | 131.7 (83.67 to 195.37) |
| Niger | 132.18 | 192.67 | 85.82 | 132.18 (85.82 to 192.67) |
| Bolivia (Plurinational State of) | 132.22 | 202.74 | 82.11 | 132.22 (82.11 to 202.74) |
| Botswana | 132.39 | 197.44 | 85.06 | 132.39 (85.06 to 197.44) |
| Honduras | 132.58 | 200.62 | 81.5 | 132.58 (81.5 to 200.62) |
| Qatar | 132.67 | 197.22 | 81.99 | 132.67 (81.99 to 197.22) |
| Singapore | 133.31 | 197.15 | 82.58 | 133.31 (82.58 to 197.15) |
| Ecuador | 135 | 208.81 | 80.42 | 135 (80.42 to 208.81) |
| Togo | 136.01 | 207.68 | 85.99 | 136.01 (85.99 to 207.68) |
| Kuwait | 136.44 | 202.39 | 83.93 | 136.44 (83.93 to 202.39) |
| Japan | 137.42 | 209.03 | 85.93 | 137.42 (85.93 to 209.03) |
| Djibouti | 137.42 | 209.33 | 88.36 | 137.42 (88.36 to 209.33) |
| Burkina Faso | 139.13 | 204.6 | 88.97 | 139.13 (88.97 to 204.6) |
| Trinidad and Tobago | 140.05 | 209.78 | 85.57 | 140.05 (85.57 to 209.78) |
| Colombia | 140.64 | 212.41 | 84.21 | 140.64 (84.21 to 212.41) |
| Dominican Republic | 141.4 | 213.62 | 89.5 | 141.4 (89.5 to 213.62) |
| Mozambique | 141.91 | 209.79 | 91.24 | 141.91 (91.24 to 209.79) |
| Bulgaria | 142.93 | 213.56 | 87.58 | 142.93 (87.58 to 213.56) |
| Montenegro | 143 | 212.66 | 87.58 | 143 (87.58 to 212.66) |
| Kiribati | 143.37 | 203.73 | 94.7 | 143.37 (94.7 to 203.73) |
| Oman | 144.3 | 213.58 | 88.25 | 144.3 (88.25 to 213.58) |
| Palestine | 144.53 | 211.85 | 90.13 | 144.53 (90.13 to 211.85) |
| Italy | 146.74 | 222.01 | 90.76 | 146.74 (90.76 to 222.01) |
| Uzbekistan | 147.53 | 218.39 | 95.44 | 147.53 (95.44 to 218.39) |
| Israel | 148.98 | 218.84 | 91.22 | 148.98 (91.22 to 218.84) |
| Central African Republic | 154.72 | 227.58 | 100.2 | 154.72 (100.2 to 227.58) |
| Yemen | 155.57 | 224.25 | 100.77 | 155.57 (100.77 to 224.25) |
| Democratic People's Republic of Korea | 158.33 | 232.57 | 101.64 | 158.33 (101.64 to 232.57) |
| Malawi | 158.73 | 237.37 | 101.71 | 158.73 (101.71 to 237.37) |
| Sao Tome and Principe | 158.76 | 238.37 | 104.68 | 158.76 (104.68 to 238.37) |
| Syrian Arab Republic | 160.41 | 238.41 | 103.18 | 160.41 (103.18 to 238.41) |
| Croatia | 160.56 | 237.55 | 98.46 | 160.56 (98.46 to 237.55) |
| Nicaragua | 161.02 | 246.76 | 97.69 | 161.02 (97.69 to 246.76) |
| Venezuela (Bolivarian Republic of) | 165.31 | 254.16 | 99.67 | 165.31 (99.67 to 254.16) |
| Belarus | 165.65 | 244.42 | 103.86 | 165.65 (103.86 to 244.42) |
| Germany | 167.98 | 244.93 | 103.27 | 167.98 (103.27 to 244.93) |
| South Sudan | 169.67 | 252.38 | 109.39 | 169.67 (109.39 to 252.38) |
| Lebanon | 170.29 | 250.83 | 104.92 | 170.29 (104.92 to 250.83) |
| Eritrea | 170.67 | 249.95 | 109.98 | 170.67 (109.98 to 249.95) |
| Spain | 170.75 | 248.83 | 105.34 | 170.75 (105.34 to 248.83) |
| Comoros | 174.23 | 261.41 | 110.51 | 174.23 (110.51 to 261.41) |
| Romania | 179.54 | 265.43 | 111.21 | 179.54 (111.21 to 265.43) |
| Belgium | 181.88 | 267.78 | 112.77 | 181.88 (112.77 to 267.78) |
| Brunei Darussalam | 182.54 | 264.92 | 117.42 | 182.54 (117.42 to 264.92) |
| Denmark | 182.76 | 268.27 | 114.16 | 182.76 (114.16 to 268.27) |
| Philippines | 183.63 | 270.96 | 117.07 | 183.63 (117.07 to 270.96) |
| Nigeria | 183.88 | 278.25 | 119.28 | 183.88 (119.28 to 278.25) |
| Afghanistan | 184.04 | 267.94 | 120.14 | 184.04 (120.14 to 267.94) |
| Panama | 185.68 | 283.9 | 113.17 | 185.68 (113.17 to 283.9) |
| Peru | 187.74 | 296.1 | 111.86 | 187.74 (111.86 to 296.1) |
| Brazil | 188.15 | 288.32 | 116.34 | 188.15 (116.34 to 288.32) |
| Greece | 188.25 | 277.14 | 116.86 | 188.25 (116.86 to 277.14) |
| Austria | 188.98 | 282.92 | 117.27 | 188.98 (117.27 to 282.92) |
| Taiwan (Province of China) | 190.29 | 301.51 | 119.38 | 190.29 (119.38 to 301.51) |
| Turkiye | 190.69 | 280.92 | 119.66 | 190.69 (119.66 to 280.92) |
| Burundi | 193.39 | 285.8 | 125.15 | 193.39 (125.15 to 285.8) |
| Mauritania | 193.4 | 290.05 | 122.5 | 193.4 (122.5 to 290.05) |
| Bosnia and Herzegovina | 195.77 | 288.07 | 120.95 | 195.77 (120.95 to 288.07) |
| Suriname | 205.1 | 312.31 | 127.14 | 205.1 (127.14 to 312.31) |
| Uruguay | 205.55 | 307.39 | 130 | 205.55 (130 to 307.39) |
| Uganda | 205.81 | 310.61 | 131.21 | 205.81 (131.21 to 310.61) |
| Somalia | 210 | 306.01 | 135.42 | 210 (135.42 to 306.01) |
| Paraguay | 210.99 | 323.66 | 128.67 | 210.99 (128.67 to 323.66) |
| Slovenia | 212.23 | 313.02 | 132.6 | 212.23 (132.6 to 313.02) |
| North Macedonia | 219.83 | 318.86 | 138.11 | 219.83 (138.11 to 318.86) |
| Saint Vincent and the Grenadines | 219.87 | 333.59 | 138.67 | 219.87 (138.67 to 333.59) |
| Saint Kitts and Nevis | 222.26 | 338.08 | 136.03 | 222.26 (136.03 to 338.08) |
| Switzerland | 222.71 | 332.4 | 137.27 | 222.71 (137.27 to 332.4) |
| Antigua and Barbuda | 227.82 | 345.11 | 138.6 | 227.82 (138.6 to 345.11) |
| Eswatini | 229.34 | 337.96 | 147.87 | 229.34 (147.87 to 337.96) |
| Guyana | 229.82 | 346.96 | 146.6 | 229.82 (146.6 to 346.96) |
| Belize | 230.76 | 344.6 | 143.01 | 230.76 (143.01 to 344.6) |
| Canada | 231.74 | 354.78 | 145.84 | 231.74 (145.84 to 354.78) |
| Bahamas | 232.11 | 354.9 | 140.94 | 232.11 (140.94 to 354.9) |
| Argentina | 234.78 | 353.57 | 148.03 | 234.78 (148.03 to 353.57) |
| El Salvador | 235.13 | 348.18 | 142.34 | 235.13 (142.34 to 348.18) |
| San Marino | 236.43 | 356.41 | 147.73 | 236.43 (147.73 to 356.41) |
| Jamaica | 236.53 | 351.17 | 146.56 | 236.53 (146.56 to 351.17) |
| Norway | 238.99 | 358.49 | 150.3 | 238.99 (150.3 to 358.49) |
| Monaco | 239.01 | 366.12 | 147.58 | 239.01 (147.58 to 366.12) |
| Costa Rica | 239.6 | 359.53 | 147.42 | 239.6 (147.42 to 359.53) |
| Finland | 241.51 | 352.2 | 151.61 | 241.51 (151.61 to 352.2) |
| Chile | 243.23 | 357.31 | 153.32 | 243.23 (153.32 to 357.31) |
| France | 245.55 | 365.51 | 152.07 | 245.55 (152.07 to 365.51) |
| Dominica | 247.3 | 373.42 | 156.43 | 247.3 (156.43 to 373.42) |
| United Republic of Tanzania | 249.74 | 388.2 | 156.32 | 249.74 (156.32 to 388.2) |
| Netherlands | 256.22 | 378.99 | 159.54 | 256.22 (159.54 to 378.99) |
| Andorra | 257.59 | 389.2 | 160.74 | 257.59 (160.74 to 389.2) |
| Bermuda | 262.23 | 400.73 | 160.44 | 262.23 (160.44 to 400.73) |
| Malta | 263.54 | 395.84 | 162.04 | 263.54 (162.04 to 395.84) |
| Saint Lucia | 268.85 | 400.88 | 170.36 | 268.85 (170.36 to 400.88) |
| United States Virgin Islands | 269.78 | 411.4 | 163.69 | 269.78 (163.69 to 411.4) |
| Madagascar | 272.55 | 409.76 | 178.2 | 272.55 (178.2 to 409.76) |
| Ireland | 272.73 | 403.15 | 165.31 | 272.73 (165.31 to 403.15) |
| Luxembourg | 279.8 | 419.18 | 174.68 | 279.8 (174.68 to 419.18) |
| Iceland | 289.97 | 436.28 | 176.83 | 289.97 (176.83 to 436.28) |
| Cyprus | 290.62 | 437.27 | 176.63 | 290.62 (176.63 to 437.27) |
| Sweden | 296.58 | 446.65 | 186.51 | 296.58 (186.51 to 446.65) |
| Cuba | 297.54 | 458.62 | 183.07 | 297.54 (183.07 to 458.62) |
| Greenland | 298.99 | 441.84 | 192 | 298.99 (192 to 441.84) |
| New Zealand | 300.25 | 455.75 | 193.38 | 300.25 (193.38 to 455.75) |
| Australia | 307.39 | 459.23 | 192.38 | 307.39 (192.38 to 459.23) |
| United Arab Emirates | 317.3 | 459 | 201.53 | 317.3 (201.53 to 459) |
| Barbados | 325.31 | 483.24 | 201.83 | 325.31 (201.83 to 483.24) |
| Grenada | 345.88 | 530.24 | 210.51 | 345.88 (210.51 to 530.24) |
| Rwanda | 364.41 | 549.51 | 233.52 | 364.41 (233.52 to 549.51) |
| Poland | 369.05 | 551.09 | 235.51 | 369.05 (235.51 to 551.09) |
| Portugal | 371.01 | 556.67 | 230.3 | 371.01 (230.3 to 556.67) |
| United Kingdom | 394.56 | 586.99 | 249.32 | 394.56 (249.32 to 586.99) |
| United States of America | 397.64 | 594.29 | 259.25 | 397.64 (259.25 to 594.29) |
| Puerto Rico | 399.42 | 602.52 | 246.32 | 399.42 (246.32 to 602.52) |
| Haiti | 453.92 | 657.93 | 292.48 | 453.92 (292.48 to 657.93) |

Supplementary Table 15: Global EAPCs of YLDs for asthma

| **location** | **EAPC** | **UCI** | **LCI** | **EAPC_CI** | **val** |
| --- | --- | --- | --- | --- | --- |
| Japan | -3.55544 | -3.28152 | -3.82857 | -3.55544% (-3.82857 to -3.28152) | -3.55544 |
| Turkmenistan | -3.10005 | -2.95078 | -3.24909 | -3.10005% (-3.24909 to -2.95078) | -3.10005 |
| Russian Federation | -2.95624 | -2.84857 | -3.06379 | -2.95624% (-3.06379 to -2.84857) | -2.95624 |
| Guatemala | -2.85211 | -2.57403 | -3.12939 | -2.85211% (-3.12939 to -2.57403) | -2.85211 |
| Tuvalu | -2.83163 | -2.7687 | -2.89452 | -2.83163% (-2.89452 to -2.7687) | -2.83163 |
| South Africa | -2.78699 | -2.48493 | -3.08812 | -2.78699% (-3.08812 to -2.48493) | -2.78699 |
| New Zealand | -2.63341 | -2.33231 | -2.93358 | -2.63341% (-2.93358 to -2.33231) | -2.63341 |
| Singapore | -2.5911 | -2.33257 | -2.84895 | -2.5911% (-2.84895 to -2.33257) | -2.5911 |
| Norway | -2.57945 | -2.47612 | -2.68268 | -2.57945% (-2.68268 to -2.47612) | -2.57945 |
| Honduras | -2.43123 | -2.34315 | -2.51923 | -2.43123% (-2.51923 to -2.34315) | -2.43123 |
| Maldives | -2.39032 | -2.28309 | -2.49742 | -2.39032% (-2.49742 to -2.28309) | -2.39032 |
| Bangladesh | -2.37699 | -2.27484 | -2.47904 | -2.37699% (-2.47904 to -2.27484) | -2.37699 |
| Equatorial Guinea | -2.28894 | -2.19735 | -2.38045 | -2.28894% (-2.38045 to -2.19735) | -2.28894 |
| Fiji | -2.26016 | -2.13121 | -2.38894 | -2.26016% (-2.38894 to -2.13121) | -2.26016 |
| Nauru | -2.25061 | -1.98554 | -2.51496 | -2.25061% (-2.51496 to -1.98554) | -2.25061 |
| Tokelau | -2.24661 | -2.13003 | -2.36304 | -2.24661% (-2.36304 to -2.13003) | -2.24661 |
| Ethiopia | -2.22612 | -2.07142 | -2.38058 | -2.22612% (-2.38058 to -2.07142) | -2.22612 |
| Bolivia (Plurinational State of) | -2.20343 | -2.12701 | -2.27979 | -2.20343% (-2.27979 to -2.12701) | -2.20343 |
| Angola | -2.19313 | -2.01533 | -2.37061 | -2.19313% (-2.37061 to -2.01533) | -2.19313 |
| Ecuador | -2.16531 | -1.83039 | -2.49908 | -2.16531% (-2.49908 to -1.83039) | -2.16531 |
| Micronesia (Federated States of) | -2.09245 | -2.04011 | -2.14476 | -2.09245% (-2.14476 to -2.04011) | -2.09245 |
| Belarus | -2.08139 | -1.86614 | -2.29617 | -2.08139% (-2.29617 to -1.86614) | -2.08139 |
| El Salvador | -2.05187 | -1.92632 | -2.17726 | -2.05187% (-2.17726 to -1.92632) | -2.05187 |
| Italy | -2.00814 | -1.84 | -2.17599 | -2.00814% (-2.17599 to -1.84) | -2.00814 |
| Mexico | -2.0061 | -1.74606 | -2.26546 | -2.0061% (-2.26546 to -1.74606) | -2.0061 |
| Niue | -2.00534 | -1.80481 | -2.20546 | -2.00534% (-2.20546 to -1.80481) | -2.00534 |
| Ireland | -1.99615 | -1.88381 | -2.10836 | -1.99615% (-2.10836 to -1.88381) | -1.99615 |
| Lao People's Democratic Republic | -1.98764 | -1.92136 | -2.05388 | -1.98764% (-2.05388 to -1.92136) | -1.98764 |
| Mauritius | -1.98189 | -1.88641 | -2.07727 | -1.98189% (-2.07727 to -1.88641) | -1.98189 |
| Vanuatu | -1.97311 | -1.83579 | -2.11024 | -1.97311% (-2.11024 to -1.83579) | -1.97311 |
| Afghanistan | -1.96246 | -1.85783 | -2.06697 | -1.96246% (-2.06697 to -1.85783) | -1.96246 |
| Guam | -1.96012 | -1.513 | -2.40521 | -1.96012% (-2.40521 to -1.513) | -1.96012 |
| Nicaragua | -1.95814 | -1.87012 | -2.04608 | -1.95814% (-2.04608 to -1.87012) | -1.95814 |
| Sudan | -1.95779 | -1.8199 | -2.09548 | -1.95779% (-2.09548 to -1.8199) | -1.95779 |
| Palau | -1.9564 | -1.69827 | -2.21386 | -1.9564% (-2.21386 to -1.69827) | -1.9564 |
| Iran (Islamic Republic of) | -1.95117 | -1.86873 | -2.03354 | -1.95117% (-2.03354 to -1.86873) | -1.95117 |
| Turkiye | -1.9082 | -1.69333 | -2.1226 | -1.9082% (-2.1226 to -1.69333) | -1.9082 |
| Brazil | -1.89387 | -1.76755 | -2.02004 | -1.89387% (-2.02004 to -1.76755) | -1.89387 |
| Kiribati | -1.89294 | -1.78032 | -2.00543 | -1.89294% (-2.00543 to -1.78032) | -1.89294 |
| Pakistan | -1.87288 | -1.68761 | -2.0578 | -1.87288% (-2.0578 to -1.68761) | -1.87288 |
| Burundi | -1.8728 | -1.80724 | -1.93831 | -1.8728% (-1.93831 to -1.80724) | -1.8728 |
| Marshall Islands | -1.86888 | -1.71448 | -2.02303 | -1.86888% (-2.02303 to -1.71448) | -1.86888 |
| Myanmar | -1.84844 | -1.7608 | -1.936 | -1.84844% (-1.936 to -1.7608) | -1.84844 |
| Madagascar | -1.84657 | -1.7641 | -1.92897 | -1.84657% (-1.92897 to -1.7641) | -1.84657 |
| Belgium | -1.83264 | -1.52831 | -2.13604 | -1.83264% (-2.13604 to -1.52831) | -1.83264 |
| Ukraine | -1.83078 | -1.7483 | -1.9132 | -1.83078% (-1.9132 to -1.7483) | -1.83078 |
| Indonesia | -1.82675 | -1.69846 | -1.95488 | -1.82675% (-1.95488 to -1.69846) | -1.82675 |
| Greenland | -1.82078 | -1.72351 | -1.91795 | -1.82078% (-1.91795 to -1.72351) | -1.82078 |
| Bhutan | -1.81747 | -1.74114 | -1.89375 | -1.81747% (-1.89375 to -1.74114) | -1.81747 |
| Yemen | -1.79654 | -1.76028 | -1.8328 | -1.79654% (-1.8328 to -1.76028) | -1.79654 |
| Israel | -1.79111 | -1.64759 | -1.93442 | -1.79111% (-1.93442 to -1.64759) | -1.79111 |
| Malaysia | -1.77792 | -1.68348 | -1.87227 | -1.77792% (-1.87227 to -1.68348) | -1.77792 |
| Sweden | -1.77162 | -1.3636 | -2.17795 | -1.77162% (-2.17795 to -1.3636) | -1.77162 |
| Luxembourg | -1.764 | -1.67153 | -1.85639 | -1.764% (-1.85639 to -1.67153) | -1.764 |
| Rwanda | -1.75551 | -1.659 | -1.85193 | -1.75551% (-1.85193 to -1.659) | -1.75551 |
| Republic of Moldova | -1.74885 | -1.6934 | -1.80427 | -1.74885% (-1.80427 to -1.6934) | -1.74885 |
| Australia | -1.73584 | -1.44943 | -2.02142 | -1.73584% (-2.02142 to -1.44943) | -1.73584 |
| Papua New Guinea | -1.73143 | -1.62585 | -1.8369 | -1.73143% (-1.8369 to -1.62585) | -1.73143 |
| Kyrgyzstan | -1.71043 | -1.62768 | -1.79311 | -1.71043% (-1.79311 to -1.62768) | -1.71043 |
| Venezuela (Bolivarian Republic of) | -1.70427 | -1.47725 | -1.93077 | -1.70427% (-1.93077 to -1.47725) | -1.70427 |
| Eritrea | -1.69684 | -1.62596 | -1.76766 | -1.69684% (-1.76766 to -1.62596) | -1.69684 |
| American Samoa | -1.68514 | -1.42655 | -1.94305 | -1.68514% (-1.94305 to -1.42655) | -1.68514 |
| Timor-Leste | -1.68329 | -1.58791 | -1.77857 | -1.68329% (-1.77857 to -1.58791) | -1.68329 |
| United Kingdom | -1.67524 | -1.4887 | -1.86143 | -1.67524% (-1.86143 to -1.4887) | -1.67524 |
| Peru | -1.6519 | -1.46468 | -1.83876 | -1.6519% (-1.83876 to -1.46468) | -1.6519 |
| Iraq | -1.64127 | -1.53511 | -1.74731 | -1.64127% (-1.74731 to -1.53511) | -1.64127 |
| Iceland | -1.62716 | -1.53965 | -1.71459 | -1.62716% (-1.71459 to -1.53965) | -1.62716 |
| Northern Mariana Islands | -1.58833 | -1.10364 | -2.07065 | -1.58833% (-2.07065 to -1.10364) | -1.58833 |
| Congo | -1.587 | -1.4988 | -1.67513 | -1.587% (-1.67513 to -1.4988) | -1.587 |
| Djibouti | -1.57933 | -1.49682 | -1.66177 | -1.57933% (-1.66177 to -1.49682) | -1.57933 |
| Austria | -1.56888 | -1.43506 | -1.70253 | -1.56888% (-1.70253 to -1.43506) | -1.56888 |
| Germany | -1.55219 | -1.18035 | -1.92264 | -1.55219% (-1.92264 to -1.18035) | -1.55219 |
| Bahrain | -1.5449 | -1.42523 | -1.66442 | -1.5449% (-1.66442 to -1.42523) | -1.5449 |
| France | -1.54114 | -1.37739 | -1.70462 | -1.54114% (-1.70462 to -1.37739) | -1.54114 |
| China | -1.52254 | -1.168 | -1.87581 | -1.52254% (-1.87581 to -1.168) | -1.52254 |
| Colombia | -1.50045 | -1.20326 | -1.79675 | -1.50045% (-1.79675 to -1.20326) | -1.50045 |
| Latvia | -1.47871 | -1.34382 | -1.61341 | -1.47871% (-1.61341 to -1.34382) | -1.47871 |
| Guinea-Bissau | -1.47833 | -1.42823 | -1.5284 | -1.47833% (-1.5284 to -1.42823) | -1.47833 |
| Uzbekistan | -1.46963 | -1.33153 | -1.60754 | -1.46963% (-1.60754 to -1.33153) | -1.46963 |
| Solomon Islands | -1.46486 | -1.35408 | -1.57551 | -1.46486% (-1.57551 to -1.35408) | -1.46486 |
| Egypt | -1.44866 | -1.37606 | -1.52121 | -1.44866% (-1.52121 to -1.37606) | -1.44866 |
| North Macedonia | -1.4475 | -1.23289 | -1.66165 | -1.4475% (-1.66165 to -1.23289) | -1.4475 |
| Andorra | -1.44408 | -1.34289 | -1.54517 | -1.44408% (-1.54517 to -1.34289) | -1.44408 |
| Croatia | -1.43489 | -1.2599 | -1.60956 | -1.43489% (-1.60956 to -1.2599) | -1.43489 |
| Denmark | -1.43111 | -1.21373 | -1.64801 | -1.43111% (-1.64801 to -1.21373) | -1.43111 |
| India | -1.41534 | -1.08313 | -1.74644 | -1.41534% (-1.74644 to -1.08313) | -1.41534 |
| Republic of Korea | -1.40108 | -1.28321 | -1.5188 | -1.40108% (-1.5188 to -1.28321) | -1.40108 |
| Thailand | -1.40079 | -1.25777 | -1.54361 | -1.40079% (-1.54361 to -1.25777) | -1.40079 |
| Cabo Verde | -1.38803 | -1.24324 | -1.53261 | -1.38803% (-1.53261 to -1.24324) | -1.38803 |
| Switzerland | -1.38791 | -1.3395 | -1.4363 | -1.38791% (-1.4363 to -1.3395) | -1.38791 |
| Netherlands | -1.37881 | -1.10167 | -1.65518 | -1.37881% (-1.65518 to -1.10167) | -1.37881 |
| Panama | -1.34783 | -1.27455 | -1.42105 | -1.34783% (-1.42105 to -1.27455) | -1.34783 |
| Liberia | -1.3299 | -1.26979 | -1.38997 | -1.3299% (-1.38997 to -1.26979) | -1.3299 |
| South Sudan | -1.32349 | -1.24491 | -1.40201 | -1.32349% (-1.40201 to -1.24491) | -1.32349 |
| Cambodia | -1.3145 | -1.2383 | -1.39064 | -1.3145% (-1.39064 to -1.2383) | -1.3145 |
| Malta | -1.29723 | -1.23007 | -1.36436 | -1.29723% (-1.36436 to -1.23007) | -1.29723 |
| Gabon | -1.28964 | -1.23415 | -1.3451 | -1.28964% (-1.3451 to -1.23415) | -1.28964 |
| Estonia | -1.28266 | -1.155 | -1.41016 | -1.28266% (-1.41016 to -1.155) | -1.28266 |
| Philippines | -1.25708 | -1.15957 | -1.35448 | -1.25708% (-1.35448 to -1.15957) | -1.25708 |
| Mongolia | -1.23258 | -1.1662 | -1.29892 | -1.23258% (-1.29892 to -1.1662) | -1.23258 |
| Sri Lanka | -1.22991 | -1.15666 | -1.3031 | -1.22991% (-1.3031 to -1.15666) | -1.22991 |
| Namibia | -1.22951 | -1.16801 | -1.29098 | -1.22951% (-1.29098 to -1.16801) | -1.22951 |
| Sao Tome and Principe | -1.2214 | -1.14195 | -1.30078 | -1.2214% (-1.30078 to -1.14195) | -1.2214 |
| Bulgaria | -1.21183 | -1.11854 | -1.30503 | -1.21183% (-1.30503 to -1.11854) | -1.21183 |
| Samoa | -1.21164 | -1.15473 | -1.26851 | -1.21164% (-1.26851 to -1.15473) | -1.21164 |
| Comoros | -1.20221 | -1.13097 | -1.27339 | -1.20221% (-1.27339 to -1.13097) | -1.20221 |
| Kenya | -1.18493 | -1.06595 | -1.30377 | -1.18493% (-1.30377 to -1.06595) | -1.18493 |
| Benin | -1.17965 | -1.13188 | -1.2274 | -1.17965% (-1.2274 to -1.13188) | -1.17965 |
| Mozambique | -1.15567 | -1.09094 | -1.22036 | -1.15567% (-1.22036 to -1.09094) | -1.15567 |
| Costa Rica | -1.15276 | -0.99868 | -1.30661 | -1.15276% (-1.30661 to -0.99868) | -1.15276 |
| Somalia | -1.14708 | -1.10107 | -1.19308 | -1.14708% (-1.19308 to -1.10107) | -1.14708 |
| Uganda | -1.14599 | -1.07556 | -1.21637 | -1.14599% (-1.21637 to -1.07556) | -1.14599 |
| Mauritania | -1.14083 | -1.0712 | -1.21041 | -1.14083% (-1.21041 to -1.0712) | -1.14083 |
| Greece | -1.13035 | -1.0055 | -1.25505 | -1.13035% (-1.25505 to -1.0055) | -1.13035 |
| Azerbaijan | -1.1195 | -1.05052 | -1.18844 | -1.1195% (-1.18844 to -1.05052) | -1.1195 |
| Tajikistan | -1.11746 | -1.07294 | -1.16196 | -1.11746% (-1.16196 to -1.07294) | -1.11746 |
| Slovenia | -1.11192 | -1.05798 | -1.16584 | -1.11192% (-1.16584 to -1.05798) | -1.11192 |
| Niger | -1.0971 | -1.03607 | -1.15809 | -1.0971% (-1.15809 to -1.03607) | -1.0971 |
| Tonga | -1.09574 | -0.96928 | -1.22204 | -1.09574% (-1.22204 to -0.96928) | -1.09574 |
| Uruguay | -1.09348 | -1.03236 | -1.15457 | -1.09348% (-1.15457 to -1.03236) | -1.09348 |
| Cook Islands | -1.08568 | -0.63608 | -1.53325 | -1.08568% (-1.53325 to -0.63608) | -1.08568 |
| Dominican Republic | -1.08305 | -0.91941 | -1.24641 | -1.08305% (-1.24641 to -0.91941) | -1.08305 |
| Portugal | -1.07688 | -0.73349 | -1.41909 | -1.07688% (-1.41909 to -0.73349) | -1.07688 |
| United Arab Emirates | -1.05343 | -0.96597 | -1.14081 | -1.05343% (-1.14081 to -0.96597) | -1.05343 |
| Zambia | -1.04908 | -0.97125 | -1.12685 | -1.04908% (-1.12685 to -0.97125) | -1.04908 |
| Malawi | -1.04462 | -0.9781 | -1.11109 | -1.04462% (-1.11109 to -0.9781) | -1.04462 |
| Senegal | -1.03291 | -0.95393 | -1.11183 | -1.03291% (-1.11183 to -0.95393) | -1.03291 |
| Poland | -1.02683 | -0.60662 | -1.44527 | -1.02683% (-1.44527 to -0.60662) | -1.02683 |
| Eswatini | -0.99962 | -0.92187 | -1.07732 | -0.99962% (-1.07732 to -0.92187) | -0.99962 |
| Jamaica | -0.99443 | -0.85201 | -1.13665 | -0.99443% (-1.13665 to -0.85201) | -0.99443 |
| Hungary | -0.9921 | -0.85517 | -1.12883 | -0.9921% (-1.12883 to -0.85517) | -0.9921 |
| Seychelles | -0.98984 | -0.85117 | -1.12831 | -0.98984% (-1.12831 to -0.85117) | -0.98984 |
| Nepal | -0.98731 | -0.93182 | -1.04276 | -0.98731% (-1.04276 to -0.93182) | -0.98731 |
| Democratic Republic of the Congo | -0.96101 | -0.89169 | -1.03028 | -0.96101% (-1.03028 to -0.89169) | -0.96101 |
| Libya | -0.952 | -0.89228 | -1.01168 | -0.952% (-1.01168 to -0.89228) | -0.952 |
| Democratic People's Republic of Korea | -0.94937 | -0.87834 | -1.02034 | -0.94937% (-1.02034 to -0.87834) | -0.94937 |
| Botswana | -0.93641 | -0.87771 | -0.99508 | -0.93641% (-0.99508 to -0.87771) | -0.93641 |
| Nigeria | -0.91779 | -0.84875 | -0.98679 | -0.91779% (-0.98679 to -0.84875) | -0.91779 |
| Ghana | -0.91212 | -0.85863 | -0.96558 | -0.91212% (-0.96558 to -0.85863) | -0.91212 |
| Guinea | -0.91208 | -0.86273 | -0.96141 | -0.91208% (-0.96141 to -0.86273) | -0.91208 |
| Central African Republic | -0.88911 | -0.80754 | -0.97061 | -0.88911% (-0.97061 to -0.80754) | -0.88911 |
| Gambia | -0.88509 | -0.83077 | -0.93937 | -0.88509% (-0.93937 to -0.83077) | -0.88509 |
| Serbia | -0.87385 | -0.73472 | -1.01278 | -0.87385% (-1.01278 to -0.73472) | -0.87385 |
| Jordan | -0.87221 | -0.73066 | -1.01356 | -0.87221% (-1.01356 to -0.73066) | -0.87221 |
| Cameroon | -0.87008 | -0.80775 | -0.93237 | -0.87008% (-0.93237 to -0.80775) | -0.87008 |
| Brunei Darussalam | -0.86707 | -0.81415 | -0.91995 | -0.86707% (-0.91995 to -0.81415) | -0.86707 |
| Argentina | -0.86233 | -0.81115 | -0.91349 | -0.86233% (-0.91349 to -0.81115) | -0.86233 |
| Haiti | -0.86052 | -0.79037 | -0.93063 | -0.86052% (-0.93063 to -0.79037) | -0.86052 |
| Georgia | -0.85764 | -0.75255 | -0.96262 | -0.85764% (-0.96262 to -0.75255) | -0.85764 |
| Viet Nam | -0.84971 | -0.71664 | -0.98259 | -0.84971% (-0.98259 to -0.71664) | -0.84971 |
| Puerto Rico | -0.81665 | -0.65003 | -0.98299 | -0.81665% (-0.98299 to -0.65003) | -0.81665 |
| Romania | -0.81296 | -0.67833 | -0.94739 | -0.81296% (-0.94739 to -0.67833) | -0.81296 |
| Sierra Leone | -0.80854 | -0.75976 | -0.85729 | -0.80854% (-0.85729 to -0.75976) | -0.80854 |
| Tunisia | -0.80527 | -0.7382 | -0.87229 | -0.80527% (-0.87229 to -0.7382) | -0.80527 |
| Qatar | -0.79228 | -0.72818 | -0.85634 | -0.79228% (-0.85634 to -0.72818) | -0.79228 |
| Lebanon | -0.78699 | -0.73991 | -0.83404 | -0.78699% (-0.83404 to -0.73991) | -0.78699 |
| Saint Lucia | -0.77856 | -0.6492 | -0.90776 | -0.77856% (-0.90776 to -0.6492) | -0.77856 |
| Kuwait | -0.76444 | -0.64558 | -0.88317 | -0.76444% (-0.88317 to -0.64558) | -0.76444 |
| Algeria | -0.72024 | -0.66841 | -0.77205 | -0.72024% (-0.77205 to -0.66841) | -0.72024 |
| Cote d'Ivoire | -0.70971 | -0.63669 | -0.78269 | -0.70971% (-0.78269 to -0.63669) | -0.70971 |
| Mali | -0.7001 | -0.63714 | -0.76303 | -0.7001% (-0.76303 to -0.63714) | -0.7001 |
| Lesotho | -0.6817 | -0.62578 | -0.7376 | -0.6817% (-0.7376 to -0.62578) | -0.6817 |
| Palestine | -0.64161 | -0.53616 | -0.74696 | -0.64161% (-0.74696 to -0.53616) | -0.64161 |
| Lithuania | -0.63269 | -0.53702 | -0.72827 | -0.63269% (-0.72827 to -0.53702) | -0.63269 |
| Cyprus | -0.62504 | -0.54156 | -0.70846 | -0.62504% (-0.70846 to -0.54156) | -0.62504 |
| Canada | -0.60472 | -0.40913 | -0.79993 | -0.60472% (-0.79993 to -0.40913) | -0.60472 |
| Finland | -0.5832 | -0.52413 | -0.64223 | -0.5832% (-0.64223 to -0.52413) | -0.5832 |
| Belize | -0.58159 | -0.45117 | -0.71183 | -0.58159% (-0.71183 to -0.45117) | -0.58159 |
| Trinidad and Tobago | -0.55001 | 0.01471 | -1.11154 | -0.55001% (-1.11154 to 0.01471) | -0.55001 |
| Morocco | -0.54984 | -0.46974 | -0.62987 | -0.54984% (-0.62987 to -0.46974) | -0.54984 |
| Chad | -0.54431 | -0.50029 | -0.5883 | -0.54431% (-0.5883 to -0.50029) | -0.54431 |
| Grenada | -0.54341 | -0.46259 | -0.62416 | -0.54341% (-0.62416 to -0.46259) | -0.54341 |
| Czechia | -0.52675 | -0.4542 | -0.59925 | -0.52675% (-0.59925 to -0.4542) | -0.52675 |
| Taiwan (Province of China) | -0.5146 | -0.25268 | -0.77582 | -0.5146% (-0.77582 to -0.25268) | -0.5146 |
| Spain | -0.50272 | -0.29531 | -0.70969 | -0.50272% (-0.70969 to -0.29531) | -0.50272 |
| Guyana | -0.49569 | -0.33094 | -0.66016 | -0.49569% (-0.66016 to -0.33094) | -0.49569 |
| Togo | -0.49511 | -0.44493 | -0.54526 | -0.49511% (-0.54526 to -0.44493) | -0.49511 |
| Monaco | -0.46992 | -0.42151 | -0.5183 | -0.46992% (-0.5183 to -0.42151) | -0.46992 |
| Antigua and Barbuda | -0.45815 | -0.24577 | -0.67008 | -0.45815% (-0.67008 to -0.24577) | -0.45815 |
| Chile | -0.43071 | -0.31517 | -0.54611 | -0.43071% (-0.54611 to -0.31517) | -0.43071 |
| Burkina Faso | -0.42406 | -0.37192 | -0.47618 | -0.42406% (-0.47618 to -0.37192) | -0.42406 |
| Dominica | -0.42347 | -0.20345 | -0.643 | -0.42347% (-0.643 to -0.20345) | -0.42347 |
| Suriname | -0.3971 | -0.24565 | -0.54833 | -0.3971% (-0.54833 to -0.24565) | -0.3971 |
| Cuba | -0.38998 | -0.27482 | -0.50502 | -0.38998% (-0.50502 to -0.27482) | -0.38998 |
| Slovakia | -0.38689 | -0.32936 | -0.44439 | -0.38689% (-0.44439 to -0.32936) | -0.38689 |
| Armenia | -0.3693 | -0.11448 | -0.62347 | -0.3693% (-0.62347 to -0.11448) | -0.3693 |
| Albania | -0.34534 | -0.20401 | -0.48646 | -0.34534% (-0.48646 to -0.20401) | -0.34534 |
| Saint Vincent and the Grenadines | -0.33059 | -0.15818 | -0.5027 | -0.33059% (-0.5027 to -0.15818) | -0.33059 |
| San Marino | -0.31805 | -0.28472 | -0.35137 | -0.31805% (-0.35137 to -0.28472) | -0.31805 |
| United Republic of Tanzania | -0.31613 | -0.24561 | -0.3866 | -0.31613% (-0.3866 to -0.24561) | -0.31613 |
| Kazakhstan | -0.30465 | -0.21729 | -0.39193 | -0.30465% (-0.39193 to -0.21729) | -0.30465 |
| Bosnia and Herzegovina | -0.30015 | -0.23885 | -0.36142 | -0.30015% (-0.36142 to -0.23885) | -0.30015 |
| United States Virgin Islands | -0.2874 | -0.15537 | -0.41925 | -0.2874% (-0.41925 to -0.15537) | -0.2874 |
| Saint Kitts and Nevis | -0.27094 | -0.05593 | -0.48549 | -0.27094% (-0.48549 to -0.05593) | -0.27094 |
| Bahamas | -0.20575 | 0.02074 | -0.43172 | -0.20575% (-0.43172 to 0.02074) | -0.20575 |
| Zimbabwe | -0.18213 | -0.12882 | -0.23541 | -0.18213% (-0.23541 to -0.12882) | -0.18213 |
| Bermuda | -0.15195 | 0.06236 | -0.36579 | -0.15195% (-0.36579 to 0.06236) | -0.15195 |
| Montenegro | -0.13089 | -0.05921 | -0.20252 | -0.13089% (-0.20252 to -0.05921) | -0.13089 |
| Paraguay | 0.00122 | 0.08634 | -0.08383 | 0.00122% (-0.08383 to 0.08634) | 0.00122 |
| Saudi Arabia | 0.12596 | 0.24527 | 0.00678 | 0.12596% (0.00678 to 0.24527) | 0.12596 |
| Barbados | 0.15779 | 0.28868 | 0.02706 | 0.15779% (0.02706 to 0.28868) | 0.15779 |
| Syrian Arab Republic | 0.17618 | 0.42706 | -0.07407 | 0.17618% (-0.07407 to 0.42706) | 0.17618 |
| Oman | 0.34211 | 0.46474 | 0.21963 | 0.34211% (0.21963 to 0.46474) | 0.34211 |
| United States of America | 0.97021 | 1.33323 | 0.60848 | 0.97021% (0.60848 to 1.33323) | 0.97021 |

Supplementary Table16: ASPR by global asthma subregions and sex groups in 1990 and 2021

| **location** | **1990 ASPR Male** | **1990 ASPR Female** | **2021 ASPR Male** | **2021 ASPR Female** |
| --- | --- | --- | --- | --- |
| Caribbean | 8692.8 | 9603.26 | 7418.63 | 7820.02 |
|  | (7504.42 to 9927.18) | (8498.74 to 10763.08) | (6474.28 to 8440.14) | (6915.6 to 8750.8) |
| Oceania | 4054.69 | 5299.26 | 2390.36 | 3384.79 |
|  | (3695.77 to 4461.37) | (4855.4 to 5784.75) | (2176.04 to 2621.39) | (3139.32 to 3663.75) |
| Australasia | 12498.21 | 13260.68 | 7153.73 | 8296.66 |
|  | (11371.84 to 13765.58) | (12087.19 to 14656.82) | (5869.5 to 8581.33) | (7030.96 to 9807.43) |
| Southern Sub-Saharan Africa | 2871.38 | 3553.17 | 1707.58 | 2042.97 |
|  | (2497.96 to 3337.19) | (3058.18 to 4071.26) | (1500.21 to 1956.18) | (1776.96 to 2389.91) |
| Western Sub-Saharan Africa | 4213.61 | 5076.7 | 3271.49 | 3976.34 |
|  | (3657.14 to 4865.6) | (4477.63 to 5746.69) | (2837.93 to 3779.36) | (3514.96 to 4487.5) |
| North Africa and Middle East | 4871.44 | 5527.68 | 3263.94 | 3718.44 |
|  | (4296.33 to 5475.82) | (4942.92 to 6160.59) | (2846.99 to 3785.2) | (3328.06 to 4169.18) |
| Eastern Sub-Saharan Africa | 6204.49 | 6092.27 | 4247.8 | 4158.45 |
|  | (5412.21 to 7108.73) | (5380.89 to 6850.76) | (3672.89 to 4861.41) | (3670.94 to 4676.2) |
| Central Sub-Saharan Africa | 4017.45 | 4074.82 | 2666.12 | 2938.27 |
|  | (3499.57 to 4597.33) | (3594.65 to 4620.6) | (2342.14 to 3085.9) | (2622.8 to 3288.71) |
| Andean Latin America | 7023.78 | 7514.21 | 3957.73 | 4128.72 |
|  | (5743.55 to 8374.5) | (6212.47 to 8909.22) | (3136.51 to 5048.51) | (3282.89 to 5177.42) |
| Tropical Latin America | 7706.69 | 8014.91 | 4474.21 | 5006.94 |
|  | (6174.31 to 9532.22) | (6624.21 to 9762.54) | (3551.99 to 5710.32) | (4068.54 to 6146.52) |
| Southern Latin America | 6748.39 | 7893.91 | 5174.17 | 6817.78 |
|  | (5909.98 to 7749.34) | (6875.41 to 9023.03) | (4339.59 to 6213.48) | (5729.72 to 8055.34) |
| Central Latin America | 5547.98 | 5601.84 | 3071.56 | 3063.8 |
|  | (4645.31 to 6604.61) | (4760.82 to 6536.76) | (2466.43 to 3863.19) | (2556.46 to 3661.01) |
| Western Europe | 9468.59 | 10080.52 | 5043.42 | 6727.82 |
|  | (8178.95 to 10993.89) | (8729.87 to 11537.1) | (4152.02 to 6023.65) | (5602.11 to 7941.72) |
| Eastern Europe | 5236.8 | 5535.48 | 2525.81 | 2687.38 |
|  | (4461.88 to 6102.65) | (4748.41 to 6391.29) | (2027.81 to 3146.5) | (2195.55 to 3275.23) |
| Central Europe | 7262.15 | 7758.29 | 5437.1 | 5803.79 |
|  | (6318.75 to 8280.16) | (6721.32 to 8750.46) | (4493.85 to 6592.12) | (4927.12 to 6835.27) |
| Southeast Asia | 4860.13 | 4196.04 | 3269.27 | 2583.84 |
|  | (4330.21 to 5492.85) | (3758.81 to 4699.59) | (2902.71 to 3657.92) | (2304.81 to 2881.44) |
| South Asia | 4234.62 | 3677.62 | 2311.83 | 2214.69 |
|  | (3563.87 to 4991.32) | (3142.56 to 4266.36) | (2009.92 to 2686.03) | (1939.86 to 2512.8) |
| East Asia | 3297.58 | 3070.11 | 2227.27 | 1833.91 |
|  | (2742.32 to 4045.47) | (2595.31 to 3700.97) | (1793.5 to 2836.51) | (1481.14 to 2296.42) |
| Central Asia | 3083.1 | 3597.99 | 2340.83 | 2783.1 |
|  | (2673.37 to 3547.67) | (3166.54 to 4049.56) | (1996.36 to 2755.06) | (2415.84 to 3195.34) |
| High-income Asia Pacific | 8287.42 | 8563.75 | 2979.25 | 3634.27 |
|  | (7323.4 to 9470.59) | (7436.02 to 9842.26) | (2433.35 to 3682.64) | (2968.46 to 4419.91) |
| High-income North America | 8856.18 | 10256.13 | 8808.07 | 10551.66 |
|  | (7466.23 to 10796.7) | (8736.33 to 12074.98) | (7621.15 to 10369.82) | (9256.11 to 12038.6) |

Supplementary Table 17: ASIR by global asthma subregions and sex groups in 1990 and 2021

| **location** | **1990 ASIR Male** | **1990 ASIR Female** | **2021 ASIR Male** | **2021 ASIR Female** |
| --- | --- | --- | --- | --- |
| Caribbean | 1277.6 | 1319.75 | 1180.68 | 1204.16 |
|  | (1033.46 to 1593.92) | (1119.18 to 1588.28) | (970.08 to 1450.55) | (1020.1 to 1441.82) |
| Oceania | 663.86 | 765.51 | 435.76 | 533.29 |
|  | (569.66 to 773.18) | (664.63 to 885.07) | (378.85 to 508.44) | (472.14 to 599.58) |
| Australasia | 869.05 | 658.6 | 662.99 | 508.04 |
|  | (671.62 to 1104.12) | (559.48 to 782.32) | (494.07 to 886.54) | (411.13 to 646.39) |
| Southern Sub-Saharan Africa | 514.48 | 587.39 | 313.66 | 367.42 |
|  | (420.75 to 636.83) | (482.84 to 730.14) | (257.42 to 388.22) | (297.48 to 459.87) |
| Western Sub-Saharan Africa | 702.78 | 744.38 | 586.92 | 620.62 |
|  | (575.24 to 872.62) | (627.4 to 910.03) | (475.35 to 727.08) | (523.44 to 753.44) |
| North Africa and Middle East | 735.06 | 738.83 | 535.97 | 546.76 |
|  | (618.06 to 888.46) | (639.37 to 863.43) | (444.09 to 654.05) | (469.01 to 653.79) |
| Eastern Sub-Saharan Africa | 949.03 | 892.3 | 701.07 | 672.97 |
|  | (779.67 to 1181.58) | (755.11 to 1078.38) | (578.9 to 862.14) | (561.36 to 816.65) |
| Central Sub-Saharan Africa | 663.17 | 663.45 | 469.86 | 500.99 |
|  | (550.16 to 817.66) | (559.59 to 802.88) | (390.46 to 575.55) | (422.44 to 588.75) |
| Andean Latin America | 1216.62 | 1216.91 | 773.08 | 771.52 |
|  | (955.45 to 1615.44) | (971.07 to 1545.16) | (577.84 to 1024.29) | (593.29 to 991.87) |
| Tropical Latin America | 1326.83 | 1348.87 | 859.96 | 927.53 |
|  | (1042.83 to 1729.74) | (1079.69 to 1724.17) | (641.06 to 1185.7) | (719.64 to 1200.54) |
| Southern Latin America | 711.83 | 694.37 | 663.53 | 671.84 |
|  | (588.16 to 895.3) | (572.9 to 842.32) | (518.4 to 860.44) | (541.46 to 847.19) |
| Central Latin America | 948.29 | 882.78 | 585.59 | 547.43 |
|  | (754.95 to 1214.75) | (733.39 to 1071.91) | (453.09 to 765.06) | (439.2 to 691.57) |
| Western Europe | 629.71 | 669.89 | 467.69 | 529.49 |
|  | (519.67 to 766.71) | (574.81 to 785.45) | (365.25 to 604.02) | (433.69 to 656.44) |
| Eastern Europe | 733.48 | 737.72 | 456.57 | 461.57 |
|  | (600.67 to 917.14) | (611.19 to 910.09) | (354 to 606.1) | (362.91 to 594.29) |
| Central Europe | 897.72 | 891.27 | 923.9 | 870.84 |
|  | (757.2 to 1089.27) | (755.41 to 1054.49) | (727.42 to 1198.96) | (714.01 to 1082.35) |
| Southeast Asia | 772.69 | 629.18 | 546.57 | 420.35 |
|  | (651.27 to 932.93) | (539.83 to 749.74) | (457.47 to 656.15) | (360.46 to 501.57) |
| South Asia | 675.34 | 582.87 | 366.39 | 349.59 |
|  | (535.02 to 867.91) | (473.6 to 737.05) | (300.64 to 460.43) | (293.19 to 431.51) |
| East Asia | 570.81 | 492.33 | 412.65 | 329.25 |
|  | (457.18 to 735.1) | (399.92 to 625.35) | (320.22 to 557.49) | (257.33 to 439.65) |
| Central Asia | 460.51 | 492.33 | 392.36 | 425.12 |
|  | (387.77 to 566.8) | (415.71 to 600.67) | (324.14 to 492.2) | (351.61 to 525.36) |
| High-income Asia Pacific | 932.24 | 890.49 | 484.7 | 528.96 |
|  | (772.1 to 1147.56) | (747.62 to 1074.58) | (375.15 to 644.17) | (416.55 to 679.63) |
| High-income North America | 1384.11 | 1460.55 | 1394.94 | 1404.51 |
|  | (1074.09 to 1827.3) | (1186.55 to 1865.5) | (1098.83 to 1816.79) | (1168.74 to 1724.21) |

Supplementary Table 18: ASIR by global asthma subregions and sex groups in 1990 and 2021

| **location** | **1990_ASYR_Male** | **1990_ASYR_Female** | **2021_ASYR_Male** | **2021_ASYR_Female** |
| --- | --- | --- | --- | --- |
| Caribbean | 346.98 | 379.09 | 296.84 | 308.74 |
|  | (218.84 to 519.95) | (236.74 to 553.7) | (185.89 to 442.67) | (196.15 to 451.69) |
| Oceania | 158.21 | 204.86 | 93.62 | 130.76 |
|  | (104.27 to 228.11) | (132.38 to 290.5) | (59.78 to 136.5) | (84.51 to 185.31) |
| Australasia | 492.78 | 516.81 | 285.27 | 325.43 |
|  | (320.06 to 712.16) | (337.44 to 743.4) | (177.39 to 428.95) | (206.82 to 484.15) |
| Southern Sub-Saharan Africa | 112.99 | 138.9 | 66.88 | 79.37 |
|  | (73.31 to 170.13) | (88.28 to 208.54) | (43.26 to 100.49) | (50.89 to 119.62) |
| Western Sub-Saharan Africa | 164.95 | 195.87 | 129.21 | 154.1 |
|  | (107.32 to 248.68) | (126.51 to 290.83) | (82.82 to 193.53) | (99.5 to 230.12) |
| North Africa and Middle East | 191.58 | 214.11 | 128.56 | 143.44 |
|  | (122.61 to 283.34) | (136.7 to 310.6) | (80.7 to 193.34) | (91.53 to 206.86) |
| Eastern Sub-Saharan Africa | 243.94 | 238.18 | 167.99 | 163.5 |
|  | (157.63 to 362.96) | (150.36 to 352.36) | (107.67 to 253.91) | (105.75 to 242.8) |
| Central Sub-Saharan Africa | 157.12 | 157.73 | 104.77 | 114.28 |
|  | (100.6 to 232.84) | (100.76 to 232.71) | (66.54 to 156.82) | (74.44 to 167.66) |
| Andean Latin America | 282 | 300.41 | 159.53 | 165.26 |
|  | (170.63 to 421.43) | (181.05 to 447.46) | (95.93 to 251.32) | (100.11 to 250.27) |
| Tropical Latin America | 306.98 | 316.54 | 179.2 | 197.81 |
|  | (190.07 to 468.96) | (196.67 to 482.53) | (108.91 to 278) | (121.54 to 300.29) |
| Southern Latin America | 264.46 | 306.7 | 204.09 | 265.15 |
|  | (169.73 to 387.9) | (198.64 to 444.91) | (127.35 to 310.36) | (168.93 to 396.12) |
| Central Latin America | 220.73 | 221.36 | 123.26 | 121.66 |
|  | (140.39 to 329.17) | (137.91 to 326.75) | (75.11 to 186.87) | (74.23 to 180.74) |
| Western Europe | 371.47 | 391.5 | 200.45 | 263.16 |
|  | (235.21 to 548.04) | (250.65 to 568.37) | (123.25 to 300.68) | (164.55 to 382.91) |
| Eastern Europe | 204.91 | 215.01 | 100.84 | 106.05 |
|  | (130.25 to 298.79) | (136.44 to 313.36) | (61.48 to 152.48) | (64.98 to 161.27) |
| Central Europe | 280.81 | 299.81 | 216.7 | 228.3 |
|  | (177.11 to 410.78) | (190.28 to 439.11) | (135.87 to 328.28) | (143.69 to 338.93) |
| Southeast Asia | 191.15 | 164.03 | 129.19 | 101.31 |
|  | (122.19 to 283.3) | (103.8 to 241.73) | (83.24 to 195.61) | (64.07 to 150.29) |
| South Asia | 164.2 | 140.77 | 89.45 | 84.46 |
|  | (106.81 to 249.78) | (90.28 to 212.13) | (56.81 to 133.57) | (54.41 to 124.84) |
| East Asia | 130.54 | 120.5 | 88.88 | 72.85 |
|  | (82.49 to 196.11) | (76.54 to 178.03) | (55.11 to 136.3) | (44.89 to 110.35) |
| Central Asia | 120.9 | 139.6 | 92.39 | 108.39 |
|  | (77.12 to 178.59) | (90.23 to 205.82) | (58.49 to 137.81) | (69.81 to 161.08) |
| High-income Asia Pacific | 322.35 | 333.55 | 118.57 | 143.93 |
|  | (205.39 to 474.12) | (212.91 to 486.97) | (74 to 180.43) | (89.67 to 214.53) |
| High-income North America | 350.13 | 401.5 | 348.97 | 410.3 |
|  | (227.97 to 527.83) | (256.54 to 587.14) | (227.95 to 528.08) | (267.43 to 605.38) |

ASPR: age-standardized prevalence rate. EAPC: estimated annual percentage change. ASIR: age-standardized incidence rate. ASYR: age-standardized YLDs rate. YLDs: incidence and years of disability.


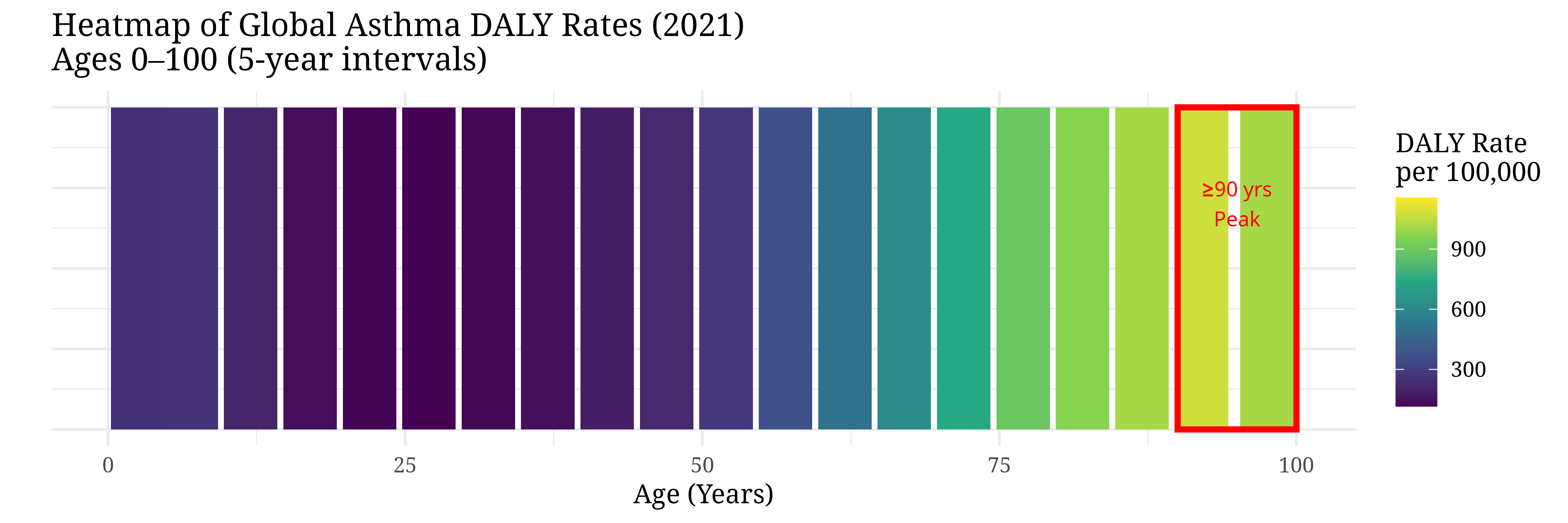


Supplemental Figure 1 The heatmap of DALY rates for ages 0-100 (in groups of 5 years old) shows that the darker the yellow color, the higher the DALY rate, and the darker the purple color, the lower the DALY rate.





**Supplemental Figure 2** A. Comparative analysis of global risk factors Comparative analysis of risk factors in regions with different levels of SDI (B. High SDI, C. High middle SDI, D. Middle SDI, E. Low middle SDI, and F. Low SDI).


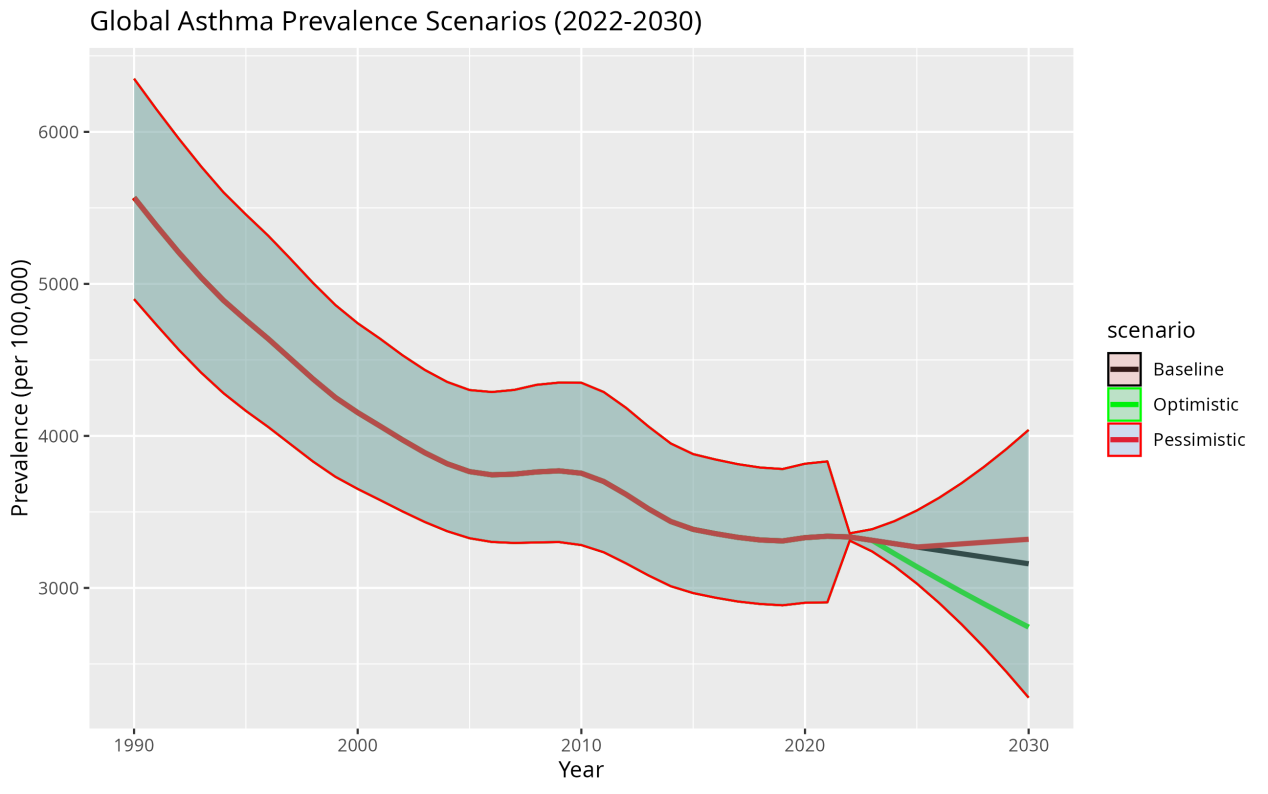


**Supplemental Figure 3** Global Asthma Prevalence Scenarios Simulation. The shaded area represents the 95% confidence interval (CI) for each scenario
